# Effects of local heat on metabolic health, frailty risk, and exercise adaptations in pre-diabetic older adults: Protocol for the Heat and Exercise in Aging as Therapy (HEAT) clinical trial

**DOI:** 10.1371/journal.pone.0351577

**Published:** 2026-06-15

**Authors:** Hui-Ying Luk, Casey R. Appell, Fangyuan Zhang, Jarrod Blinch, K. Sreekumaran Nair, Chwan-Li Shen, Danielle E. Levitt

**Affiliations:** 1 Applied Exercise Physiology Laboratory, Department of Kinesiology and Sport Management, Texas Tech University, Lubbock, Texas, United States of America; 2 Department of Mathematics and Statistics, Texas Tech University, Lubbock, Texas, United States of America; 3 Perception, Cognition, and Action Laboratory, Department of Kinesiology and Sport Management, Texas Tech University, Lubbock, Texas, United States of America; 4 Division of Endocrinology, Diabetes, Metabolism and Nutrition, Mayo Clinic, Rochester, Minnesota, United States of America; 5 Department of Pathology, Texas Tech University Health Sciences Center, Lubbock, Texas, United States of America; 6 Metabolic Health and Muscle Physiology Laboratory, Department of Kinesiology and Sport Management, Texas Tech University, Lubbock, Texas, United States of America; Université de Lille: Universite de Lille, FRANCE

## Abstract

**Introduction:**

Glycemic dysregulation is a hallmark of type 2 diabetes (T2D) and contributes to skeletal muscle (SKM) loss and frailty risk, especially in older adults. Glycemic control and physical function are supported by SKM capillarization and mitochondrial function, and their impairment contributes to T2D development. While high-intensity interval training (HIIT) is a promising intervention, adherence and effectiveness remain concerns for prescribing HIIT among older adults at risk for T2D. Local heat therapy (LHT) may be a more practical initial strategy to improve SKM architectural factors and precondition SKM, enhancing physiological adaptations to exercise in this population.

**Methods and analysis:**

Heat and Exercise in Aging as Therapy (HEAT) is a two-phase, randomized, sham-controlled clinical trial investigating the efficacy of LHT to improve glycemic control and decrease frailty risk via improved SKM architecture among older adults with prediabetes. LHT is tested as a standalone intervention and as a means to precondition SKM for subsequent HIIT, improving exercise adaptations. In Phase 1, LHT and sham (CON) groups apply heat pads for 90 minutes/day, 6 days/week, for 12 weeks. A separate HIIT group completes 4x4-minute cycling intervals at 90–95% VO₂peak, 3 days/week. In Phase 2, LHT and CON groups begin HIIT. Participants (≥50 years) have impaired fasting glucose (100–125 mg/dL) and/or HbA1c (5.7–6.4%). Biospecimen collection and clinical assessments occur at baseline (T1), after Phase 1 (T2), and Phase 2 (T3). To our knowledge, this is the first study to determine the use of local heat pad on pre-diabetic older population. If successful, LHT may be a practical, scalable, non-invasive intervention to improve glycemic control and reduce frailty risk in older adults with prediabetes, preventing progression to T2D.

## Introduction

Skeletal muscle (SKM) metabolic function is dysregulated in type 2 diabetes mellitus (T2D). This dysregulation is associated with changes in muscle architecture which contribute to phenotypic frailty including slow gait speed, weakness, unintentional weight loss, fatigue, and low physical activity levels [1]. Glycemic dysregulation (e.g., insulin resistance) is a hallmark of T2D and contributes to SKM loss in adults with diabetes [2]. The pathogenic development of insulin resistance in T2D is multifaceted and includes, but is not limited to, mitochondrial dysfunction [3,4], decreased capillarization [5,6], and decreased SKM mass [7,8]. Evidence suggests that capillary networks and mitochondria-mediated metabolic alterations occur before the development of overt T2D; thus, it is critical to explore potential therapies in individuals with prediabetes to decrease T2D risk.

Exercise training is a key strategy to manage metabolic disease and decrease T2D risk [9]. While different exercise modalities improve SKM health across age groups, high intensity interval training (HIIT) can improve capillary density [10,11], mitochondrial content and function [12–14], and SKM mass [15] in older adults and those with T2D and obesity. However, exercise adherence is low [9,16,17]. In older adults at risk for T2D, compromised oxygen delivery and mitochondrial dysfunction [18] promote exercise intolerance, a factor contributing to low exercise adherence [19]. Alternative means to manage metabolic disease and improve metabolic health are needed, and heat therapy is a possible solution.

Repeated exposure to whole-body and local (e.g., thigh) heat therapy (LHT) improve mitochondrial biogenesis, muscle capillarization, glycemic control, and increase whole-muscle and muscle fiber cross-sectional area (CSA) [20]. Of particular interest, repeated whole-body heat therapy and moderate-intensity continuous exercise training in young, sedentary adults similarly increased SKM capillary density, capillary-fiber perimeter exchange index, aerobic capacity, and insulin sensitivity [21]. Moreover, whole-body heat therapy can decrease fasting glucose and insulin levels in sedentary, overweight men exposed to temperatures of 34–39°C for 1–6 hrs across 10 sessions and in obese rats exposed to 38°C for 30 minutes, three times per week, for 12 weeks [22–25]. Recent evidence shows that repeated exposure to local (e.g., thigh) and whole-body heat therapy alone improves mitochondrial biogenesis (i.e., PGC-1α and respiratory chain complex expression) [26], muscle capillarization (e.g., vascular endothelial growth factor [VEGF], angiopoietin 1, and capillary density) [20,27,28], glycemic control (e.g., fasting glucose and insulin), and increases whole muscle and muscle fiber cross-sectional area (CSA). Mechanistically, heating the thighs alone using hot water (48–52°C) perfused through tubing to raise leg skin temperature to 39–40°C without altering core temperature increased VEGF, angiopoietin 1, and heat shock protein (HSP) mRNA expression [29]. These data suggest that heat therapy could be an effective treatment to improve glycemic control and metabolic disease-induced muscle atrophy, thus reducing the risk of T2D and frailty.

The magnitude of angiogenic adaptations to local heat therapy (LHT) can be similar to whole-body heating [29], providing a foundation to support examination of the therapeutic potential of LHT. Although it has not been examined in individuals with prediabetes, a single lower body exposure to hot water circulation at 43°C for 90 min in patients with peripheral artery disease tended to improve walking performance [30]. Given these promising initial findings, LHT may be useful to improve muscle architecture (i.e., capillarization, mitochondria, muscle fiber CSA). Such changes could improve exercise tolerance by preconditioning the microenvironment of SKM, likely enhancing exercise adherence and improving subsequent exercise adaptations. Therefore, changes in the SKM with LHT alone, and with exercise training following LHT, may improve glycemic control and physical function, thereby decreasing risk for T2D and frailty, in older individuals.

Strong evidence supports the benefits of heat therapy on SKM and glycemic control. However, the efficacy of LHT to decrease risk for T2D and frailty in older adults with prediabetes has not been examined. Therefore, we developed the Heat and Exercise in Aging as Therapy (HEAT) study. This randomized, controlled clinical trial aims to evaluate the effectiveness of LHT among older adults with prediabetes using a two-phased approach. The aim of Phase 1 is to compare the effectiveness of LHT alone versus HIIT to improve muscle architecture, glycemic control, exercise capacity, and risk of frailty (Aim 1). The aim of Phase 2 is to assess whether a period of LHT preceding the initiation of HIIT improves exercise responsiveness, resulting in further improvements in SKM architecture, glycemic control, and risk of frailty (Aim 2). Given the practicality and ease of LHT application, findings can be easily translated to clinical recommendations.

## Methods

### Study overview

The Heat and Exercise in Aging as Therapy (HEAT) clinical trial is a two-phase, randomized, sham-controlled, parallel-design study examining the effects of LHT alone, and LHT applied prior to HIIT, on SKM architecture, glycemic regulation, frailty risk, and exercise capacity in older adults with prediabetes (Clinical Trial NCT06580964 [https://clinicaltrials.gov/study/NCT06580964]). Eligible participants are randomized to one of three intervention groups for Phase 1 of the study: HIIT alone (HIIT group), LHT followed by HIIT (LHT group), or sham heat pad followed by HIIT (control [CON] group; thermoneutral temperature).

**Fig 1:**
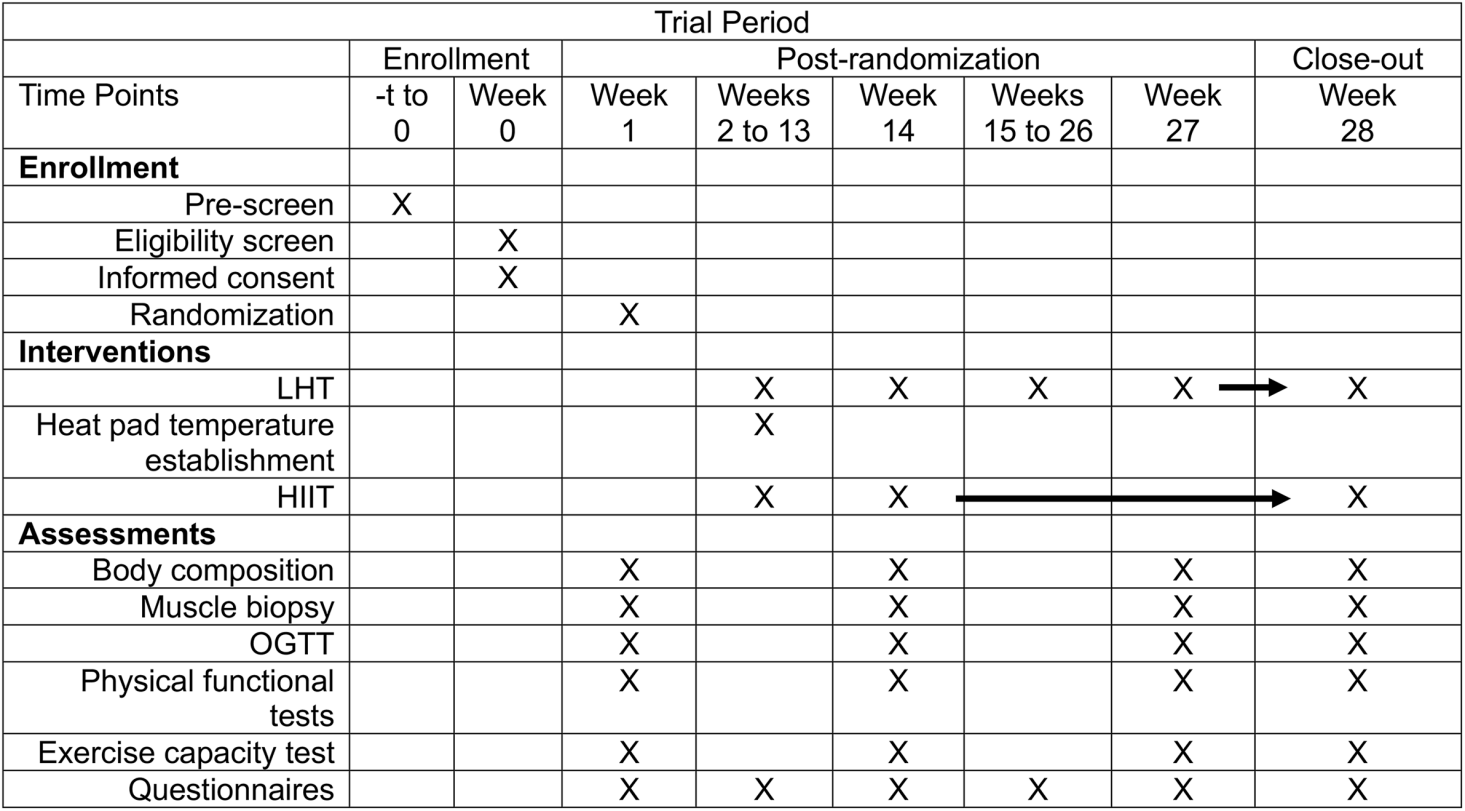
The schedule of enrollment, interventions, and assessments.

**Fig 2:**
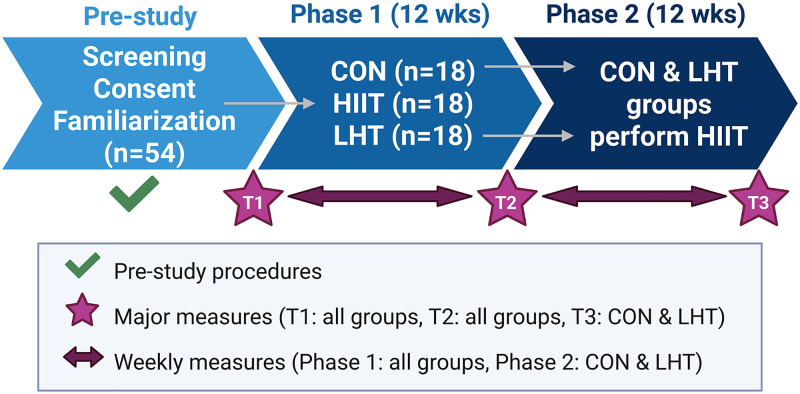
An overview of the study design.

All primary outcome measures are assessed at baseline (timepoint [T]1), after Phase 1 (T2), and after Phase 2 (T3). Phase 1 consists of 12 weeks of the LHT intervention for the LHT and CON groups, or 12 weeks of high-intensity interval training for the HIIT group. Following T2, only participants in the LHT and CON groups progress to Phase 2, which involves 12 weeks of high-intensity interval training. Participants assigned to the HIIT group conclude their participation in the study after T2. The schedule of enrollment, interventions, and assessments is presented in **Fig 1**, in accordance with SPIRIT guidelines (**S1 Table**). An overview of the study design is presented in **Fig 2**. This study is approved by the Texas Tech University Institutional Review Board (IRB #2024-365) (**S1 File**). Any future modifications to the protocol will be submitted for IRB review and approval prior to implementation, reported to the study safety officer, and documented in the manual of procedures. The first participant will be enrolled on July 31^st^, 2025. Participant enrollment is expected to conclude by July 2028, data collection will be completed by February 2029, and study results are anticipated by December 2029.

### Power analysis

A power analysis was performed using G*Power (version 3.1.9.4). Because capillary-fiber perimeter exchange index (CFPE) is a widely measured variable and an important component of anticipated adaptations, we used the results from Kim et al.[27] to power this study. They observed significantly greater CFPE with LHT vs CON (d > 1.5). We used a more conservative, d = 1.2 (reduced by 20%), because older adults might have a blunted response to LHT. We used the statistical analysis of Aim 1 to power the study because it involves more groups (three) and thus requires the largest number of participants. The one-way ANOVA with three groups (d = 1.2, f = 0.4899, α = 0.05) will require 45 total participants (15 per group) to achieve 80% power. This will be followed by two non-orthogonal planned contrasts (CON vs. LHT, LHT vs. HIIT). These *t*-*t*ests (d = 1.2, α = 0.05/2 [Bonferroni]) also require 15 participants per group to achieve 80% power. In the statistical analysis of Aim 2, only two of the three groups are included, leading to greater statistical power. The planned *t*-*t*est (d = 1.2, a = 0.05, 15 participants per group) will achieve 89% power. If needed, the one-way ANOVA with two groups (d = 1.2, a = 0.05, 15 participants per group) will also achieve 89% power. Comparing this number of participants per group to previously published work examining LHT and SKM mitochondria, capillarization, and size [26–28], 15 participants per group is a comparable estimate of sample size. Although not identical to our proposed work, sample sizes of 10 (within-subjects) [23] and 18 (9 per group, between-subjects) [31] were sufficient to detect improvements in glycemic control in overweight men and in obese women with polycystic ovarian syndrome, respectively, in response to whole-body heat therapy. Our power analysis and previously published research support a target sample size of 15 participants per group (CON, HIIT, and LHT). Accounting for a potential 18% attrition rate, we aim to enroll 18 participants per group (9 men, 9 women) for a total target enrollment of 54 participants.

### Settings

This study takes place in the Department of Kinesiology and Sport Management facility at Texas Tech University in Lubbock, TX, USA. This is an academic research setting equipped with exercise, clinical, and biochemical laboratories.

### Recruitment, consent, screening, and group allocation

Potential participants are invited to voluntarily join the study. Recruitment efforts include posting study flyers in local pharmacies, supermarkets, and at relevant community events, and posting electronic advertisements on listservs and social media. Social media announcements specify targeting older adults (50+) in the Lubbock, TX area.

#### Initial phone pre-screening.

Potential participants undergo telephone pre-screening to determine preliminary eligibility. During this pre-screening, participants must confirm being informed by a physician that they have prediabetes, may be at risk for developing diabetes, or exhibit elevated blood glucose levels. Additional eligibility criteria include leading a sedentary lifestyle [32,33], absence of medical conditions that could limit participation in HIIT, no heavy alcohol consumption, no use of nicotine or cannabis products, and not currently taking medications known to influence intervention outcomes (**Table 1**).

**Table 1:** Inclusion and exclusion criteria for the HEAT study.

Inclusion	Exclusion
• Age ≥ 50 years of age • Structured exercise < 30 minutes, 3x/week • Body mass ≥ 110 lbs • Fasting blood glucose 100–125 mg/dL and hemoglobin A1c 5.7–6.4% • Consume <8 (women) or <15 (men) alcohol-containing beverages per week • Do not use nicotine or cannabis • Do not take any medications that could interfere with responses to the interventions (e.g., corticosteroids, opiates, benzodiazepines, tricyclic antidepressants, beta blockers, sulfonylureas, insulin, metformin, anticoagulants, barbiturates, insulin sensitizers, fibrates, immunosuppressants)	• History of peripheral neuropathies • Medical complications that could contraindicate participation in the high intensity interval training intervention including: orthopedic complications that would limit your ability to perform cycling exercise, significant cardiovascular impairments (e.g., history of arrhythmias, severe uncontrolled hypertension, etc.), diagnosed metabolic disease (e.g., diabetes), renal disease, sickle cell anemia, or cancer in remission for <6 months • Known history of slow wound healing • Allergy to lidocaine or latex • Pregnancy • > 1.5” subcutaneous fat over the thigh muscle. • Symptoms suggestive of cardiovascular, respiratory, metabolic, or renal diseases including discomfort, pressure, or pain in your chest, neck, jaw, arms, calves, or other areas potentially related to ischemia; shortness of breath at rest or with mild exertion; dizziness or fainting (syncope); difficulty breathing while lying flat (orthopnea) or sudden nighttime breathing difficulties (paroxysmal nocturnal dyspnea) • Palpitations or rapid heartbeat (tachycardia); pain or cramping in legs during physical activity (intermittent claudication); a known heart murmur; swelling in ankles (edema); unusual fatigue or shortness of breath during routine activities or at rest

#### Consent/screening visit.

After pre-screening, potential eligible participants are invited for a consent and screening visit. Participants are asked to fast (no food or drink except water) for at least 12 hours before this visit. After a thorough explanation of the study procedures, risks, and benefits by the clinical research coordinator, participants must demonstrate understanding of the study before providing written informed consent. Following the written informed consent, blood pressure is measured twice, with a third measurement if either systolic (SBP) or diastolic (DBP) blood pressure differs by >5 mmHg between the first two measurements [34]. Fasting blood glucose (FBG) and hemoglobin A1c (HbA1c) levels are measured to confirm fasting dysglycemia (FBG 100–125 mg/dL) and/or elevated HbA1c (5.7–6.4%), indicative of prediabetes [35]. Then, they complete medical and physical activity history questionnaires (International Physical Activity Questionnaire [IPAQ] [36]). Full inclusion and exclusion criteria are listed in **Table 1**. Participants receive compensation for all testing procedures, exercise sessions, and weekly laboratory visits.

#### Randomization and stratification.

Participants are stratified based on sex assigned at birth (male, female), age brackets (e.g., 50–59, 60–69 years, 70–79, etc.), and glucose tolerance (glucose concentration at the 1-hour blood draw following ingestion of 75 g glucose: < 155 mg/dL, normal glucose tolerance; 155 to <209 mg/dL, intermediate hyperglycemia; and ≥209, potential T2D) [37] at baseline. A permuted block randomization method [38] with a block size of three (1:1:1 ratio) is used within each stratum to ensure balanced allocation among groups (CON, HIIT, LHT). Accordingly, an allocation table is used to randomize participants to groups. Group assignments are recorded in a separate randomization form, accessible only to a designated unblinded investigator, on the HIPAA-compliant Research Electronic Data Capture (REDCap)2 database, adhering to NIH guidelines for clinical trials. This balanced permuted block randomization can ensure baseline comparability between treatment groups, allow further analysis of treatment effects for different subgroups, and maximize statistical power.

#### Blinding and unblinding.

A designated unblinded investigator is responsible for ensuring appropriate randomization in REDCap2. The statistician and additional investigators will remain blind to group assignments until all analyses are complete, except in specific circumstances. The clinical research coordinator and other study staff receive only the information necessary to carry out the intervention (e.g., assignment to HIIT or one of the heat pad therapy groups). Staff members working with participants assigned to heat pad groups (CON or LHT) must monitor and adjust heat pad settings as needed based on intramuscular (IM) temperature measurement, monitor in-laboratory heat pad sessions, and communicate the heat pad’s target temperature to the participant. Study staff will remain blinded to group assignments and/or heat pad temperature settings for participants under the care of other team members, with exceptions made only when necessary (e.g., staff reassignment due to illness, etc.). Investigators performing IM temperature measurement procedure become aware of whether participants are assigned to one of the heat pad groups (CON or LHT) since only these groups undergo this procedure. As all participants perform HIIT during one of the two study phases, knowledge of a participant performing HIIT does not reveal initial group assignment.

The study team maintains participant blinding to the extent possible. Following T1 testing and randomization, participants are informed of their assignment to a heat pad therapy group or to HIIT. However, those assigned to a heat pad group are not informed about assignment to CON or LHT; instead, they are informed that the heat pad could be set to maintain muscle temperature up to approximately 40°C.

To protect blinding, randomization codes are accessible to only the designated unblinded investigator. Coded labels, rather than descriptive condition names, are used during testing and in datasets provided to the statistician. Additionally, testing and assessments are performed in the same manner for all participants, regardless of group assignment. Unblinding of LHT versus CON assignments will occur only if a serious adverse event requiring medical intervention occurs or after data collection for the entire study is complete. If any unblinding occurs outside of these predefined parameters, the event will be documented in a secure log, including the reason for unblinding, the personnel involved, and the potential impact on study integrity. Such events will also be reported to the NIH and the Institutional Review Board, as required.

### Testing visits

#### Primary outcomes testing.

Testing for primary outcomes will be conducted at T1, T2, and T3. Each testing period will occur over two separate days. Day 1 includes a muscle biopsy and an oral glucose tolerance test (OGTT), and Day 2 includes assessments of physical function and exercise capacity. There will be 2–3 days between Testing Days 1 and 2. At T2 and T3, testing will begin 2 days following the last intervention session of each respective phase. For each testing visit (i.e., Day 1 and Day 2), participants will be instructed to refrain from certain medications (e.g., acetaminophen, NSAIDs) for 48 hours, avoid alcohol consumption for 24 hours, and maintain a 12-hour fast (nothing to eat or drink except water) prior to their scheduled assessments.

### Day 1

#### Arrival procedures.

On Day 1 of primary outcomes testing, hydration status (urine specific gravity [USG], urine color, and thirst sensation) are assessed upon participant arrival. Participants confirm adherence to a 12-hour overnight fast (water only) and abstinence from exercise, alcohol, and non-steroidal anti-inflammatory drugs (NSAIDs) for at least 24 hours. Then, they provide a urine sample to verify hydration status via urine refractometry, with adequate hydration defined as urine specific gravity (USG) <1.020 [39,40]. Visual assessment of urine color against a standardized chart [41] and evaluation of thirst sensation using a validated thirst scale [42] complement USG for hydration assessment. At least 8 oz of water are provided if participants are insufficiently hydrated, and hydration status is reassessed after 15 minutes. This procedure is repeated until adequate hydration is achieved. Subsequently, blood pressure, height, body mass, waist and hip circumferences, body composition via dual-energy X-ray absorptiometry (DXA; GE Lunar Prodigy, Madison, WI) will be measured and muscle sampling, and OGTT will be performed. These assessments are conducted at T1 and T2 for all groups, with an additional assessment at T3 for participants in the CON and LHT groups. For T2 and T3, these assessments are conducted at least 48-hour following the final session of Phase 1 and Phase 2, respectively.

#### Anthropometric measurements.

Prior to data collection, all anthropometric equipment is calibrated according to the manufacturers’ specifications. A digital weighing scale with integrated stadiometer (500KL, Health o Meter Professional, McCook, IL, USA) is used to assess standing height and body mass. Participants are instructed to wear lightweight clothing and to remove shoes, heavy outer garments, and items from their pockets before measurement.

Height is measured with participants standing barefoot, facing away from the stadiometer, with feet flat on the scale platform, heels together, arms relaxed at their sides, and the head positioned in the Frankfort horizontal plane. The heels, buttocks, and upper back are positioned in light contact with the stadiometer without hyperextension. Once positioned, the headpiece is lowered to make contact with the scalp, and participants are instructed to inhale deeply and stand as tall as possible without lifting their heels. Height is recorded to the nearest 0.1 cm.

Body mass is measured using a digital weighing scale. Participants stand in the center of the scale platform with their weight evenly distributed across both feet and remain still until the reading stabilizes. Body mass is recorded to the nearest 0.1 kg.

#### Waist and hip circumference measurements.

Waist and hip circumferences are measured using a flexible, non-elastic tape measure following standardized anthropometric protocols. All measurements are taken with the participant standing and recorded to the nearest 0.1 cm.

For waist circumference, participants stand upright with feet shoulder-width apart and arms relaxed at their sides. They are instructed to breathe normally and to keep their abdominal muscles relaxed throughout the procedure. The waist measurement is taken at the narrowest part of the torso, typically located midway between the inferior margin of the last palpable rib and the superior border of the iliac crest. If the narrowest point is not easily identifiable, participants are asked to place a finger on their umbilicus, and the measurement is taken just above that point. If this alternative method is used, it is documented and applied consistently for all subsequent measurements. The tape measure is wrapped horizontally around the waist at the identified site, ensuring it is snug against the skin but not compressing it. The tape is kept parallel to the floor during the measurement. Participants are asked to exhale gently, and the measurement is taken at the end of the exhalation.

For hip circumference, participants remain standing with feet together and arms relaxed. The measurement is taken at the point of greatest gluteal protrusion, which typically corresponds to the level of the greater trochanter of the femur. The tape is wrapped around the hips at this level, ensuring it is snug but not compressing the skin, and remains parallel to the floor. The waist-to-hip ratio (WHR) is calculated by dividing the waist circumference by the hip circumference.

#### Dual-energy X-ray absorptiometry (DXA) scanning procedures.

Whole-body scans are performed using a DXA scanner (GE Lunar Prodigy, Madison, WI) to assess body composition. All scans follow standardized procedures to ensure data quality and participant safety. Participants are instructed to lie supine on the DXA scanner bed with the body fully extended and aligned along the center axis of the scanning field. The head, torso, arms, and legs are positioned to prevent overlap, with the entire body supported by the scanning surface. Arms are placed alongside the body, with palms facing downward and not in contact with the torso. Legs remain straight with feet slightly apart. The participant’s body is fully contained within the white boundary box of the scanner bed.

Proper alignment is confirmed by ensuring the head is centered and free of rotation. A foam roll or positioning block is placed under the knees to enhance comfort and reduce lower back arching. Additional positioning aids (e.g., foam wedges or straps) are used as needed to minimize movement during scanning.

Prior to initiating the scan, participants are instructed to remain still and breathe normally throughout the procedure to prevent motion artifacts. The scanner is set to the appropriate whole-body mode based on the study protocol. The scan is initiated in accordance with the manufacturer’s operating instructions. Scan duration typically ranges from 7 to 12 minutes, depending on the model of the scanner and participant body size.

#### Muscle biopsy.

Following assessment of body composition, a SKM sample is collected using sterile technique and a suction-modified Bergström biopsy needle [43]. The biopsy leg is randomized and counterbalanced across groups. At each testing time point, muscle biopsies will be obtained from a distinct site on the *vastus lateralis* (VL), each separated by 3 cm. The primary biopsy site (used at T1) is located at the anatomical midpoint of the vastus lateralis, defined as the midpoint between the greater trochanter and the lateral border of the patella. The second site (used at T2) is located 3 cm proximal to this midpoint, and the third site (used at T3; LHT and CON groups only) is located 3 cm distal to the midpoint. After disinfecting the area with povidone-iodine, lidocaine without epinephrine is injected subcutaneously at the incision site above the VL, followed by a deeper injection directly into the VL muscle beneath the incision site. A small incision is then made using a #11 scalpel blade to penetrate the skin and fascia over the VL muscle. A suction-modified Bergström needle (5 mm) inserted through this entry point is used to collect approximately 200 mg of VL muscle tissue. Muscle is then quickly cleaned of excess blood and fat, and portioned into sections that will be flash frozen for protein and enzyme analyses, OCT-embedded and frozen in liquid nitrogen-cooled isopentane for histochemistry, formalin-fixed (zinc-buffered formalin; Z-fix) for histochemistry, or fixed in 2.5% glutaraldehyde + 1% paraformaldehyde in phosphate buffer, pH 7.4 for electron microscopy. Immediately following the biopsy, 10 minutes of direct pressure is applied, followed by incision site closure using an adhesive bandage. Pressure dressing is applied over the bandage and participants are instructed to leave this dressing on for at least three hours. Research staff provide participants with biopsy care instructions and contact each participant approximately 24 hours post-biopsy to inquire about potential adverse events and to remind them to contact study personnel if any adverse events occur within the subsequent 72 hours.

#### Oral glucose tolerance test (OGTT) [44].

After completion of the muscle biopsy, participants rest for 20 minutes, after which a baseline blood sample is collected. Participants then consume a solution containing 75 g of glucose (Trutol™, Thermo Scientific) within a 5- to 10-minute window. Subsequent blood samples are collected at 30 minutes, 60 minutes, and 120 minutes post-glucose beverage consumption. During this two-hour period, participants remain seated, abstain from food or fluid intake, and complete study questionnaires.

#### Questionnaires.

To monitor sleep quality, physical activity, alcohol consumption, and dietary intake throughout the study period, participants complete a series of questionnaires. These questionnaires assess sleep quality (Hooper Questionnaire [45]), physical activity levels (International Physical Activity Questionnaire [IPAQ] [36]), alcohol consumption (Timeline Follow-back [TLFB] [46]), and dietary intake (24-hour dietary recall using the ASA24 [47]). These questionnaires are administered in interview format at each major testing visit and weekly during laboratory visits.

### Day 2

#### Arrival procedures.

Participants return for testing Day 2 approximately 2–3 days following testing Day 1. Fasting is not required prior to testing Day 2, but participants are asked to finish their pre-visit meal at least 1 hour prior to their appointment time and log this pre-visit intake at T1 for replication prior to testing Day 2 at T2 (and T3, if applicable). On arrival, participants attest to abstaining from exercise, alcohol, and NSAIDs for 24 hours, and hydration status is assessed as on testing Day 1. Then, participants complete three physical function assessments (Timed Up and Go test, handgrip strength test, and 30-second sit-to-stand test), and subsequently, a cycle ergometer-based exercise capacity (VO_2peak_) test. These assessments are conducted at T1 and T2 for all groups, with an additional assessment at T3 for participants in the CON and LHT groups.

#### Timed up and go test [48].

Participants wear their regular/frequently worn footwear. They begin seated in a standard chair without arms (placed against a wall for safety purposes), with a clearly marked line placed 3 meters from the chair. On the researcher’s signal, participants stand, walk to the marked line at their usual walking pace, turn around, return to the chair at their usual pace, and sit down. Timing stops once participants are fully seated. After a 1-minute rest, participants complete a second trial.

#### Handgrip strength test [49].

Participants sit upright with elbows flexed at approximately 90 degrees and wrists in a neutral position. They squeeze a hand dynamometer (Jamar Hydraulic Hand Dynamometer, Performance Health Supply, Inc., USA) as forcefully as possible for 3–5 seconds. The dynamometer has a gauge that retains the highest grip value obtained until it is reset. Resetting occurs between attempts, after recording the grip strength value obtained for each completed attempt. This test is performed on each hand, alternating sides for a total of three attempts per hand with one minute of rest between attempts.

#### 30-second sit-to-stand test [50].

Participants sit in the middle of a 43.18 cm (i.e., 17”) high standard chair without arms (placed against a wall). They are instructed to rise to a fully standing position and sit back down as many times as possible within 30 seconds, keeping arms crossed over the chest, with the total number of complete stands recorded. Participants perform one trial.

#### Exercise capacity (VO_2_ peak) test [13].

Following the physical function assessments, participants complete a multi-stage exercise capacity test on a cycle ergometer to determine VO_2_ peak. Participants are connected to a calibrated metabolic cart (C09074-01–99, COSMED, USA), blood pressure (HEM-907XL, Omron, Kyoto, Japan), and heart rate monitor (1W, Polar H10, Bethpage, NY, USA). After the equipment is connected, participants begin by sitting quietly on the cycle ergometer for a 2-minute rest period, followed by a 5-minute warm-up at 15 W (women) or 30 W (men), maintaining a cadence between 60–80 rpm. Immediately after the warm-up, the test commences to Stage 1 with participants cycling at an initial workload of 30 W (women) or 50 W (men) for three minutes at a cadence between 60–80 rpm. Thereafter, the resistance increases at the end of each three-minute stage by 10 W (women) or 15 W (men) until the participant can no longer maintain the required cadence for two consecutive minutes or until volitional exhaustion is reached, with a goal of achieving a respiratory exchange ratio greater than 1.1. The metabolic cart records breath-by-breath VO_2_ (ml/kg/min) values. After test completion, 6-breath rolling VO_2_ averages are calculated and the peak 6-breath average value is retained as VO_2_ peak. Participants report their perceived exertion (rating of perceived exertion; RPE) at the end of each 3-minute stage and upon test completion using the Borg 6–20 scale [51]. Heart rate and blood pressure are continuously monitored during the test for participant safety and accurate prescription of exercise intensity for subsequent high-intensity interval training. After completing the test, participants perform a three-minute cooldown. Individual heart rate responses and corresponding VO_2_ values recorded during the exercise capacity test are used to establish appropriate exercise intensities for the HIIT intervention [13].

### Interventions

#### High intensity interval training.

Participants assigned to the HIIT group visit the laboratory for exercise 3 times per week for 12 weeks. On arrival to the laboratory on the first visit of each week, participants complete the Hooper questionnaire, IPAQ, TLFB, and ASA24; void their bladder; and then anthropometric measures (body mass, waist circumference, and hip circumference) are obtained. Then, participants don a heart rate monitor and begin with a 5-minute cycling warm-up at 50–60% of their VO_2_ peak heart rate (HR), maintaining a pedaling cadence between 60 and 80 rpm. Immediately following the warm-up, the HIIT protocol begins. This protocol consists of four high-intensity intervals, each lasting 4 minutes, with 3-minute active recovery periods between intervals [13]. To safely and appropriately progress the intensity for this population, the high-intensity intervals gradually increase from 70–75% of VO_2_ peak HR (determined by the exercise capacity test from T1) during weeks 1–2, to 80–85% by week 3, and 90–95% by week 4. After completing the final high-intensity interval, participants cool-down at a low intensity for 3 minutes. Heart rate is continuously monitored throughout the session and RPE is obtained at the end of each stage (warm-up, high-intensity intervals, active recovery intervals, and cool-down) to ensure participant safety and to adjust resistance as needed.

During Phase 2, participants assigned to the LHT and CON groups complete the 12-week HIIT intervention. The intervention is carried out in an identical manner as it was for the HIIT group, except the exercise capacity test from T2 (rather than T1) is used for exercise prescription.

#### Local heat therapy.

**Intramuscular (IM) Temperature Measurement:** To ensure that SKM temperature is consistently raised by 3–4 °C for the LHT group and maintained at a thermoneutral temperature for the CON group, participants undergo a preliminary procedure to establish the appropriate heat pad settings. This procedure takes place on the first day of the 12-week local heat therapy intervention. Specifically, with the participant in a supine position, the midpoint of the VL muscle on the contralateral leg from the biopsy is identified and marked, and the depth of the subcutaneous fat-SKM interface is determined using ultrasound. Then, the site is cleaned thoroughly with Chloraprep (BD ChloraPrep^TM^, Becton Dickinson, NJ, USA) to ensure sterility. Local anesthetic (1% lidocaine without epinephrine) is then injected subcutaneously and directly into the muscle beneath the temperature measurement site. An 18-G intravenous (IV) catheter is inserted perpendicularly into the vastus lateralis muscle at the designated site to a depth of approximately 1 cm deep to the fascia. Following insertion, the needle is removed, leaving only the flexible catheter tip in place. An implantable, sterile thermocouple probe (IT-18, Physitemp instruments, LLC. Clifton, NJ) is guided through the catheter into the targeted muscle layer to the catheter depth (approximately 1 cm into the VL muscle). After proper placement of the thermocouple, the catheter is carefully removed while stabilizing the thermocouple within the muscle. A second thermocouple is positioned on the thigh approximately 3 cm distal to the incision site to monitor thigh skin temperature. A third thermocouple is placed on the skin of the contralateral thigh, in the corresponding location, to compare skin temperature between legs. To ensure the thermocouples remain securely in place and do not shift during temperature measurement, both IM and skin thermocouples are firmly secured to the participant’s skin using Steri-strips (3M, MN, USA) and Tegaderm film (3M, MN, USA) [52]. After securing the thermocouples, skin temperature and IM temperature from VL are detected with a Type T thermocouple (TC-2000, Sable Systems Internation, North Las Vegas, NV). Analog output from the thermocouple will be connected to a data acquisition device (USB-6002, National Instruments Corp.) and then to a laptop. Data from the acquisition device is recorded at approximately 1 Hz with a custom MATLAB script using the Data Acquisition Toolbox. The script can be found at the following link https://osf.io/zngb9.

**Establishment of Heat Pad Temperature:** After securing the thermocouple, a commercially available heat pad (Theratherm, Chattanooga, TN, USA) is positioned to surround each of the thighs. To minimize the risk of heat-related skin discomfort or burns, a size-matched flannel cloth is inserted into the heat pad sleeve as a protective barrier. The heat pad is centered over the VL muscle, aligning with the thermocouple placement site. To ensure secure placement and consistent surface contact, two Velcro straps are applied around the upper and lower thigh to firmly secure the heat pad. After heat pad setup is complete, the bed is adjusted so that the participant achieves a seated position, and a cushion is placed beneath both knees to enhance comfort and prevent excessive heat buildup on the posterior portion of the thighs.

Based on extensive pilot testing, for both groups (LHT and CON), the heat pad is initially set to 50–55 °C for the first 20 min of heat pad temperature establishment sessions. If IM temperature fails to increase by approximately 1 °C within that period, the pad temperature is increased by 5 °C; otherwise, it remains at the same temperature. For the remainder of the session, we aim to achieve an approximate 2.5–3 °C rise in IM temperature by 60 minutes and a total increase of 3–4 °C by 90 min. If the IM temperature reaches a plateau or the rate of increase is insufficient to reach the IM temperature targets, the pad temperature is increased by 5 °C. To maintain participant blinding, they are informed that the heat pad will be set to maintain a target muscle temperature which could be *up to* maximum temperature. Throughout the 90-minute heating session, skin temperature on the thigh, IM temperature, and forehead skin temperature are monitored at 5-minute intervals. If skin temperature on the thigh surpasses 41.5°C, the heat pad is temporarily loosened to allow the skin temperature to decrease and excess sweat will be removed as needed throughout the session. Once skin temperature is below 41.5°C, the heat pad is reapplied. In addition to the skin temperature, if the participant reports thermal discomfort, the heat pad will be loosened, and the heat pad temperature lowered.

Based on the initial temperature establishment session, participants are told their heat pad settings for at-home sessions. For the LHT group, the heat pad temperature setting required to achieve the target IM temperature increase of 3–4°C is used. For the CON group, heat pad temperature settings will be 37°C- 40°C to ensure the muscle temperature remains at a thermoneutral level (approximately 36°C). Each participant uses their personal recorded settings for their at-home local heat therapy sessions. For subsequent IM temperature monitoring sessions (weeks 6 and 12 of Phase 1 for CON and LHT groups), the heat pad is set to the participant’s personalized settings and adjusted as needed if targets are not reached.

**Local Heat Therapy Sessions:** Participants assigned to the LHT and CON groups complete the local heat therapy intervention by applying heat pads 6 days per week for 12 weeks, with each session lasting 90 minutes, during Phase 1 of the study. Throughout this 12-week intervention, participants perform local heat therapy sessions 5 days per week at home and 1 day per week in the laboratory.

**Fig 3:**
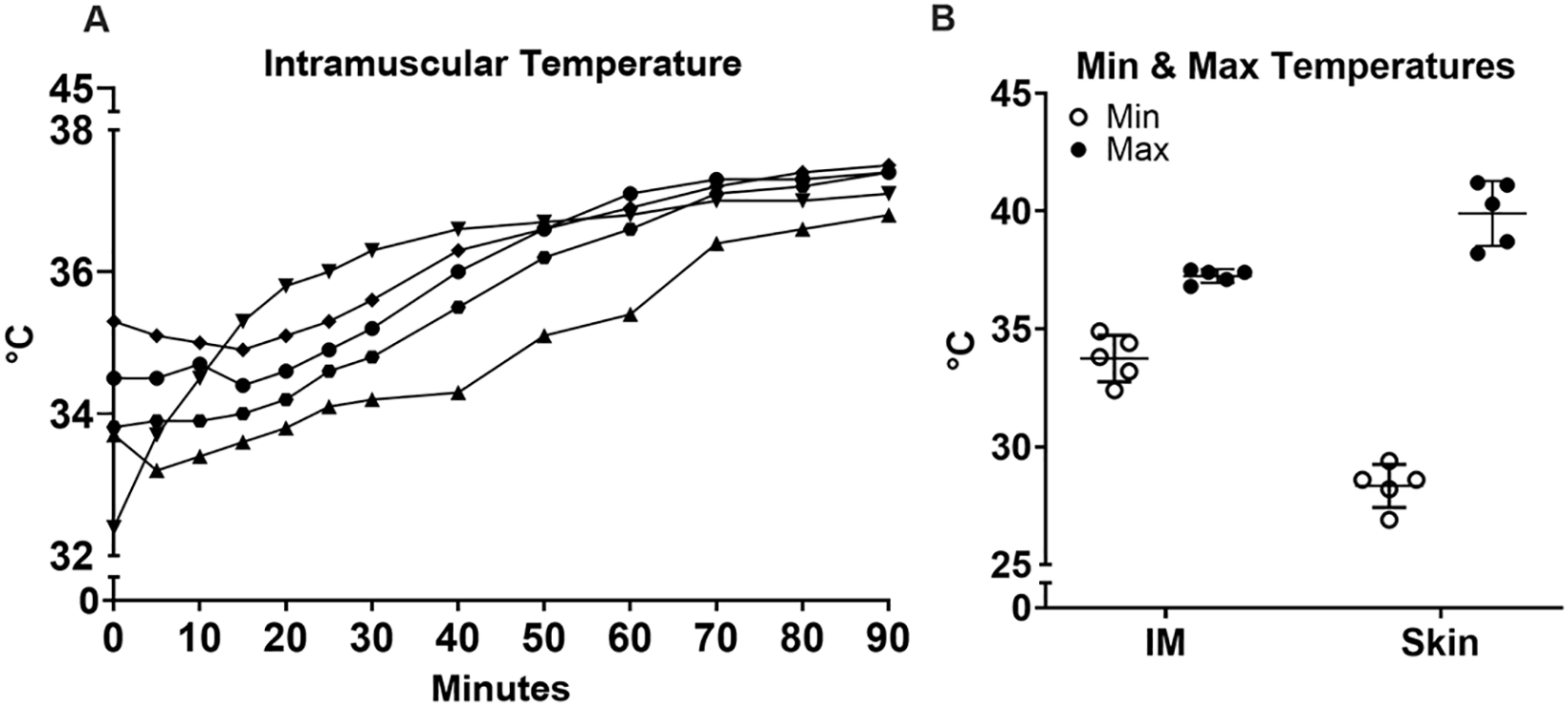
Intramuscular (IM) and skin temperature responses to a 90‑minute local heat therapy session. **(A)** IM temperature was continuously monitored using an implanted thermocouple. Each unique symbol represents data recorded from a different participant. **(B)** The minimum (min; open circles) and maximum (max; filled circles) IM and skin temperatures recorded during a 90-minute local heat therapy session.

**Feasibility, Tolerability, and Safety:** Preliminary data from participants who completed the local heat therapy protocol demonstrate that the intervention is both feasible and well tolerated. IM temperature and skin temperature are continuously monitored throughout the initial 90‑minute heating session and again after 6 and 12 weeks of the CON and LHT interventions to evaluate thermal load, tolerability, and skin safety. Data from the first 5 participants who completed the IM temperature measurement procedure demonstrate an average increase of 3.5 °C in IM temperature (Fig 3A**, 3B)**, and an average maximum skin temperature of 39.9 °C (Fig 3B). Collectively, these findings confirm that the heat therapy protocol reliably elevates IM temperature to the targeted temperature range without exceeding safe limits, supporting the feasibility of the intervention for use in this population.

### At-home local heat therapy sessions

Participants are provided with their two heat pads with flannel inserts, four Velcro straps (two per leg), and a log to record details of each at-home local heat therapy session. Participants are instructed to empty their bladder before starting each session to minimize interruptions. Then, they securely place heating pads around each thigh, ensuring a snug fit with Velcro straps, and set them continuously at the predetermined temperature for 90 minutes. Participants record the start and end times for each session, the number of times the heating pads are removed, duration of and reason for each removal, and any discomfort experienced. There are no specific restrictions regarding session location (e.g., home or office), time of day, or permitted activities during the session (e.g., watching TV, reading). Participants are advised to set a timer or alarm and to remain awake during each session. Lastly, the number of at-home local heat therapy sessions completed each week will be recorded to evaluate adherence and compliance with the protocol.

To ensure safety during at-home heat therapy and to facilitate participant acclimation over the 12-week intervention period. For all at-home heat therapy sessions, participants will document the temperature used, the duration of heat exposure, and any pertinent notes (e.g., discomfort) allowing individualized monitoring of thermal exposure and acclimation and ensuring safe progression. These records will be reviewed to monitor adherence, track thermal acclimation, and ensure that progression remains within safe limits.

### In-laboratory local heat therapy sessions

Immediately on arrival for each weekly in-laboratory heating session, participants return their logs from the prior week’s at-home heating sessions to confirm compliance. Then, they void their bladder and anthropometric measures (body mass, waist circumference, and hip circumference) are obtained. Following anthropometric measurements, heating pads are applied to both legs and set to the pre-determined temperature for 90 minutes. During the 90-minute heat pad application, research staff monitor participants’ comfort level and skin temperature for both legs every 5 minutes and measure blood pressure every 10 minutes. During the 90-minute session, participants complete the Hooper questionnaire, IPAQ, TLFB, and ASA-24. At weeks 6 and 12 of Phase 1, the IM temperature measurement is repeated during the in-laboratory heat therapy session.

### Study monitoring

#### Evaluation of adverse events.

Local heat therapy may cause mild thermal discomfort, erythema, or, rarely, localized blistering following heat exposure, especially if used improperly or for prolonged periods. However, the risk of significant injury is low, as the heat pad applied to the thigh is unlikely to raise core body temperature. Skin and body temperature are monitored during lab sessions, and therapy will be stopped if body temperature exceeds 40°C. To ensure safety, the lowest effective heat setting is used, and participants are asked to set a timer to limit sessions to 90 minutes. Minor skin redness may occur but typically resolves within 12 hours. Any skin discomfort experienced during the heat pad sessions is documented during both in-laboratory and at-home use, and any discomfort reported during in-laboratory sessions is promptly addressed and used to inform adjustments for subsequent at-home sessions. Participants receive contact information for reporting any issues. Weekly in-lab sessions are supervised by the study team to ensure safety throughout the intervention.

In the event of mild adverse thermal reactions (e.g., erythema or blistering), participants will be instructed to apply aloe vera or fragrance-free moisturizer to the affected area. If the blister opens spontaneously, participants will be advised to apply a thin layer of antibiotic ointment to protect the exposed skin. Both in-laboratory and at-home heat therapy will be temporarily suspended for the affected limb. Adverse reactions will be monitored by the clinical research coordinator through follow-up communication and photographic documentation until complete resolution. Communications and photographs will be relayed to our safety officer (SO) and reported to appropriate parties in accordance with our Data and Safety Management Plan. The study team will follow any additional guidance provided by the SO.

#### Criteria for discontinuing.

Participants may be withdrawn from the study by the research team if necessary. Specifically, this may occur if a participant initiates a significant lifestyle change (e.g., starting a new exercise regimen, dietary program, or supplement routine) or develop a new health condition that may increase their risk. Additionally, participants who do not adhere to at least 80% of the study requirements may be subject to early discontinuation.

#### Retention.

In addition to compensation, participant retention and complete follow-up are supported through multiple strategies. The anticipated time commitment is clearly communicated during screening, and participant availability is confirmed prior to enrollment. Flexible scheduling options (e.g., evenings, weekends, and rescheduling windows) are employed, along with structured engagement and retention strategies, including regular reminders, check-ins, and timely follow-up after missed visits. Adherence and patterns of missing data are monitored throughout the study to allow early identification of barriers and implementation of corrective measures (e.g., adjustment of visit timing or tailored reminders). Lastly, outcome measures, such as body composition, HbA1c, functional performance, and exercise capacity, etc., are provided at each major assessment point. An accompanying information sheet outlines the importance of these non-diagnostic results in relation to overall health (e.g., glucose regulation, body composition, fall risk). Study staff also review and explain the results to participants to ensure understanding. Collectively, these approaches are intended to optimize completion rates while preserving protocol integrity.

### Assessment of outcomes

#### Assessment of glycemic control.

Blood samples collected during the OGTT (before and 30, 60, and 120 minutes after consuming 75 g glucose) at T1, T2, and T3 will be analyzed for glucose, insulin, and C-peptide concentrations to assess glycemic control. Glucose concentrations will be measured using a GL5 Analyzer (Analox Instruments, UK) and insulin and C-peptide levels will be quantified using commercially available ELISA kits. Resulting values will be used to calculate fasting glucose (pre-OGTT) [53], glucose tolerance (1- hour and 2-hour glucose and glucose area under the curve [AUC] during OGTT) [53–55], insulin sensitivity (fasting insulin [56], insulin AUC [53], and the Quantitative Insulin Sensitivity Check Index [QISCI] [57]), insulin resistance (homeostatic model assessment of insulin resistance [HOMA-IR]), and beta-cell function (homeostatic model assessment of beta-cell function [HOMA-β] [58], C-Peptide Index [59], and Insulinogenic Index [59]).

#### Assessment of frailty risk.

Frailty risk will be assessed at T1, T2, and T3 using the Frailty Phenotype Score [1]. Frailty risk (robust, pre-frail, frail) will be determined based on gait speed (timed up and go [60]), SKM strength (handgrip dynamometry [1], complemented by 30-s sit-to-stand test [61]), unintentional weight loss (self-report, ≥ 10 lbs or ≥5% of body mass in past year [1] or between major study timepoints, assessed during questionnaires), fatigue (SF-36 energy subscale [60]), and physical activity (IPAQ [36]). These measures will be complemented by exercise capacity (VO_2peak_) testing and body composition (DEXA, anthropometric measures) since fat-free body mass is associated with lower frailty risk [49].

#### Assessment of muscle architecture.

**Muscle fiber CSA and capillarization:** Immunohistochemistry (IHC) will be used to analyze type I and type II CSA and capillary density. Muscle samples in Z-fix will be paraffin embedded, sectioned at ~5 um using a microtome, and two sections from the same sample mounted on each slide for analysis in duplicate. Samples will be prepared and immunofluorescence (IF) or immunohistochemistry (IHC) performed using previously published methods [62–64]. Briefly, after deparaffinization and epitope retrieval, sections will be blocked, and primary antibodies added followed by appropriate fluorophore-conjugated secondary antibodies. Slides will be mounted using media containing DAPI (Vectashield, Vector Laboratories, Newark, CA, USA) and imaged using widefield fluorescence (IF) or brightfield (IHC) microscopy (Cytation C10, Agilent Technologies, Santa Clara, CA, USA). Stains will include type I and type II fiber stains to assess fiber type, laminin to delineate muscle fiber borders and accurately assess CSA, and CD31 (platelet endothelial cell adhesion molecule-1; PECAM-1) to assess capillarization. CFPE will be calculated based on the proportion of muscle fibers bordered by capillaries. Images will be analyzed using ImageJ.

**Mitochondrial morphology and functional indices:** Transmission electron microscopy (TEM) will be used to analyze mitochondrial content and morphology in glutaraldehyde-paraformaldehyde-fixed muscle samples using previously published methods [65] with slight modification. Briefly, fixed samples will be rinsed, cut into small (~1 × 2 mm) segments, and post-fixed in 1% OsO_4_. Samples will then be dehydrated, embedded in epoxy resin, and sliced into ultrathin sections (~60 nm) before imaging on a Hitachi H-8100 Scanning/Transmission Electron Microscope (STEM).

As an additional index of mitochondrial content, mitochondrial DNA (mtDNA; *DLOOP*:*B2M*) will be measured using qPCR [66]. DNA will be extracted from a small portion (~10 mg) of flash frozen SKM using a commercially available DNA extraction kit (DNeasy, Qiagen) and quantified (NanoDrop, ThermoFisher Scientific, Waltham, MA, USA). Previously validated [67] custom primers designed to span exon-exon junctions will be purchased (Integrated DNA Technologies, Coralville, IA, USA). Final reactions will contain gDNA (25 ng), primers (500 mM), SyBr green (Qiagen, Venlo, Netherlands), and nuclease-free water to 20 ul. All samples from a single participant will be analyzed on the same PCR plate and reactions will be run in triplicate.

Succinate dehydrogenase (SDH) activity will be measured using histochemistry in OCT-fixed samples according to previously published methods [67]. Briefly, samples will be cross-sectioned and incubated with phosphate buffer containing sodium succinate and nitro blue tetrazolium (NBT), washed with acetone and distilled water, and mounted. Images will be acquired using brightfield microscopy (Cytation C10) and analyzed using ImageJ and custom Python code that we will make available on GitHub.

Western blotting [39,40,68] will be used to detect the concentration of electron chain complexes in flash frozen SKM samples. Proteins will be separated using SDS-PAGE, electrophoretically transferred to a low-fluorescence PVDF membrane, and total protein imaged using a ChemiDoc MP (Bio-Rad Laboratories, Hercules, CA, USA). The membrane will be blocked and incubated overnight with commercially available primary antibodies against selected proteins in each electron transport chain complex (Total OXPHOS Human Antibody Cocktail, Abcam, UK) among other mitochondrial proteins. To provide insight into potential mechanism, primary antibodies against p-TRPV1 and TRPV1 will also be applied. Membranes will then be rinsed, incubated with the appropriate secondary antibodies, and imaged using enhanced chemiluminescence on a ChemiDoc MP (Bio-Rad Laboratories). Band density will be determined using Bio-Rad image analysis software and normalized to β actin.

Citrate synthase activity [69] will be measured in lysed flash frozen skeletal muscle samples in duplicate using a commercially available enzymatic assay (CS0720, Sigma-Aldrich, Burlington, MA, USA) and spectrophotometry.

### Data management

This clinical trial will generate a diverse set of scientific data from human participants. As described, these data will include demographic (e.g., age, sex, race, ethnicity) and anthropometric (e.g., body mass, body composition, height) information, clinical data (e.g., medical history, oral glucose tolerance test parameters, and responses to questionnaires), functional outcomes (e.g., exercise capacity, grip and leg strength, gait speed), and molecular measurements (e.g., muscle fiber cross-sectional area, capillarization, mitochondrial measures). These data will be collected from 54 older adults at risk for type 2 diabetes at multiple time points. Data collected at study visits will be entered into the two-way encrypted, HIPAA-compliant REDCap2 database and linked only by unique study ID codes, with personal identifiers stored separately in REDCap2. Biospecimens will be stored in a deidentified manner. Raw data generated from analysis of deidentified biospecimens will be stored on secure, cloud-based servers at Texas Tech University before uploading analysis-ready data into REDCap2.

Deidentified data from REDCap2 will be uploaded to the NIA Aging Research Biobank, a controlled-access data repository, where new unique IDs will be assigned, providing an additional layer of data protection. These data will be made available by request (submitted through the repository) on release of associated preprints or publication of associated manuscripts, and will remain accessible as long as the hosting repositories are supported. Metadata (e.g., protocols, data dictionary, etc.) will accompany each dataset. All custom code will be shared via GitHub with detailed documentation in a README file.

To ensure data quality, assessors are trained on all operating procedures. Standardized procedures and instruments with known reliability and validity are used where available. Duplicate measurements, range checks, and data validation (e.g., double entry where appropriate) are employed.

Anthropometric outcomes will be monitor weekly to ensure participants are not deviating from their normal routine and diet outside of the study. In addition, interim analyses for major measurements will be performed every 6 months from the beginning of the second year onwards.

### Statistical analyses

All outcome measures at T1, T2, and T3 will be summarized using descriptive statistics. The study evaluates treatment efficacy by analyzing changes across three distinct time points (T1, T2, and T3). While the data are longitudinal, the primary aims focus on the magnitude of change within specific intervals. Therefore, the magnitude of change between time points for each outcome measure will be calculated for analyses.

To achieve Aim 1 (comparing LHT with CON and LHT with HIIT; k = 3), changes between T1 and T2 values (i.e., before and after Phase 1) for all muscle architecture outcomes, glycemic control outcomes, and frailty risk indicators will be calculated and checked for the assumption of normality using the Shapiro-Wilk test. An appropriate transformation will be conducted if normality is violated. Then, one-way ANOVA with three groups (CON, HIIT, LHT) will be run. This will be followed by two non-orthogonal planned contrasts (CON vs. LHT, LHT vs. HIIT). The Bonferroni correction will be used to control the familywise error rate.

To achieve Aim 2 (comparing LHT with CON after participants in both groups undergo HIIT during Phase 2 of the study; k = 2), changes between T2 and T3 values for all muscle architecture outcomes, glucose tolerance outcomes, and frailty risk indicators will be calculated and checked for the assumption of normality using the Shapiro-Wilk test. An appropriate transformation will be conducted if normality is violated. Then, a *t* test will be run to compare CON and LHT, and confidence intervals will be constructed to compare each variable between the two treatment groups. If improvements for participants in the LHT group from T1 to T2 make the absolute change from T2 to T3 smaller, we will also compare the combined improvements across both phases for the 2 treatment groups. Changes between T1 and T3 in muscle architecture outcomes, glucose tolerance outcomes, and frailty risk indicators will be calculated and transformed if needed. ANOVA analyses will be run and confidence intervals constructed to compare the combined improvements of each factor between the two treatment groups (CON and LHT). We can also employ a one-way ANCOVA for the T2-to-T3 analysis, including the T1-to-T2 change score as a covariate. This ANCOVA method can clarify the relationship between early-phase response and late-phase progress.

Because the study utilizes balanced permuted block randomization, we expect key baseline characteristics, such as sex assigned at birth, age, and glucose tolerance, to be well-balanced across the three treatment groups. Our analysis will begin with a one-way ANOVA to evaluate primary treatment effects. To ensure the model is both parsimonious and robust, we will perform residual diagnostics by plotting residuals against various participant characteristics (demographic, clinical, and molecular). Non-random residual distributions will signal the need to include missing main effects, while diverging patterns across treatment levels will indicate potential interaction effects. These additional terms will be formally tested for significance before being integrated into the final model.

Note that integrating covariates into the model generally enhances the power to detect treatment effects by reducing error variance. Conversely, the power to detect interaction effects is usually low, as these effects typically exhibit smaller magnitudes and larger standard errors. Given that our sample size is determined based on a one-way ANOVA, all interaction analyses will be treated as exploratory.

While we anticipate complete data sets for primary outcomes assessed at T1, T2, and T3, there is a possibility of missing data. To address potential bias and maintain statistical power in the presence of potential missing data, we will employ Multiple Imputation by Chained Equations. We will generate several imputed datasets using all available baseline characteristics and previous timepoint values. Each dataset will be analyzed independently using the specified ANOVA or ANCOVA model, and the resulting estimates and standard errors will be pooled to produce a single inference according to Rubin’s Rules. This approach accounts for both within-imputation and between-imputation uncertainty.

To assess the robustness of our primary findings, a sensitivity analysis will be performed. While the primary analysis assumes data are Missing At Random, we will explore the impact of Missing Not At Random data. Specifically, we will apply a penalty to the imputed values of the treatment group to simulate a scenario where participants who dropped out had poorer outcomes than those who remained. If the treatment effect remains statistically significant under these conservative assumptions, the primary results will be considered robust.

## Discussion

Frailty and type 2 diabetes mellitus (T2D) are increasingly burdensome health issues for older adults. While exercise training is a cornerstone for managing metabolic diseases, adherence to exercise can be challenging in this population [9,16,17] due to compromised oxygen delivery and mitochondrial dysfunction [19]. Similar to whole-body heat therapy, short-term local heat application has been shown to promote mitochondrial health and preserve or improve capillary density, muscle size, and strength in younger adults [26,27,29,70]. Therefore, the overarching goal of this clinical trial is to examine how local heat therapy improves muscle architecture, glycemic control, and subsequent exercise adaptations, thereby decreasing risk for frailty and metabolic disease progression in older adults with prediabetes.

SKM metabolic function is dysregulated in T2D and is associated with alterations in muscle architecture, including mitochondrial dysfunction [71–73], reduced capillarization [5,6], and decreased SKM mass [7,8]. Notably, mitochondrial dysfunction also appears to underlie decreased capillarization in adults with cardiometabolic disease [74], potentially compromising the capillary network including in SKM. Greater muscle capillary density provides a larger muscle-to-blood exchange area for oxygen diffusion into SKM to support mitochondrial function [75]. Moreover, the SKM capillary network accounts for about 70–90% of insulin-mediated glucose disposal [76,77]. Improved capillarization as an adaptation to exercise training contributes to improved exercise capacity [78]. In contrast, decreased exercise capacity in individuals with T2D can be partially explained by capillary rarefaction [79] and thus reduced peripheral oxygen extraction [80]. These impairments not only contribute to glycemic dysregulation in T2D but also play a role in the loss of SKM mass by limiting nutrient and oxygen delivery necessary to maintain metabolic activity (CITE). Ultimately, this decline in muscle mass and quality leads to reduced function and contributes to the development of phenotypic frailty (CITE). In older adults at risk for T2D, mitochondrial dysfunction and impaired oxygen delivery may contribute to exercise intolerance, which can lead to decreased exercise participation and adherence [19], further elevating the risk of developing T2D and related complications.

Studies in both humans and rodent models suggest that whole-body heat therapy enhances mitochondrial function, muscle capillarization, glycemic control, and muscle fiber CSA, even in the absence of structured exercise training. With whole-body heat therapy, fasting glucose and insulin decreased in sedentary, overweight men (34–39°C for 1–6 hours, 10 sessions) and obese rats (38°C for 30 min, 3 times per week for 12 weeks) [22–25]. In contrast, 3 or 6 weeks of whole-body heat did not correct the glucose dysregulation in a Type I diabetic (T1D) model [81]. Al Sabagh et al. speculated that the specific models used (obese, at risk for T2D vs T1D) may explain the discordant findings [81]. Still, 6 weeks of whole body heat therapy was able to alleviate SKM mass loss and attenuate functional decreases in the T1D model [81].

While whole-body heat therapy modalities such as sauna and hot water immersion show promise, they may not be practical for widespread use. Studies using LHT (e.g., shortwave diathermy, hot water circulation) demonstrate similar benefits to those observed with whole-body heat therapy [70,82]. Applying shortwave diathermy 2 hours per day for 6–10 days to raise the IM temperature by approximately 3–4°C increased mitochondrial biogenesis and attenuated muscle atrophy in young sedentary men and women [26,70]. Heating the thighs alone using hot (48–52°C) water to raise leg skin temperature to 39–40°C without altering core temperature increased vascular endothelial growth factor (*VEGF*) and angiopoietin 1 mRNA expression [29], consistent with improved angiogenic capacity. However, mitochondrial density did not change with these heat therapy settings [21]. Other work using a single 4-hour local heat application that only raised the IM temperature to ~36.3°C (average skin temperature of 37.3°C) also reported no changes in mitochondrial gene expression [82], suggesting that there may be a higher threshold for heat-induced SKM mitochondrial adaptations. These results suggest that by understanding how heat elicits specific physiological adaptations and refining LHT settings accordingly, LHT can be an alternative therapeutic approach. However, shortwave diathermy and hot water circulation apparatuses remain costly, making these modalities of heat delivery less accessible for widespread use. Therefore, the clinical trial described herein will test the efficacy of LHT using affordable heat pads as a standalone intervention to improve SKM architecture, glycemic control, and decrease frailty risk, and as a means of preconditioning SKM to improve exercise adaptations among older adults with prediabetes.

## Conclusions

The HEAT Study will be the first to determine the impact of using affordable local heat pad therapy on SKM architecture, glycemic control, and frailty risk and to explore how these changes may enhance skeletal muscle adaptations to exercise training in older adults with prediabetes. Findings from this study will expand our understanding of physiological effects of local heat therapy, and biospecimen analyses will provide evidence for mechanistic insights. These data will inform parallel mechanistic studies to undercover therapeutic targets. Most importantly, findings will provide foundational evidence to include local heat pad therapy in clinical recommendations to combat metabolic disease progression and frailty risk in older adults, potentially transforming clinical practice, and ultimately reducing the health burden of T2D and frailty.

## Supporting information

S1 Table SPIRIT checklist. (DOCX)

S1 File Detailed plan. (PDF)
