# Supplementary material for: Effects of local heat on metabolic health, frailty risk, and exercise adaptations in pre-diabetic older adults: Protocol for the Heat and Exercise in Aging as Therapy (HEAT) clinical trial
Source: PLoS One. 2026 Jun 15;21(6):e0351577. doi: 10.1371/journal.pone.0351577 (PMC13268183; doi:10.1371/journal.pone.0351577)
Supplement: S1 File — (PDF) [file pone.0351577.s002.pdf]

Initial Submission

1: Human Subject Research

Answer the following questions to determine if you need IRB review and approval.

1-1 Research

*required

Research is defined by 45 CFR 46.102(l) as:

A systematic investigation, including research development, testing and evaluation, designed to develop or contribute to generalizable knowledge.

Does your proposed study meet the definition of research?

✓ Yes

No

1-2 Human Subjects

*required

Human Subject is defined by 45 CFR 46.102(e) as:

A living individual about whom an investigator (whether professional or student) conducting research

- Obtains or receives private identifiable information or identifiable biospecimens about the individual(s)
- Obtains information or biospecimens through intervention or interaction with the individual(s)

Does your proposed study involve and meet the definition of human subjects?

✓ Yes

No

If you answered YES to all questions in Section 1 complete the rest of the sections.

2: Review Type

Complete the following section.

*required

2-1 Review Type

Select the appropriate review type.

Exempt

✓ Expedited

Research activities defined by the list of expedited categories and the project involves no more than minimal risk.

Minimal risk means that the probability and magnitude of harm or discomfort anticipated in the research are not greater in and of themselves than those ordinarily encountered in daily life or during the performance of routine physical or psychological examinations or tests.

Full Board

Relying on IRB approval from another institution (IAA)

2-2 External Collaboration

Only select collaboration if there are Non Texas Tech researchers, employees, and/or students working on this research.

Collaboration

International Research

2-3

Research will be conducted outside of the United States of America and/or data will be shared across borders.

International Research

3: Expedited Review

Select the category that best describes your research study.

*required

Claim for Expedited Review

Federal regulations and University policy require that research must pose no more than minimal risk and that one or more of the following categories must be checked in order for research to receive expedited review.

Minimal risk means that the probability and magnitude of harm or discomfort anticipated in the research are not greater in and of themselves than those ordinarily encountered in daily life or during the performance of routine physical or psychological examinations or tests.

Category 1a: Drugs for which an Investigational New Drug Application (21 CFR Part 312) is NOT Required

Category 1b: Medical Devices

✓ **Category 2a: Collection of Blood Samples for Nonpregnant Adults who Weigh At Least 110 pounds**

The amounts drawn may not exceed 550 ml in an 8 week period and collection may not occur more frequently than 2 times per week.

*required

2a(1) **Data collection to be used.**

Check all that apply.

- ✓ Finger stick
- Heel stick
- Ear stick
- ✓ Venipuncture

✓ Category 2b: Collection of Blood Samples from Other Adults and Children

Consider the age, weight, and health of the subjects, the collection procedure, the amount of blood to be collected, and the frequency with which it will be collected.

The amount drawn may not exceed the lesser of 50 ml or 3 ml per kg in an 8 week period and collection may not occur more frequently than 2 times per week.

*required

2b(1) **Data collection to be used.**

Check all that apply.

✓ Finger stick

Heel stick

Ear stick

✓ Venipuncture

Category 3: Prospective Collection of Biological Specimens for Research Purposes by Noninvasive Means

✓ **Category 4: Collection of Data Through Noninvasive Procedures Routinely Employed in Clinical Practice**

The following procedures are excluded from expedited review:

- General anesthesia or sedation
- X-rays
- Microwaves

- Where medical devices are employed, they must be cleared/approved for marketing. (Studies intended to evaluate the safety and effectiveness of the medical device are not generally eligible for expedited review, including studies of cleared medical devices for new indications.)

Examples include:

- Physical sensors that are applied either to the surface of the body or at a distance and do not involve input of significant amounts of energy into the subject or an invasion of the subject's privacy
- Weighing or testing sensory acuity
- Magnetic resonance imaging
- Electrocardiography, electroencephalography, thermography, detection of naturally occurring radioactivity, electroretinography, ultrasound, diagnostic infrared imaging, doppler blood flow, and echocardiography
- Moderate exercise, muscular strength testing, body composition assessment, and flexibility testing where appropriate given the age, weight, and health of the individual

---

Category 5: Materials Collected, or Will Be Collected Solely for Nonresearch Purposes

---

✓ **Category 6: Collection of Data from Voice, Video, Digital, or Image Recordings Made for Research Purposes**

---

*required

Methods to be used.

6a

Check all that apply.

- [ ] Data from voice or audio recordings made for research purposes
- [ ] Data from video recordings made for research purposes
- [ ] Data from digital recordings made for research purposes
    - Audio signals are transformed into a series of pulses that correspond to patterns of binary digits
- [x] ✓ Data from image recordings made for research purposes

Category 7: Individual or Group Characteristics or Behavior

Complete the following section. List all researchers involved with recruitment, data collection, and analysis.

NOTICE: HUMAN SUBJECT TRAINING REQUIREMENT

All research study personnel who engage with participants will need to complete human subject training prior to IRB approval and prior to conducting any human research-related activities. The new training requirement will be applied when an existing study is due for renewal, requires modification(s), or is chosen for a post approval monitoring.

Human research-related activities include recruitment of participants, consenting of participants, data collection (anonymous or identifiable), gaining access to identifiable data, and/or conducting analysis of identifiable data. These training requirements will apply to all Principal Investigators, Co-Investigators, and research staff/students. This includes both individuals within the TTU system and external collaborators.

This requirement will go into effect January 1, 2023. For more information visit our webpage.

Effective January 1, 2023, TTU research personnel must complete one of the following trainings every 3 years:

- CITI - TTU Human Subject Research - Required Basic
- TTU Human Subject Training
- External – CITI Human Subject Training

4-1 **Principal Investigator**

TTU OP 74.09

*required

4-1a **Check the appropriate box.**

✓ TTU full-time or tenured faculty member

*required

4-1a(1) **College**

College of Arts and Sciences

*required

4-1a(2) **Department**

Kinesiology & Sport Management

TTU full-time employee with a terminal degree in their discipline

*required

Find and add PI to the submission.

4-1b Name: Hui Ying Luk
Organization: Kinesiology and Sport Mgmt
Address: 2500 Broadway MS3011, Lubbock, TX 79409
Phone: 8068340827
Email: huiying.luk@ttu.edu

4-1a(3)a Attach human subject training.

HuiYing CITY (2).pdf

Primary Contact

4-2

Primary contacts need to be included in section 4.3 as a co-investigator if they are not the principal investigator. The primary contact and principal investigator will receive all notifications and letters.

*required

4-2a **Find and add primary contact to the submission.**

Name: Danielle Levitt
Organization: Kinesiology and Sport Mgmt
Address: 2500 Broadway MS3011, Lubbock, TX 79409
Phone: 8068341830
Email: danielle.levitt@ttu.edu

Name: Hui Ying Luk
Organization: Kinesiology and Sport Mgmt
Address: 2500 Broadway MS3011, Lubbock, TX 79409
Phone: 8068340827
Email: huiying.luk@ttu.edu

4-3 **Co-Investigators**

Include investigators assisting with instrument development, recruitment, data collection, and data analysis.

*required

4-3a **Select all investigators involved with this research project.**

None

✓ List TTU Faculty or Staff Co-Investigator(s):

*required

4-3a(1) *Multiple names are allowed.*

Name: Fangyuan Zhang
Organization: Mathematics and Statistics
Address: 2500 Broadway MS1042, Lubbock, TX 79409
Phone: 8068342587
Email: fangyuan.zhang@ttu.edu

Name: Heather Vellers
Organization: Kinesiology and Sport Mgmt
Address: 2500 Broadway MS43011, Lubbock, TX 79409-3011
Phone: 8068348554
Email: heather.l.vellers@ttu.edu

Name: Danielle Levitt
Organization: Kinesiology and Sport Mgmt
Address: 2500 Broadway MS3011, Lubbock, TX 79409
Phone: 8068341830
Email: danielle.levitt@ttu.edu

4-3a(1)(a) Attach human subject training.

✓ List TTU Graduate or Undergraduate Co-Investigator(s):

4-3a(2) *Multiple names and selections are allowed.*

✓ Graduate Student(s)

*required

Name: Casey Appell
Organization: Kinesiology and Sport Mgmt
Address: , Lubbock, TX 79409-1035
Phone:
4-3a(2)a Email: Casey.Appell@ttu.edu

Name: Alexandra Khartabil
Organization: Kinesiology and Sport Mgmt
Address: , Lubbock, TX 79409-1035
Phone:
Email: akhartab@ttu.edu

4-3a(2)a1 Attach human subject training.

*required

4-3a(2)b **The purpose of this study:**

Doctoral Dissertation

Master's Thesis

Research conducted as a class assignment with the intention of publishing results

Class assignment requiring IRB submission with no intention of publishing results

✓ Students are members of the research team

Other

✓ Undergraduate Student(s)

*required

4-3a(2)c

Name: Rylea Helmberger
Organization: Kinesiology and Sport Mgmt
Address: , Lubbock, TX 79409-1035
Phone:
Email: Rhelmber@ttu.edu

Name: Ashley Williams
Organization: Kinesiology and Sport Mgmt
Address: , Lubbock, TX 79409-1035
Phone:
Email: wil98200@ttu.edu

Name: Matthew DiLeo
Organization: Kinesiology and Sport Mgmt
Address: , Lubbock, TX 79409-1035
Phone:
Email: madileo@ttu.edu

Name: Connor Mills
Organization: Kinesiology and Sport Mgmt
Address: , Lubbock, TX 79409-1035
Phone:
Email: mil67126@ttu.edu

Name: Ernan Carrasco
Organization: Kinesiology and Sport Mgmt
Address: , Lubbock, TX 79409-1035
Phone:
Email: erncarra@ttu.edu

4-3a(2)c1 Attach human subject training.

*required

4-3a(2)d **The purpose of this study:**

Undergraduate Research

✓ Students are members of the research team

Research conducted as a class assignment with the intention of publishing results

Class assignment requiring IRB submission with no intention of publishing results

Other

✓ External Researchers: Co-investigators outside of Texas Tech University

*required

4-3a(3) **List non-TTU investigator(s) engaged in this research. Provide their contact information, title, and place of work.**

Robyn Richmond, MD
Assistant Professor of Surgery
Associate Program Director, General Surgery Residency
Trauma, Surgical Critical Care, Acute Care Surgery
Department of Surgery
Texas Tech University Health Sciences Center
robyn.richmond@ttuhsc.edu
972 987 9616

*required

4-3a(3)a **Check all that apply.**

Developing instrument

Recruiting participants

Interaction with participants

Working with de-identified data

Working with identifiable data

Assisting with writing manuscript

✓ Other

*required

4-3a(3)a1 **Include any information that helps to explain their role with the research team.**

Dr. Richmond will provide medical-related oversight/consultation on the muscle biopsy procedures. There will be no direct interaction between Dr. Richmond and the participants.

Attach human subject training.

4-3a(3)b

5: Funding

Complete the following section.

5-1 **Funding**

Funding is used to supplement the cost of the research and/or participant payment.

*required

5-1a **Select the type of funding to be used.**

✓ External (Office of Research Services - ORS)

*required

5-1a(1) **Cayuse SP - Sponsored Project Number (Example: 16-0123)**

23-0477

*required

5-1a(2) **Sponsored Project Title**

Glycemic control and frailty risk in older people at risk for type 2 diabetes: Impact of local heat therapy

*required

Find and add the Sponsor's name.

5-1a(3) **Name**
-

- Name - A to Z
- Name - Z to A

NIH - National Institute on Aging

*required

Attach a copy of the sponsored project that was submitted to the funding agency.

5-1a(4)

For example: scope of work, abstract, research description.

FINAL_ESI R01_HYLDEL_Specific aims.docx

FINAL_ESI R01_HYLDEL_Research Strategy.docx

Internal (TTU Funding)

Personal Monies

No Funding

Future Funding

5-2

If funding is secured, a modification will be required.

Enter the potential sponsor(s) name below.

5-2a

NIH-National Institute on Aging (1R01AG084597 [mPI: Luk/Levitt]; Just In Time information has been requested)

6: Rationale

Please respond to the following statements to help our IRB reviewers understand your research project.

6-1 Research Objective

*required

Briefly summarize the relevant background information.

Frailty-related medical expenses cost approximately $18 billion annually in the USA. Progressive losses in skeletal muscle (SKM) mass and function, often observed with aging and type 2 diabetes (T2D), contribute to phenotypic frailty characterized by slow gait speed, weakness, weight loss, fatigue, and low physical activity. T2D increases frailty risk by nearly 50% and risk for both conditions increases with age; therefore, preventing progression to T2D in older adults with prediabetes is critical. Additionally, SKM is the largest glucose disposal site in the body and insulin responsiveness, a component of glycemic control, is essential to maintain functional SKM mass. Thus, impaired glycemic control, a pathophysiological change underlying the development of T2D and observed in prediabetes, increases frailty risk. Conversely, improving capillarization and mitochondrial function support increased SKM mass and glycemic control, thereby decreasing T2D and frailty risk. Together, these SKM architectural variables (e.g., cross-sectional area [CSA], capillarization, mitochondria) are attractive targets for interventions such as exercise in prediabetic older people. High-intensity interval training (HIIT) has been used effectively in older adults with SKM benefits similar to those from aerobic and resistance training, and is more time-efficient. However, prediabetic older people may be exercise-intolerant or -resistant, underscoring the need for alternative therapies in place of (or in addition to) exercise. Emerging evidence supports repeated heat therapy as an alternative method to improve glycemic control and SKM architecture, and such adaptations may also improve muscle growth responses to subsequent exercise. While whole-body heat therapy is widely studied, local heat therapy (e.g., heat pad) is more practical and likely provides similar health benefits. Whether local heat therapy would have similar benefits to HIIT or whether heat pre-conditioning would improve adaptations to subsequent HIIT in prediabetic older adults is unknown.

6-2 **Importance of Research**

---

*required

Explain the scientific importance of the knowledge to be obtained as a result of this research.

Exercise is a first-line intervention for improving glycemic control and decreasing frailty risk due largely to adaptations in SKM. While exercise has a host of whole-body benefits, older individuals in general and those at risk for T2D (i.e., prediabetic) in particular may be exercise-resistant or -intolerant. Therefore, there is an urgent need for **efficacious and practical interventions** to improve muscle health in aging people to decrease their risk of developing T2D and frailty. The proposed project is innovative because we will:

- Use local heat therapy as an *easily implemented intervention* to improve the fundamental aspects of muscle health in aging prediabetic people, a novel application of this widely-used therapy
- Compare the efficacy of our proposed intervention alone and as a means of *preconditioning muscle* for improved exercise adaptations

Finally, this project will provide essential fundamental evidence to lay the groundwork for a subsequent clinical trial that examines the use of this technique in aging people with other SKM-related comorbidities. This work has the potential to **shift clinical practice** by providing direct evidence that supports a low-cost, practical intervention to improve metabolic health in at-risk individuals.

6-3 **Research Questions/Specific Aims**

---

*required

List the research question(s)/specific aims to be addressed.

The overarching hypothesis of this study is that local heat therapy improves muscle architecture, glycemic control, and subsequent exercise adaptations, and decreases frailty risk in prediabetic older adults, with TRPV1 as an underlying mechanism [mechanistic experiments will be in Aim 3, not part of this IRB application]. We will test this hypothesis through the following specific aims:

Aim 1: Test the hypothesis that local heat therapy improves muscle architecture, glucose tolerance, and frailty indicators similarly to HIIT in older prediabetic people.

Aim 2: Test the hypothesis that local heat therapy pre-conditioning improves the skeletal muscle response to HIIT in older prediabetic people.

Citations

6-5

Enter or attach the references cited .

The references below are from our Specific Aims and Research Strategy documents. Although they are not cited in the project summary, importance, and aim above, they are cited in the funding application.

ESI R01_Bibliography & References Cited.docx

7: Human Subjects

Please respond to the following statements/questions to help our IRB reviewers know more about the participants in this study.

7-1 Relationship with Participants

*required

7-1a **Is there the potential for any relationship between the researcher(s) and the proposed target population?**

(Note: if recruiting in the Lubbock (which includes TTU) community please click yes as students and employees are possible)

✓ Yes

*required

7-1a(1) **Explain**

Recruiting older adults with prediabetes in Lubbock; it is possible that other TTU employees could be recruited.

No

Coercion or Undue Influence

Guidance for IRB and Investigators

Coercion - Coercion entails influencing an individual's decision about whether or not to

7-2 do something by using explicit or implied threats (loss of good standing in a job, poor grades, etc.). This area also includes the perception of students as to any potential backlash for failing to take part in an instructor's research.

Undue Influence - This is defined as an offer of an excessive, unwarranted, inappropriate, or improper reward or other overture in order to obtain compliance (used in association with recruiting research participants).

*required

7-2a **Does this research involve participants who could be coerced or unduly influenced to participate or may feel that there is perceived coercion or undue influence (i.e., students, employees, prisoners, colleagues, friends/acquaintances, family members, etc.)?**

✔ Yes

*required

Describe the plan to mitigate (perceived) coercion or undue influence to participants.

7-2a(1) One example of mitigating coercion/undue influence would be to add a sentence to the consent form that clearly states their decision to participate, not participate, or withdraw in the research will not impact their employment status or academic standing.

We will emphasize to participants that their enrollment and continued participation is completely up to them. They can withdraw from the study at any time for any reason without consequences. The PIs of this study do not currently have any influence over the employment of individuals who would qualify for this study. Any future non-traditional student who might qualify will be assured that their grade(s) will noty be impacted, positively or negatively, by their participation or withdrawal. Colleagues in KSM who might qualify and enroll will be assured that our working relationships will not be impacted, positively or negatively, by their participation or withdrawal. The PIs are committed to ensuring that participation and/or withdrawal of KSM colleagues who might choose to participate and/or withdrawal does not impact our decisions for any future faculty votes, recommendations, etc. Finally, we will emphasize the importance of confiidentiality with our research team, **especially** when a participant might be a TTU (including KSM) employee.

No

7-3 Target Population

*required

7-3a **Describe the population of human subjects to be recruited and why this research specifically targets this group?**

In this study, participants with elevated blood glucose, prediabetes, or are at risk for developing diabetes; are sedentary will be recruited.

This population is targeted because the muscular pathophysiology of T2D (i.e., insulin resistance [IR], decreased skeletal muscle [SKM] mass and function) contributes to phenotypic frailty including slow gait speed, weakness, unintentional weight loss, fatigue, and low physical activity. Approximately 7-15% of community-dwelling older adults meet criteria for frailty, and 25-40% of these individuals are prediabetic. Moreover, a recent meta-analysis of community-based studies found that baseline T2D is associated with nearly 50% increased risk of frailty development.

Although exercise training is a key technique to manage metabolic disease and decrease T2D risk, in older adults at risk for T2D, compromised oxygen delivery and mitochondrial dysfunction result in exercise intolerance, a potential contributor to low exercise adherence. Thus, it is critical to find alternative means to manage metabolic disease risk. Heat therapy is one possible alternative. Heat therapy adaptations could improve exercise tolerance by preconditioning the microenvironment of skeletal muscle, likely enhancing exercise adherence and improving subsequent exercise adaptations.

*required

7-3b **Does your target population require specific characteristics?**

Yes, my target population **DOES** require specific characteristics.

✓ No, my target population **DOES NOT** require specific characteristics.

*required

7-3c **Age Range**

Check all that apply.

✓ Adults

*required

7-3c(1) **Enter the age range.**

≥60

Children

*required

7-3d **Enter the number of participants.**

27 men and 27 women

7-4 **Inclusion/Exclusion Criteria**

Enter or attach all criteria used to include or exclude participants for this research.

Individuals with elevated blood glucose, prediabetes, or are at risk for developing diabetes; are sedentary

Inclusion:
•Sedentary (structured exercise <30 minutes, 3x/week)
•Meet criteria for prediabetes (fasting blood glucose 100-125 mg/dl and/or hemoglobin A1c 5.7-6.4%)
•Age ≥ 60 years
• Body weight is at least 110 pounds
•Consume <8 (women) or <15 (men) alcohol-containing beverages per week
•Do not use nicotine or cannabis

7-4a

•Not taking any medications that could interfere with responses to the interventions (e.g., corticosteroids, opiates, benzodiazepines, tricyclic antidepressants, beta blockers, sulfonylureas, insulin, metformin, anticoagulants, barbiturates, insulin sensitizers, fibrates [PPAR gamma agonist])

Exclusion:
•History of peripheral neuropathies
•Currently taking prescription blood thinners
•Medical complications that could would contraindicate participation in the high intensity interval training (HIIT) intervention including: orthopedic complications that would limit the ability of the participant to perform cycling exercise, significant cardiovascular impairments (e.g., history of arrhythmias, severe uncontrolled hypertension, etc.), diagnosed metabolic disease (e.g., diabetes), renal disease, and cancer in remission for <6 months.
•Excessive subcutaneous fat over the muscle (greater than 1.5")
• Currently pregnant

We will screen for these criteria using the medical history form (attached). After inquiring with the participant, "Yes" to the following questions would result in exclusion from the study: 1, 3-5, 7-10, 13-15 (unless acute illness), 17 (lidocaine only), 18, and 20. Answers to other questions will be compared against inclusion/exclusion criteria (i.e., injuries, illnesses, chronic conditions, other reason that they should not exercise, etc.) to determine whether the participant meets all inclusion and no exclusion criteria. A key for research staff is included as a separate page in the form (beginning in Column Z on the spreadsheet).

7-4a(1) Inclusion/Exclusion Screening Form (if applicable)

R2_with KEY_HEAT study_Medical History Questionnaire.xlsx

7-5 End of Subject Participation

*required

Enter specific criteria used by the researchers to prematurely end a subject's participation in the study.

Sometimes a PI will decide to remove a participant from a research study. List any reasons you would anticipate that may require this removal.

7-5a *This is not a participant deciding they would like to stop/withdraw from the research. This is a researcher ending their participation. For example: unforeseen risk to the participants, risks to the other participants/research team members, non-adherence, disruptive behavior.*

The researchers will end participants' involvement in the study early if they do not follow the study's instructions, which include:

- Adopting a new lifestyle
- Starting a new exercise routine and/or diet program
- Taking any new supplements
- New medical conditions or complications that would place participants at increased risk
- Low adherence to interventions (<80% compliance)

7-6 Investigator Assurances

*required

7-6a Data Storage and Retention

✓ I will provide participants with information about data storage and retention if they withdraw from the study, when contacted by subjects per contact information provided in the consent form.

I will **NOT** provide participants with information about data storage and retention if they withdraw from the study, when contacted by subjects per contact information provided in the consent form.

*required

7-6b Participant Withdraw

✓ I will provide participants with information about how to withdraw from the study through the provided consent form.

I will **NOT** provide participants with information about how to withdraw from the study

through the provided consent form.

8: Recruitment Procedures and Material

Please respond to the following statements/questions.

8-1 **Recruitment Procedures**

*required

Describe the process of how participants will be recruited and provide the sequence of these events.

This section needs to include the details of each step for recruitment up to the point of consent. This includes pre-screening procedures.

8-1a All planned recruitment efforts and scripts will be approved by the Texas Tech University IRB. We will begin recruitment through the University Medical Center in partnership with Texas Tech University Health Sciences Center physicians who see patients from the community that may qualify. Email addresses and/or phone numbers will be obtained by searching for internal medicine, family medicine, geriatrics, and endocrinology clinics.

Electronic advertisements will be posted on listservs (e.g., TechAnnounce, Obesity Research Institute listserv) and on social media (e.g., Facebook). Social media announcements will specify targeting older adults (60+) in the Lubbock, TX area. Additionally, after site-specific approval, flyers will be distributed around the community to frequently visited locations such as grocery stores, pharmacies, and community centers.

Drs. Luk and Levitt will be responsible for executing recruitment efforts, collecting contact information for potential participants, conducting initial phone screenings, and following up with potential participants to schedule them for their informed consent and screening visit. Once a clinical coordinator is hired, these responsibilities will primarily shift to that person. The initial phone screening is to ensure participant is at least 60 years old, sedentary, and their physician has informed them they have elevated blood glucose, prediabetes, or are at risk for developing diabetes.

8-2 Recruitment Methods

Templates

*required

8-2a Select all methods of recruitment.

Check all that apply.

No Recruitment

✓ Oral Script

*required

8-2a(2) Enter or attach the Oral Script(s).

If more than one oral script is entered or attached, label each script separately.

Script for UMC and TTUHSC phone recruitment is attached

8-2a(2)a If you attach a document, state *See Attachment* in the text box above.

Script_UMC and TTUHSC recruitment.docx

✓ Tech Announce

*required

8-2a(3) Enter or attach TechAnnounce ad.

See attached

8-2a(3)a If you attach a document, state *See Attachment* in the text box above.

Tech Announce Ad - HEAT study.docx

✓ Recruiting Letter(s) or Email(s)

*required

8-2a(4) **Explain how email addresses or physical addresses of potential participants are obtained.**

We will search for any email addresses for UMC or TTUHSC physicians' offices.

*required

8-2a(5) **Enter or attach all recruiting emails or letters.**

If more than one email is entered or attached, label each separately.

Script attached

8-2a(5)a If you attach a document, state *See Attachment* in the text box above.

Script_UMC and TTUHSC recruitment.docx

Information Sheet

✓ Advertisement(s)

*required

8-2a(8) **Check all that apply.**

✓ Print Advertisement (eg. newspaper, magazine, fliers)

Website

✓ Social Media

*required

8-2a(9) **Enter or attach the advertisement.**

If more than one advertisement is entered, label each separately.

The attached flyer will be used for print and social media advertising.

8-2a(9)a If you attach a document, state *See Attachment* in the text box above.

R2_Flyer- HEAT study (1).doc

Subject/Participant Pools

Third Party Recruitment

Other

8-2b **Investigator Assurances**

*required

8-2b(1) **Permission to Recruit Participants**

✓ I will acquire proper permission prior to recruiting participants.

I will **NOT** acquire proper permission prior to recruiting participants.

8-3 **Permission to Conduct Research**

*required

8-3a **Is this research study being conducted in a setting that requires prior permission from an entity to conduct research on their premises (i.e., school system, business, government entity, etc.)?**

Yes

✓ No

9: Consent, Assent & Waivers

Visit the HRPP website for instructions and templates on the Consent Process, Assent Process with Minors, Short Form Consent Process, and Waivers of Consent.

Consent forms can be destroyed three years after the completion of the research. 45 CFR 46.115(7)(b).

9-1 **Consent**

*required

9-1a **Consent Process & Forms**

Check all that apply.

✓ Consent (Adult)

*required

9-1a(1) **Check all that apply.**

✓ Participant

Legal Authorized Representative Consent (LAR)

Parent/Legal Guardian

*required

Describe the process for obtaining consent. Include where and when the consent process takes place and who will be obtaining consent.

Drs. Luk or Levitt will provide a brief overview of the study using the information sheet and then seek informed consent from all potential participants in their offices in the KSM building to ensure privacy. All participants will be provided with copies of the information sheet and informed consent sheet. They will also be provided with a complete verbal explanation regarding the reasons for conducting the study, the procedures, risks, and benefits of the study, and any questions will be answered. At the end of the consent process, participants will be asked the following questions to establish capacity to consent:

9-1a(2)

Do you understand why this study is being done?
If you decide to participate in the study, what are some of the things you will be asked to do?
Describe some of the risks or discomforts you may experience if you participate in this study.
Will this study help you?
Do you have to be in this study?
What will happen if you decide not to be in the study?
Who should you contact if you have questions or experience a problem while in the study?

Correctly answering these questions will demonstrate an understanding of the study and determine the capacity to provide consent. Adults unable to demonstrate the capacity to consent will not be enrolled in the study.

*required

Enter or attach the consent form.

9-1a(3)

If more than one consent form is entered or attached, label each separately.

See attachment

If you attach a document, state *See Attachment* in the text box above.

9-1a(3)a

DXA Acknowledgement Form (1) (1).pdf

R2_no IM temp_Informed Consent - HEAT study_Laymen term .docx

*required

Will a Short Form Consent be used?

9-1a(4)

Only used in complex studies when the PI cannot be sure that a signed written consent is understood well enough to indicate a valid consent process.

Yes

✓ No

Assent (Minors & Individuals with Impaired Decision Making Skills that cannot consent for themselves)

Texas Tech Neuroimaging Institute (TTNI) Consent Form

No consent will be obtained.

9-2 **Waivers**

A consent form must still be included in the documentation.

9-2a **Check all that apply.**

Request for a Waiver of Written Consent

Request for a Waiver or Alteration of the Elements of Consent

FERPA Authorization

IRB approval or determination of exemption of the research project does not constitute institutional permission to access the requested records.

9-3 **Guidance:** FERPA applies to personally identifiable information in educational records. This includes items such as the student's name, names of family members, addresses, personal identifiers such as social security numbers, and personal characteristics or

other information that make the student's identity easily traceable.

Educational records are all records that contain information directly related to a student and are maintained by an educational agency or institution, or by a party acting on its behalf. A record means any information recorded in any way, including handwriting, print, tape, film, microfilm, microfiche, and digital images.

9-3a **Will you be using student records protected under FERPA?**

Yes

✓ No

HIPAA Authorization or Waiver

9-4

Indicate whether you are obtaining Participant Authorization or requesting a Full or Partial Waiver of HIPAA Authorization.

9-4a **Select the appropriate authorization.**

Participant Authorization of Protected Health Information

Full or Partial Waiver of Protected Health Information

Complete the following section.

Data Collection

10-1 IRB members find it very helpful to have copies of all instruments. Reading the instrument(s) is essential to the board's assessment of participant risk and burden and the adequacy of the study description in the informed consent. Occasionally, however, investigators do not include all or full copies of instruments on the grounds that the instrument is copyrighted and in the belief that making copies to submit with an IRB application is a violation of copyright law. However, instrument developers and publishers should have a reasonable expectation that instrument users must obtain IRB approval of their research and that approval requires review of all study materials. Further, IRB members are well aware of their own ethical responsibility to honor copyright and purchase contracts by investigators and to refrain from use of copyrighted material for purposes other than those related to their IRB obligations. If records are being withheld pursuant to a Non-Disclosure Agreement (NDA), you (the investigator) are expected to provide the relevant portion of the NDA or any similar documentation requiring the records be withheld specifically from the IRB. If the NDA or license precludes sharing with the IRB, it is the PI's responsibility to get approval from the company or licensor to share the instrument(s) with TTU's IRB for review.

*required

Describe the sequence of events and timeline the participants will experience.

A randomized controlled intervention design will be used. The study consists of 2 phases, each 12-weeks in duration.

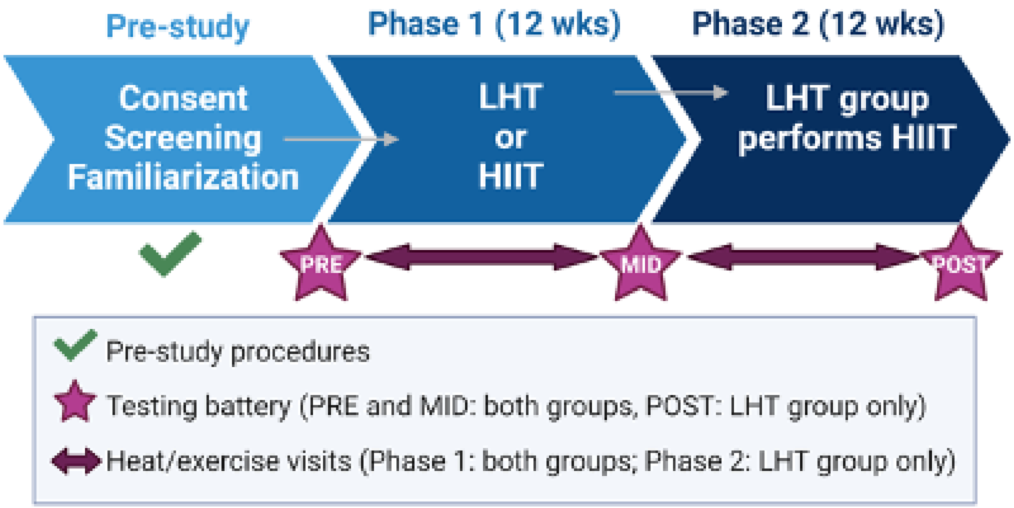

Visit 1: Consent and screening: (~1h)
•Participant will need to fast (i.e., no food or beverages other than water; plain water allowed and encouraged) for 12h before consent and screening.
•After an explanation of study requirements, participant will review and sign the informed consent form.
•We will collect a drop of participant blood using finger prick to determine their fasting blood glucose and HbA1c concentrations to make sure they have met the inclusion criteria.
•We will put a small drop of jelly on participant's thigh and use an ultrasound machine to image the participant's vastus lateralis muscle. This will help us make sure that the lidocaine can reach the muscle (i.e., subcutaneous fat over the muscle no greater than 1.5").

Visit 2: Familiarization: (~2h)
1.Bike Settings:
•We will predetermine participant bike settings (e.g., seat height, etc.) for exercise capacity test and HIIT.

2.Establish heat pad temperature:
•We're going to determine the appropriate temperature for a heat pad to warm participant's skin temperature to 40-43 C for 90 min.

To estimate the intramuscular temperature using skin temperature, we will utilize the following validated estimation equations (Flouris et al. 2014). These equations has been previously used to predict intramuscular temperature with heat and exercise (Maunder et al. 2024; Soo et al., 2019).

Equations specific to estimating intramuscular temperature for the vastus lateralis:
Tm_{rest}=iDISK·0.597−$iDISK_{lag2}$·0.439+$iDISK_{lag3}$·0.554−$iDISK_{lag4}$·0.709+14.767
Tm_{ex}=iDISK·0.599−$iDISK_{lag4}$·0.311+15.63
Tm_{rec} =iDISK·0.657−iDISKlag4·0.538+13.283

where:
Tm_{rest} = predicted intramuscular temperature of the vastus lateralis at rest
Tm_{ex} = predicted intramuscular temperature of the vastus lateralis during exercise

Tm_{rec} = predicted intramuscular temperature of the vastus lateralis during recovery
iDISK = temperature reading of the iDISK
$iDISK_{lag"n"}$ = temperature reading of the iDISK at "*n*" minutes prior (i.e., lag)

Steps:

1. Temperature sensors will be placed on the skin above the vastus lateralis (15 cm superior to the patella).
2. An insulation disk will be placed directly on top of the temperature sensors and secured with adhesive dressing.
3. Heat pad will be placed on the thigh and initially set at 40°C for about 10 min to allow intramuscular temperature to reach a steady state.
4. Skin temperature will be monitored continuously for 90 min and estimated intramuscular temperature will be calculated every 5 min using the above equation.

o If the estimated intramuscular temperature is below 40°C, we will increase the heat pad temperature in an increment of 0.5°C until the estimated intramuscular temperature reaches 40°C.

o If the estimated intramuscular temperature is above 40°C, we will decrease the heat pad temperature by an increment of 0.5°C until the estimated intramuscular temperature reaches 40°C using the recovery equation (i.e. Tm_{rec}; intramuscular temperature is decreasing)

5. Once the estimated intramuscular temperature reaches 40°C, the heat pad temperature will be set and temperature (skin and estimated intramuscular temperatures) will be monitored for the remainder of the session.

o *Note:* We are aiming for an intramuscular temperature of 40°C. We anticipate that this will be well-tolerated because 1) core temperature remains stable during local heating on the thigh and 2) intramuscular temperatures of 40°C (vastus lateralis) have been documented in human participants without adverse effects. However, if a participant is unable to tolerate this, we will reduce the initial target intramuscular temperature as needed to a minimum of 38°C based on intramuscular adaptations previously documented in the literature.

3.Questionnaires:

• Participants will be familiarized with a set of questionnaires related to:

1. Unintentional weight loss (self-report, ≥10 lbs or ≥5% of body mass in the past year or between major study time points)
2. Fatigue (SF-36 energy subscale)
3. Physical activity (International Physical Activity Questionnaire)
4. 24h Diet Recall (ASA24: Healthy Eating Index 1-100 scale).
5.Sleep quality and well-being (7-point likert scale)
6.Alcohol use (Timeline Follow Back)

PRE Testing (Before Phase 1 Begin): All major measurements for the PRE Testing will be completed over 3 separate days. These major measurements will also be conducted at MID (end of Phase 1), and POST (end of Phase 2). However, the sequence of these major

measurements will be different.

Preparing for Visit 3-5:
1. Overnight fast for 12h (only water allowed)
2. No alcohol for 24h
3. No medications or supplements from this list for 48h:
•Tylenol (acetaminophen)
•Ibuprofen

Visit 3 (Day 1 of the PRE Testing): (~ 1h)
1.Anthropometric Measurements:
•We will measure your height, weight, waist-hip ratio, and a body composition scan will be performed using dual X-ray absorptiometry (DXA).
2.Muscle Biopsy: We will take muscle sample (~200 mg) from your outer thigh. This procedure will last about 30 minutes.

Visit 4 (Day 2 of the PRE Testing): (~ 3 h)
1.Oral Glucose Tolerance test (OGTT):
• Participants will consume a beverage containing 75 g glucose (TRUTOL, ThermoFisher Scientific).
•Before and at 30 min, 1 hr, and 2 hrs after beverage consumption, venous blood will be collected into vacuum tubes using the standard venipuncture technique.
2. Questionnaires: During the OGTT, you will complete the following questionnaires.
10-1a Questionnaires 2-6 will also be filled out weekly during the study.
1.Unintentional weight loss (self-report, ≥10 lbs or ≥5% of body mass in the past year or between major study time points)
2.Fatigue (SF-36 energy subscale)
3.Physical activity (International Physical Activity Questionnaire)
4.24h Diet Recall (ASA24: Healthy Eating Index 1-100 scale).
5.Sleep quality and well-being (7-point likert scale)
6.Alcohol use (Timeline Follow Back)

Visit 5 (Day 3 of the PRE Testing) (~1.5h)
1.Physical Function (part of Phenotypic Frailty Assessment) Testing:
•Perform the gait speed test (4-m walk test), 30-second Sit-to-stand test, and handgrip strength test on both hands.

2.Exercise Capacity Test:
•Perform an exercise capacity (VO2peak) test using a cycle ergometer.

Phase 1:

For the Local Heat Therapy Groups
If participants are assigned to one of the local heat therapy(LHT) groups (Sham (CON) and LHT), they will be sent home with 2 heating pads and apply local heat to their thigh 7 days for 12 weeks (6 sessions at home and 1 session at the Lab). Participants assigned to the heating group will be advised to set their heat pads to the temperature previously determined

to achive an intramuscular temperature of up to 40 C; participants assigned to the sham (CON) group will be advised to set their heat pads to a lower tempereature (e.g., 36 C) to maintain a neutral intramuscualr temperature.

At home Local Heat Therapy Sessions (6d/wk):
• Participants will be encouraged to empty their bladder before the start of each session to minimize the number of times they have to remove the heat pads during a session. For any reason they have to remove the heat pad, they will record it.

•For each session, participant will place the heating pads to completely surround each thigh with a snug fit. The heating pads will be secured in place using Velcro and set to the predetermined temperature continuously for 90 minutes.

• Participants will record the following for each session:
1. Start time
2. End time
3. Number of times you need to remove the heat pad during a session
4. The duration for which it is removed
5. Discomfort

•There is no restriction on the location (at home or in the office), time of day, and seated activity during the session (e.g., watching TV, reading a book, etc.).

In-Lab Local Heat Therapy Sessions (1d/wk): Visit 6-17 (~2h each)
•During the weekly lab visit (1d/wk), comfort will be monitored, and we will collect the logs of participants' at-home sessions to confirm compliance.
•The following will also be assessed each week: sleep quality and activity (Hooper questionnaire and International Physical Activity Questionnaire [IPAQ]), alcohol use (Timeline Follow-back), 24-hr diet recall (ASA24), and anthropometrics (height, body mass, waist circumference, and hip circumference)

For the High_Intensity Training (HITT) Groups

In Lab HIIT Session: Visit 6-41 (Total of 36 visits) (~1h each)
•Participants assigned to the HIIT group will arrive at the laboratory 3 d/wk for 12 weeks. They will be fitted with a heart rate monitor (Polar) and skin temperature sensors (thigh) and will perform a 3-minute cycling warm-up at 30W before beginning the HIIT protocol.
•The following will also be assessed each week: sleep quality and activity (Hooper questionnaire and International Physical Activity Questionnaire [IPAQ]), alcohol use (Timeline Follow-back), 24-hr diet recall (ASA24), and anthropometrics (height, body mass, waist circumference, and hip circumference)

MID Testing (End of Phase 1):
All major measurements for the MID Testing is identical to PRE Testing except for the order of measurements. Detail protocols, please refer to PRE Testing.

Preparing for the following Visits:

1. Overnight fast for 12h (only water allowed)
2. No alcohol for 24h
3. No medications or supplements from this list for 48h:
•Tylenol (acetaminophen)
•Ibuprofen

Visit 18 (LHT)/ Visit 42 (HIIT): Day 1 MID Testing (~1h)
1. Anthropometric measurements
2. Frailty test
3. Exercise capacity test

Visit 19 (LHT)/ Visit 43 (HIIT): Day 2 MID Testing (~3h)
1.OGTT
2.Questionnaires

Visit 20 (LHT)/ Visit 44 (HIIT): Day 3 MID Testing (~ 1.5h)
1. Muscle biopsy

Phase 2:
Visit 21- 56 - Phase 2 (Sham (CON) and LHT groups): Participants assigned to the Sham (CON) and LHT groups will continue to Phase 2. In Phase 2, they will complete HIIT 3d/wk for 12 weeks. For detailed protocols, please refer to above (HIIT group).

POST Testing (End of Phase 2):
All major measurements and order of measurements for the POST Testing is identical to MID Testing. Detail protocols, please refer to PRE Testing.

Preparing for the following Visits:
1.Overnight fast for 12h (only water allowed)
2.No alcohol for 24h
3.No medications or supplements from this list for 48h:
•Tylenol (acetaminophen)
•Ibuprofen

Visit 57 (LHT): Day 1 POST Testing (~1h)
1. Anthropometric measurements
2. Frailty test
3. Exercise capacity test

Visit 58 (LHT): Day 2 POST Testing (~3h)
1.OGTT
2.Questionnaires

Visit 59 (LHT): Day 3 POST Testing (~ 1.5h)
1. Muscle biopsy

*For Sham (CON) and LHT groups, participants will have a total of **59 Lab visits** with a total time spent for about **79.5h (Lab)** plus **108h (at home heat pad therapy)**.*

*For the HIIT group, participants will have a total of **44 Lab visits** with a total time spent for about **50h**.*

*required

10-1a(1) **What is the total time commitment for the participant?**

For CON (Sham, considered under the "LHT groups" in the consent form) or true LHT group, the duration will be approximately 7 months (testing: 1 month total; Phase 1: 3 months, Phase 2: 3 months) with a total of about 79.5h (lab) plus 108h (at home heat pad therapy). For HIIT group, the participation duration will last for approximately 4 months (testing: 3 wks total; Phase 1: 3 months) with a total of about 50h.

*required

10-1b **Select procedure(s) to be used.**

Check all that apply.

✓ Surveys

*required

10-1b(1) **Check all that apply.**

Online

✓ On-Site

Mailing

Other

*required

10-1b(2) **How much participant time is involved?**

The time commitment for surveys is included in the overall study visit time.

*required

Enter or attach the survey(s) questions or a list of survey items.

If multiple surveys are used, label them separately.

Hooper well-being survey questions (completed weekly; scored on 1-7 scale, low (fatigue, stress, soreness) or good (sleep quality) and 7 indicates high (fa soreness) or bad (sleep quality):

1. Please rate your fatigue level over the past 24 hours.
2. Please rate your level of stress over the past 24 hours.
3. Please rate your level of soreness over the past 24 hours.
4. Please rate the quality of sleep you had last night.

10-1b(3) Automated Self-Administered 24-hour Dietary Assessment Tool (ASA-24; cor Registration for use of the ASA-24 will be completed upon Notice of Award ar monies. A link to a demonstration website for the ASA-24 is here: https://asa2 (2024 version)

Unintentional weight loss (at PRE, MID, and POST testing): In the past year [I 12 weeks [MID and POST], have you unintentionally lost body weight? If so, h

Other surveys: see attachments.
1) Medical history (screening visit)
2) International Physical Activity Questionnaire (IPAQ)-SF (weekly)
3) Timeline Follow Back (TLFB, weekly)
4) 36-item Short Form Health Survey (SF-36) energy subscale (weekly; only i and 31;
scored per https://www.rand.org/health-care/surveys_tools/mos/36-item-short

If you attach a document, state *See Attachment* in the text box above.

10-1b(3)a

IPAQ-SF.pdf

Medical History Questionnaire (3).xls

TLFB_Alcoholinstructions.pdf

TLFB_ALoverview.docx

SF-36.pdf

*required

Describe the process for the distribution and return of surveys.

10-1b(4) Surveys questions will be uploaded into Research Electronic Data Capture (REDCap), a free, secure, web-based application designed to support data capture. Researchers will assist particiants to electronically complete surveys during their laboratory visits.

Interviews

Focus Groups

Observation

Non-Invasive Procedures (MRI, X-Rays, etc.)
Invasive Medical Procedures (Blood draws, skin pricks, biopsies, etc.)

✓ Intervention

*required

10-1b(25) **How much participant time is involved?**

LHT groups ~79.5h (lab) plus 108h (at home heat therapy) and HIIT group ~50h in the laboratory

*required

10-1b(26) **Where will the intervention(s) take place?**

At home (Sham (CON) and LHT groups) and KSM building (all groups)

*required

10-1b(27) **Describe in detail the intervention procedures.**

Attach any documentation if applicable.

See attachment. The LHT group described in the Informed Consent document includes LHT (heat) and CON (control; sham - low-temperature heat pads).

10-1b(27)a If you attach a document, state *See Attachment* in the text box above.

[Revision_no IM_Detail the intervention procedures.docx]

*required

Who will be conducting the intervention(s)? What are their qualifications?

Attach any documentation if applicable.

For Oral Glucose Tolerance Testing (OGTT): Drs. Luk and Levitt are experienced in phlebotomy; Dr. Levitt has carried out the OGTT procedure. Additional study team members are trained in phlebotomy (Alexandra Khartabil and Casey Appell) and will help carry out this procedure under

the supervision of Drs. Luk and Levitt.

For body composition measurement, Drs. Luk and Levitt are certified DEXA technicians in the State of Texas and are approved to operate the DEXA housed in the Department of Kinesiology & Sport Management (KSM) at Texas Tech University (TTU). Their students trained on use of the DEXA will carry out this procedure under their supervision.

All biopsy procedures will be performed by Dr. Hui-Ying Luk, who is not a licensed physician but is trained specifically in the muscle biopsy procedure. Robyn Richmond, M.D. (robyn.richmond@ttuhsc.edu) will oversee the procedure (i.e., approve safety protocols, provide technical and medical advice regarding the procedures and potential adverse effects of biopsy). Dr. Richmond will determine the competency of the person who will perform the preparation (sterilized procedures, lidocaine injection) and the operation (maintaining sterile procedure for the primary operator: Dr. Luk). Since the assistant (applying suction through a syringe) does not
10-1b(28) have direct contact with the participant and does not require sterile procedures, the competency of the assistant will be determined by Dr. Luk. Our members are not going to diagnose these symptoms, instead, if the participant is self-reporting pain, erythema (warmth), or ecchymosis (bruising) to us, we will advise them to use a cold pack. Also, our members will follow up with them regarding the self-reported signs. Similarly, if the participant is seeing and self-reporting signs of infection, inflammation, hematoma (e.g., prolonged redness, puss formation, increasing pain, bruising at the site of biopsy, and fever, etc.), and numbness at the site of biopsy, we will refer them to consult with a medical provider. Lastly, an individual with CPR training (current) will be on-site within close proximity to the subject during procedures.

For exercise testing and intervention, Drs. Luk and Levitt have experience in exercise testing and intervention and are Certified Strength and Conditioning Specialists (CSCS) through the National Strength and Conditioning Association (NSCA). The proposed exercise capacity testing and HIIT intervention have been safely and successfully implemented in older adult participants (e.g. PMID: 29077855, 28273480). Dr. Luk and Levitt will train and supervise all personnel (graduate and undergraduate students on this IRB application) in conducting the exercise testing and intervention included herein. Only study personnel with currect CPR and first aid training will perform exercise testing and intervention. Dr. Levitt has an up-to-date CPR and first aid certification, and has held valid CPR certification continuously since 2003.

For frailty risk assessment, Dr. Levitt has assessed frailty risk in people living with HIV. She will train and supervise all personnel (graduate and undergraduate students on this IRB application) in conducting the physical function testing to assess frailty risk.

10-1b(28)a If you attach a document, state *See Attachment* in the text box above.

incidents updated (1) (2) (2) (2) (1).docx

Other

10-2 **Deception**

The result of investigators providing false or incomplete information to participants for the purpose of misleading research subjects.

*required

10-2a **Does this research project involve the use of deception?**

✓ Yes

*required

10-2a(1) **Explain why deception is necessary.**

A sham group is needed for scientific purposes.

No

10-3 **Debriefing**

10-3a **Will a debriefing script be used at the conclusion of the research?**

✓ Yes

*required

10-3a(1) **Select the reason for needing a debriefing script.**

✓ Deception

Sexual Misconduct

Mental Health, Self-harm, Substance Abuse, Criminal Behavior

✓ Education Tool

*required

10-3a(2) **Enter or attach a copy of the debriefing script.**

See attachment

10-3a(2)a If you attach a document, state *See Attachment* in the text box above.

Biopsy care kit.doc

Deception Script.docx

*required

10-3a(3) **Provide information on who will conduct the debriefing and how it will be delivered to the participant.**

Dr. Luk, Dr. Levitt, or clinical coordinator (future) will conduct the debriefing.

Upon the completion of the biopsy procedures, we will provide a take-home kit (i.e., notes and necessary consumables, e.g., extra bandage, alcohol wipes). Also, we will verbally explain the biopsy care instructions and a list of drugs that can increase bleeding (see the attachment), especially focusing on the medication that should be avoided. Furthermore, our research team will follow up with the participant in the following two days by text/call to make sure no adverse effect was observed (swelling and warmth at the site of biopsy). You can also send us a photo of the biopsy site through email or text message if you have any concerns. We will look at your biopsy site on your next visit.

For the control group (CON), we will disclose that the temperature setting for the heat pad used during the study was for control purposes, and we will specify the temperature they need to set to achieve an intramuscular temperature of 40 degrees Celsius.

No

10-4 TTU Compliance Committees

Additional approvals

*required

10-4a **Does this research require internal approval from another TTU compliance committee or entity?**

✓ Yes

10-4a(1) **Select the TTU Committee(s) that grants approval to conduct research at that location and attach the approval letter.**

Texas Tech Neuroimaging Institute (TTNI)

✓ TTU Institutional Biosafety Committee (IBC)

*required

10-4a(1)b **Attach the approval letter.**

IBC-2023-1083 approval_with biopsy protocol.pdf

TTU Institutional Laboratory Safety Committee (ILSC)

Institutional Animal Care and Use Committee (IACUC)

TTU Radiation and Laser Safety Committee (RLSC)

Other

No

Complete the following section.

11-1 Personal Identifiers

*required

11-1a Select personal identifiers that will be collected for the purpose of this research.

Check any identifier you will be in possession of during the research process.

✓ Names

✓ Telephone numbers

✓ Any elements of dates (other than year) for dates directly related to an individual, birth date, admission date, discharge date, date of death. For ages over 89 all elements of dates (including year) indicative of such age, expect that ages and elements may be aggregated into a single category of age 90 and older

Any geographic subdivisions smaller than a state, including street address, city, county, precinct, zip code and their equivalent geocodes except for the initial three digits of a zip code

Fax numbers

✓ E-mail addresses

Social security number

Medical record number

Student ID username/number

Health plan beneficiary numbers

Account numbers

Certificate/license numbers

Vehicle identifiers and serial numbers (VIN), including license tag identification

Device identifiers and serial numbers (e.g., implanted medical device)

Web universal resource locators (URLs)

Internet protocol (IP) address numbers

Biometric identifiers, including finger and voice prints

Full face photographic images and any comparable images (i.e., videos, etc.)

✓ Biological specimens

Any other unique identifying number, code, or characteristic, other than dummy identifiers that are not derived from actual identifiers and for which the re-identification key is maintained by the health care provider and not disclosed to the researcher

No personal identifiers will be collected

11-2 Privacy of Participants

*required

11-2a **Describe how you will protect the privacy of the participants.**

Consider how you will protect the participant's privacy during:

- Recruitment
- Consent
- Data Collection
- Storage of Data
- Analysis of Data
- Findings

Data Safety:
To protect against breach of confidentiality, any hard copy materials (e.g., informed consent form, any hard copies of exercise records with heart rate and rating of perceived exertion

data, etc.) will be kept in a locked drawer in Dr. Luk's private office. The informed consent form and demographics files will be the only places participants' names appear, and only the PIs and essential study personnel will have access. Participant samples, data, and records will be deidentified and labeled using a unique ID number. Data, images, and files generated from the project will always be stored in raw form, separate from files with calculations and analyses. Lastly, all electronic material, including data from questionnaires administered using computer-assisted interview techniques (i.e., no hard copy) will be stored in a secure database (REDCap).

11-3 De-Identifying Data

[Anonymous v. Identifiable Data]

*required

11-3a **Explain how you will de-identify the data collected. If data will be given a code, explain this process.**

For example:
Personal identifiers will be removed from the data and replaced with a participant code. The participant code will be used on the individual's data. The master list containing the personal identifier(s) and participant code will be in a separate document stored away from the data. The master list and data will be password protected. The passwords will be different for the master list and data.

For each identifier explain when and how you will code/de-identify/destroy the identifier.
Consider the following:

- What identifiers are associated with data?
- What identifiers are NOT associated with the data?
- What descriptors can be used to identify someone? For example: schools, towns, buildings, colleagues, companies, titles, etc.

Upon participant's admission to the study, a unique study identification code (ID codes) will be assigned to the participant in the order they are enrolled. The file containing participants' names and IDs will be stored electronically in REDCap.

Thereafter, all data will be kept in coded participant files. All trained investigators, professional staff, and technicians are aware of the confidentiality involved with this study.

Identifiable data will not be available or divulged to anyone outside of the experimental research team and data files will be kept for at least 3 years after the study is terminated. The confidentiality of individual information will be maintained in any publications or presentations regarding this study.

11-4 **Data Transcription**

*required

11-4a **Select who will transcribe audio or voice recording.**

Check all that apply.

Member of the research team/lab

Professional transcription company

Other

✓ Not applicable

11-5 **Transfer of Data**

*required

11-5a **Will data be transferred from one location to another?**

Transfer of data can be done electronically (email, removable storage device, shared drives, remote access, hyperlinks, etc.) and/or physically (carrying, mailing, etc).

✓ Yes

*required

Explain how it will be securely transported and by whom.

1. Data type (human subjects data only)

A. **Types and amount of scientific data expected to be generated in the project:**
Data from human subjects: Demographic and anthropometric (e.g., age, sex, race, ethnicity, body mass, body composition, height), clinical (e.g., medical history, oral glucose tolerance test parameters, and questionnaires about alcohol use, diet, activity, sleep, changes in body mass, and fatigue), functional (e.g., exercise capacity, grip and leg strength, gait speed), and molecular (e.g., type I and II muscle fiber cross-sectional area, capillarization, mitochondrial enzyme activity, mitochondrial and TRPV1 protein expression, mitochondrial DNA quantification, and mitochondrial content and morphology) data will be collected from 54 older adults at risk for type 2 diabetes (described in detail in the “Human Subjects” attachment) at several time points throughout the study. Weekly assessments will include body mass measurement, diet and alcohol questionnaires, and activity and sleep monitoring via and self-report questionnaires. All other data will be collected only at the beginning of the study (i.e., demographics, medical history) or at the 2-3 major time points. Participants assigned to the high-intensity interval training (HIIT) group (n=18) for phase 1 will have 2 major time points (before [T1] and after [T2] phase 1). Participants assigned to the LHT (n=18) and CON (n=18) groups for phase 1 and HIIT for phase 2 will have 3 major time points (before phase 1 [T1], between phases 1 and 2 [T2], and after phase 2 [T3]).

Large-scale data sets: It is possible that large scale mitochondrial genomic data (human participants) and proteomic data (cell culture) could be generated from this project if alternative approaches must be used as described in the research strategy.

The total amount of anticipated data if alternative approaches are not needed is ~25 GB of data.

B. **Scientific data that will be preserved, shared, and the rationale for doing so:** All raw data generated from this project will be stored in a password-protected manner on cloud-based servers at Texas Tech University. Cleaned data in an analysis-ready format will be stored in REDCap and periodically uploaded to repositories. Data of sufficient quality will be uploaded to allow others to validate and replicate research findings described in the Aims.

C. **Metadata, other relevant data, and associated documentation**: In alignment with the Data Submission Worksheet for the Aging Research Biobank, the following will be submitted to accompany data derived from human subjects:
•Full study protocol
•Names and descriptions of data sets
•Manual of operations

•Annotated data collection forms
•Data dictionary
•Documentation of calculated variables
•Summary of deidentification and crosslinking of study ID with new randomized ID
•Any changes made to the protocol over time
•Frozen datasets used for the primary publication, when applicable
Similarly, the full study protocol, dataset names/descriptions, data dictionary, documentation of calculated variables, and any changes to the protocol over time will be deposited in TTU Dataverse for molecular procedures.

2. **Related tools, software, and/or code:** Statistical analyses will be run using standard statistical software (SPSS version 29) and graphs made using GraphPad Prism (Version 9). Image processing will be performed using ImageJ and in part using custom Python code to automate quantification where appropriate.

11-5a(1)

3. **Standards:** No consensus standards of data formatting such as Dublin or Darwin core exist for our discipline. Data dictionaries defining each variable and decoding any coded responses will accompany all uploaded data.

4. **Data preservation, access, and associated timelines:**
A. Repository where scientific data and metadata will be archived:
Human subjects data: NIA's Aging Research Biobank
Molecular data: TTU Dataverse Collection
Mitochondrial genomic data: Database of Genotypes and Phenotypes (dbGaP; if performed)
Custom code: GitHub

B. How scientific data will be findable and identifiable: Data will be findable for the research community through the repositories listed above. Collections will be established in the Aging Research Biobank and in Dataverse when this application is funded. Collections will be established on dbGaP should a large data set be generated. Data stored in the Aging Research Biobank will be made available by reasonable request, and this will be noted in the "Data Availability Statement" in associated publications. When data are made public via Dataverse, a digital object identifier (DOI) will be assigned. This DOI be referenced in associated publications to allow the research community access to the data used in each publication. If dbGaP is used, researchers can submit requests to dbGaP to access and browse data, and this information will be included in any associated publications. Any custom code will be shared on GitHub, accessible by searching "rbudnar" on GitHub. The readme.md file for the project will include instructions for the analyses and rationale for parameter choices.

C. When and how long the scientific data will be made available: Data associated with each publication will be made available when preprints are available and will continue to be available on the repositories and long as

they are supported. Any data that are not yet available by the end of the award period will be made available at that time.

5. Access, distribution, or reuse considerations:
A. Factors affecting subsequent access, distribution, or reuse of scientific data: Broad data sharing will be included in the informed consent form. Data deidentification, linking data using unique identifiers, protection of privacy, and making data available will be explained to participants and any questions answered as part of the informed consent process.

B. Whether access to scientific data will be controlled: Access to human subjects data will be controlled. Requests will be submitted through the appropriate repository (Aging Research Biobank, Dataverse, dbGaP).

C. Protections for privacy, rights, and confidentiality of human research participants: The only location that personal information will be paired with participant ID will be on a single administrative data collection form in the secure REDCap database. This is necessary because data will be collected from the same participants and multiple time points. However, each participant will be assigned an ID code and only the ID code will be linked to data so that the data are not identifiable. Data from human participants will be further deidentified upon submission to the repository(ies) and linked only through the study ID. The Aging Research Biobank further anonymizes data by randomly generating a new ID for each subject. dbGaP interfaces with the Aging Research Biobank so clinical and mitochondrial genomic data (if generated) can be linked, using only the randomly generated ID. Further, access to these datasets will be controlled and require that users register and submit reasonable requests. The PIs attest that no attempt will be made to reidentify participants from deidentified data.

6. Oversight of DMS: Drs. Hui-Ying Luk and Danielle Levitt (PIs) will deposit clinical and associated data into the NIA Aging Research Biobank, TTU Dataverse, and into dbGaP if mitochondrial genomic data are generated. Data upload will occur when publications associated with each data set are submitted and made public (where applicable) upon acceptance. Data sharing will be included in the annual progress report.

No

Certificate of Confidentiality

*required

11-6a **Has a Certificate of Confidentiality been requested?**

Yes

✓ No

11-7 Investigator Assurances

*required

11-7a **Data Security**

✓ I will protect the data through at least one of these security measures: encryption software, password protection, and /or physical barrier (locked doors, file cabinets, etc.).

I will **NOT** protect the data through at least one of these security measures: encryption software, password protection, and /or physical barrier (locked doors, file cabinets, etc.).

*required

11-7b **Access to Data**

✓ I will only allow the research team access to the identifiable data.

I will allow others outside of the research team to have access to identifiable data.

*required

11-7c **Destruction of Personal Identifiers**

✓ I will destroy personal identifiers prior to closing the IRB with the exception of the consent forms.

I will **NOT** destroy personal identifiers prior to closing the IRB with the exception of the consent forms.

---

*required

11-7d **Retention Policy**

✓ I will keep de-identified data and/or consent forms for at least 3 years following the closure of the IRB.

I will **NOT** keep de-identified data and/or consent forms for at least 3 years following the closure of the IRB.

---

*required

11-7e **Data Repository**

✓ I will post the de-identified data to a data repository.

I will **NOT** post the de-identified data to a data repository.

12: Benefits & Risks

Please respond to the following statements/questions to help our IRB reviewers recognize the benefits and risks associated with this study.

12-1 **Benefits**

*required

Describe the potential direct benefit(s) to subjects.

Payments to subjects, course credit or contribution to science should not be included in this section.

12-1a

All efforts will be made throughout the study to minimize any study-related risks to subjects. The Physical Activity Guidelines for Americans, 2nd Edition (2018), suggest that adults should engage in at least 150 minutes per week of moderate-intensity aerobic exercise (or an equivalent 75 minutes per week of vigorous-intensity aerobic exercise, including HIIT) to maximize the many health benefits of aerobic exercise. Even if people do not meet the minimum of 150 minutes, smaller amounts of exercise are still beneficial. Participating in this study will provide participants with a structured environment to help meet that exercise goal during one of the two phases. Among anticipated benefits from exercise, we emphasize that there might be metabolic health benefits and decreased frailty risk, particularly because participants will be prediabetic older adults, indicating risk for the development of type 2 diabetes and frailty.

Furthermore, benefits of heat therapy are becoming increasingly recognized and we anticipate that participants assigned to the local heat therapy group will have improved skeletal muscle health, glycemic control, and decreased frailty risk. Because of the benefits anticipated from local heat therapy, all participants will be provided with heating pads regardless of group. The HIIT group will be provided with heating pads and instructions for use at the end of their participation (T2). The control group will be provided with heating pads but instructed to use a lower heat setting T1. At the end of their study participation (T3), they will be provided with instructions for use. Regardless of group, participants will have regular interaction with members of the study team. Together with exercise, prosocial interaction may have psychological benefits.

Participants will be provided with the following non-diagnostic results from testing performed at each major time point (PRE, MID, and POST):

- Dual-energy X-ray absorptiometry (DEXA) reports: Body composition and bone mineral density compared to age- and sex-specific norms.
- Body mass index (BMI): Value calculated from body mass and height (kg/m^2) and BMI category (i.e., underweight, normal weight, overweight, obese).
- Gait speed: 4-m walk time compared to age- and sex- specific norms.
- Muscle strength: Average grip strength (3 trials per hand) and 30-second sit-to-stand test with comparison to age- and sex- specific norms.
- Exercise capacity: VO_{2peak} and comparison to age- and sex-specific norms.
- Blood sugar control: If fasting blood sugar, HbA1c, or 2-hr glucose values after the glucose tolerance test fall into the diabetic range at any time point, the participant will receive a letter advising you of the laboratory measurement and to follow up with your physician.

12-2 Risks

*required

Describe any potential risk to subjects and how you will minimize that risk.

While exercise and heat have many potential benefits, they always carry possible risks. These risks are minimal, and the benefits of exercise and heat therapy largely outweigh the risks of a sedentary lifestyle. Those with higher-than-average risk for complications of exercise (e.g., preexisting cardiovascular conditions) will be excluded from the study. Risks of sample collection are also minimal, and the precautions taken will further offset these risks. The qualifications of the PIs and the protections in place for human subjects will ensure a safe environment for study participation.

Protections against risk
Strategies for minimizing risks associated with medical history and questionnaires, collection of biological specimens, and exercise are described below. All procedures will be fully approved by the TTU IRB. Drs. Luk and Levitt are trained in phlebotomy and muscle biopsies techniques and have previously conducted these procedures in IRB-approved studies. Dr. Luk is currently approved and actively performing muscle biopsies at TTU, and Dr. Levitt is in the process of obtaining such approval. All study personnel will be required to maintain valid CPR and First Aid certifications, and emergency contact information will be kept on hand. All personal information and data will be kept in a secure database (REDCap).

Protection against and minimization of risks by procedure:
•Interviews and completing questionnaires: To minimize risks of emotional stress and fatigue, the length and number of questionnaires will be kept to a minimum, and the study team will establish rapport with participants to minimize emotional stress. Contact information for

guidance counselors will be available. Protection against breach of confidentiality will be ensured by keeping all physical study materials in locked, secure locations and all electronic material in a secure database (REDCap). Except for the demographics form, all data will only be linked with unique identifiers and not with any personally identifiable information. Data uploaded to repositories will have different randomly generated unique identifiers generated during upload to the NIA Aging Research Biobank that are different from those used in REDCap or on study forms.

•Blood collection: Participants will be informed of the risks of venipuncture. Risk for infection is low with the use of sterile technique, and a sterile adhesive bandage will be applied to the blood collection sites. Blood collection will be performed by trained personnel using standard laboratory techniques: disinfecting the site with alcohol, using sterile disposable supplies, and applying pressure with sterile gauze to minimize bleeding and bruising. Syncope is possible, so phlebotomy procedures will be performed using phlebotomy chairs in case legs need to be elevated. Cold packs will be available. Participants will be asked if they have a history of fainting prior to blood collection.

•Muscle biopsy: To minimize risks, muscle biopsies will be performed using standard sterile technique. The skin over the vastus lateralis will be cleaned using povidone-iodine and the area anesthetized using 1% lidocaine without epinephrine. A small incision will be made through the skin and fascia using a single-use sterile scalpel (#11). A sterilized Bergstrom biopsy needle will be inserted into the muscle and approximately 200 mg of muscle will be collected. After the sample has been collected, pressure will be applied to the area for 5 min. The skin will be closed with a sterile adhesive bandage, covered using sterile gauze, and compression wrapped. Each participant will be given verbal and written instructions for biopsy care and extra supplies (e.g., sterile adhesive bandages, alcohol pads) will be provided. The study team will follow up with each participant 2 and 5 days following their biopsies. Sterile supplies (gloves, gauze, scalpel, needles, etc.) will be used and the PIs will train personnel to assist in the procedure. In case a participant becomes lightheaded or feels faint, we will provide food or beverages containing simple sugar (e.g., juice).

12-2a

•Anthropometric measurements: To minimize risks associated with fatigue, these procedures will be performed near a wall or other support and chairs kept nearby in case participants need to rest between measurements. Contact information for guidance counselors will be available.

•Dual-energy x-ray absorptiometry (DEXA): DEXA is considered a low-risk procedure. However, the use of x-rays, even in low amounts, does present some risk associated with ionizing radiation. To minimize these risks, the number of DEXA scans will be kept to a minimum.

•Functional tests, exercise capacity (VO2peak) testing, and HIIT: During screening, we will ensure that participants meet ACSM medical criteria for initiating an exercise program and do not have medical complications that would put them at undue risk (e.g., diabetes, renal disease, uncontrolled hypertension, other cardiovascular disease, etc.). Participants may become faint, nauseous, experience muscle strains or soreness, or fall. To minimize risk associated with physical activity, participants will be counseled on the importance of proper hydration and will regularly be asked about their physical condition and adjustments made as

necessary. Chairs will be available for rest during functional testing, and a researcher will remain close by the participant in case they lose their balance. During VO2peak testing and HIIT, we will utilize cycle-based exercise instead of treadmill exercise to prevent the risk of falls while walking, and participants will be helped on and off the cycle ergometer or exercise bicycle as needed. Participants will perform proper warm-ups and cool downs. We will also monitor heart rate and rating of perceived exertion during exercise. The VO2peak test will be performed in 3-minute stages with predetermined power output (i.e., males start at 50W, females astart at 25W, and power increases by 20W every 3 minutes). If the participants cannot maintain the power output for a given stage, their cycling cadence falls below 50 rpm for two consecutive minutes, or they ask to stop the test, the test will end. In case a participant becomes lightheaded or feels faint, that day's exercise will be stopped and we will provide food or beverages containing simple sugar (e.g., juice).

•Local heat therapy: Core temperature increases are not typically observed with local heating on the thigh and thus is not considered a potential risk for the proposed study. The risk of discomfort will be minimized by determining the lowest optimal heat pad temperature prior to the onset of the heat therapy intervention and that temperature will not be exceeded. Further, participants will be provided with contact information for the PIs should any problems occur during their at-home local heat therapy sessions and will be asked about any discomfort during their weekly check-ins. Heat therapy sessions will occur in the laboratory once weekly and will be monitored by study personnel to ensure that no problems occur during heating sessions. The risk of burns due to personal heating pad use in older adults is not well-documented. However, in a 2021 study (PMID: 33031521), a review of burn injuries in older adults (age 65+) registered in the National Electronic Injury Surveillance System was performed. For adults aged 65-84 years, heating pads were not in the top 5 causes of burn injuries. In the 85+ year-old age group, heating pads were in the top 5 causes of burn injuries. An estimated 204 burns across 100 emergency departments in the United States over a 10-year period were attributable to electric heating pad use over a ten-year period, and all were in women. The risk of burns due to heating pads in this age group is increased by chronic comorbidities that are exclusionary for this study (i.e., diabetes, peripheral vascular disease, chronic kidney disease, neuropathy), minimizing risk. The risk also increases if heating pads are used while sleeping, so we will instruct participants to set a timer and/or alarm to ensure they do not exceed the 90-minute application time, also minimizing risk. Finally, it is estimated that a surface temperature between 42-43°C would produce a burn injury in 12-20 hours (PMID:1885636). Because the difference between skin and muscle temperature is approximately 2°C across a variety of studies, we anticipate that the heating pad setting to produce an intramuscular temperature of 40°C (calculated using estimation equations) will be approximately 42°C. The 90-minute application time is far below the conservative end of the estimated contact time frame to produce a burn injury (12 hours).

12-2a(1) **Is a liability plan offered?**

Yes

✓ No

13: Participant Compensation

13-1 Compensation - Payments to Research Participants

Compensation through TTU funds must comply with [TTU OP 62.25](TTU OP 62.25).

*required

13-1a Are subjects being compensated for their participation in this study?

✓ Yes

*required

13-1a(1) Describe the compensation process.

Paeticipant will be paid via ClinCard upon completion of each step:

Consent/screening ($10)
Familiarization ($50)

Major Measurement Testing (PRE, MID, POST):
•OGTT/Questionnaires ($50)
•Muscle biopsy ($50)
•Physical function and exercise capacity tests ($50)
The major time point testing will be conducted twice for the HIIT group and three times for Local Heat groups.

For each laboratory visit, participants will be compensated $15.
Phase 1:
•LHT: 12 visits = $180
•HIIT: 36 visits = $540

Phase 2:
•LHT: 36 visits = $540

Total:
•LHT: $1230
•HIIT: $900

13-1a(2) Select the payment type used.

✓ Cash

*required

Amount of Payment

13-1a(2)a

Amount will be paid according to the completion of each stage, please see above for details. Participants who are assigned to the LHT groups and complete each visit in its entirety will receive a total of $1230. Participants who are assigned to the HIIT groups and complete each visit in its entirety will receive a total of $900.

*required

13-1a(2)b

When and how will the participant be paid?

ClinCard; payments will be disbursed after each visit

Check

Gift Card (not to be checked if a drawing is involved)

Drawing

Course Credit

Other (includes payment or credit from 3rd party affiliations (i.e., MTurk, SSI, etc.)

Investigator Assurances

Guidance: The Institutional Review Board (IRB) should determine that the risks to subjects are reasonable in relation to anticipated benefits [21 CFR 56.111(a)(2)] and that the consent document contains an adequate description of the study procedures [21 CFR 50.25(a)(1)] as well as the risks [21 CFR 50.25(a)(2)] and benefits [21 CFR 50.25(a)(3)]. It is not uncommon for subjects to be paid for their participation in research, especially in the early phases of investigational drug, biologic or device development. Payment to research subjects for participation in studies is not considered a benefit, it is a recruitment incentive. Financial incentives are often used when health benefits to subjects are remote or non-existent. The amount and schedule of all payments should be presented to the IRB at the time of initial review. The IRB should review both the

amount of payment and the proposed method and timing of disbursement to assure that neither are coercive or present undue influence [21 CFR 50.20].

13-1a(3)

Any credit for payment should accrue as the study progresses and not be contingent upon the subject completing the entire study. Unless it creates undue inconvenience or a coercive practice, payment to subjects who withdraw from the study may be made at the time they would have completed the study (or completed a phase of the study) had they not withdrawn. For example, in a study lasting only a few days, an IRB may find it permissible to allow a single payment date at the end of the study, even to subjects who had withdrawn before that date.

While the entire payment should not be contingent upon completion of the entire study, payment of a small proportion as an incentive for completion of the study is acceptable to FDA, providing that such incentive is not coercive. The IRB should determine that the amount paid as a bonus for completion is reasonable and not so large as to unduly induce subjects to stay in the study when they would otherwise have withdrawn. All information concerning payment, including the amount and schedule of payment(s), should be set forth in the informed consent document.

---

*required

13-1a(3)a **Participant Payment**

✓ I will provide compensation to all participants who complete the consent process and begin the study, regardless of whether they complete the study.

I will **NOT** provide compensation to all participants who complete the consent process and begin the study, regardless of whether they complete the study.

---

*required

TTU OP 62.25

13-1a(3)b

All questions concerning participant payment should be directed to the TTU Payroll & Tax Services office or the researcher's business manager.

✓ I will follow the procedures outlined in TTU OP 62.25 regarding participant payment.

I will **NOT** follow the procedures outlined in TTU OP 62.25 regarding participant payment.

No

Conflict of Interest

Guidance: A conflict of interest refers to a situation in which an employee(s) financial, professional, or other personal considerations may directly or indirectly affect, or have the appearance of affecting, the employee(s) judgment in exercising any duty or responsibility, including the conduct or reporting of research, owed to the institution (TTU OP 10.20 (2)(d)).

14-1

"Significant business or financial interest" means anything of monetary value including, but not limited to, salary or other payments for services (e.g., consulting fees or honorarium; equity interests (e.g., stocks, stock options, or other ownership interests); and intellectual property rights (e.g., patents, copyrights, and royalties from such rights). (TTU OP 74.17).

"...a set of conditions in which an investigator's judgment concerning a primary interest (e.g., subject welfare, integrity of research) could be biased by a secondary interest (e.g., personal or financial gain)." Institutional Review Board Management and Function, Bankert and Amdur, 2006, p. 167.

*required

14-1a **Does anyone on the research team have a potential conflict of interest with the research project?**

Yes

✓ No

15: Investigator Attestations

*required

I, as the PI and co-investigators, understand Texas Tech University's policy concerning research involving human subjects and by checking below, I certify:

- ✓ I have read The Belmont Report "Ethical Principles and Guidelines for the Protection of Human Subjects of Research" and subscribe to the principles it contains.
- ✓ I have read 45 CFR 46, Protection of Human Subjects and subscribe to the regulations it contains.
- ✓ I accept responsibility for the scientific and ethical conduct of this research study and understand my responsibility.
- ✓ I will ensure that all study personnel are appropriately trained and are competent to perform the study.
- ✓ I will obtain prior approval from the Institutional Review Board (IRB) before making any modifications of the previously approved research, including modifications to the informed consent process and document.
- ✓ I will submit a Study Closure Submission upon completion of this study and agree to honor any other commitments.

✓ I will follow TTU procedures and guidelines to protect the health and well-being of research participants, research team members, and liaison(s) during any unforeseen circumstances.

✓ I will immediately report to the IRB any deviations, violations, non-compliance, unanticipated problems, and adverse events related to subjects or others which occurred or possibly occurred as a result of this study.

45 CFR 46.103(a) and TTU IRB Policy 5.4 require Adverse Events, Unanticipated Problems and Deviations from the research protocol to be reported **IMMEDIATELY** to the HRPP office.

Human Research Protection Program
Box 41075
Lubbock, Texas 79409
Phone: (806) 742-2064
Email: hrpp@ttu.edu

16: Additional Information

16-1 **Include any additional points to help clarify the research, if applicable.**

16-1a

16-2 **Enter or attach any additional documentation.**

16-2a

Modification Submission

Modification

*required

Cayuse IRB Modification:

Provide a summary of the changes you are requesting in the text box below. Details of the changes are required in the appropriate sections. The "Compare" feature is a useful tool as you edit each section.

Summary of Changes.

The purpose of this IRB modification is to incorporate hydration assessment and an intramuscular temperature measurement as outlined in the recently awarded grant. Intramuscular temperature provides a more accurate reflection of muscle tissue thermoregulation and is better aligned with the study's focus on skeletal muscle physiology and metabolic health.

*required

Current Research Activities

Check all that apply.

✓ Research procedures have **NOT** started.

Participants are being recruited for this research.

Recruitment has ended.

Participants have been consented to do the research.

Participants have **NOT** been consented to do the research.

Data collection is **ONGOING.**

Data collection is **COMPLETE.**

Analysis of **IDENTIFIABLE** data.

Analysis of **DE-IDENTIFIED** data.

1: Human Subject Research

Answer the following questions to determine if you need IRB review and approval.

1-1 Research

*required

Research is defined by 45 CFR 46.102(l) as:

A systematic investigation, including research development, testing and evaluation, designed to develop or contribute to generalizable knowledge.

Does your proposed study meet the definition of research?

✓ Yes

No

1-2 Human Subjects

*required

Human Subject is defined by 45 CFR 46.102(e) as:

A living individual about whom an investigator (whether professional or student) conducting research

- Obtains or receives private identifiable information or identifiable biospecimens about the individual(s)
- Obtains information or biospecimens through intervention or interaction with the individual(s)

Does your proposed study involve and meet the definition of human subjects?

✓ Yes

No

If you answered YES to all questions in Section 1 complete the rest of the sections.

Complete the following section.

*required

Review Type

2-1

Select the appropriate review type.

Exempt

Expedited

✓ Full Board

The project involves greater than minimal risk to human subjects.

Relying on IRB approval from another institution (IAA)

External Collaboration

2-2

Only select collaboration if there are Non Texas Tech researchers, employees, and/or students working on this research.

Collaboration

International Research

2-3

Research will be conducted outside of the United States of America and/or data will be shared across borders.

International Research

Complete the following section.

Lay Summary

*required

Provide a description of your study in vernacular language.

Avoid the use of technical terms, undefined acronyms, or excessive scientific terminology.

Aging people with prediabetes are at increased risk for frailty, and skeletal muscle architectural factors (which may improve with exercise or heat therapy) could underlie development of frailty and progression to type 2 diabetes. The proposed study will test local heat therapy as a method to improve skeletal muscle architecture (i.e., cross sectional area, capillarization, mitochondria), glucose tolerance, frailty indicators, and exercise adaptation in prediabetic older adults. Data generated will provide evidence supporting a directly translatable, easily implemented therapeutic intervention to ameliorate risk of developing overt type 2 diabetes and frailty in at-risk aging individuals.

Complete the following section. List all researchers involved with recruitment, data collection, and analysis.

NOTICE: HUMAN SUBJECT TRAINING REQUIREMENT

All research study personnel who engage with participants will need to complete human subject training prior to IRB approval and prior to conducting any human research-related activities. The new training requirement will be applied when an existing study is due for renewal, requires modification(s), or is chosen for a post approval monitoring.

Human research-related activities include recruitment of participants, consenting of participants, data collection (anonymous or identifiable), gaining access to identifiable data, and/or conducting analysis of identifiable data. These training requirements will apply to all Principal Investigators, Co-Investigators, and research staff/students. This includes both individuals within the TTU system and external collaborators.

This requirement will go into effect January 1, 2023. For more information visit our webpage.

Effective January 1, 2023, TTU research personnel must complete one of the following trainings every 3 years:

- CITI - TTU Human Subject Research - Required Basic
- TTU Human Subject Training
- External – CITI Human Subject Training

4-1 **Principal Investigator**

TTU OP 74.09

*required

4-1a **Check the appropriate box.**

✓ TTU full-time or tenured faculty member

*required

4-1a(1) **College**

College of Arts and Sciences

*required

4-1a(2) **Department**

Kinesiology & Sport Management

TTU full-time employee with a terminal degree in their discipline

*required

Find and add PI to the submission.

4-1b Name: Hui Ying Luk
Organization: Kinesiology and Sport Mgmt
Address: 2500 Broadway MS3011, Lubbock, TX 79409
Phone: 8068340827
Email: HuiYing.Luk@ttu.edu

4-1a(3)a Attach human subject training.

Luk_CITI.pdf

Primary Contact

4-2

Primary contacts need to be included in section 4.3 as a co-investigator if they are not the principal investigator. The primary contact and principal investigator will receive all notifications and letters.

*required

4-2a **Find and add primary contact to the submission.**

Name: Danielle Levitt-Budnar
Organization: Kinesiology and Sport Mgmt
Address: 2500 Broadway MS3011, Lubbock, TX 79409
Phone: 8068341830
Email: Danielle.Levitt@ttu.edu

Name: Hui Ying Luk
Organization: Kinesiology and Sport Mgmt
Address: 2500 Broadway MS3011, Lubbock, TX 79409
Phone: 8068340827
Email: HuiYing.Luk@ttu.edu

4-3 **Co-Investigators**

Include investigators assisting with instrument development, recruitment, data collection, and data analysis.

*required

4-3a **Select all investigators involved with this research project.**

None

✓ List TTU Faculty or Staff Co-Investigator(s):

*required

Multiple names are allowed.

Name: Fangyuan Zhang
Organization: Mathematics and Statistics
Address: 2500 Broadway MS1042, Lubbock, TX 79409
Phone: 8068342587
Email: fangyuan.zhang@ttu.edu

Name: Heather Vellers
Organization: Kinesiology and Sport Mgmt
Address: 2500 Broadway MS43011, Lubbock, TX 79409-3011
Phone: 8068348554
Email: heather.l.vellers@ttu.edu

4-3a(1) Name: Danielle Levitt-Budnar
Organization: Kinesiology and Sport Mgmt
Address: 2500 Broadway MS3011, Lubbock, TX 79409
Phone: 8068341830
Email: Danielle.Levitt@ttu.edu

Name: Terrell Hilliard
Organization: Kinesiology and Sport Mgmt
Address: 2500 Broadway Ms 3011, Lubbock, TX 79409
Phone: 8068345585
Email: terhilli@ttu.edu

Name: Jaden Hendrix
Organization: Kinesiology and Sport Mgmt
Address: , Lubbock, TX 79409-1035
Phone: 8067423371
Email: jadhendr@ttu.edu

Attach human subject training.

4-3a(1)(a)

Terrell_ Human Subject Research.pdf

Jaden_Human_Subject_JH_7.24.pdf

✓ List TTU Graduate or Undergraduate Co-Investigator(s):

4-3a(2) *Multiple names and selections are allowed.*

✓ Graduate Student(s)
*required

4-3a(2)a

Name: Casey Appell
Organization: Kinesiology and Sport Mgmt
Address: , Lubbock, TX 79409-1035
Phone:
Email: Casey.Appell@ttu.edu

Name: Parisa Foroozan
Organization: Kinesiology and Sport Mgmt
Address: , Lubbock, TX 79409-1035
Phone:
Email: parisa.foroozan@ttu.edu

Name: Masoumeh Rasoli
Organization: Kinesiology and Sport Mgmt
Address: , Lubbock, TX 79409-1035
Phone:
Email: mrasoli@ttu.edu

Attach human subject training.

4-3a(2)a1

*required

4-3a(2)b **The purpose of this study:**

Doctoral Dissertation

Master's Thesis

Research conducted as a class assignment with the intention of publishing results

Class assignment requiring IRB submission with no intention of publishing results

✓ Students are members of the research team

Other

✓ Undergraduate Student(s)

*required

Name: Ashley Williams
Organization: Kinesiology and Sport Mgmt
Address: , Lubbock, TX 79409-1035
Phone:
Email: wil98200@ttu.edu

Name: Matthew DiLeo
Organization: Kinesiology and Sport Mgmt
Address: , Lubbock, TX 79409-1035
Phone:
4-3a(2)c Email: madileo@ttu.edu

Name: Connor Mills
Organization: Kinesiology and Sport Mgmt
Address: , Lubbock, TX 79409-1035
Phone:
Email: mil67126@ttu.edu

Name: Ernan Carrasco
Organization: Kinesiology and Sport Mgmt
Address: , Lubbock, TX 79409-1035
Phone:
Email: erncarra@ttu.edu

Attach human subject training.

4-3a(2)c1

*required

4-3a(2)d **The purpose of this study:**

Undergraduate Research

✓ Students are members of the research team

Research conducted as a class assignment with the intention of publishing results

Class assignment requiring IRB submission with no intention of publishing results

Other

✓ External Researchers: Co-investigators outside of Texas Tech University

*required

List non-TTU investigator(s) engaged in this research. Provide their contact information, title, and place of work.

Robyn Richmond, MD
Assistant Professor of Surgery
Associate Program Director, General Surgery Residency
Trauma, Surgical Critical Care, Acute Care Surgery
Department of Surgery
Texas Tech University Health Sciences Center
robyn.richmond@ttuhsc.edu
972 987 9616

4-3a(3) Leslie Shen, Ph.D.
Associate Dean for Research & Professor of Pathology
School of Medicine
Department of Pathology
Texas Tech University Health Sciences Center
leslie.shen@ttuhsc.edu

Rama Chemitiganti, MD
ECHD Endowed Chair of Medicine
Director, Center of Excellence for Diabetes and Endocrinology
School of Medicine
Department of Internal Medicine

Texas Tech University Health Sciences Center at Permian Basin
rama.chemitiganti@ttuhsc.edu
806-743-3280

*required

4-3a(3)a **Check all that apply.**

Developing instrument

Recruiting participants

Interaction with participants

Working with de-identified data

Working with identifiable data

Assisting with writing manuscript

✓ Other

*required

Include any information that helps to explain their role with the research team.

4-3a(3)a1

Dr. Richmond will provide medical-related oversight/consultation on the muscle biopsy procedures. There will be no direct interaction between Dr. Richmond and the participants.

Dr. Shen will provide consultation on participant recruitment.

Dr. Chemitiganti is the NIH-approved Safety Officer for this study. There will be no direct interaction between Dr. Chemitiganti and the participants.

4-3a(3)b Attach human subject training.

5: Funding

Complete the following section.

5-1 Funding

Funding is used to supplement the cost of the research and/or participant payment.

*required

5-1a **Select the type of funding to be used.**

✓ External (Office of Research Services - ORS)

*required

5-1a(1) **Cayuse SP - Sponsored Project Number (Example: 16-0123)**

23-0477

*required

5-1a(2) **Sponsored Project Title**

Glycemic control and frailty risk in older people at risk for type 2 diabetes: Impact of local heat therapy

*required

5-1a(3) **Find and add the Sponsor's name.**

Name

-

- Name - A to Z
- Name - Z to A

NIH - National Institute on Aging

*required

5-1a(4) **Attach a copy of the sponsored project that was submitted to the funding agency.**

For example: scope of work, abstract, research description.

FINAL_ESI R01_HYLDEL_Specific aims.docx

FINAL_ESI R01_HYLDEL_Research Strategy.docx

Internal (TTU Funding)

Personal Monies

No Funding

5-2 **Future Funding**

If funding is secured, a modification will be required.

5-2a **Enter the potential sponsor(s) name below.**

6: Rationale

Please respond to the following statements to help our IRB reviewers understand your research project.

Research Objective

6-1

*required

Briefly summarize the relevant background information.

Frailty-related medical expenses cost approximately $18 billion annually in the USA. Progressive losses in skeletal muscle (SKM) mass and function, often observed with aging and type 2 diabetes (T2D), contribute to phenotypic frailty characterized by slow gait speed, weakness, weight loss, fatigue, and low physical activity. T2D increases frailty risk by nearly 50% and risk for both conditions increases with age; therefore, preventing progression to T2D in older adults with prediabetes is critical. Additionally, SKM is the largest glucose disposal site in the body and insulin responsiveness, a component of glycemic control, is essential to maintain functional SKM mass. Thus, impaired glycemic control, a pathophysiological change underlying the development of T2D and observed in prediabetes, increases frailty risk. Conversely, improving capillarization and mitochondrial function support increased SKM mass and glycemic control, thereby decreasing T2D and frailty risk. Together, these SKM architectural variables (e.g., cross-sectional area [CSA], capillarization, mitochondria) are attractive targets for interventions such as exercise in prediabetic older people. High-intensity interval training (HIIT) has been used effectively in older adults with SKM benefits similar to those from aerobic and resistance training, and is more time-efficient. However, prediabetic older people may be exercise-intolerant or -resistant, underscoring the need for alternative therapies in place of (or in addition to) exercise. Emerging evidence supports repeated heat therapy as an alternative method to improve glycemic control and SKM architecture, and such adaptations may also improve muscle growth responses to subsequent exercise. While whole-body heat therapy is widely studied, local heat therapy (e.g., heat pad) is more practical and likely provides similar health benefits. Whether local heat therapy would have similar benefits to HIIT or whether heat pre-conditioning would improve adaptations to subsequent HIIT in prediabetic older adults is unknown.

6-2 **Importance of Research**

*required

Explain the scientific importance of the knowledge to be obtained as a result of this research.

Exercise is a first-line intervention for improving glycemic control and decreasing frailty risk due largely to adaptations in SKM. While exercise has a host of whole-body benefits, older individuals in general and those at risk for T2D (i.e., prediabetic) in particular may be exercise-resistant or -intolerant. Therefore, there is an urgent need for **efficacious and practical interventions** to improve muscle health in aging people to decrease their risk of developing T2D and frailty. The proposed project is innovative because we will:

- Use local heat therapy as an *easily implemented intervention* to improve the fundamental aspects of muscle health in aging prediabetic people, a novel application of this widely-used therapy
- Compare the efficacy of our proposed intervention alone and as a means of *preconditioning muscle* for improved exercise adaptations

Finally, this project will provide essential fundamental evidence to lay the groundwork for a subsequent clinical trial that examines the use of this technique in aging people with other SKM-related comorbidities. This work has the potential to **shift clinical practice** by providing direct evidence that supports a low-cost, practical intervention to improve metabolic health in at-risk individuals.

6-3 **Research Questions/Specific Aims**

*required

List the research question(s)/specific aims to be addressed.

The overarching hypothesis of this study is that local heat therapy improves muscle architecture, glycemic control, and subsequent exercise adaptations, and decreases frailty risk in prediabetic older adults, with TRPV1 as an underlying mechanism [mechanistic experiments will be in Aim 3, not part of this IRB application]. We will test this hypothesis through the following specific aims:

Aim 1: Test the hypothesis that local heat therapy improves muscle architecture, glucose tolerance, and frailty indicators similarly to HIIT in older prediabetic people.

Aim 2: Test the hypothesis that local heat therapy pre-conditioning improves the skeletal muscle response to HIIT in older prediabetic people.

6-5 Citations

Enter or attach the references cited .

The references below are from our Specific Aims and Research Strategy documents. Although they are not cited in the project summary, importance, and aim above, they are cited in the funding application.

ESI R01_Bibliography & References Cited.docx

Please respond to the following statements/questions to help our IRB reviewers know more about the participants in this study.

7-1 **Relationship with Participants**

*required

7-1a **Is there the potential for any relationship between the researcher(s) and the proposed target population?**

(Note: if recruiting in the Lubbock (which includes TTU) community please click yes as students and employees are possible)

✓ Yes

*required

7-1a(1) **Explain**

Recruiting older adults with prediabetes in Lubbock; it is possible that other TTU employees could be recruited.

No

Coercion or Undue Influence

Guidance for IRB and Investigators

Coercion - Coercion entails influencing an individual's decision about whether or not to

7-2 do something by using explicit or implied threats (loss of good standing in a job, poor grades, etc.). This area also includes the perception of students as to any potential backlash for failing to take part in an instructor's research.

Undue Influence - This is defined as an offer of an excessive, unwarranted, inappropriate, or improper reward or other overture in order to obtain compliance (used in association with recruiting research participants).

*required

7-2a **Does this research involve participants who could be coerced or unduly influenced to participate or may feel that there is perceived coercion or undue influence (i.e., students, employees, prisoners, colleagues, friends/acquaintances, family members, etc.)?**

✔ Yes

*required

Describe the plan to mitigate (perceived) coercion or undue influence to participants.

7-2a(1) One example of mitigating coercion/undue influence would be to add a sentence to the consent form that clearly states their decision to participate, not participate, or withdraw in the research will not impact their employment status or academic standing.

We will emphasize to participants that their enrollment and continued participation is completely up to them. They can withdraw from the study at any time for any reason without consequences. The PIs of this study do not currently have any influence over the employment of individuals who would qualify for this study. Any future non-traditional student who might qualify will be assured that their grade(s) will noty be impacted, positively or negatively, by their participation or withdrawal. Colleagues in KSM who might qualify and enroll will be assured that our working relationships will not be impacted, positively or negatively, by their participation or withdrawal. The PIs are committed to ensuring that participation and/or withdrawal of KSM colleagues who might choose to participate and/or withdrawal does not impact our decisions for any future faculty votes, recommendations, etc. Finally, we will emphasize the importance of confiidentiality with our research team, **especially** when a participant might be a TTU (including KSM) employee.

No

7-3 Target Population

*required

7-3a **Describe the population of human subjects to be recruited and why this research specifically targets this group?**

In this study, participants with elevated blood glucose, prediabetes, or are at risk for developing diabetes; are sedentary will be recruited.

This population is targeted because the muscular pathophysiology of T2D (i.e., insulin resistance [IR], decreased skeletal muscle [SKM] mass and function) contributes to phenotypic frailty including slow gait speed, weakness, unintentional weight loss, fatigue, and low physical activity. Approximately 7-15% of community-dwelling older adults meet criteria for frailty, and 25-40% of these individuals are prediabetic. Moreover, a recent meta-analysis of community-based studies found that baseline T2D is associated with nearly 50% increased risk of frailty development.

Although exercise training is a key technique to manage metabolic disease and decrease T2D risk, in older adults at risk for T2D, compromised oxygen delivery and mitochondrial dysfunction result in exercise intolerance, a potential contributor to low exercise adherence. Thus, it is critical to find alternative means to manage metabolic disease risk. Heat therapy is one possible alternative. Heat therapy adaptations could improve exercise tolerance by preconditioning the microenvironment of skeletal muscle, likely enhancing exercise adherence and improving subsequent exercise adaptations.

*required

7-3b **Does your target population require specific characteristics?**

Yes, my target population **DOES** require specific characteristics.

✓ No, my target population **DOES NOT** require specific characteristics.

*required

7-3c **Age Range**

Check all that apply.

✓ Adults

*required

7-3c(1) **Enter the age range.**

≥60

Children

*required

7-3d **Enter the number of participants.**

27 men and 27 women

7-4 **Inclusion/Exclusion Criteria**

Enter or attach all criteria used to include or exclude participants for this research.

Individuals with elevated blood glucose, prediabetes, or are at risk for developing diabetes; are sedentary

Inclusion:
•Sedentary (structured exercise <30 minutes, 3x/week)
•Meet criteria for prediabetes (fasting blood glucose 100-125 mg/dl and/or hemoglobin A1c 5.7-6.4%)
•Age ≥ 60 years
• Body weight is at least 110 pounds
•Consume <8 (women) or <15 (men) alcohol-containing beverages per week
•Do not use nicotine or cannabis

•Not taking any medications that could interfere with responses to the interventions (e.g., corticosteroids, opiates, benzodiazepines, tricyclic antidepressants, beta blockers, sulfonylureas, insulin, metformin, anticoagulants, barbiturates, insulin sensitizers, fibrates [PPAR gamma agonist], immunosuppressants)

Exclusion:
•History of peripheral neuropathies
•Currently taking prescription blood thinners
7-4a •Medical complications that could would contraindicate participation in the high intensity interval training (HIIT) intervention including: orthopedic complications that would limit your ability to perform cycling exercise, significant cardiovascular impairments (e.g., history of arrhythmias, severe uncontrolled hypertension, etc.), diagnosed metabolic disease (e.g., diabetes), renal disease, sickle cell anemia, or cancer in remission for <6 months.
•Known history of slow wound healing
•Excessive subcutaneous fat over the vastus lateralis (greater than 1.5")
• Have experienced symptoms suggestive of cardiovascular, respiratory, metabolic, or renal diseases including discomfort, pressure, or pain in your chest, neck, jaw, arms, calves, or other areas potentially related to ischemia; shortness of breath at rest or with mild exertion; dizziness or fainting (syncope); difficulty breathing while lying flat (orthopnea) or sudden nighttime breathing difficulties (paroxysmal nocturnal dyspnea); palpitations or rapid heartbeat (tachycardia); pain or cramping in your legs during physical activity (intermittent claudication); a known heart murmur; swelling in your ankles (edema); unusual fatigue or shortness of breath during routine activities or at rest.
• Currently pregnant
• Lidocaine allergy
• Latex allergy

We will screen for these criteria using the medical history form (attached). After inquiring with the participant, "Yes" to the following questions would result in exclusion from the study: 1, 3-7, 9-12, 15-17 (unless acute illness), 19 (lidocaine only), 20 and 23. Answers to other questions will be compared against inclusion/exclusion criteria (i.e., injuries, illnesses, chronic conditions, other reason that they should not exercise, etc.) to determine whether the participant meets all inclusion and no exclusion criteria. A key for research staff is included as a separate page in the form (beginning in Column Z on the spreadsheet).

7-4a(1) Inclusion/Exclusion Screening Form (if applicable)

R4_Feb 3_IM_HEAT study_Medical History Questionnaire.xlsx

7-5 **End of Subject Participation**

*required

7-5a **Enter specific criteria used by the researchers to prematurely end a subject's participation in the study.**

Sometimes a PI will decide to remove a participant from a research study. List any reasons you would anticipate that may require this removal.

This is not a participant deciding they would like to stop/withdraw from the research. This is a researcher ending their participation. For example: unforeseen risk to the participants, risks to the other participants/research team members, non-adherence, disruptive behavior.

The researchers will end participants' involvement in the study early if they do not follow the study's instructions, which include:

- Adopting a new lifestyle
- Starting a new exercise routine and/or diet program
- Taking any new supplements
- New medical conditions or complications that would place participants at increased risk
- Low adherence to interventions (<80% compliance)

7-6 **Investigator Assurances**

*required

7-6a **Data Storage and Retention**

✓ I will provide participants with information about data storage and retention if they withdraw from the study, when contacted by subjects per contact information provided in the consent form.

I will **NOT** provide participants with information about data storage and retention if they withdraw from the study, when contacted by subjects per contact information provided in the consent form.

*required

7-6b Participant Withdraw

✓ I will provide participants with information about how to withdraw from the study through the provided consent form.

I will **NOT** provide participants with information about how to withdraw from the study through the provided consent form.

Please respond to the following statements/questions.

8-1 **Recruitment Procedures**

*required

Describe the process of how participants will be recruited and provide the sequence of these events.

This section needs to include the details of each step for recruitment up to the point of consent. This includes pre-screening procedures.

All planned recruitment efforts and scripts will be approved by the Texas Tech University IRB. We will begin recruitment through the University Medical Center in partnership with Texas Tech University Health Sciences Center physicians who see patients from the community that may qualify. Email addresses and/or phone numbers will be obtained by searching for internal medicine, family medicine, geriatrics, and endocrinology clinics. Specially, fliers will be placed in the clinic and provided to physicians to distribute to potential participants.

8-1a Electronic advertisements will be posted on listservs (e.g., TechAnnounce, Obesity Research Institute listserv) and on social media (e.g., Facebook). Social media announcements will specify targeting older adults (60+) in the Lubbock, TX area. Additionally, after site-specific approval, flyers will be distributed around the community to frequently visited locations such as grocery stores, pharmacies, and community centers.

Drs. Luk and Levitt will be responsible for executing recruitment efforts, collecting contact information for potential participants, conducting initial phone screenings, and following up with potential participants to schedule them for their informed consent and screening visit. Once a clinical coordinator is hired, these responsibilities will primarily shift to that person. The initial phone screening is to ensure participant is at least 60 years old, sedentary, and their physician has informed them they have elevated blood glucose, prediabetes, or are at risk for developing diabetes.

8-2 **Recruitment Methods**

[Templates]

*required

8-2a **Select all methods of recruitment.**

Check all that apply.

No Recruitment

✓ Oral Script

*required

Enter or attach the Oral Script(s).

8-2a(2)

If more than one oral script is entered or attached, label each script separately.

Script for UMC and TTUHSC phone recruitment is attached

8-2a(2)a If you attach a document, state *See Attachment* in the text box above.

R4_Feb 4_Script_UMC and TTUHSC recruitment.docx

✓ Tech Announce

*required

8-2a(3) **Enter or attach TechAnnounce ad.**

See attached

8-2a(3)a If you attach a document, state *See Attachment* in the text box above.

R5_March 5_IM_Tech Announce Ad - HEAT study (2) (3).docx

✓ Recruiting Letter(s) or Email(s)

*required

8-2a(4) **Explain how email addresses or physical addresses of potential participants are obtained.**

We will search for any email addresses for UMC or TTUHSC physicians' offices.

*required

8-2a(5) **Enter or attach all recruiting emails or letters.**

If more than one email is entered or attached, label each separately.

Script attached

8-2a(5)a If you attach a document, state *See Attachment* in the text box above.

R4_Feb 4_Script_UMC and TTUHSC recruitment.docx

Information Sheet

✓ Advertisement(s)

*required

8-2a(8) **Check all that apply.**

✓ Print Advertisement (eg. newspaper, magazine, fliers)

Website

✓ Social Media

*required

8-2a(9) **Enter or attach the advertisement.**

If more than one advertisement is entered, label each separately.

The attached flyer will be used for print and social media advertising.

8-2a(9)a If you attach a document, state *See Attachment* in the text box above.

R5_Feb 4_IM_Flyer- HEAT study (3).doc

Subject/Participant Pools

Third Party Recruitment

Other

8-2b **Investigator Assurances**

*required

8-2b(1) **Permission to Recruit Participants**

✓ I will acquire proper permission prior to recruiting participants.

I will **NOT** acquire proper permission prior to recruiting participants.

8-3 **Permission to Conduct Research**

*required

8-3a **Is this research study being conducted in a setting that requires prior permission from an entity to conduct research on their premises (i.e., school system, business, government entity, etc.)?**

Yes

✓ No

Visit the HRPP website for instructions and templates on the Consent Process, Assent Process with Minors, Short Form Consent Process, and Waivers of Consent.

Consent forms can be destroyed three years after the completion of the research. 45 CFR 46.115(7)(b).

9-1 **Consent**

*required

9-1a **Consent Process & Forms**

Check all that apply.

✓ Consent (Adult)

*required

9-1a(1) **Check all that apply.**

✓ Participant

Legal Authorized Representative Consent (LAR)

Parent/Legal Guardian

*required

Describe the process for obtaining consent. Include where and when the consent process takes place and who will be obtaining consent.

9-1a(2)

Drs. Luk or Levitt will provide a brief overview of the study using the information sheet and then seek informed consent from all potential participants in their offices in the KSM building to ensure privacy. All participants will be provided with copies of the information sheet and informed consent sheet. They will also be provided with a complete verbal explanation regarding the reasons for conducting the study, the procedures, risks, and benefits of the study, and any questions will be answered. At the end of the consent process, participants will be asked the following questions to establish capacity to consent:

Do you understand why this study is being done?
If you decide to participate in the study, what are some of the things you will be asked to do?
Describe some of the risks or discomforts you may experience if you participate in this study.
Will this study help you?
Do you have to be in this study?
What will happen if you decide not to be in the study?
Who should you contact if you have questions or experience a problem while in the study?

Correctly answering these questions will demonstrate an understanding of the study and determine the capacity to provide consent. Adults unable to demonstrate the capacity to consent will not be enrolled in the study.

*required

9-1a(3)

Enter or attach the consent form.

If more than one consent form is entered or attached, label each separately.

See attachment

If you attach a document, state *See Attachment* in the text box above.

9-1a(3)a

DXA Acknowledgement Form (1) (1).pdf

R5_March 5_Informed Consent - HEAT study_Laymen term (4).docx

*required

9-1a(4)

Will a Short Form Consent be used?

Only used in complex studies when the PI cannot be sure that a signed written consent is understood well enough to indicate a valid consent process.

Yes

✓ No

Assent (Minors & Individuals with Impaired Decision Making Skills that cannot consent for themselves)

Texas Tech Neuroimaging Institute (TTNI) Consent Form

No consent will be obtained.

9-2 **Waivers**

A consent form must still be included in the documentation.

9-2a **Check all that apply.**

Request for a Waiver of Written Consent

Request for a Waiver or Alteration of the Elements of Consent

FERPA Authorization

IRB approval or determination of exemption of the research project does not constitute institutional permission to access the requested records.

9-3 **Guidance:** FERPA applies to personally identifiable information in educational records. This includes items such as the student's name, names of family members, addresses, personal identifiers such as social security numbers, and personal characteristics or

other information that make the student's identity easily traceable.

Educational records are all records that contain information directly related to a student and are maintained by an educational agency or institution, or by a party acting on its behalf. A record means any information recorded in any way, including handwriting, print, tape, film, microfilm, microfiche, and digital images.

9-3a **Will you be using student records protected under FERPA?**

Yes

✓ No

HIPAA Authorization or Waiver

9-4

Indicate whether you are obtaining Participant Authorization or requesting a Full or Partial Waiver of HIPAA Authorization.

9-4a **Select the appropriate authorization.**

Participant Authorization of Protected Health Information

Full or Partial Waiver of Protected Health Information

Complete the following section.

Data Collection

10-1 IRB members find it very helpful to have copies of all instruments. Reading the instrument(s) is essential to the board's assessment of participant risk and burden and the adequacy of the study description in the informed consent. Occasionally, however, investigators do not include all or full copies of instruments on the grounds that the instrument is copyrighted and in the belief that making copies to submit with an IRB application is a violation of copyright law. However, instrument developers and publishers should have a reasonable expectation that instrument users must obtain IRB approval of their research and that approval requires review of all study materials. Further, IRB members are well aware of their own ethical responsibility to honor copyright and purchase contracts by investigators and to refrain from use of copyrighted material for purposes other than those related to their IRB obligations. If records are being withheld pursuant to a Non-Disclosure Agreement (NDA), you (the investigator) are expected to provide the relevant portion of the NDA or any similar documentation requiring the records be withheld specifically from the IRB. If the NDA or license precludes sharing with the IRB, it is the PI's responsibility to get approval from the company or licensor to share the instrument(s) with TTU's IRB for review.

*required

Describe the sequence of events and timeline the participants will experience.

A randomized controlled intervention design will be used. The study consists of 2 phases, each 12-weeks in duration.

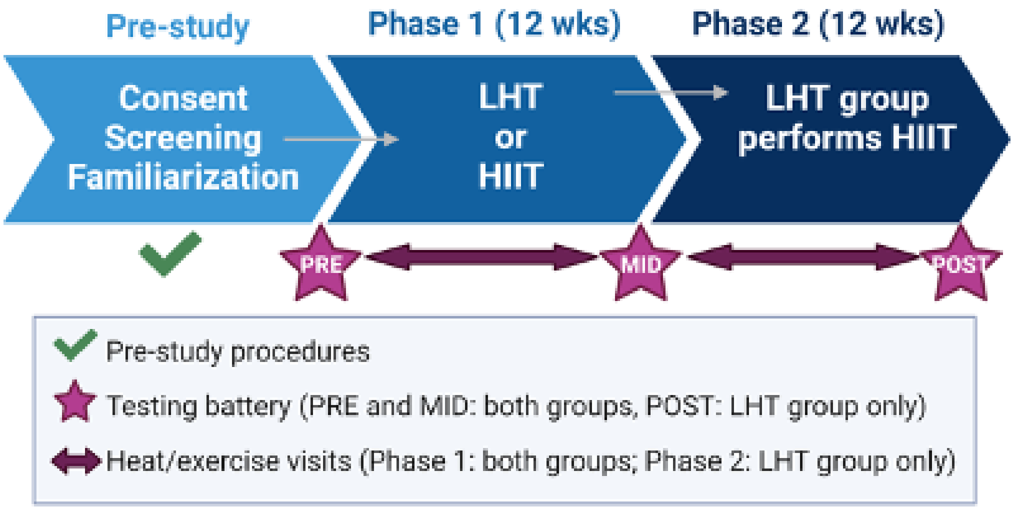

Visit 1: Consent and screening: (~1h)
•Participant will need to fast (i.e., no food or beverages other than water; plain water allowed and encouraged) for 12h before consent and screening.
•After an explanation of study requirements, participant will review and sign the informed consent form.
•We will collect a drop of participant blood using finger prick to determine their fasting blood glucose and HbA1c concentrations to make sure they have met the inclusion criteria.
•We will put a small drop of jelly on participant's thigh and use an ultrasound machine to image the participant's vastus lateralis muscle. This will help us make sure that the lidocaine can reach the muscle (i.e., subcutaneous fat over the muscle no greater than 1.5").

PRE Testing (Before Phase 1 Begin): All major measurements for the PRE Testing will be completed over 2 separate days within a week. These major measurements will also be conducted at MID (end of Phase 1), and POST (end of Phase 2). However, the sequence of these major measurements will be different.

Preparing for Visit 3-5:
1. Overnight fast for 12h (only water allowed)
2. No alcohol for 24h
3. No medications or supplements from this list for 48h:
•Tylenol (acetaminophen)
•Non-steroidal anti-inflammatory drugs (e.g., Ibuprofen)

Visit 2 (Day 1 of the PRE Testing): (~ 5h)
1. Hydration assessment: Participant will provide a urine sample so we can assess the hydration status (urine specific gravity, a measure of hydration, must be <1.020). Also, blood sample will be collected for plasma osmolality measurement. In addition, thirst sensation will be assessed using a thirst scale and urine color will be recorded. If participant is not sufficiently hydrated, then they will be given at least 8 oz of water and re-test their hydration after 15 minutes. After 15 minutes, urine sample and thirst sensation will be measured.

2.Anthropometric Measurements:

•We will measure your height, weight, waist-hip ratio, and a body composition scan will be performed using dual X-ray absorptiometry (DXA) and .

3. Blood pressure:
• We will measure your blood pressure.

4.Muscle Biopsy: We will take muscle sample (~200 mg) from your outer thigh. This procedure will last about 30 minutes.

5.Oral Glucose Tolerance test (OGTT):
• Participants will consume a beverage containing 75 g glucose (TRUTOL, ThermoFisher Scientific).
•Before and at 30 min, 1 hr, and 2 hrs after beverage consumption, venous blood will be collected into vacuum tubes using the standard venipuncture technique.
6. Questionnaires: During the OGTT, you will be familiarized and complete the following set of questionnaires. Questionnaires 2-6 will also be filled out weekly during the study.
1.Unintentional weight loss (self-report, ≥10 lbs or ≥5% of body mass in the past year or between major study time points)
2.Fatigue (SF-36 energy subscale)
3.Physical activity (International Physical Activity Questionnaire)
4.24h Diet Recall (ASA24: Healthy Eating Index 1-100 scale).
5.Sleep quality and well-being (7-point likert scale)
6.Alcohol use (Timeline Follow Back)

Visit 3 (Day 2 of the PRE Testing) (~1.5h)
1. **Hydration assessment:** Participant will provide a urine sample so we can assess the hydration status (urine specific gravity, a measure of hydration, must be <1.020). In addition, thirst sensation will be assessed using a thirst scale and urine color will be recorded. If participant is not sufficiently hydrated, then they will be given at least 8 oz of water and re-test their hydration after 15 minutes. After 15 minutes, urine sample and thirst sensation will be measured.
2. Body weight: Body mass will be measured
3.Physical Function (part of Phenotypic Frailty Assessment) Testing:
•Perform the gait speed test (4-m walk test), 30-second Sit-to-stand test, and handgrip strength test on both hands.
4.Exercise Capacity Test:
•Perform an exercise capacity (VO2peak) test using a cycle ergometer.

Phase 1:

For the Local Heat Therapy Groups
If participants are assigned to one of the local heat therapy(LHT) groups (Sham (CON) and LHT), they will be sent home with 2 heating pads and apply local heat to their thigh 6 days for 12 weeks (5 sessions at home and 1 session at the Lab). Participants assigned to the heating group will be advised to set their heat pads to the temperature previosuly determined to achive an intramuscular temperature of up to 40 C; participants assigned to the sham (CON) group will be advised to set their heat pads to a lower tempereature (e.g., 36 C) to

maintain a neutral intramuscualr temperature.

Visit 4: Establish heat pad temperature: (1st week of the 12-week LHT intervention)

• During the familiarization visit, we will establish the specific heat pad temperature needed to elevate each participant's estimated intramuscular temperature to a maximum of about 40°C. Intramuscular temperature sensors will be used to calibrate the heat pad to approximately 104°F (40°C) over a 90-minute period. For participants in the LHT group, these measurements will determine the optimal heat pad temperature. To reach the target intramuscular temperature of 40°C, the heat pad temperature will be increased by 2–3°C every 5 minutes. If the intramuscular temperature exceeds 40°C, the heat pad temperature will be decreased to maintain the intramuscular temperature at 40°C. After the 90-minute heating session, sensors will remain in the muscle for an additional 60 minutes to monitor temperature changes.

• In addition, a temperature probe will be placed on the skin, 5 cm below the incision site for the intramuscular probe, to record the skin temperature.

To measure the intramuscular temperature using intramuscular temperature probe, we will utilize the following published method (Richey et al., 2024). The PIs will receive training from consultant Dr. Steven Romero to ensure accurate technique and proper care throughout the following procedure.

1. The opposite leg from the biopsy site will be used to measure intramuscular temperature.
2. Apply lidocaine under the skin and into the muscle.
3. Put a sterile catheter or sterile 18G needle into the midpoint of the vastus lateralis, then remove the needle from the catheter.
4. Insert the thermocouple (IT-18; Physitemp: Type T thermocouple; temperature range: -100 to +150 C/-148F to +300F) through the needle or catheter.
5. Position the thermocouple approximately 1 cm deep into the muscle.
6. Carefully retract the needle or catheter to ensure the thermocouple is correctly positioned within the muscle.
7. Cover the area with skin-safe adhesive tape (Tegaderm Film) to ensure everything stays in place.
8. Apply heat pad on the thigh for 90 min.
9. Then remove for up to 60 additional minutes (or until the intramuscular temperature returns to baseline, whichever is first).
10. Muscle temperature and body temperature (forehead scanning) will be monitored throughout.
11. Blood pressure will be measured every 10 minutes throughout the 90-min heat pad application.
12. A bandage will be placed on the site of the incision.

o If the intramuscular temperature is below 40°C, we will increase the heat pad temperature in an increment of 0.5°C until the intramuscular temperature reaches 40°C.
o If the intramuscular temperature is above 40°C, we will decrease the heat pad temperature by an increment of 0.5°C until the intramuscular temperature reaches 40°C.
5. Once the intramuscular temperature reaches 40°C, the heat pad temperature will be set and temperature (skin and intramuscular temperatures) will be monitored for the remainder of the session.

10-1a o *Note:* We are aiming for an intramuscular temperature of 40°C. We anticipate that this will be well-tolerated because 1) core temperature remains stable during local heating on the thigh and 2) intramuscular temperatures of 40°C (vastus lateralis) have been documented in human participants without adverse effects. However, if a participant is unable to tolerate this, we will reduce the initial target intramuscular temperature as needed to a minimum of 38°C based on intramuscular adaptations previously documented in the literature.

To measure skin temperature:
1. One temperature sensor will be placed on the skin, 5cm below the incision site of the intramuscular probe.
2. One insulation disk will be placed on the temperature sensor and secured with adhesive tape.

To estimate the intramuscular temperature using skin temperature, we will utilize the following validated estimation equations (Flouris et al. 2014). These equations has been previously used to predict intramuscular temperature with heat and exercise (Maunder et al. 2024; Soo et al., 2019).

Equations specific to estimating intramuscular temperature for the vastus lateralis:
$Tmrest = iDISK \cdot 0.597 - iDISKlag2 \cdot 0.439 + iDISKlag3 \cdot 0.554 - iDISKlag4 \cdot 0.709 + 14.767$
$Tmex = iDISK \cdot 0.599 - iDISKlag4 \cdot 0.311 + 15.63$
$Tmrec = iDISK \cdot 0.657 - iDISKlag4 \cdot 0.538 + 13.283$

where:
Tmrest = predicted intramuscular temperature of the vastus lateralis at rest
Tmex = predicted intramuscular temperature of the vastus lateralis during exercise
Tmrec = predicted intramuscular temperature of the vastus lateralis during recovery
iDISK = temperature reading of the iDISK
iDISKlag”*n”* = temperature reading of the iDISK at “*n*” minutes prior (i.e., lag)

Steps:
1. Temperature sensors will be placed on the skin 5 cm below the incision site for intramuscular probe.
2. An insulation disk will be placed directly on top of the temperature sensors and secured with adhesive dressing.
4. Skin temperature will be monitored continuously for 90 min and estimated intramuscular temperature will be calculated every 5 min using the above equation.

<u>At home Local Heat Therapy Sessions (5d/wk):</u>

• Participants will be encouraged to empty their bladder before the start of each session to minimize the number of times they have to remove the heat pads during a session. For any reason they have to remove the heat pad, they will record it.

•For each session, participant will place the heating pads to completely surround each thigh with a snug fit. The heating pads will be secured in place using Velcro and set to the predetermined temperature continuously for 90 minutes.

• Participants will record the following for each session:

1. Start time
2. End time
3. Number of times you need to remove the heat pad during a session
4. The duration for which it is removed
5. Discomfort

•There is no restriction on the location (at home or in the office), time of day, and seated activity during the session (e.g., watching TV, reading a book, etc.).

In-Lab Local Heat Therapy Sessions (1d/wk): Visit 5-15 (~2h each) (1d/wk for the remaining 11 weeks of the 12-week LHT intervention)
•During the weekly lab visit (1d/wk), comfort will be monitored, and we will collect the logs of participants' at-home sessions to confirm compliance.
• Blood pressure will be measured every 10 minutes while the heating pad is applied.
• The following will also be assessed each week: sleep quality and activity (Hooper questionnaire and International Physical Activity Questionnaire [IPAQ]), alcohol use (Timeline Follow-back), 24-hr diet recall (ASA24), and anthropometrics (height, body mass, waist circumference, and hip circumference)
• At weeks 6 and 12 of Phase 1, we will measure both intramuscular and skin temperatures during the in-lab heat pad therapy session.

For the High_Intensity Training (HITT) Groups

In Lab HIIT Session: Visit 4-39 (Total of 36 visits) (~1h each)
•Participants assigned to the HIIT group will arrive at the laboratory 3 d/wk for 12 weeks. They will be fitted with a heart rate monitor (Polar) and skin temperature sensors (thigh) and will perform a 3-minute cycling warm-up at 30W before beginning the HIIT protocol.
•On the first day of each training week, the following will also be assessed each week: sleep quality and activity (Hooper questionnaire and International Physical Activity Questionnaire [IPAQ]), alcohol use (Timeline Follow-back), 24-hr diet recall (ASA24), and anthropometrics (height, body mass, waist circumference, and hip circumference)
• At weeks 1, 6, and 12 of Phase 1, we will measure skin temperatures during the HIIT session.

MID Testing (End of Phase 1):
The final LHT or HIIT session must be completed 2 days before MID testing.
All major measurements for the MID Testing is identical to PRE Testing including the order of measurements. Detail protocols, please refer to PRE Testing.

Preparing for the following Visits:
1. Overnight fast for 12h (only water allowed)
2. No alcohol for 24h
3. No medications or supplements from this list for 48h:
•Tylenol (acetaminophen)
•Non-steroidal anti-inflammatory drugs (e.g., Ibuprofen)

Visit 16 (LHT)/ Visit 40 (HIIT): Day 1 MID Testing (~5h)
1. Hydration assessment
2. Anthropometric measurements
3. Blood Pressure
4. Muscle Biopsy
5. OGTT
6. Questionnaires

Visit 17 (LHT)/ Visit 41 (HIIT): Day 2 MID Testing (~ 1.5h)
1. Hydration assessment
2. Body weight
3. Physical functional tests
4. Exercise capacity test

Phase 2:
Visit 18 - 53 - Phase 2 (Sham (CON) and LHT groups): Participants assigned to the Sham (CON) and LHT groups will continue to Phase 2. In Phase 2, they will complete HIIT 3d/wk for 12 weeks. For detailed protocols, please refer to above (HIIT group).

POST Testing (End of Phase 2):
All major measurements and order of measurements for the POST Testing is identical to PRE and MID Testing. Detail protocols, please refer to PRE Testing.

The final LHT session must be completed 2 days before POST testing.
All major measurements for the MID Testing is identical to PRE Testing including the order of measurements. Detail protocols, please refer to PRE Testing.

Preparing for the following Visits:
1. Overnight fast for 12h (only water allowed)
2. No alcohol for 24h
3. No medications or supplements from this list for 48h:
•Tylenol (acetaminophen)
•Non-steroidal anti-inflammatory drugs (e.g., Ibuprofen)

Visit 54 (LHT): Day 1 POST Testing (~5h)
1. Hydration assessment
2. Anthropometric measurements
3. Blood Pressure
4. Muscle Biopsy
5. OGTT
6. Questionnaires

Visit 55 (LHT): Day 2 POST Testing (~ 1.5h)
1. Hydration assessment
2. Body weight
3. Physical functional tests

4. Exercise capacity test

*For Sham (CON) and LHT groups, participants will have a total of **55 Lab visits** with a total time spent for about **81.5h (Lab)** plus **90h (at home heat pad therapy)**.*

*For the HIIT group, participants will have a total of **41 Lab visits** with a total time spent for about **50h**.*

*required

10-1a(1) **What is the total time commitment for the participant?**

For CON (Sham, considered under the "LHT groups" in the consent form) or true LHT group, the duration will be approximately 7 months (testing: 1 month total; Phase 1: 3 months, Phase 2: 3 months) with a total of about 81.5h (lab) plus 90h (at home heat pad therapy). For HIIT group, the participation duration will last for approximately 4 months (testing: 3 wks total; Phase 1: 3 months) with a total of about 50h.

*required

10-1b **Select procedure(s) to be used.**

Check all that apply.

✓ Surveys

*required

10-1b(1) **Check all that apply.**

Online

✓ On-Site

Mailing

Other

*required

10-1b(2) **How much participant time is involved?**

The time commitment for surveys is included in the overall study visit time.

*required

Enter or attach the survey(s) questions or a list of survey items.

If multiple surveys are used, label them separately.

Hooper well-being survey questions (completed weekly; scored on 1-7 scale,
low (fatigue, stress, soreness) or good (sleep quality) and 7 indicates high (fa
soreness) or bad (sleep quality):

1. Please rate your fatigue level over the past 24 hours.
2. Please rate your level of stress over the past 24 hours.
3. Please rate your level of soreness over the past 24 hours.
4. Please rate the quality of sleep you had last night.

10-1b(3)

Automated Self-Administered 24-hour Dietary Assessment Tool (ASA-24; cor
Registration for use of the ASA-24 will be completed upon Notice of Award ar
monies. A link to a demonstration website for the ASA-24 is here: https://asa2
(2024 version)

Unintentional weight loss (at PRE, MID, and POST testing): In the past year [
12 weeks [MID and POST], have you unintentionally lost body weight? If so, h

Other surveys: see attachments.
1) Medical history (screening visit)
2) International Physical Activity Questionnaire (IPAQ)-SF (weekly)
3) Timeline Follow Back (TLFB, weekly)
4) 36-item Short Form Health Survey (SF-36) energy subscale (weekly; only i
and 31;
scored per https://www.rand.org/health-care/surveys_tools/mos/36-item-short

If you attach a document, state *See Attachment* in the text box above.

10-1b(3)a

IPAQ-SF.pdf

TLFB_Alcoholinstructions.pdf

TLFB_ALoverview.docx

SF-36.pdf

Thirst Scale.docx

urine color chart.jpg

R1_11.19_IM_Detail the intervention procedures.docx

*required

10-1b(4) **Describe the process for the distribution and return of surveys.**

Surveys questions will be uploaded into Research Electronic Data Capture (REDCap), a free, secure, web-based application designed to support data capture. Researchers will assist particiants to electronically complete surveys during their laboratory visits.

Interviews

Focus Groups

Observation

Non-Invasive Procedures (MRI, X-Rays, etc.)
Invasive Medical Procedures (Blood draws, skin pricks, biopsies, etc.)

✔ Intervention

*required

10-1b(25) **How much participant time is involved?**

LHT groups ~81.5h (lab) plus 90h (at home heat therapy) and HIIT group ~50h in the laboratory

*required

10-1b(26) **Where will the intervention(s) take place?**

At home (Sham (CON) and LHT groups) and KSM building (all groups)

*required

10-1b(27) **Describe in detail the intervention procedures.**

Attach any documentation if applicable.

See attachment. The LHT group described in the Informed Consent document includes LHT (heat) and CON (control; sham - low-temperature heat pads).

10-1b(27)a If you attach a document, state *See Attachment* in the text box above.

*required

Who will be conducting the intervention(s)? What are their qualifications?

Attach any documentation if applicable.

For Oral Glucose Tolerance Testing (OGTT): Drs. Luk and Levitt are experienced in phlebotomy; Dr. Levitt has carried out the OGTT procedure. Additional study team members are trained in phlebotomy (Casey Appell) and will help carry out this procedure under the supervision of Drs. Luk and Levitt.

For body composition measurement, Drs. Luk and Levitt are certified DEXA technicians in the State of Texas and are approved to operate the DEXA housed in the Department of Kinesiology & Sport Management (KSM) at Texas Tech University (TTU). Their students trained on use of the DEXA will carry out this procedure under their supervision.

10-1b(28)

All biopsy procedures will be performed by Dr. Hui-Ying Luk, who is not a licensed physician but is trained specifically in the muscle biopsy procedure. Robyn Richmond, M.D. (robyn.richmond@ttuhsc.edu) will oversee the procedure (i.e., approve safety protocols, provide technical and medical advice regarding the procedures and potential adverse effects of biopsy). Dr. Richmond will determine the competency of the person who will perform the preparation (sterilized procedures, lidocaine injection) and the operation (maintaining sterile procedure for the primary operator: Dr. Luk). Since the assistant (applying suction through a syringe) does not have direct contact with the participant and does not require sterile procedures, the competency of the assistant will be determined by Dr. Luk. Our members are not going to diagnose these symptoms, instead, if the participant is self-reporting pain, erythema (warmth), or ecchymosis (bruising) to us, we will advise them to use a cold pack. Also, our members will follow up with them regarding the self-reported signs. Similarly, if the participant is seeing and self-reporting signs of infection, inflammation, hematoma (e.g., prolonged redness, pus formation, increasing pain, bruising at the site of biopsy, and fever, etc.), and numbness at the site of biopsy, we will refer them to consult with a medical provider. Lastly, an individual with CPR training (current) will be on-site within close proximity to the subject during procedures.

All intramuscular temperature measurement will be performed by Drs. Hui Ying Luk and Danielle Levitt who are trained specifically in this procedure by Dr. Steven Romero. The proposed technique has been safely and successfully implemented in participants (e.g., PMID: 38059292).

For exercise testing and intervention, Drs. Luk and Levitt have experience in exercise testing and intervention and are Certified Strength and Conditioning Specialists (CSCS) through the National Strength and Conditioning Association (NSCA). The proposed exercise capacity testing and HIIT intervention have been safely and successfully implemented in older adult participants (e.g. PMID: 29077855, 28273480). Dr. Luk and

Levitt will train and supervise all personnel (graduate and undergraduate students on this IRB application) in conducting the exercise testing and intervention included herein. Only study personnel with currect CPR and first aid training will perform exercise testing and intervention. Dr. Levitt has an up-to-date CPR and first aid certification, and has held valid CPR certification continuously since 2003.

For frailty risk assessment, Dr. Levitt has assessed frailty risk in people living with HIV. She will train and supervise all personnel (graduate and undergraduate students on this IRB application) in conducting the physical function testing to assess frailty risk.

If you attach a document, state *See Attachment* in the text box above.

10-1b(28)a

incidents updated (1) (2) (2) (2) (1).docx

IM temp and heat therapy in older adults.docx

Other

Deception

10-2

The result of investigators providing false or incomplete information to participants for the purpose of misleading research subjects.

*required

10-2a **Does this research project involve the use of deception?**

✓ Yes

*required

10-2a(1) **Explain why deception is necessary.**

A sham group is needed for scientific purposes.

No

10-3 **Debriefing**

10-3a **Will a debriefing script be used at the conclusion of the research?**

✓ Yes

*required

10-3a(1) **Select the reason for needing a debriefing script.**

✓ Deception

Sexual Misconduct

Mental Health, Self-harm, Substance Abuse, Criminal Behavior

✓ Education Tool

*required

10-3a(2) **Enter or attach a copy of the debriefing script.**

See attachment

If you attach a document, state *See Attachment* in the text box above.

10-3a(2)a [Biopsy care kit.doc]

[Deception Script.docx]

[IM care kit.doc]

*required

Provide information on who will conduct the debriefing and how it will be delivered to the participant.

Dr. Luk, Dr. Levitt, or clinical coordinator (future) will conduct the debriefing.

Upon the completion of the biopsy procedures, we will provide a take-home

10-3a(3) kit (i.e., notes and necessary consumables, e.g., extra bandage, alcohol wipes). Also, we will verbally explain the biopsy care instructions and a list of drugs that can increase bleeding (see the attachment), especially focusing on the medication that should be avoided. Furthermore, our research team will follow up with the participant in the following two days by text/call to make sure no adverse effect was observed (swelling and warmth at the site of biopsy). You can also send us a photo of the biopsy site through email or text message if you have any concerns. We will look at your biopsy site on your next visit.

For the control group (CON), we will disclose that the temperature setting for the heat pad used during the study was for control purposes, and we will specify the temperature they need to set to achieve an intramuscular temperature of 40 degrees Celsius.

No

10-4 TTU Compliance Committees

Additional approvals

*required

10-4a Does this research require internal approval from another TTU compliance committee or entity?

✓ Yes

10-4a(1) Select the TTU Committee(s) that grants approval to conduct research at that location and attach the approval letter.

Texas Tech Neuroimaging Institute (TTNI)

✓ TTU Institutional Biosafety Committee (IBC)

*required

Attach the approval letter.

Luk_IBC.pdf

IBC-2023-1083 approved v9.pdf

10-4a(1)b

2023-2025_KSM_DXA_Sublicense.pdf

Luk_DEXA training.pdf

Luk_Handling Radioactive Material.pdf

Luk_Introduction to Radiation Safety.pdf

Luk_Radiation Safety.pdf

Luk_X-Ray Safety.pdf

✓ TTU Institutional Laboratory Safety Committee (ILSC)
*required

10-4a(1)c

Attach the approval letter.

Luk_IBC.pdf

IBC-2023-1083 approved v9.pdf

2023-2025_KSM_DXA_Sublicense.pdf

Luk_DEXA training.pdf

Luk_Handling Radioactive Material.pdf

Luk_Introduction to Radiation Safety.pdf

Luk_Radiation Safety.pdf

Luk_X-Ray Safety.pdf

Institutional Animal Care and Use Committee (IACUC)

✓ TTU Radiation and Laser Safety Committee (RLSC)
*required

Attach the approval letter.

Luk_IBC.pdf

IBC-2023-1083 approved v9.pdf

2023-2025_KSM_DXA_Sublicense.pdf

10-4a(1)e Luk_DEXA training.pdf

Luk_Handling Radioactive Material.pdf

Luk_Introduction to Radiation Safety.pdf

Luk_Radiation Safety.pdf

Luk_X-Ray Safety.pdf

Other

No

Complete the following section.

11-1 Personal Identifiers

*required

11-1a Select personal identifiers that will be collected for the purpose of this research.

Check any identifier you will be in possession of during the research process.

- ✓ Names
- ✓ Telephone numbers
- ✓ Any elements of dates (other than year) for dates directly related to an individual, birth date, admission date, discharge date, date of death. For ages over 89 all elements of dates (including year) indicative of such age, expect that ages and elements may be aggregated into a single category of age 90 and older
- Any geographic subdivisions smaller than a state, including street address, city, county, precinct, zip code and their equivalent geocodes except for the initial three digits of a zip code
- Fax numbers
- ✓ E-mail addresses
- Social security number
- Medical record number
- Student ID username/number
- Health plan beneficiary numbers
- Account numbers

Certificate/license numbers

Vehicle identifiers and serial numbers (VIN), including license tag identification

Device identifiers and serial numbers (e.g., implanted medical device)

Web universal resource locators (URLs)

Internet protocol (IP) address numbers

Biometric identifiers, including finger and voice prints

Full face photographic images and any comparable images (i.e., videos, etc.)

✔ Biological specimens

Any other unique identifying number, code, or characteristic, other than dummy identifiers that are not derived from actual identifiers and for which the re-identification key is maintained by the health care provider and not disclosed to the researcher

No personal identifiers will be collected

11-2 **Privacy of Participants**

---

*required

11-2a **Describe how you will protect the privacy of the participants.**

Consider how you will protect the participant's privacy during:

- Recruitment
- Consent
- Data Collection
- Storage of Data
- Analysis of Data
- Findings

Data Safety:
To protect against breach of confidentiality, any hard copy materials (e.g., informed consent form, any hard copies of exercise records with heart rate and rating of perceived exertion

data, etc.) will be kept in a locked drawer in Dr. Luk's private office. The informed consent form and demographics files will be the only places participants' names appear, and only the PIs and essential study personnel will have access. Participant samples, data, and records will be deidentified and labeled using a unique ID number. Data, images, and files generated from the project will always be stored in raw form, separate from files with calculations and analyses. Lastly, all electronic material, including data from questionnaires administered using computer-assisted interview techniques (i.e., no hard copy) will be stored in a secure database (REDCap).

11-3 De-Identifying Data

[Anonymous v. Identifiable Data]

*required

Explain how you will de-identify the data collected. If data will be given a code, explain this process.

For example:
Personal identifiers will be removed from the data and replaced with a participant code. The participant code will be used on the individual's data. The master list containing the personal identifier(s) and participant code will be in a separate document stored away from the data. The master list and data will be password protected. The passwords will be different for the master list and data.

For each identifier explain when and how you will code/de-identify/destroy the identifier.

11-3a Consider the following:

- What identifiers are associated with data?
- What identifiers are NOT associated with the data?
- What descriptors can be used to identify someone? For example: schools, towns, buildings, colleagues, companies, titles, etc.

Upon participant's admission to the study, a unique study identification code (ID codes) will be assigned to the participant in the order they are enrolled. The file containing participants' names and IDs will be stored electronically in REDCap.

Thereafter, all data will be kept in coded participant files. All trained investigators, professional staff, and technicians are aware of the confidentiality involved with this study.

Identifiable data will not be available or divulged to anyone outside of the experimental research team and data files will be kept for at least 3 years after the study is terminated. The confidentiality of individual information will be maintained in any publications or presentations regarding this study.

11-4 **Data Transcription**

*required

11-4a **Select who will transcribe audio or voice recording.**

Check all that apply.

Member of the research team/lab

Professional transcription company

Other

✓ Not applicable

11-5 **Transfer of Data**

*required

11-5a **Will data be transferred from one location to another?**

Transfer of data can be done electronically (email, removable storage device, shared drives, remote access, hyperlinks, etc.) and/or physically (carrying, mailing, etc).

✓ Yes

*required

Explain how it will be securely transported and by whom.

1. Data type (human subjects data only)

A. **Types and amount of scientific data expected to be generated in the project:**

Data from human subjects: Demographic and anthropometric (e.g., age, sex, race, ethnicity, body mass, body composition, height), clinical (e.g., medical history, oral glucose tolerance test parameters, and questionnaires about alcohol use, diet, activity, sleep, changes in body mass, and fatigue), functional (e.g., exercise capacity, grip and leg strength, gait speed), and molecular (e.g., type I and II muscle fiber cross-sectional area, capillarization, mitochondrial enzyme activity, mitochondrial and TRPV1 protein expression, mitochondrial DNA quantification, and mitochondrial content and morphology) data will be collected from 54 older adults at risk for type 2 diabetes (described in detail in the "Human Subjects" attachment) at several time points throughout the study. Weekly assessments will include body mass measurement, diet and alcohol questionnaires, and activity and sleep monitoring via and self-report questionnaires. All other data will be collected only at the beginning of the study (i.e., demographics, medical history) or at the 2-3 major time points. Participants assigned to the high-intensity interval training (HIIT) group (n=18) for phase 1 will have 2 major time points (before [T1] and after [T2] phase 1). Participants assigned to the LHT (n=18) and CON (n=18) groups for phase 1 and HIIT for phase 2 will have 3 major time points (before phase 1 [T1], between phases 1 and 2 [T2], and after phase 2 [T3]).

Large-scale data sets: It is possible that large scale mitochondrial genomic data (human participants) and proteomic data (cell culture) could be generated from this project if alternative approaches must be used as described in the research strategy.

The total amount of anticipated data if alternative approaches are not needed is ~25 GB of data.

B. **Scientific data that will be preserved, shared, and the rationale for doing so:** All raw data generated from this project will be stored in a password-protected manner on cloud-based servers at Texas Tech University. Cleaned data in an analysis-ready format will be stored in REDCap and periodically uploaded to repositories. Data of sufficient quality will be uploaded to allow others to validate and replicate research findings described in the Aims.

C. **Metadata, other relevant data, and associated documentation**: In alignment with the Data Submission Worksheet for the Aging Research Biobank, the following will be submitted to accompany data derived from human subjects:

•Full study protocol
•Names and descriptions of data sets
•Manual of operations

•Annotated data collection forms
•Data dictionary
•Documentation of calculated variables
•Summary of deidentification and crosslinking of study ID with new randomized ID
•Any changes made to the protocol over time
•Frozen datasets used for the primary publication, when applicable
Similarly, the full study protocol, dataset names/descriptions, data dictionary, documentation of calculated variables, and any changes to the protocol over time will be deposited in TTU Dataverse for molecular procedures.

2. **Related tools, software, and/or code:** Statistical analyses will be run using standard statistical software (SPSS version 29) and graphs made using GraphPad Prism (Version 9). Image processing will be performed using ImageJ and in part using custom Python code to automate quantification where appropriate.

3. **Standards:** No consensus standards of data formatting such as Dublin or Darwin core exist for our discipline. Data dictionaries defining each variable and decoding any coded responses will accompany all uploaded data.

4. **Data preservation, access, and associated timelines:**
A. Repository where scientific data and metadata will be archived:
Human subjects data: NIA's Aging Research Biobank
Molecular data: TTU Dataverse Collection
Mitochondrial genomic data: Database of Genotypes and Phenotypes
11-5a(1) (dbGaP; if performed)
Custom code: GitHub

B. How scientific data will be findable and identifiable: Data will be findable for the research community through the repositories listed above. Collections will be established in the Aging Research Biobank and in Dataverse when this application is funded. Collections will be established on dbGaP should a large data set be generated. Data stored in the Aging Research Biobank will be made available by reasonable request, and this will be noted in the "Data Availability Statement" in associated publications. When data are made public via Dataverse, a digital object identifier (DOI) will be assigned. This DOI be referenced in associated publications to allow the research community access to the data used in each publication. If dbGaP is used, researchers can submit requests to dbGaP to access and browse data, and this information will be included in any associated publications. Any custom code will be shared on GitHub, accessible by searching "rbudnar" on GitHub. The readme.md file for the project will include instructions for the analyses and rationale for parameter choices.

C. When and how long the scientific data will be made available: Data associated with each publication will be made available when preprints are available and will continue to be available on the repositories and long as

they are supported. Any data that are not yet available by the end of the award period will be made available at that time.

5. Access, distribution, or reuse considerations:
A. Factors affecting subsequent access, distribution, or reuse of scientific data: Broad data sharing will be included in the informed consent form. Data deidentification, linking data using unique identifiers, protection of privacy, and making data available will be explained to participants and any questions answered as part of the informed consent process.

B. Whether access to scientific data will be controlled: Access to human subjects data will be controlled. Requests will be submitted through the appropriate repository (Aging Research Biobank, Dataverse, dbGaP).

C. Protections for privacy, rights, and confidentiality of human research participants: The only location that personal information will be paired with participant ID will be on a single administrative data collection form in the secure REDCap database. This is necessary because data will be collected from the same participants and multiple time points. However, each participant will be assigned an ID code and only the ID code will be linked to data so that the data are not identifiable. Data from human participants will be further deidentified upon submission to the repository(ies) and linked only through the study ID. The Aging Research Biobank further anonymizes data by randomly generating a new ID for each subject. dbGaP interfaces with the Aging Research Biobank so clinical and mitochondrial genomic data (if generated) can be linked, using only the randomly generated ID. Further, access to these datasets will be controlled and require that users register and submit reasonable requests. The PIs attest that no attempt will be made to reidentify participants from deidentified data.

6. Oversight of DMS: Drs. Hui-Ying Luk and Danielle Levitt (PIs) will deposit clinical and associated data into the NIA Aging Research Biobank, TTU Dataverse, and into dbGaP if mitochondrial genomic data are generated. Data upload will occur when publications associated with each data set are submitted and made public (where applicable) upon acceptance. Data sharing will be included in the annual progress report.

7. Representatives of the following people/groups within *Texas Tech University* may use your study records and share them with other specific groups in connection with this research study.
• The principal investigators (*Hui Ying Luk, Danielle Levitt*)
• The *Texas Tech University* Institutional Review Board
• The *Texas Tech University* Human Subjects Protection Office
The above people/groups may share your unidentifable study records with the following people/groups outside *Texas Tech University* for their use in connection with this research study. These groups, while monitoring the research study, may also review and/or copy your original *Texas Tech University* records.

• The Office of Human Research Protections in the U. S. Department of Health and Human Services.
• The Safety Officer appointed for this study.
• The National Institutes of Health and its authorized representatives.

No

11-6 Certificate of Confidentiality

*required

11-6a Has a Certificate of Confidentiality been requested?

Yes

✓ No

11-7 Investigator Assurances

*required

11-7a Data Security

✓ I will protect the data through at least one of these security measures: encryption software, password protection, and /or physical barrier (locked doors, file cabinets, etc.).

I will NOT protect the data through at least one of these security measures: encryption software, password protection, and /or physical barrier (locked doors, file cabinets, etc.).

*required

11-7b **Access to Data**

✓ I will only allow the research team access to the identifiable data.

I will allow others outside of the research team to have access to identifiable data.

*required

11-7c **Destruction of Personal Identifiers**

✓ I will destroy personal identifiers prior to closing the IRB with the exception of the consent forms.

I will **NOT** destroy personal identifiers prior to closing the IRB with the exception of the consent forms.

*required

11-7d **Retention Policy**

✓ I will keep de-identified data and/or consent forms for at least 3 years following the closure of the IRB.

I will **NOT** keep de-identified data and/or consent forms for at least 3 years following the closure of the IRB.

*required

11-7e **Data Repository**

✓ I will post the de-identified data to a data repository.

I will **NOT** post the de-identified data to a data repository.

12: Benefits & Risks

Please respond to the following statements/questions to help our IRB reviewers recognize the benefits and risks associated with this study.

12-1 **Benefits**

*required

Describe the potential direct benefit(s) to subjects.

12-1a

Payments to subjects, course credit or contribution to science should not be included in this section.

All efforts will be made throughout the study to minimize any study-related risks to subjects. The Physical Activity Guidelines for Americans, 2nd Edition (2018), suggest that adults should engage in at least 150 minutes per week of moderate-intensity aerobic exercise (or an equivalent 75 minutes per week of vigorous-intensity aerobic exercise, including HIIT) to maximize the many health benefits of aerobic exercise. Even if people do not meet the minimum of 150 minutes, smaller amounts of exercise are still beneficial. Participating in this study will provide participants with a structured environment to help meet that exercise goal during one of the two phases. Among anticipated benefits from exercise, we emphasize that there might be metabolic health benefits and decreased frailty risk, particularly because participants will be prediabetic older adults, indicating risk for the development of type 2 diabetes and frailty.

Furthermore, benefits of heat therapy are becoming increasingly recognized and we anticipate that participants assigned to the local heat therapy group will have improved skeletal muscle health, glycemic control, and decreased frailty risk. Because of the benefits anticipated from local heat therapy, all participants will be provided with heating pads regardless of group. The HIIT group will be provided with heating pads and instructions for use at the end of their participation (T2). The control group will be provided with heating pads but instructed to use a lower heat setting T1. At the end of their study participation (T3), they will be provided with instructions for use. Regardless of group, participants will have regular interaction with members of the study team. Together with exercise, prosocial interaction may have psychological benefits.

Participants will be provided with the following non-diagnostic results from testing performed at each major time point (PRE, MID, and POST):

- Dual-energy X-ray absorptiometry (DEXA) reports: Body composition and bone mineral density compared to age- and sex-specific norms.
- Body mass index (BMI): Value calculated from body mass and height (kg/m^2) and BMI category (i.e., underweight, normal weight, overweight, obese).
- Gait speed: 4-m walk time compared to age- and sex- specific norms.
- Muscle strength: Average grip strength (3 trials per hand) and 30-second sit-to-stand test with comparison to age- and sex- specific norms.
- Exercise capacity: VO_{2peak} and comparison to age- and sex-specific norms.
- Blood sugar control: If fasting blood sugar, HbA1c, or 2-hr glucose values after the glucose tolerance test fall into the diabetic range at any time point, the participant will receive a letter advising you of the laboratory measurement and to follow up with your physician.

12-2 **Risks**

*required

Describe any potential risk to subjects and how you will minimize that risk.

While exercise and heat have many potential benefits, they always carry possible risks. These risks are minimal, and the benefits of exercise and heat therapy largely outweigh the risks of a sedentary lifestyle. Those with higher-than-average risk for complications of exercise (e.g., preexisting cardiovascular conditions) will be excluded from the study. Risks of sample collection are also minimal, and the precautions taken will further offset these risks. The qualifications of the PIs and the protections in place for human subjects will ensure a safe environment for study participation.

Protections against risk
Strategies for minimizing risks associated with medical history and questionnaires, collection of biological specimens, and exercise are described below. All procedures will be fully approved by the TTU IRB. Drs. Luk and Levitt are trained in phlebotomy and muscle biopsies techniques and have previously conducted these procedures in IRB-approved studies. Dr. Luk is currently approved and actively performing muscle biopsies at TTU, and Dr. Levitt is in the process of obtaining such approval. All study personnel will be required to maintain valid CPR and First Aid certifications, and emergency contact information will be kept on hand. All personal information and data will be kept in a secure database (REDCap).

Protection against and minimization of risks by procedure:
•Interviews and completing questionnaires: To minimize risks of emotional stress and fatigue, the length and number of questionnaires will be kept to a minimum, and the study team will establish rapport with participants to minimize emotional stress. Contact information for

guidance counselors will be available. Protection against breach of confidentiality will be ensured by keeping all physical study materials in locked, secure locations and all electronic material in a secure database (REDCap). Except for the demographics form, all data will only be linked with unique identifiers and not with any personally identifiable information. Data uploaded to repositories will have different randomly generated unique identifiers generated during upload to the NIA Aging Research Biobank that are different from those used in REDCap or on study forms.

•Blood collection: Participants will be informed of the risks of venipuncture and finger prick. Risk for infection is low with the use of sterile technique, and a sterile adhesive bandage will be applied to the blood collection sites. Blood collection will be performed by trained personnel using standard laboratory techniques: disinfecting the site with alcohol, using sterile disposable supplies, and applying pressure with sterile gauze to minimize bleeding and bruising. Syncope is possible, so phlebotomy procedures will be performed using phlebotomy chairs in case legs need to be elevated. Cold packs will be available. Participants will be asked if they have a history of fainting prior to blood collection.

•Muscle biopsy: The following are risks that are related to the muscle biopsy procedure and are only associated with/around the incision site: Pain, bleeding, excessive redness, bruising, tenderness, swelling, warmness of the skin, localized numbness, and pink-tinted drainage from the incision. In extreme cases, a low-grade fever may occur, which would indicate that the site may be infected. None of these complications are usually life-threatening, and most complications can be managed conservatively without hospitalization.

To minimize risks, muscle biopsies will be performed using standard sterile technique. The skin over the vastus lateralis will be cleaned using povidone-iodine and the area anesthetized using 1% lidocaine without epinephrine. A small incision will be made through the skin and fascia using a single-use sterile scalpel (#11). A sterilized Bergstrom biopsy needle will be inserted into the muscle and approximately 200 mg of muscle will be collected. After the sample has been collected, pressure will be applied to the area for 5 min. The skin will be closed with a sterile adhesive bandage, covered using sterile gauze, and compression wrapped. Each participant will be given verbal and written instructions for biopsy care and extra supplies (e.g., sterile adhesive bandages, alcohol pads) will be provided. The study team will follow up with each participant 2 and 5 days following their biopsies. Sterile supplies (gloves, gauze, scalpel, needles, etc.) will be used and the PIs will train personnel to assist in the procedure. In case a participant becomes lightheaded or feels faint, we will provide food or beverages containing simple sugar (e.g., juice).

• Intramuscular temperature measurement: There may be some discomfort during the insertion of the probe into your muscle. The topical anesthetic will numb the insertion area to minimize this discomfort, but you may feel pressure or a dull ache in the muscle as the needle moves through the muscle. At the end of the session, the probe will be withdrawn and a sterile dressing will be applied. Any swelling or redness after the study should be gone a few hours after completion of the study, but you may feel some muscle soreness for several days. Although the small probes are sterile, there is a slight risk of infection at the sites where the probes were placed. You will be instructed on how to keep the area clean
12-2a for a day or two following the study and will need to inform the researchers immediately

if you have any redness or swelling in the area. To minimize risks, this measurement will be performed using standard sterile technique. The skin over the incision site will be cleaned using povidone-iodine and the area anesthetized using topical anesthetic cream. At the end of the session, the probe will be withdrawn and the skin will be closed with a sterile adhesive bandage, covered using sterile gauze, and compression wrapped. Each participant will be given verbal and written instructions for care and extra supplies (e.g., sterile adhesive bandages, alcohol pads) will be provided. Sites will also be re-assessed at the next study visit.

•Anthropometric measurements: To minimize risks associated with fatigue, these procedures will be performed near a wall or other support and chairs kept nearby in case participants need to rest between measurements. Contact information for guidance counselors will be available.

•Dual-energy x-ray absorptiometry (DEXA): DEXA is considered a low-risk procedure. However, the use of x-rays, even in low amounts, does present some risk associated with ionizing radiation. To minimize these risks, the number of DEXA scans will be kept to a minimum.

•Functional tests, exercise capacity (VO2peak) testing, and HIIT: During screening, we will ensure that participants meet ACSM medical criteria for initiating an exercise program and do not have medical complications that would put them at undue risk (e.g., diabetes, renal disease, uncontrolled hypertension, other cardiovascular disease, etc.). Participants may become faint, nauseous, experience muscle strains or soreness, or fall. To minimize risk associated with physical activity, participants will be counseled on the importance of proper hydration and will regularly be asked about their physical condition and adjustments made as necessary. Chairs will be available for rest during functional testing, and a researcher will remain close by the participant in case they lose their balance. During VO2peak testing and HIIT, we will utilize cycle-based exercise instead of treadmill exercise to prevent the risk of falls while walking, and participants will be helped on and off the cycle ergometer or exercise bicycle as needed. Participants will perform proper warm-ups and cool downs. We will also monitor heart rate and rating of perceived exertion during exercise. The VO2peak test will be performed in 3-minute stages with predetermined power output (i.e., males start at 50W, females start at 25W, and power increases by 20W every 3 minutes). If the participants cannot maintain the power output for a given stage, their cycling cadence falls below 50 rpm for two consecutive minutes, or they ask to stop the test, the test will end. In case a participant becomes lightheaded or feels faint, that day's exercise will be stopped and we will provide food or beverages containing simple sugar (e.g., juice).

•Local heat therapy: The potential risks of using local heat therapy include discomfort, skin redness, and, in rare cases, burns, particularly if used improperly or for extended periods. Core temperature increases are not typically observed with local heating on the thigh and thus is not considered a potential risk for the proposed study. However, we will monitor body temperature using a forehead scanner. If the body temperature reaches 40°C, we will stop the heat pad therapy. The risk of discomfort will be minimized by determining the lowest optimal heat pad temperature prior to the onset of the heat therapy intervention and that temperature will not be exceeded. Further, participants will be provided with contact information for the PIs should any problems occur during their at-home local heat therapy

sessions and will be asked about any discomfort during their weekly check-ins. Sometimes, redness may appear on your skin in a pattern after the heat therapy session, but it should fade within a day. Heat therapy sessions will occur in the laboratory once weekly and will be monitored by study personnel to ensure that no problems occur during heating sessions. The risk of burns due to personal heating pad use in older adults is not well-documented. However, in a 2021 study (PMID: 33031521), a review of burn injuries in older adults (age 65+) registered in the National Electronic Injury Surveillance System was performed. For adults aged 65-84 years, heating pads were not in the top 5 causes of burn injuries. In the 85+ year-old age group, heating pads were in the top 5 causes of burn injuries. An estimated 204 burns across 100 emergency departments in the United States over a 10-year period were attributable to electric heating pad use over a ten-year period, and all were in women. The risk of burns due to heating pads in this age group is increased by chronic comorbidities that are exclusionary for this study (i.e., diabetes, peripheral vascular disease, chronic kidney disease, neuropathy), minimizing risk. The risk also increases if heating pads are used while sleeping, so we will instruct participants to set a timer and/or alarm to ensure they do not exceed the 90-minute application time, also minimizing risk. Finally, it is estimated that a surface temperature between 42-43°C would produce a burn injury in 12-20 hours (PMID:1885636). Because the difference between skin and muscle temperature is approximately 2°C across a variety of studies, we anticipate that the heating pad setting to produce an intramuscular temperature of 40°C will be approximately 42°C. The 90-minute application time is far below the conservative end of the estimated contact time frame to produce a burn injury (12 hours).

12-2a(1) **Is a liability plan offered?**

Yes

✓ No

13: Participant Compensation

13-1 Compensation - Payments to Research Participants

Compensation through TTU funds must comply with TTU OP 62.25.

*required

13-1a **Are subjects being compensated for their participation in this study?**

✓ Yes

*required

Describe the compensation process.

13-1a(1)

Participant will be paid via cash upon completion of each visit:

Consent/screening ($10)

Major Measurement Testing (PRE, MID, POST):
•Muscle biopsy ($50) and OGTT/Questionnaires ($50)
•Physical function and exercise capacity tests ($50)
The major time point testing will be conducted twice for the HIIT group and three times for Local Heat groups.

For each laboratory visit, participants will be compensated $15.
Phase 1:
•LHT: 12 visits = $180
•HIIT: 36 visits = $540

Phase 2:
•LHT: 36 visits = $540

Total:
•LHT: $1180
•HIIT: $850

13-1a(2) **Select the payment type used.**

✓ Cash

*required

Amount of Payment

13-1a(2)a

Amount will be paid according to the completion of each visit, please see above for details. Participants who are assigned to the LHT groups and complete each visit in its entirety will receive a total of $1180. Participants who are assigned to the HIIT groups and complete each visit in its entirety will receive a total of $850.

*required

13-1a(2)b

When and how will the participant be paid?

payments in cash will be disbursed after each visit

Check

Gift Card (not to be checked if a drawing is involved)

Drawing

Course Credit

Other (includes payment or credit from 3rd party affiliations (i.e., MTurk, SSI, etc.)

Investigator Assurances

Guidance: The Institutional Review Board (IRB) should determine that the risks to subjects are reasonable in relation to anticipated benefits [21 CFR 56.111(a)(2)] and that the consent document contains an adequate description of the study procedures [21 CFR 50.25(a)(1)] as well as the risks [21 CFR 50.25(a)(2)] and benefits [21 CFR 50.25(a)(3)]. It is not uncommon for subjects to be paid for their participation in research, especially in the early phases of investigational drug, biologic or device development. Payment to research subjects for participation in studies is not considered a benefit, it is a recruitment incentive. Financial incentives are often used when health benefits to subjects are remote or non-existent. The amount and schedule of all payments should be presented to the IRB at the time of initial review. The IRB should review both the amount of payment and the proposed method and timing of disbursement to assure that neither are coercive or present undue

influence [21 CFR 50.20].

13-1a(3)

Any credit for payment should accrue as the study progresses and not be contingent upon the subject completing the entire study. Unless it creates undue inconvenience or a coercive practice, payment to subjects who withdraw from the study may be made at the time they would have completed the study (or completed a phase of the study) had they not withdrawn. For example, in a study lasting only a few days, an IRB may find it permissible to allow a single payment date at the end of the study, even to subjects who had withdrawn before that date.

While the entire payment should not be contingent upon completion of the entire study, payment of a small proportion as an incentive for completion of the study is acceptable to FDA, providing that such incentive is not coercive. The IRB should determine that the amount paid as a bonus for completion is reasonable and not so large as to unduly induce subjects to stay in the study when they would otherwise have withdrawn. All information concerning payment, including the amount and schedule of payment(s), should be set forth in the informed consent document.

---

*required

13-1a(3)a **Participant Payment**

✓ I will provide compensation to all participants who complete the consent process and begin the study, regardless of whether they complete the study.

I will **NOT** provide compensation to all participants who complete the consent process and begin the study, regardless of whether they complete the study.

---

*required

13-1a(3)b **TTU OP 62.25**

All questions concerning participant payment should be directed to the TTU Payroll & Tax Services office or the researcher's business manager.

✓ I will follow the procedures outlined in TTU OP 62.25 regarding participant payment.

I will **NOT** follow the procedures outlined in TTU OP 62.25 regarding participant payment.

No

Conflict of Interest

Guidance: A conflict of interest refers to a situation in which an employee(s) financial, professional, or other personal considerations may directly or indirectly affect, or have the appearance of affecting, the employee(s) judgment in exercising any duty or responsibility, including the conduct or reporting of research, owed to the institution (TTU OP 10.20 (2)(d)).

14-1

"Significant business or financial interest" means anything of monetary value including, but not limited to, salary or other payments for services (e.g., consulting fees or honorarium; equity interests (e.g., stocks, stock options, or other ownership interests); and intellectual property rights (e.g., patents, copyrights, and royalties from such rights). (TTU OP 74.17).

"...a set of conditions in which an investigator's judgment concerning a primary interest (e.g., subject welfare, integrity of research) could be biased by a secondary interest (e.g., personal or financial gain)." Institutional Review Board Management and Function, Bankert and Amdur, 2006, p. 167.

*required

14-1a **Does anyone on the research team have a potential conflict of interest with the research project?**

Yes

✓ No

15: Investigator Attestations

*required

I, as the PI and co-investigators, understand Texas Tech University's policy concerning research involving human subjects and by checking below, I certify:

- ✓ I have read The Belmont Report "Ethical Principles and Guidelines for the Protection of Human Subjects of Research" and subscribe to the principles it contains.
- ✓ I have read 45 CFR 46, Protection of Human Subjects and subscribe to the regulations it contains.
- ✓ I accept responsibility for the scientific and ethical conduct of this research study and understand my responsibility.
- ✓ I will ensure that all study personnel are appropriately trained and are competent to perform the study.
- ✓ I will obtain prior approval from the Institutional Review Board (IRB) before making any modifications of the previously approved research, including modifications to the informed consent process and document.
- ✓ I will submit a Study Closure Submission upon completion of this study and agree to honor any other commitments.

✓ I will follow TTU procedures and guidelines to protect the health and well-being of research participants, research team members, and liaison(s) during any unforeseen circumstances.

✓ I will immediately report to the IRB any deviations, violations, non-compliance, unanticipated problems, and adverse events related to subjects or others which occurred or possibly occurred as a result of this study.

45 CFR 46.103(a) and TTU IRB Policy 5.4 require Adverse Events, Unanticipated Problems and Deviations from the research protocol to be reported **IMMEDIATELY** to the HRPP office.

Human Research Protection Program
Box 41075
Lubbock, Texas 79409
Phone: (806) 742-2064
Email: hrpp@ttu.edu

16: Additional Information

16-1 **Include any additional points to help clarify the research, if applicable.**

16-1a

16-2 **Enter or attach any additional documentation.**

16-2a

Modification Submission

Modification

*required

Cayuse IRB Modification:

Provide a summary of the changes you are requesting in the text box below. Details of the changes are required in the appropriate sections. The "Compare" feature is a useful tool as you edit each section.

Summary of Changes.

1) We have simplified the flyer.
2) We have added the following statement in the informed consent under "we will happen to my data" "A description of this clinical trial (NCT06580964) will be available on http://www.ClinicalTrials.gov as required by U.S. Law. This website will not include information that can identify you. At most, the Web site will include a summary of the results. You can search this website at any time."
3) *Following extensive piloting, we have revised the heat pad protocol, including adjustments to the setup and the intramuscular temperature target.*
4) *Piloting revealed that thoroughly explaining the questionnaires took more time than expected. Therefore, we will move the questionnaire familiarization from T1 Day 1 to the consent/screening visit. Due to the addition of questionnaires familiarization to the consent/screening visit, the time required for this day will increase from 1 hour to 2 hours. To accommodate this change, we have reduced the visit time on Day 1 from 5 hours to 4-5 hours. As a result, the total time commitment for the entire study remains unchanged.*
5) *After piloting, we determined that using skin temperature to estimate intramuscular temperature was not feasible within our study protocol. As a result, we have removed the estimation equation. However, we continue to record skin temperature for monitoring participant safety and comfort.*
6) We have added Ben Barr (postdoc) under the research team.

*required

Current Research Activities

Check all that apply.

Research procedures have **NOT** started.

✔ Participants are being recruited for this research.

*required

Explain the recruitment procedures that have started.

Fliers have been posted, and two potential participants have been pre-screened for the study.

Recruitment has ended.

Participants have been consented to do the research.

Participants have **NOT** been consented to do the research.

Data collection is **ONGOING.**

Data collection is **COMPLETE.**

Analysis of **IDENTIFIABLE** data.

Analysis of **DE-IDENTIFIED** data.

1: Human Subject Research

Answer the following questions to determine if you need IRB review and approval.

1-1 **Research**

---

*required

Research is defined by 45 CFR 46.102(l) as:

A systematic investigation, including research development, testing and evaluation, designed to develop or contribute to generalizable knowledge.

Does your proposed study meet the definition of research?

✓ Yes

No

1-2 **Human Subjects**

---

*required

Human Subject is defined by 45 CFR 46.102(e) as:

A living individual about whom an investigator (whether professional or student) conducting research

- Obtains or receives private identifiable information or identifiable biospecimens about the individual(s)
- Obtains information or biospecimens through intervention or interaction with the individual(s)

Does your proposed study involve and meet the definition of human subjects?

✓ Yes

No

If you answered YES to all questions in Section 1 complete the rest of the sections.

Complete the following section.

*required

Review Type

2-1

Select the appropriate review type.

Exempt

Expedited

✓ Full Board

The project involves greater than minimal risk to human subjects.

Relying on IRB approval from another institution (IAA)

External Collaboration

2-2

Only select collaboration if there are Non Texas Tech researchers, employees, and/or students working on this research.

Collaboration

International Research

2-3

Research will be conducted outside of the United States of America and/or data will be shared across borders.

International Research

Complete the following section.

Lay Summary

*required

Provide a description of your study in vernacular language.

Avoid the use of technical terms, undefined acronyms, or excessive scientific terminology.

Aging people with prediabetes are at increased risk for frailty, and skeletal muscle architectural factors (which may improve with exercise or heat therapy) could underlie development of frailty and progression to type 2 diabetes. The proposed study will test local heat therapy as a method to improve skeletal muscle architecture (i.e., cross sectional area, capillarization, mitochondria), glucose tolerance, frailty indicators, and exercise adaptation in prediabetic older adults. Data generated will provide evidence supporting a directly translatable, easily implemented therapeutic intervention to ameliorate risk of developing overt type 2 diabetes and frailty in at-risk aging individuals.

Complete the following section. List all researchers involved with recruitment, data collection, and analysis.

NOTICE: HUMAN SUBJECT TRAINING REQUIREMENT

All research study personnel who engage with participants will need to complete human subject training prior to IRB approval and prior to conducting any human research-related activities. The new training requirement will be applied when an existing study is due for renewal, requires modification(s), or is chosen for a post approval monitoring.

Human research-related activities include recruitment of participants, consenting of participants, data collection (anonymous or identifiable), gaining access to identifiable data, and/or conducting analysis of identifiable data. These training requirements will apply to all Principal Investigators, Co-Investigators, and research staff/students. This includes both individuals within the TTU system and external collaborators.

This requirement will go into effect January 1, 2023. For more information visit our webpage.

Effective January 1, 2023, TTU research personnel must complete one of the following trainings every 3 years:

- CITI - TTU Human Subject Research - Required Basic
- TTU Human Subject Training
- External – CITI Human Subject Training

4-1 **Principal Investigator**

TTU OP 74.09

*required

4-1a **Check the appropriate box.**

✓ TTU full-time or tenured faculty member

*required

4-1a(1) **College**

College of Arts and Sciences

*required

4-1a(2) **Department**

Kinesiology & Sport Management

TTU full-time employee with a terminal degree in their discipline

*required

Find and add PI to the submission.

4-1b Name: Hui Ying Luk
Organization: Kinesiology and Sport Mgmt
Address: 2500 Broadway MS3011, Lubbock, TX 79409
Phone: 8068340827
Email: huiying.luk@ttu.edu

4-1a(3)a Attach human subject training.

Luk_CITI.pdf

Primary Contact

4-2

Primary contacts need to be included in section 4.3 as a co-investigator if they are not the principal investigator. The primary contact and principal investigator will receive all notifications and letters.

*required

4-2a **Find and add primary contact to the submission.**

Name: Danielle Levitt-Budnar
Organization: Kinesiology and Sport Mgmt
Address: 2500 Broadway MS3011, Lubbock, TX 79409
Phone: 8068341830
Email: Danielle.Levitt@ttu.edu

Name: Hui Ying Luk
Organization: Kinesiology and Sport Mgmt
Address: 2500 Broadway MS3011, Lubbock, TX 79409
Phone: 8068340827
Email: huiying.luk@ttu.edu

4-3 **Co-Investigators**

Include investigators assisting with instrument development, recruitment, data collection, and data analysis.

*required

4-3a **Select all investigators involved with this research project.**

None

✓ List TTU Faculty or Staff Co-Investigator(s):

*required

Multiple names are allowed.

Name: Fangyuan Zhang
Organization: Mathematics and Statistics
Address: 2500 Broadway MS1042, Lubbock, TX 79409
Phone: 8068342587
Email: fangyuan.zhang@ttu.edu

Name: Heather Vellers
Organization: Kinesiology and Sport Mgmt
Address: 2500 Broadway MS43011, Lubbock, TX 79409-3011
Phone: 8068348554
Email: heather.l.vellers@ttu.edu

4-3a(1) Name: Danielle Levitt-Budnar
Organization: Kinesiology and Sport Mgmt
Address: 2500 Broadway MS3011, Lubbock, TX 79409
Phone: 8068341830
Email: Danielle.Levitt@ttu.edu

Name: Terrell Hilliard
Organization: Kinesiology and Sport Mgmt
Address: 2500 Broadway Ms 3011, Lubbock, TX 79409
Phone: 8068345585
Email: terhilli@ttu.edu

Name: Jaden Hendrix
Organization: Kinesiology and Sport Mgmt
Address: , Lubbock, TX 79409-1035
Phone: 8067423371
Email: jadhendr@ttu.edu

Name: Benjamin Barr
Organization: Kinesiology and Sport Mgmt
Address: 2500 Broadway MS 3131, Lubbock, TX 79409-3131
Phone: 8067423371
Email: Benjamin.Barr@ttu.edu

Attach human subject training.

4-3a(1)(a)

[Terrell_ Human Subject Research.pdf]

[Jaden_Human_Subject_JH_7.24.pdf]

[TTU Human Subject Research - CITI.pdf]

✓ List TTU Graduate or Undergraduate Co-Investigator(s):

4-3a(2) *Multiple names and selections are allowed.*

✓ Graduate Student(s)

*required

Name: Casey Appell
Organization: Kinesiology and Sport Mgmt
Address: , Lubbock, TX 79409-1035
Phone:
Email: Casey.Appell@ttu.edu

Name: Parisa Foroozan
Organization: Kinesiology and Sport Mgmt
4-3a(2)a Address: , Lubbock, TX 79409-1035

Phone:
Email: parisa.foroozan@ttu.edu

Name: Masoumeh Rasoli
Organization: Kinesiology and Sport Mgmt
Address: , Lubbock, TX 79409-1035
Phone:
Email: mrasoli@ttu.edu

4-3a(2)a1 Attach human subject training.

---

*required

4-3a(2)b **The purpose of this study:**

Doctoral Dissertation

Master's Thesis

Research conducted as a class assignment with the intention of publishing results

Class assignment requiring IRB submission with no intention of publishing results

✓ Students are members of the research team

Other

✓ Undergraduate Student(s)

*required

4-3a(2)c

Name: Ashley Williams
Organization: Kinesiology and Sport Mgmt
Address: , Lubbock, TX 79409-1035
Phone:
Email: wil98200@ttu.edu

Name: Matthew DiLeo
Organization: Kinesiology and Sport Mgmt
Address: , Lubbock, TX 79409-1035
Phone:
Email: madileo@ttu.edu

Name: Connor Mills
Organization: Kinesiology and Sport Mgmt
Address: , Lubbock, TX 79409-1035

Phone:
Email: mil67126@ttu.edu

Name: Ernan Carrasco
Organization: Kinesiology and Sport Mgmt
Address: , Lubbock, TX 79409-1035
Phone:
Email: erncarra@ttu.edu

4-3a(2)c1 Attach human subject training.

*required

4-3a(2)d **The purpose of this study:**

Undergraduate Research

✓ Students are members of the research team

Research conducted as a class assignment with the intention of publishing results

Class assignment requiring IRB submission with no intention of publishing results

Other

✓ External Researchers: Co-investigators outside of Texas Tech University

*required

List non-TTU investigator(s) engaged in this research. Provide their contact information, title, and place of work.

Robyn Richmond, MD
Assistant Professor of Surgery
Associate Program Director, General Surgery Residency
Trauma, Surgical Critical Care, Acute Care Surgery
Department of Surgery
Texas Tech University Health Sciences Center
robyn.richmond@ttuhsc.edu
972 987 9616

4-3a(3) Leslie Shen, Ph.D.
Associate Dean for Research & Professor of Pathology

School of Medicine
Department of Pathology
Texas Tech University Health Sciences Center
leslie.shen@ttuhsc.edu

Rama Chemitiganti, MD
ECHD Endowed Chair of Medicine
Director, Center of Excellence for Diabetes and Endocrinology
School of Medicine
Department of Internal Medicine
Texas Tech University Health Sciences Center at Permian Basin
rama.chemitiganti@ttuhsc.edu
806-743-3280

*required

4-3a(3)a **Check all that apply.**

Developing instrument

Recruiting participants

Interaction with participants

Working with de-identified data

Working with identifiable data

Assisting with writing manuscript

✓ Other

*required

Include any information that helps to explain their role with the research team.

4-3a(3)a1

Dr. Richmond will provide medical-related oversight/consultation on the muscle biopsy procedures. There will be no direct interaction between Dr. Richmond and the participants.

Dr. Shen will provide consultation on participant recruitment.

Dr. Chemitiganti is the NIH-approved Safety Officer for this study. There will be no direct interaction between Dr. Chemitiganti and the participants.

Attach human subject training.

5: Funding

Complete the following section.

5-1 **Funding**

Funding is used to supplement the cost of the research and/or participant payment.

*required

5-1a **Select the type of funding to be used.**

✓ External (Office of Research Services - ORS)

*required

5-1a(1) **Cayuse SP - Sponsored Project Number (Example: 16-0123)**

23-0477

*required

5-1a(2) **Sponsored Project Title**

Glycemic control and frailty risk in older people at risk for type 2 diabetes: Impact of local heat therapy

*required

5-1a(3) **Find and add the Sponsor's name.**

Name

-

- Name - A to Z
- Name - Z to A

NIH - National Institute on Aging

*required

5-1a(4) **Attach a copy of the sponsored project that was submitted to the funding agency.**

For example: scope of work, abstract, research description.

FINAL_ESI R01_HYLDEL_Specific aims.docx

FINAL_ESI R01_HYLDEL_Research Strategy.docx

Internal (TTU Funding)

Personal Monies

No Funding

5-2 **Future Funding**

If funding is secured, a modification will be required.

5-2a **Enter the potential sponsor(s) name below.**

6: Rationale

Please respond to the following statements to help our IRB reviewers understand your research project.

6-1 Research Objective

*required

Briefly summarize the relevant background information.

Frailty-related medical expenses cost approximately $18 billion annually in the USA. Progressive losses in skeletal muscle (SKM) mass and function, often observed with aging and type 2 diabetes (T2D), contribute to phenotypic frailty characterized by slow gait speed, weakness, weight loss, fatigue, and low physical activity. T2D increases frailty risk by nearly 50% and risk for both conditions increases with age; therefore, preventing progression to T2D in older adults with prediabetes is critical. Additionally, SKM is the largest glucose disposal site in the body and insulin responsiveness, a component of glycemic control, is essential to maintain functional SKM mass. Thus, impaired glycemic control, a pathophysiological change underlying the development of T2D and observed in prediabetes, increases frailty risk. Conversely, improving capillarization and mitochondrial function support increased SKM mass and glycemic control, thereby decreasing T2D and frailty risk. Together, these SKM architectural variables (e.g., cross-sectional area [CSA], capillarization, mitochondria) are attractive targets for interventions such as exercise in prediabetic older people. High-intensity interval training (HIIT) has been used effectively in older adults with SKM benefits similar to those from aerobic and resistance training, and is more time-efficient. However, prediabetic older people may be exercise-intolerant or -resistant, underscoring the need for alternative therapies in place of (or in addition to) exercise. Emerging evidence supports repeated heat therapy as an alternative method to improve glycemic control and SKM architecture, and such adaptations may also improve muscle growth responses to subsequent exercise. While whole-body heat therapy is widely studied, local heat therapy (e.g., heat pad) is more practical and likely provides similar health benefits. Whether local heat therapy would have similar benefits to HIIT or whether heat pre-conditioning would improve adaptations to subsequent HIIT in prediabetic older adults is unknown.

6-2 **Importance of Research**

*required

Explain the scientific importance of the knowledge to be obtained as a result of this research.

Exercise is a first-line intervention for improving glycemic control and decreasing frailty risk due largely to adaptations in SKM. While exercise has a host of whole-body benefits, older individuals in general and those at risk for T2D (i.e., prediabetic) in particular may be exercise-resistant or -intolerant. Therefore, there is an urgent need for **efficacious and practical interventions** to improve muscle health in aging people to decrease their risk of developing T2D and frailty. The proposed project is innovative because we will:

- Use local heat therapy as an *easily implemented intervention* to improve the fundamental aspects of muscle health in aging prediabetic people, a novel application of this widely-used therapy
- Compare the efficacy of our proposed intervention alone and as a means of *preconditioning muscle* for improved exercise adaptations

Finally, this project will provide essential fundamental evidence to lay the groundwork for a subsequent clinical trial that examines the use of this technique in aging people with other SKM-related comorbidities. This work has the potential to **shift clinical practice** by providing direct evidence that supports a low-cost, practical intervention to improve metabolic health in at-risk individuals.

6-3 **Research Questions/Specific Aims**

*required

List the research question(s)/specific aims to be addressed.

The overarching hypothesis of this study is that local heat therapy improves muscle architecture, glycemic control, and subsequent exercise adaptations, and decreases frailty risk in prediabetic older adults, with TRPV1 as an underlying mechanism [mechanistic experiments will be in Aim 3, not part of this IRB application]. We will test this hypothesis through the following specific aims:

Aim 1: Test the hypothesis that local heat therapy improves muscle architecture, glucose tolerance, and frailty indicators similarly to HIIT in older prediabetic people.

Aim 2: Test the hypothesis that local heat therapy pre-conditioning improves the skeletal muscle response to HIIT in older prediabetic people.

6-5 Citations

Enter or attach the references cited .

The references below are from our Specific Aims and Research Strategy documents. Although they are not cited in the project summary, importance, and aim above, they are cited in the funding application.

ESI R01_Bibliography & References Cited.docx

Please respond to the following statements/questions to help our IRB reviewers know more about the participants in this study.

7-1 **Relationship with Participants**

*required

7-1a **Is there the potential for any relationship between the researcher(s) and the proposed target population?**

(Note: if recruiting in the Lubbock (which includes TTU) community please click yes as students and employees are possible)

✔ Yes

*required

7-1a(1) **Explain**

Recruiting older adults with prediabetes in Lubbock; it is possible that other TTU employees could be recruited.

No

Coercion or Undue Influence

Guidance for IRB and Investigators

Coercion - Coercion entails influencing an individual's decision about whether or not to

7-2 do something by using explicit or implied threats (loss of good standing in a job, poor grades, etc.). This area also includes the perception of students as to any potential backlash for failing to take part in an instructor's research.

Undue Influence - This is defined as an offer of an excessive, unwarranted, inappropriate, or improper reward or other overture in order to obtain compliance (used in association with recruiting research participants).

*required

7-2a **Does this research involve participants who could be coerced or unduly influenced to participate or may feel that there is perceived coercion or undue influence (i.e., students, employees, prisoners, colleagues, friends/acquaintances, family members, etc.)?**

✓ Yes

*required

Describe the plan to mitigate (perceived) coercion or undue influence to participants.

7-2a(1) One example of mitigating coercion/undue influence would be to add a sentence to the consent form that clearly states their decision to participate, not participate, or withdraw in the research will not impact their employment status or academic standing.

We will emphasize to participants that their enrollment and continued participation is completely up to them. They can withdraw from the study at any time for any reason without consequences. The PIs of this study do not currently have any influence over the employment of individuals who would qualify for this study. Any future non-traditional student who might qualify will be assured that their grade(s) will noty be impacted, positively or negatively, by their participation or withdrawal. Colleagues in KSM who might qualify and enroll will be assured that our working relationships will not be impacted, positively or negatively, by their participation or withdrawal. The PIs are committed to ensuring that participation and/or withdrawal of KSM colleagues who might choose to participate and/or withdrawal does not impact our decisions for any future faculty votes, recommendations, etc. Finally, we will emphasize the importance of confiidentiality with our research team, **especially** when a participant might be a TTU (including KSM) employee.

No

7-3 **Target Population**

*required

7-3a **Describe the population of human subjects to be recruited and why this research specifically targets this group?**

In this study, participants with elevated blood glucose, prediabetes, or are at risk for developing diabetes; are sedentary will be recruited.

This population is targeted because the muscular pathophysiology of T2D (i.e., insulin resistance [IR], decreased skeletal muscle [SKM] mass and function) contributes to phenotypic frailty including slow gait speed, weakness, unintentional weight loss, fatigue, and low physical activity. Approximately 7-15% of community-dwelling older adults meet criteria for frailty, and 25-40% of these individuals are prediabetic. Moreover, a recent meta-analysis of community-based studies found that baseline T2D is associated with nearly 50% increased risk of frailty development.

Although exercise training is a key technique to manage metabolic disease and decrease T2D risk, in older adults at risk for T2D, compromised oxygen delivery and mitochondrial dysfunction result in exercise intolerance, a potential contributor to low exercise adherence. Thus, it is critical to find alternative means to manage metabolic disease risk. Heat therapy is one possible alternative. Heat therapy adaptations could improve exercise tolerance by preconditioning the microenvironment of skeletal muscle, likely enhancing exercise adherence and improving subsequent exercise adaptations.

*required

7-3b **Does your target population require specific characteristics?**

Yes, my target population **DOES** require specific characteristics.

✓ No, my target population **DOES NOT** require specific characteristics.

*required

7-3c **Age Range**

Check all that apply.

✓ Adults

*required

7-3c(1) **Enter the age range.**

≥60

Children

*required

7-3d **Enter the number of participants.**

27 men and 27 women

7-4 **Inclusion/Exclusion Criteria**

Enter or attach all criteria used to include or exclude participants for this research.

Individuals with elevated blood glucose, prediabetes, or are at risk for developing diabetes; are sedentary

Inclusion:
•Sedentary (structured exercise <30 minutes, 3x/week)
•Meet criteria for prediabetes (fasting blood glucose 100-125 mg/dl and/or hemoglobin A1c 5.7-6.4%)
•Age ≥ 60 years
• Body weight is at least 110 pounds
•Consume <8 (women) or <15 (men) alcohol-containing beverages per week
•Do not use nicotine or cannabis

•Not taking any medications that could interfere with responses to the interventions (e.g., corticosteroids, opiates, benzodiazepines, tricyclic antidepressants, beta blockers, sulfonylureas, insulin, metformin, anticoagulants, barbiturates, insulin sensitizers, fibrates [PPAR gamma agonist], immunosuppressants)

Exclusion:
•History of peripheral neuropathies
•Currently taking prescription blood thinners
7-4a •Medical complications that could would contraindicate participation in the high intensity interval training (HIIT) intervention including: orthopedic complications that would limit your ability to perform cycling exercise, significant cardiovascular impairments (e.g., history of arrhythmias, severe uncontrolled hypertension, etc.), diagnosed metabolic disease (e.g., diabetes), renal disease, sickle cell anemia, or cancer in remission for <6 months.
•Known history of slow wound healing
•Excessive subcutaneous fat over the vastus lateralis (greater than 1.5")
• Have experienced symptoms suggestive of cardiovascular, respiratory, metabolic, or renal diseases including discomfort, pressure, or pain in your chest, neck, jaw, arms, calves, or other areas potentially related to ischemia; shortness of breath at rest or with mild exertion; dizziness or fainting (syncope); difficulty breathing while lying flat (orthopnea) or sudden nighttime breathing difficulties (paroxysmal nocturnal dyspnea); palpitations or rapid heartbeat (tachycardia); pain or cramping in your legs during physical activity (intermittent claudication); a known heart murmur; swelling in your ankles (edema); unusual fatigue or shortness of breath during routine activities or at rest.
• Currently pregnant
• Lidocaine allergy
• Latex allergy

We will screen for these criteria using the medical history form (attached). After inquiring with the participant, "Yes" to the following questions would result in exclusion from the study: 1, 3-7, 9-12, 15-17 (unless acute illness), 19 (lidocaine only), 20 and 23. Answers to other questions will be compared against inclusion/exclusion criteria (i.e., injuries, illnesses, chronic conditions, other reason that they should not exercise, etc.) to determine whether the participant meets all inclusion and no exclusion criteria. A key for research staff is included as a separate page in the form (beginning in Column Z on the spreadsheet).

7-4a(1) Inclusion/Exclusion Screening Form (if applicable)

R4_Feb 3_IM_HEAT study_Medical History Questionnaire.xlsx

7-5 **End of Subject Participation**

*required

7-5a **Enter specific criteria used by the researchers to prematurely end a subject's participation in the study.**

Sometimes a PI will decide to remove a participant from a research study. List any reasons you would anticipate that may require this removal.

This is not a participant deciding they would like to stop/withdraw from the research. This is a researcher ending their participation. For example: unforeseen risk to the participants, risks to the other participants/research team members, non-adherence, disruptive behavior.

The researchers will end participants' involvement in the study early if they do not follow the study's instructions, which include:

- Adopting a new lifestyle
- Starting a new exercise routine and/or diet program
- Taking any new supplements
- New medical conditions or complications that would place participants at increased risk
- Low adherence to interventions (<80% compliance)

7-6 **Investigator Assurances**

*required

7-6a **Data Storage and Retention**

✓ I will provide participants with information about data storage and retention if they withdraw from the study, when contacted by subjects per contact information provided in the consent form.

I will **NOT** provide participants with information about data storage and retention if they withdraw from the study, when contacted by subjects per contact information provided in the consent form.

*required

7-6b **Participant Withdraw**

✓ I will provide participants with information about how to withdraw from the study through the provided consent form.

I will **NOT** provide participants with information about how to withdraw from the study through the provided consent form.

Please respond to the following statements/questions.

8-1 **Recruitment Procedures**

*required

Describe the process of how participants will be recruited and provide the sequence of these events.

This section needs to include the details of each step for recruitment up to the point of consent. This includes pre-screening procedures.

All planned recruitment efforts and scripts will be approved by the Texas Tech University IRB. We will begin recruitment through the University Medical Center in partnership with Texas Tech University Health Sciences Center physicians who see patients from the community that may qualify. Email addresses and/or phone numbers will be obtained by searching for internal medicine, family medicine, geriatrics, and endocrinology clinics. Specially, fliers will be placed in the clinic and provided to physicians to distribute to potential participants.

8-1a Electronic advertisements will be posted on listservs (e.g., TechAnnounce, Obesity Research Institute listserv) and on social media (e.g., Facebook). Social media announcements will specify targeting older adults (60+) in the Lubbock, TX area. Additionally, after site-specific approval, flyers will be distributed around the community to frequently visited locations such as grocery stores, pharmacies, and community centers.

Drs. Luk and Levitt will be responsible for executing recruitment efforts, collecting contact information for potential participants, conducting initial phone screenings, and following up with potential participants to schedule them for their informed consent and screening visit. Once a clinical coordinator is hired, these responsibilities will primarily shift to that person. The initial phone screening is to ensure participant is at least 60 years old, sedentary, and their physician has informed them they have elevated blood glucose, prediabetes, or are at risk for developing diabetes.

8-2 **Recruitment Methods**

[Templates]

*required

8-2a **Select all methods of recruitment.**

Check all that apply.

No Recruitment

✓ Oral Script

*required

8-2a(2) **Enter or attach the Oral Script(s).**

If more than one oral script is entered or attached, label each script separately.

Script for UMC and TTUHSC phone recruitment is attached

8-2a(2)a If you attach a document, state *See Attachment* in the text box above.

R4_Feb 4_Script_UMC and TTUHSC recruitment.docx

✓ Tech Announce

*required

8-2a(3) **Enter or attach TechAnnounce ad.**

See attached

8-2a(3)a If you attach a document, state *See Attachment* in the text box above.

R5_March 5_IM_Tech Announce Ad - HEAT study (2) (3).docx

✓ Recruiting Letter(s) or Email(s)

*required

8-2a(4) **Explain how email addresses or physical addresses of potential participants are obtained.**

We will search for any email addresses for UMC or TTUHSC physicians' offices.

*required

8-2a(5) **Enter or attach all recruiting emails or letters.**

If more than one email is entered or attached, label each separately.

Script attached

8-2a(5)a If you attach a document, state *See Attachment* in the text box above.

R4_Feb 4_Script_UMC and TTUHSC recruitment.docx

Information Sheet

✓ Advertisement(s)

*required

8-2a(8) **Check all that apply.**

✓ Print Advertisement (eg. newspaper, magazine, fliers)

Website

✓ Social Media

*required

8-2a(9) **Enter or attach the advertisement.**

If more than one advertisement is entered, label each separately.

The attached flyer will be used for print and social media advertising.

8-2a(9)a If you attach a document, state *See Attachment* in the text box above.

M1_R1_June 11_HEAT Flier (2).doc

Subject/Participant Pools

Third Party Recruitment

Other

8-2b **Investigator Assurances**

*required

8-2b(1) **Permission to Recruit Participants**

✓ I will acquire proper permission prior to recruiting participants.

I will **NOT** acquire proper permission prior to recruiting participants.

8-3 **Permission to Conduct Research**

*required

8-3a **Is this research study being conducted in a setting that requires prior permission from an entity to conduct research on their premises (i.e., school system, business, government entity, etc.)?**

Yes

✓ No

Visit the HRPP website for instructions and templates on the Consent Process, Assent Process with Minors, Short Form Consent Process, and Waivers of Consent.

Consent forms can be destroyed three years after the completion of the research. 45 CFR 46.115(7)(b).

9-1 **Consent**

*required

9-1a **Consent Process & Forms**

Check all that apply.

✓ Consent (Adult)

*required

9-1a(1) **Check all that apply.**

✓ Participant

Legal Authorized Representative Consent (LAR)

Parent/Legal Guardian

*required

Describe the process for obtaining consent. Include where and when the consent process takes place and who will be obtaining consent.

Drs. Luk or Levitt will provide a brief overview of the study using the information sheet and then seek informed consent from all potential participants in their offices in the KSM building to ensure privacy. All participants will be provided with copies of the information sheet and informed consent sheet. They will also be provided with a complete verbal explanation regarding the reasons for conducting the study, the procedures, risks, and benefits of the study, and any questions will be answered. At the end of the consent process, participants will be asked the following questions to establish capacity to consent:

9-1a(2)

Do you understand why this study is being done?
If you decide to participate in the study, what are some of the things you will be asked to do?
Describe some of the risks or discomforts you may experience if you participate in this study.
Will this study help you?
Do you have to be in this study?
What will happen if you decide not to be in the study?
Who should you contact if you have questions or experience a problem while in the study?

Correctly answering these questions will demonstrate an understanding of the study and determine the capacity to provide consent. Adults unable to demonstrate the capacity to consent will not be enrolled in the study.

*required

Enter or attach the consent form.

9-1a(3)

If more than one consent form is entered or attached, label each separately.

See attachment

If you attach a document, state *See Attachment* in the text box above.

9-1a(3)a

DXA Acknowledgement Form (1) (1).pdf

M1_R1_June 11_Informed Consent - HEAT study_Laymen term (4).docx

*required

Will a Short Form Consent be used?

9-1a(4) Only used in complex studies when the PI cannot be sure that a signed written consent is understood well enough to indicate a valid consent process.

Yes

✓ No

Assent (Minors & Individuals with Impaired Decision Making Skills that cannot consent for themselves)

Texas Tech Neuroimaging Institute (TTNI) Consent Form

No consent will be obtained.

9-2 **Waivers**

A consent form must still be included in the documentation.

9-2a **Check all that apply.**

Request for a Waiver of Written Consent

Request for a Waiver or Alteration of the Elements of Consent

FERPA Authorization

IRB approval or determination of exemption of the research project does not constitute institutional permission to access the requested records.

9-3 **Guidance:** FERPA applies to personally identifiable information in educational records. This includes items such as the student's name, names of family members, addresses, personal identifiers such as social security numbers, and personal characteristics or

other information that make the student's identity easily traceable.

Educational records are all records that contain information directly related to a student and are maintained by an educational agency or institution, or by a party acting on its behalf. A record means any information recorded in any way, including handwriting, print, tape, film, microfilm, microfiche, and digital images.

9-3a **Will you be using student records protected under FERPA?**

Yes

✓ No

HIPAA Authorization or Waiver

9-4

Indicate whether you are obtaining Participant Authorization or requesting a Full or Partial Waiver of HIPAA Authorization.

9-4a **Select the appropriate authorization.**

Participant Authorization of Protected Health Information

Full or Partial Waiver of Protected Health Information

Complete the following section.

Data Collection

10-1 IRB members find it very helpful to have copies of all instruments. Reading the instrument(s) is essential to the board's assessment of participant risk and burden and the adequacy of the study description in the informed consent. Occasionally, however, investigators do not include all or full copies of instruments on the grounds that the instrument is copyrighted and in the belief that making copies to submit with an IRB application is a violation of copyright law. However, instrument developers and publishers should have a reasonable expectation that instrument users must obtain IRB approval of their research and that approval requires review of all study materials. Further, IRB members are well aware of their own ethical responsibility to honor copyright and purchase contracts by investigators and to refrain from use of copyrighted material for purposes other than those related to their IRB obligations. If records are being withheld pursuant to a Non-Disclosure Agreement (NDA), you (the investigator) are expected to provide the relevant portion of the NDA or any similar documentation requiring the records be withheld specifically from the IRB. If the NDA or license precludes sharing with the IRB, it is the PI's responsibility to get approval from the company or licensor to share the instrument(s) with TTU's IRB for review.

*required

Describe the sequence of events and timeline the participants will experience.

A randomized controlled intervention design will be used. The study consists of 2 phases, each 12-weeks in duration.

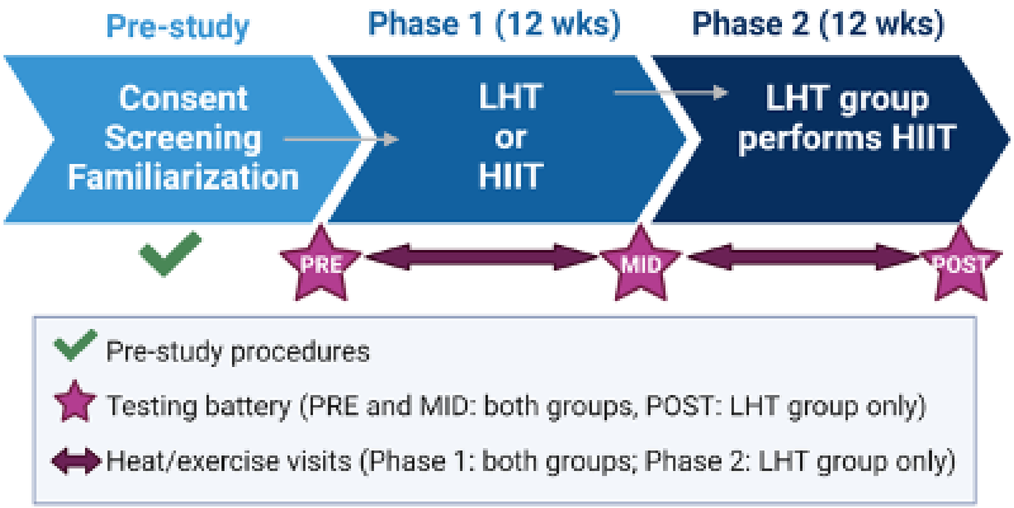

Visit 1: Consent and screening: (~2h)
•Participant will need to fast (i.e., no food or beverages other than water; plain water allowed and encouraged) for 12h before consent and screening.
•After an explanation of study requirements, participant will review and sign the informed consent form.
• Complete the medical history questionnaires
•We will collect a drop of participant blood using finger prick to determine their fasting blood glucose and HbA1c concentrations to make sure they have met the inclusion criteria.
•We will put a small drop of jelly on participant's thigh and use an ultrasound machine to image the participant's vastus lateralis muscle. This will help us make sure that the lidocaine can reach the muscle (i.e., subcutaneous fat over the muscle no greater than 1.5").
• Lastly, you will be familiarized the following set of questionnaires.
Questionnaires 1-6 will be assessed at T1, T2, and T3.
Questionnaires 2-6 will also be assessed weekly during your lab visits.
1.Unintentional weight loss (self-report, ≥10 lbs or ≥5% of body mass in the past year or between major study time points)
2.Fatigue (SF-36 energy subscale)
3.Physical activity (International Physical Activity Questionnaire)
4.24h Diet Recall log (ASA24: Healthy Eating Index 1-100 scale).
5.Sleep quality and well-being (7-point likert scale)
6. Alcohol use (TLFB: Timeline Follow Back)

PRE Testing (Before Phase 1 Begin): All major measurements for the PRE Testing will be completed over 2 separate days within a week. These major measurements will also be conducted at MID (end of Phase 1), and POST (end of Phase 2). However, the sequence of these major measurements will be different.

Preparing for Visits 2 and 3:
1. Overnight fast for 12h (only water allowed)
2. No alcohol for 24h
3. No medications or supplements from this list for 48h:
•Tylenol (acetaminophen)
•Non-steroidal anti-inflammatory drugs (e.g., Ibuprofen)

Visit 2 (Day 1 of the PRE Testing): (~ 4-5h)
1. Hydration assessment: Participant will provide a urine sample so we can assess the hydration status (urine specific gravity, a measure of hydration, must be <1.020). Also, blood sample will be collected for plasma osmolality measurement. In addition, thirst sensation will be assessed using a thirst scale and urine color will be recorded. If participant is not sufficiently hydrated, then they will be given at least 8 oz of water and re-test their hydration after 15 minutes. After 15 minutes, urine sample and thirst sensation will be measured.

2.Anthropometric Measurements:
•We will measure your height, weight, waist-hip ratio, and a body composition scan will be performed using dual X-ray absorptiometry (DXA).

3. Blood pressure:
• We will measure your blood pressure.

4.Muscle Biopsy: We will take muscle sample (~200 mg) from your outer thigh. This procedure will last about 30 minutes.

5.Oral Glucose Tolerance test (OGTT):
• Participants will consume a beverage containing 75 g glucose (TRUTOL, ThermoFisher Scientific).
•Before and at 30 min, 1 hr, and 2 hrs after beverage consumption, venous blood will be collected into vacuum tubes using the standard venipuncture technique.
6. Questionnaires: During the OGTT, you will complete the following set of questionnaires. Questionnaires 2-6 will also be filled out weekly during the study.
1.Unintentional weight loss (self-report, ≥10 lbs or ≥5% of body mass in the past year or between major study time points)
2.Fatigue (SF-36 energy subscale)
3.Physical activity (International Physical Activity Questionnaire)
4.24h Diet Recall (ASA24: Healthy Eating Index 1-100 scale).
5.Sleep quality and well-being (7-point likert scale)
6.Alcohol use (Timeline Follow Back)

Visit 3 (Day 2 of the PRE Testing) (~1.5h)
1. **Hydration assessment:** Participant will provide a urine sample so we can assess the hydration status (urine specific gravity, a measure of hydration, must be <1.020). In addition, thirst sensation will be assessed using a thirst scale and urine color will be recorded. If participant is not sufficiently hydrated, then they will be given at least 8 oz of water and re-test their hydration after 15 minutes. After 15 minutes, urine sample and thirst sensation will be measured.
2. Body weight: Body mass will be measured
3.Physical Function (part of Phenotypic Frailty Assessment) Testing:
•Perform the gait speed test (4-m walk test), 30-second Sit-to-stand test, and handgrip strength test on both hands.
4.Exercise Capacity Test:
•Perform an exercise capacity (VO2peak) test using a cycle ergometer.

Phase 1:

For the Local Heat Therapy Groups

If participants are assigned to one of the local heat therapy(LHT) groups (Sham (CON) and LHT), they will be sent home with 2 heating pads and apply local heat to their thigh 6 days for 12 weeks (5 sessions at home and 1 session at the Lab). Participants assigned to the heating group will be advised to set their heat pads to the temperature previosuly determined to achive an intramuscular temperature increase by 3-4 C; participants assigned to the sham (CON) group will be advised to set their heat pads to a lower tempereature (e.g., 36 C) to maintain a neutral intramuscualr temperature.

Visit 4: Establish heat pad temperature: (1st week of the 12-week LHT intervention)

• During the familiarization visit, we will establish the specific heat pad temperature needed to elevate each participant's intramuscular temperature by 3-4 °C. Intramuscular temperature sensors will be used to calibrate the heat pad to approximately 3-4°C increase over a 90-minute period. For participants in the LHT group, these measurements will determine the optimal heat pad temperature. To reach the target 3-4 C increase in intramuscular temperature, the heat pad temperature will be increased by 5°C every 5 minutes. If the intramuscular temperature rises beyond 4C after resting, the heat pad temperature will be decreased to maintain the intramuscular temperature by 3-4°C above resting temerpature. After the 90-minute heating session, sensors will remain in the muscle for an additional 60 minutes to monitor temperature changes.

• To monitor skin temperature, one temperature sensor will be placed on the skin approximately 5 cm below the site of the intramuscular temperature sensor to record skin temperature. A second skin temperature sensor will be placed on the contralateral leg in a corresponding location. After securing the thermocouple, a commercially available heat pad will be positioned to surround each of the thighs. To minimize the risk of heat-related skin discomfort or burns, a size-matched flannel cloth will be inserted into the heat pad sleeve as a protective barrier. To ensure secure placement and consistent surface contact, two Velcro straps will be used to secure the heat pad firmly around the upper and lower portion of the thigh.

To measure the intramuscular temperature using intramuscular temperature probe, we will utilize the following published method (Richey et al., 2024). The PIs will receive training from consultant Dr. Steven Romero to ensure accurate technique and proper care throughout the following procedure.

1. The opposite leg from the biopsy site will be used to measure intramuscular temperature.
2. Apply lidocaine under the skin and into the muscle.
3. Put a sterile catheter or sterile 18G needle into the midpoint of the vastus lateralis, then remove the needle from the catheter.

10-1a 4. Insert the thermocouple (IT-18; Physitemp: Type T thermocouple; temperature range: -100 to +150 C/-148F to +300F) through the needle or catheter.

5. Position the thermocouple approximately 1 cm deep into the muscle.
6. Carefully retract the needle or catheter to ensure the thermocouple is correctly positioned

within the muscle.
7. Cover the area with skin-safe adhesive tape (Tegaderm Film) to ensure everything stays in place.
8. Apply heat pad on the thigh for 90 min.
9. Then remove for up to 60 additional minutes (or until the intramuscular temperature returns to baseline, whichever is first).
10. Muscle temperature and body temperature (forehead scanning) will be monitored throughout.
11. Blood pressure will be measured every 10 minutes throughout the 90-min heat pad application.
12. A bandage will be placed on the site of the incision.

o If the intramuscular temperature is above 4°C increase, we will decrease the heat pad temperature by an increment of 1°C until the intramuscular temperature stabilizes at a 4°C. If the skin temperature exceeds 41.5°C, the heat pad temperature will also be reduced by 1°C, and the pad will be loosened to increase airflow and reduce surface heating.

5. Once the intramuscular temperature has increased by 3-4°C above resting, the heat pad temperature will be set and temperature (skin and intramuscular temperatures) will be monitored for the remainder of the session.

o *Note:* We are aiming for an intramuscular temperature increased by 3-4°C. We anticipate that this will be well-tolerated because 1) core temperature remains stable during local heating on the thigh and 2) intramuscular temperatures increased by 3-4°C (vastus lateralis) have been documented in human participants without adverse effects. However, if a participant is unable to tolerate this, we will reduce the initial target intramuscular temperature as needed to a minimum of 38°C based on intramuscular adaptations previously documented in the literature.

At home Local Heat Therapy Sessions (5d/wk):
• Participants will be encouraged to empty their bladder before the start of each session to minimize the number of times they have to remove the heat pads during a session. For any reason they have to remove the heat pad, they will record it.

•For each session, participant will place the heating pads to completely surround each thigh with a snug fit. The heating pads will be secured in place using Velcro and set to the predetermined temperature continuously for 90 minutes.

• Participants will record the following for each session:
1. Start time
2. End time
3. Number of times you need to remove the heat pad during a session
4. The duration for which it is removed
5. Discomfort

•There is no restriction on the location (at home or in the office), time of day, and seated activity during the session (e.g., watching TV, reading a book, etc.).

In-Lab Local Heat Therapy Sessions (1d/wk): Visit 5-15 (~2h each) (1d/wk for the remaining 11 weeks of the 12-week LHT intervention)

•During the weekly lab visit (1d/wk), comfort will be monitored, and we will collect the logs of participants' at-home sessions to confirm compliance.
• Blood pressure will be measured every 10 minutes while the heating pad is applied.
• The following will also be assessed each week: sleep quality and activity (Hooper questionnaire and International Physical Activity Questionnaire [IPAQ]), alcohol use (Timeline Follow-back), 24-hr diet recall (ASA24), and anthropometrics (height, body mass, waist circumference, and hip circumference)
• At weeks 6 and 12 of Phase 1, we will measure both intramuscular and skin temperatures during the in-lab heat pad therapy session.

For the High_Intensity Training (HIIT) Groups

In Lab HIIT Session: Visit 4-39 (Total of 36 visits) (~1h each)

•Participants assigned to the HIIT group will arrive at the laboratory 3 d/wk for 12 weeks. They will be fitted with a heart rate monitor (Polar) and skin temperature sensors (thigh) and will perform a 3-minute cycling warm-up at 30W before beginning the HIIT protocol.
•On the first day of each training week, the following will also be assessed each week: sleep quality and activity (Hooper questionnaire and International Physical Activity Questionnaire [IPAQ]), alcohol use (Timeline Follow-back), 24-hr diet recall (ASA24), and anthropometrics (height, body mass, waist circumference, and hip circumference)
• At weeks 1, 6, and 12 of Phase 1, we will measure skin temperatures during the HIIT session.

MID Testing (End of Phase 1):

The final LHT or HIIT session must be completed 2 days before MID testing.
All major measurements for the MID Testing is identical to PRE Testing including the order of measurements. Detail protocols, please refer to PRE Testing.

Preparing for the following Visits:
1. Overnight fast for 12h (only water allowed)
2. No alcohol for 24h
3. No medications or supplements from this list for 48h:
•Tylenol (acetaminophen)
•Non-steroidal anti-inflammatory drugs (e.g., Ibuprofen)

Visit 16 (LHT)/ Visit 40 (HIIT): Day 1 MID Testing (~4-5h)

1. Hydration assessment
2. Anthropometric measurements
3. Blood Pressure
4. Muscle Biopsy
5. OGTT
6. Questionnaires

Visit 17 (LHT)/ Visit 41 (HIIT): Day 2 MID Testing (~ 1.5h)

1. Hydration assessment
2. Body weight
3. Physical functional tests
4. Exercise capacity test

Phase 2:
Visit 18 - 53 - Phase 2 (Sham (CON) and LHT groups): Participants assigned to the Sham (CON) and LHT groups will continue to Phase 2. In Phase 2, they will complete HIIT 3d/wk for 12 weeks. For detailed protocols, please refer to above (HIIT group).

POST Testing (End of Phase 2):
All major measurements and order of measurements for the POST Testing is identical to PRE and MID Testing. Detail protocols, please refer to PRE Testing.

The final LHT session must be completed 2 days before POST testing.
All major measurements for the MID Testing is identical to PRE Testing including the order of measurements. Detail protocols, please refer to PRE Testing.

Preparing for the following Visits:
1. Overnight fast for 12h (only water allowed)
2. No alcohol for 24h
3. No medications or supplements from this list for 48h:
•Tylenol (acetaminophen)
•Non-steroidal anti-inflammatory drugs (e.g., Ibuprofen)

Visit 54 (LHT): Day 1 POST Testing (~4-5h)
1. Hydration assessment
2. Anthropometric measurements
3. Blood Pressure
4. Muscle Biopsy
5. OGTT
6. Questionnaires

Visit 55 (LHT): Day 2 POST Testing (~ 1.5h)
1. Hydration assessment
2. Body weight
3. Physical functional tests
4. Exercise capacity test

*For Sham (CON) and LHT groups, participants will have a total of **55 Lab visits** with a total time spent for about **81.5h (Lab)** plus **90h (at home heat pad therapy)**.*

*For the HIIT group, participants will have a total of **41 Lab visits** with a total time spent for about **50h**.*

*required

What is the total time commitment for the participant?

10-1a(1) For CON (Sham, considered under the "LHT groups" in the consent form) or true LHT group, the duration will be approximately 7 months (testing: 1 month total; Phase 1: 3 months, Phase 2: 3 months) with a total of about 81.5h (lab) plus 90h (at home heat pad therapy). For HIIT group, the participation duration will last for approximately 4 months (testing: 3 wks total; Phase 1: 3 months) with a total of about 50h.

*required

10-1b **Select procedure(s) to be used.**

Check all that apply.

✓ Surveys

*required

10-1b(1) **Check all that apply.**

Online

✓ On-Site

Mailing

Other

*required

10-1b(2) **How much participant time is involved?**

The time commitment for surveys is included in the overall study visit time.

*required

Enter or attach the survey(s) questions or a list of survey items.

If multiple surveys are used, label them separately.

Hooper well-being survey questions (completed weekly; scored on 1-7 scale, low (fatigue, stress, soreness) or good (sleep quality) and 7 indicates high (fa soreness) or bad (sleep quality):

1. Please rate your fatigue level over the past 24 hours.
2. Please rate your level of stress over the past 24 hours.

3. Please rate your level of soreness over the past 24 hours.
4. Please rate the quality of sleep you had last night.

10-1b(3) Automated Self-Administered 24-hour Dietary Assessment Tool (ASA-24; cor
Registration for use of the ASA-24 will be completed upon Notice of Award ar
monies. A link to a demonstration website for the ASA-24 is here: https://asa2
(2024 version)

Unintentional weight loss (at PRE, MID, and POST testing): In the past year [
12 weeks [MID and POST], have you unintentionally lost body weight? If so, h

Other surveys: see attachments.
1) Medical history (screening visit)
2) International Physical Activity Questionnaire (IPAQ)-SF (weekly)
3) Timeline Follow Back (TLFB, weekly)
4) 36-item Short Form Health Survey (SF-36) energy subscale (weekly; only i
and 31;
scored per https://www.rand.org/health-care/surveys_tools/mos/36-item-short

If you attach a document, state *See Attachment* in the text box above.

10-1b(3)a

IPAQ-SF.pdf

TLFB_Alcoholinstructions.pdf

TLFB_ALoverview.docx

SF-36.pdf

Thirst Scale.docx

urine color chart.jpg

R1_11.19_IM_Detail the intervention procedures.docx

*required

Describe the process for the distribution and return of surveys.

10-1b(4) Surveys questions will be uploaded into Research Electronic Data Capture (REDCap), a free, secure, web-based application designed to support data capture. Researchers will assist particiants to electronically complete surveys during their laboratory visits.

Interviews

Focus Groups

Observation

Non-Invasive Procedures (MRI, X-Rays, etc.)
Invasive Medical Procedures (Blood draws, skin pricks, biopsies, etc.)

✓ Intervention

*required

10-1b(25) **How much participant time is involved?**

LHT groups ~81.5h (lab) plus 90h (at home heat therapy) and HIIT group ~50h in the laboratory

*required

10-1b(26) **Where will the intervention(s) take place?**

At home (Sham (CON) and LHT groups) and KSM building (all groups)

*required

Describe in detail the intervention procedures.

10-1b(27)

Attach any documentation if applicable.

See attachment. The LHT group described in the Informed Consent document includes LHT (heat) and CON (control; sham - low-temperature heat pads).

10-1b(27)a If you attach a document, state *See Attachment* in the text box above.

*required

Who will be conducting the intervention(s)? What are their qualifications?

Attach any documentation if applicable.

For Oral Glucose Tolerance Testing (OGTT): Drs. Luk and Levitt are experienced in phlebotomy; Dr. Levitt has carried out the OGTT procedure. Additional study team members are trained in phlebotomy (Casey Appell) and will help carry out this procedure under the supervision of Drs. Luk and Levitt. Jaden Hendrix (research aide) will be primarily responsible for processing biospecimens. Drs. Barr and Hilliard, and the listed graduate and undergraduate students will assist in these procedures as needed.

For body composition measurement, Drs. Luk and Levitt are certified DEXA technicians in the State of Texas and are approved to operate the DEXA housed in the Department of Kinesiology & Sport Management (KSM) at Texas Tech University (TTU). Their students trained on use of the DEXA will carry out this procedure under their supervision. Drs. Barr and Hilliard, and the listed graduate and undergraduate students will assist in this procedure as needed.

10-1b(28)

All biopsy procedures will be performed by Dr. Hui-Ying Luk, who is not a licensed physician but is trained specifically in the muscle biopsy procedure. Robyn Richmond, M.D. (robyn.richmond@ttuhsc.edu) will oversee the procedure (i.e., approve safety protocols, provide technical and medical advice regarding the procedures and potential adverse effects of biopsy). Dr. Richmond will determine the competency of the person who will perform the preparation (sterilized procedures, lidocaine injection) and the operation (maintaining sterile procedure for the primary operator: Dr. Luk). Since the assistant (applying suction through a syringe) does not have direct contact with the participant and does not require sterile procedures, the competency of the assistant will be determined by Dr. Luk. Our members are not going to diagnose these symptoms, instead, if the participant is self-reporting pain, erythema (warmth), or ecchymosis (bruising) to us, we will advise them to use a cold pack. Also, our members will follow up with them regarding the self-reported signs. Similarly, if the participant is seeing and self-reporting signs of infection, inflammation, hematoma (e.g., prolonged redness, pus formation, increasing pain, bruising at the site of biopsy, and fever, etc.), and numbness at the site of biopsy, we will refer them to consult with a medical provider. Lastly, an individual with CPR training (current) will be on-site within close proximity to the subject during procedures.

All intramuscular temperature measurement will be performed by Drs. Hui Ying Luk and Danielle Levitt who are trained specifically in this procedure by Dr. Steven Romero. The proposed technique has been safely and successfully implemented in participants (e.g., PMID: 38059292). Drs. Barr and Hilliard and Casey Appell will assist in this procedure as needed.

For exercise testing and intervention, Drs. Luk and Levitt have experience in exercise testing and intervention and are Certified Strength and Conditioning Specialists (CSCS) through the National Strength and Conditioning Association (NSCA). The proposed exercise capacity testing and HIIT intervention have been safely and successfully implemented in older adult participants (e.g. PMID: 29077855, 28273480). Dr. Luk and Levitt will train and supervise all personnel (graduate and undergraduate students on this IRB application) in conducting the exercise testing and intervention included herein. Only study personnel with currect CPR and first aid training will perform exercise testing and intervention. Dr. Levitt has an up-to-date CPR and first aid certification, and has held valid CPR

certification continuously since 2003. Drs. Barr and Hilliard, and the listed graduate and undergraduate students will assist in these procedures as needed.

For frailty risk assessment, Dr. Levitt has assessed frailty risk in people living with HIV. She will train and supervise all personnel (graduate and undergraduate students on this IRB application) in conducting the physical function testing to assess frailty risk. Drs. Barr and Hilliard, and the listed graduate and undergraduate students will assist in these procedures as needed.

If you attach a document, state *See Attachment* in the text box above.

10-1b(28)a

incidents updated (1) (2) (2) (2) (1).docx

IM temp and heat therapy in older adults.docx

Other

Deception

10-2

The result of investigators providing false or incomplete information to participants for the purpose of misleading research subjects.

*required

10-2a **Does this research project involve the use of deception?**

✓ Yes

*required

10-2a(1) **Explain why deception is necessary.**

A sham group is needed for scientific purposes.

No

10-3 **Debriefing**

10-3a **Will a debriefing script be used at the conclusion of the research?**

✓ Yes

*required

10-3a(1) **Select the reason for needing a debriefing script.**

✓ Deception

Sexual Misconduct

Mental Health, Self-harm, Substance Abuse, Criminal Behavior

✓ Education Tool

*required

10-3a(2) **Enter or attach a copy of the debriefing script.**

See attachment

If you attach a document, state *See Attachment* in the text box above.

10-3a(2)a Biopsy care kit.doc

Deception Script.docx

IM care kit.doc

*required

Provide information on who will conduct the debriefing and how it will be delivered to the participant.

Dr. Luk, Dr. Levitt, or clinical coordinator (future) will conduct the debriefing.

Upon the completion of the biopsy procedures, we will provide a take-home kit (i.e., notes and necessary consumables, e.g., extra bandage, alcohol

10-3a(3) wipes). Also, we will verbally explain the biopsy care instructions and a list of drugs that can increase bleeding (see the attachment), especially focusing on the medication that should be avoided. Furthermore, our research team will follow up with the participant in the following two days by text/call to make sure no adverse effect was observed (swelling and warmth at the site of biopsy). You can also send us a photo of the biopsy site through email or text message if you have any concerns. We will look at your biopsy site on your next visit.

For the control group (CON), we will disclose that the temperature setting for the heat pad used during the study was for control purposes, and we will specify the temperature they need to set to achieve an intramuscular temperature of 40 degrees Celsius.

No

10-4 TTU Compliance Committees

Additional approvals

*required

10-4a Does this research require internal approval from another TTU compliance committee or entity?

✓ Yes

10-4a(1) Select the TTU Committee(s) that grants approval to conduct research at that location and attach the approval letter.

Texas Tech Neuroimaging Institute (TTNI)

✓ TTU Institutional Biosafety Committee (IBC)

*required

Attach the approval letter.

Luk_IBC.pdf

IBC-2023-1083 approved v9.pdf

2023-2025_KSM_DXA_Sublicense.pdf

10-4a(1)b	Luk_DEXA training.pdf<br>Luk_Handling Radioactive Material.pdf<br>Luk_Introduction to Radiation Safety.pdf<br>Luk_Radiation Safety.pdf<br>Luk_X-Ray Safety.pdf

✓ TTU Institutional Laboratory Safety Committee (ILSC)

*required

Attach the approval letter.

10-4a(1)c	Luk_IBC.pdf<br>IBC-2023-1083 approved v9.pdf<br>2023-2025_KSM_DXA_Sublicense.pdf<br>Luk_DEXA training.pdf<br>Luk_Handling Radioactive Material.pdf<br>Luk_Introduction to Radiation Safety.pdf<br>Luk_Radiation Safety.pdf<br>Luk_X-Ray Safety.pdf

Institutional Animal Care and Use Committee (IACUC)

✓ TTU Radiation and Laser Safety Committee (RLSC)

*required

Attach the approval letter.

10-4a(1)e	Luk_IBC.pdf<br>IBC-2023-1083 approved v9.pdf<br>2023-2025_KSM_DXA_Sublicense.pdf<br>Luk_DEXA training.pdf

Luk_Handling Radioactive Material.pdf

Luk_Introduction to Radiation Safety.pdf

Luk_Radiation Safety.pdf

Luk_X-Ray Safety.pdf

Other

No

Complete the following section.

11-1 **Personal Identifiers**

*required

11-1a **Select personal identifiers that will be collected for the purpose of this research.**

Check any identifier you will be in possession of during the research process.

- ✓ Names
- ✓ Telephone numbers
- ✓ Any elements of dates (other than year) for dates directly related to an individual, birth date, admission date, discharge date, date of death. For ages over 89 all elements of dates (including year) indicative of such age, expect that ages and elements may be aggregated into a single category of age 90 and older
- Any geographic subdivisions smaller than a state, including street address, city, county, precinct, zip code and their equivalent geocodes except for the initial three digits of a zip code
- Fax numbers
- ✓ E-mail addresses
- Social security number
- Medical record number
- Student ID username/number
- Health plan beneficiary numbers
- Account numbers

Certificate/license numbers

Vehicle identifiers and serial numbers (VIN), including license tag identification

Device identifiers and serial numbers (e.g., implanted medical device)

Web universal resource locators (URLs)

Internet protocol (IP) address numbers

Biometric identifiers, including finger and voice prints

Full face photographic images and any comparable images (i.e., videos, etc.)

✔ Biological specimens

Any other unique identifying number, code, or characteristic, other than dummy identifiers that are not derived from actual identifiers and for which the re-identification key is maintained by the health care provider and not disclosed to the researcher

No personal identifiers will be collected

11-2 **Privacy of Participants**

---

*required

11-2a **Describe how you will protect the privacy of the participants.**

Consider how you will protect the participant's privacy during:

- Recruitment
- Consent
- Data Collection
- Storage of Data
- Analysis of Data
- Findings

Data Safety:
To protect against breach of confidentiality, any hard copy materials (e.g., informed consent form, any hard copies of exercise records with heart rate and rating of perceived exertion

data, etc.) will be kept in a locked drawer in Dr. Luk's private office. The informed consent form and demographics files will be the only places participants' names appear, and only the PIs and essential study personnel will have access. Participant samples, data, and records will be deidentified and labeled using a unique ID number. Data, images, and files generated from the project will always be stored in raw form, separate from files with calculations and analyses. Lastly, all electronic material, including data from questionnaires administered using computer-assisted interview techniques (i.e., no hard copy) will be stored in a secure database (REDCap).

11-3 De-Identifying Data

Anonymous v. Identifiable Data

*required

11-3a **Explain how you will de-identify the data collected. If data will be given a code, explain this process.**

For example:
Personal identifiers will be removed from the data and replaced with a participant code. The participant code will be used on the individual's data. The master list containing the personal identifier(s) and participant code will be in a separate document stored away from the data. The master list and data will be password protected. The passwords will be different for the master list and data.

For each identifier explain when and how you will code/de-identify/destroy the identifier.
Consider the following:

- What identifiers are associated with data?
- What identifiers are NOT associated with the data?
- What descriptors can be used to identify someone? For example: schools, towns, buildings, colleagues, companies, titles, etc.

Upon participant's admission to the study, a unique study identification code (ID codes) will be assigned to the participant in the order they are enrolled. The file containing participants' names and IDs will be stored electronically in REDCap.

Thereafter, all data will be kept in coded participant files. All trained investigators, professional staff, and technicians are aware of the confidentiality involved with this study.

Identifiable data will not be available or divulged to anyone outside of the experimental research team and data files will be kept for at least 3 years after the study is terminated. The confidentiality of individual information will be maintained in any publications or presentations regarding this study.

11-4 **Data Transcription**

*required

11-4a **Select who will transcribe audio or voice recording.**

Check all that apply.

Member of the research team/lab

Professional transcription company

Other

✓ Not applicable

11-5 **Transfer of Data**

*required

Will data be transferred from one location to another?

11-5a

Transfer of data can be done electronically (email, removable storage device, shared drives, remote access, hyperlinks, etc.) and/or physically (carrying, mailing, etc).

✓ Yes

*required

Explain how it will be securely transported and by whom.

1. Data type (human subjects data only)

A. **Types and amount of scientific data expected to be generated in the project:**
Data from human subjects: Demographic and anthropometric (e.g., age, sex, race, ethnicity, body mass, body composition, height), clinical (e.g., medical history, oral glucose tolerance test parameters, and questionnaires about alcohol use, diet, activity, sleep, changes in body mass, and fatigue), functional (e.g., exercise capacity, grip and leg strength, gait speed), and molecular (e.g., type I and II muscle fiber cross-sectional area, capillarization, mitochondrial enzyme activity, mitochondrial and TRPV1 protein expression, mitochondrial DNA quantification, and mitochondrial content and morphology) data will be collected from 54 older adults at risk for type 2 diabetes (described in detail in the “Human Subjects” attachment) at several time points throughout the study. Weekly assessments will include body mass measurement, diet and alcohol questionnaires, and activity and sleep monitoring via and self-report questionnaires. All other data will be collected only at the beginning of the study (i.e., demographics, medical history) or at the 2-3 major time points. Participants assigned to the high-intensity interval training (HIIT) group (n=18) for phase 1 will have 2 major time points (before [T1] and after [T2] phase 1). Participants assigned to the LHT (n=18) and CON (n=18) groups for phase 1 and HIIT for phase 2 will have 3 major time points (before phase 1 [T1], between phases 1 and 2 [T2], and after phase 2 [T3]).

Large-scale data sets: It is possible that large scale mitochondrial genomic data (human participants) and proteomic data (cell culture) could be generated from this project if alternative approaches must be used as described in the research strategy.

The total amount of anticipated data if alternative approaches are not needed is ~25 GB of data.

B. **Scientific data that will be preserved, shared, and the rationale for doing so:** All raw data generated from this project will be stored in a password-protected manner on cloud-based servers at Texas Tech University. Cleaned data in an analysis-ready format will be stored in REDCap and periodically uploaded to repositories. Data of sufficient quality will be uploaded to allow others to validate and replicate research findings described in the Aims.

C. **Metadata, other relevant data, and associated documentation**: In alignment with the Data Submission Worksheet for the Aging Research Biobank, the following will be submitted to accompany data derived from human subjects:
•Full study protocol
•Names and descriptions of data sets
•Manual of operations

•Annotated data collection forms
•Data dictionary
•Documentation of calculated variables
•Summary of deidentification and crosslinking of study ID with new randomized ID
•Any changes made to the protocol over time
•Frozen datasets used for the primary publication, when applicable
Similarly, the full study protocol, dataset names/descriptions, data dictionary, documentation of calculated variables, and any changes to the protocol over time will be deposited in TTU Dataverse for molecular procedures.

2. **Related tools, software, and/or code:** Statistical analyses will be run using standard statistical software (SPSS version 29) and graphs made using GraphPad Prism (Version 9). Image processing will be performed using ImageJ and in part using custom Python code to automate quantification where appropriate.

3. **Standards:** No consensus standards of data formatting such as Dublin or Darwin core exist for our discipline. Data dictionaries defining each variable and decoding any coded responses will accompany all uploaded data.

4. **Data preservation, access, and associated timelines:**
A. Repository where scientific data and metadata will be archived:
Human subjects data: NIA's Aging Research Biobank
Molecular data: TTU Dataverse Collection
Mitochondrial genomic data: Database of Genotypes and Phenotypes
11-5a(1) (dbGaP; if performed)
Custom code: GitHub

B. How scientific data will be findable and identifiable: Data will be findable for the research community through the repositories listed above. Collections will be established in the Aging Research Biobank and in Dataverse when this application is funded. Collections will be established on dbGaP should a large data set be generated. Data stored in the Aging Research Biobank will be made available by reasonable request, and this will be noted in the "Data Availability Statement" in associated publications. When data are made public via Dataverse, a digital object identifier (DOI) will be assigned. This DOI be referenced in associated publications to allow the research community access to the data used in each publication. If dbGaP is used, researchers can submit requests to dbGaP to access and browse data, and this information will be included in any associated publications. Any custom code will be shared on GitHub, accessible by searching "rbudnar" on GitHub. The readme.md file for the project will include instructions for the analyses and rationale for parameter choices.

C. When and how long the scientific data will be made available: Data associated with each publication will be made available when preprints are available and will continue to be available on the repositories and long as

they are supported. Any data that are not yet available by the end of the award period will be made available at that time.

5. Access, distribution, or reuse considerations:
A. Factors affecting subsequent access, distribution, or reuse of scientific data: Broad data sharing will be included in the informed consent form. Data deidentification, linking data using unique identifiers, protection of privacy, and making data available will be explained to participants and any questions answered as part of the informed consent process.

B. Whether access to scientific data will be controlled: Access to human subjects data will be controlled. Requests will be submitted through the appropriate repository (Aging Research Biobank, Dataverse, dbGaP).

C. Protections for privacy, rights, and confidentiality of human research participants: The only location that personal information will be paired with participant ID will be on a single administrative data collection form in the secure REDCap database. This is necessary because data will be collected from the same participants and multiple time points. However, each participant will be assigned an ID code and only the ID code will be linked to data so that the data are not identifiable. Data from human participants will be further deidentified upon submission to the repository(ies) and linked only through the study ID. The Aging Research Biobank further anonymizes data by randomly generating a new ID for each subject. dbGaP interfaces with the Aging Research Biobank so clinical and mitochondrial genomic data (if generated) can be linked, using only the randomly generated ID. Further, access to these datasets will be controlled and require that users register and submit reasonable requests. The PIs attest that no attempt will be made to reidentify participants from deidentified data.

6. Oversight of DMS: Drs. Hui-Ying Luk and Danielle Levitt (PIs) will deposit clinical and associated data into the NIA Aging Research Biobank, TTU Dataverse, and into dbGaP if mitochondrial genomic data are generated. Data upload will occur when publications associated with each data set are submitted and made public (where applicable) upon acceptance. Data sharing will be included in the annual progress report.

7. Representatives of the following people/groups within *Texas Tech University* may use your study records and share them with other specific groups in connection with this research study.
• The principal investigators (*Hui Ying Luk, Danielle Levitt*)
• The *Texas Tech University* Institutional Review Board
• The *Texas Tech University* Human Subjects Protection Office
The above people/groups may share your unidentifable study records with the following people/groups outside *Texas Tech University* for their use in connection with this research study. These groups, while monitoring the research study, may also review and/or copy your original *Texas Tech University* records.

• The Office of Human Research Protections in the U. S. Department of Health and Human Services.
• The Safety Officer appointed for this study.
• The National Institutes of Health and its authorized representatives.

No

11-6 Certificate of Confidentiality

*required

11-6a Has a Certificate of Confidentiality been requested?

Yes

✓ No

11-7 Investigator Assurances

*required

11-7a Data Security

✓ I will protect the data through at least one of these security measures: encryption software, password protection, and /or physical barrier (locked doors, file cabinets, etc.).

I will NOT protect the data through at least one of these security measures: encryption software, password protection, and /or physical barrier (locked doors, file cabinets, etc.).

*required

11-7b **Access to Data**

✓ I will only allow the research team access to the identifiable data.

I will allow others outside of the research team to have access to identifiable data.

*required

11-7c **Destruction of Personal Identifiers**

✓ I will destroy personal identifiers prior to closing the IRB with the exception of the consent forms.

I will **NOT** destroy personal identifiers prior to closing the IRB with the exception of the consent forms.

*required

11-7d **Retention Policy**

✓ I will keep de-identified data and/or consent forms for at least 3 years following the closure of the IRB.

I will **NOT** keep de-identified data and/or consent forms for at least 3 years following the closure of the IRB.

*required

11-7e **Data Repository**

✓ I will post the de-identified data to a data repository.

I will **NOT** post the de-identified data to a data repository.

12: Benefits & Risks

Please respond to the following statements/questions to help our IRB reviewers recognize the benefits and risks associated with this study.

12-1 **Benefits**

*required

Describe the potential direct benefit(s) to subjects.

12-1a

Payments to subjects, course credit or contribution to science should not be included in this section.

All efforts will be made throughout the study to minimize any study-related risks to subjects. The Physical Activity Guidelines for Americans, 2nd Edition (2018), suggest that adults should engage in at least 150 minutes per week of moderate-intensity aerobic exercise (or an equivalent 75 minutes per week of vigorous-intensity aerobic exercise, including HIIT) to maximize the many health benefits of aerobic exercise. Even if people do not meet the minimum of 150 minutes, smaller amounts of exercise are still beneficial. Participating in this study will provide participants with a structured environment to help meet that exercise goal during one of the two phases. Among anticipated benefits from exercise, we emphasize that there might be metabolic health benefits and decreased frailty risk, particularly because participants will be prediabetic older adults, indicating risk for the development of type 2 diabetes and frailty.

Furthermore, benefits of heat therapy are becoming increasingly recognized and we anticipate that participants assigned to the local heat therapy group will have improved skeletal muscle health, glycemic control, and decreased frailty risk. Because of the benefits anticipated from local heat therapy, all participants will be provided with heating pads regardless of group. The HIIT group will be provided with heating pads and instructions for use at the end of their participation (T2). The control group will be provided with heating pads but instructed to use a lower heat setting T1. At the end of their study participation (T3), they will be provided with instructions for use. Regardless of group, participants will have regular interaction with members of the study team. Together with exercise, prosocial interaction may have psychological benefits.

Participants will be provided with the following non-diagnostic results from testing performed at each major time point (PRE, MID, and POST):

- Dual-energy X-ray absorptiometry (DEXA) reports: Body composition and bone mineral density compared to age- and sex-specific norms.
- Body mass index (BMI): Value calculated from body mass and height (kg/m^2) and BMI category (i.e., underweight, normal weight, overweight, obese).
- Gait speed: 4-m walk time compared to age- and sex- specific norms.
- Muscle strength: Average grip strength (3 trials per hand) and 30-second sit-to-stand test with comparison to age- and sex- specific norms.
- Exercise capacity: VO_{2peak} and comparison to age- and sex-specific norms.
- Blood sugar control: If fasting blood sugar, HbA1c, or 2-hr glucose values after the glucose tolerance test fall into the diabetic range at any time point, the participant will receive a letter advising you of the laboratory measurement and to follow up with your physician.

12-2 Risks

*required

Describe any potential risk to subjects and how you will minimize that risk.

While exercise and heat have many potential benefits, they always carry possible risks. These risks are minimal, and the benefits of exercise and heat therapy largely outweigh the risks of a sedentary lifestyle. Those with higher-than-average risk for complications of exercise (e.g., preexisting cardiovascular conditions) will be excluded from the study. Risks of sample collection are also minimal, and the precautions taken will further offset these risks. The qualifications of the PIs and the protections in place for human subjects will ensure a safe environment for study participation.

Protections against risk
Strategies for minimizing risks associated with medical history and questionnaires, collection of biological specimens, and exercise are described below. All procedures will be fully approved by the TTU IRB. Drs. Luk and Levitt are trained in phlebotomy and muscle biopsies techniques and have previously conducted these procedures in IRB-approved studies. Dr. Luk is currently approved and actively performing muscle biopsies at TTU, and Dr. Levitt is in the process of obtaining such approval. All study personnel will be required to maintain valid CPR and First Aid certifications, and emergency contact information will be kept on hand. All personal information and data will be kept in a secure database (REDCap).

Protection against and minimization of risks by procedure:
•Interviews and completing questionnaires: To minimize risks of emotional stress and fatigue, the length and number of questionnaires will be kept to a minimum, and the study team will establish rapport with participants to minimize emotional stress. Contact information for

guidance counselors will be available. Protection against breach of confidentiality will be ensured by keeping all physical study materials in locked, secure locations and all electronic material in a secure database (REDCap). Except for the demographics form, all data will only be linked with unique identifiers and not with any personally identifiable information. Data uploaded to repositories will have different randomly generated unique identifiers generated during upload to the NIA Aging Research Biobank that are different from those used in REDCap or on study forms.

•Blood collection: Participants will be informed of the risks of venipuncture and finger prick. Risk for infection is low with the use of sterile technique, and a sterile adhesive bandage will be applied to the blood collection sites. Blood collection will be performed by trained personnel using standard laboratory techniques: disinfecting the site with alcohol, using sterile disposable supplies, and applying pressure with sterile gauze to minimize bleeding and bruising. Syncope is possible, so phlebotomy procedures will be performed using phlebotomy chairs in case legs need to be elevated. Cold packs will be available. Participants will be asked if they have a history of fainting prior to blood collection.

•Muscle biopsy: The following are risks that are related to the muscle biopsy procedure and are only associated with/around the incision site: Pain, bleeding, excessive redness, bruising, tenderness, swelling, warmness of the skin, localized numbness, and pink-tinted drainage from the incision. In extreme cases, a low-grade fever may occur, which would indicate that the site may be infected. None of these complications are usually life-threatening, and most complications can be managed conservatively without hospitalization.

To minimize risks, muscle biopsies will be performed using standard sterile technique. The skin over the vastus lateralis will be cleaned using povidone-iodine and the area anesthetized using 1% lidocaine without epinephrine. A small incision will be made through the skin and fascia using a single-use sterile scalpel (#11). A sterilized Bergstrom biopsy needle will be inserted into the muscle and approximately 200 mg of muscle will be collected. After the sample has been collected, pressure will be applied to the area for 5 min. The skin will be closed with a sterile adhesive bandage, covered using sterile gauze, and compression wrapped. Each participant will be given verbal and written instructions for biopsy care and extra supplies (e.g., sterile adhesive bandages, alcohol pads) will be provided. The study team will follow up with each participant 2 and 5 days following their biopsies. Sterile supplies (gloves, gauze, scalpel, needles, etc.) will be used and the PIs will train personnel to assist in the procedure. In case a participant becomes lightheaded or feels faint, we will provide food or beverages containing simple sugar (e.g., juice).

• Intramuscular temperature measurement: There may be some discomfort during the insertion of the probe into your muscle. The topical anesthetic will numb the insertion area to minimize this discomfort, but you may feel pressure or a dull ache in the muscle as the needle moves through the muscle. At the end of the session, the probe will be withdrawn and a sterile dressing will be applied. Any swelling or redness after the study should be gone a few hours after completion of the study, but you may feel some muscle soreness for several days. Although the small probes are sterile, there is a slight risk of infection at the sites where the probes were placed. You will be instructed on how to keep the area clean
12-2a for a day or two following the study and will need to inform the researchers immediately

if you have any redness or swelling in the area. To minimize risks, this measurement will be performed using standard sterile technique. The skin over the incision site will be cleaned using povidone-iodine and the area anesthetized using topical anesthetic cream. At the end of the session, the probe will be withdrawn and the skin will be closed with a sterile adhesive bandage, covered using sterile gauze, and compression wrapped. Each participant will be given verbal and written instructions for care and extra supplies (e.g., sterile adhesive bandages, alcohol pads) will be provided. Sites will also be re-assessed at the next study visit.

•Anthropometric measurements: To minimize risks associated with fatigue, these procedures will be performed near a wall or other support and chairs kept nearby in case participants need to rest between measurements. Contact information for guidance counselors will be available.

•Dual-energy x-ray absorptiometry (DEXA): DEXA is considered a low-risk procedure. However, the use of x-rays, even in low amounts, does present some risk associated with ionizing radiation. To minimize these risks, the number of DEXA scans will be kept to a minimum.

•Functional tests, exercise capacity (VO2peak) testing, and HIIT: During screening, we will ensure that participants meet ACSM medical criteria for initiating an exercise program and do not have medical complications that would put them at undue risk (e.g., diabetes, renal disease, uncontrolled hypertension, other cardiovascular disease, etc.). Participants may become faint, nauseous, experience muscle strains or soreness, or fall. To minimize risk associated with physical activity, participants will be counseled on the importance of proper hydration and will regularly be asked about their physical condition and adjustments made as necessary. Chairs will be available for rest during functional testing, and a researcher will remain close by the participant in case they lose their balance. During VO2peak testing and HIIT, we will utilize cycle-based exercise instead of treadmill exercise to prevent the risk of falls while walking, and participants will be helped on and off the cycle ergometer or exercise bicycle as needed. Participants will perform proper warm-ups and cool downs. We will also monitor heart rate and rating of perceived exertion during exercise. The VO2peak test will be performed in 3-minute stages with predetermined power output (i.e., males start at 50W, females start at 25W, and power increases by 20W every 3 minutes). If the participants cannot maintain the power output for a given stage, their cycling cadence falls below 50 rpm for two consecutive minutes, or they ask to stop the test, the test will end. In case a participant becomes lightheaded or feels faint, that day's exercise will be stopped and we will provide food or beverages containing simple sugar (e.g., juice).

•Local heat therapy: The potential risks of using local heat therapy include discomfort, skin redness, and, in rare cases, burns, particularly if used improperly or for extended periods. Core temperature increases are not typically observed with local heating on the thigh and thus is not considered a potential risk for the proposed study. However, we will monitor body temperature using a forehead scanner. If the body temperature reaches 40°C, we will stop the heat pad therapy. The risk of discomfort will be minimized by determining the lowest optimal heat pad temperature prior to the onset of the heat therapy intervention and that temperature will not be exceeded. Further, participants will be provided with contact information for the PIs should any problems occur during their at-home local heat therapy

sessions and will be asked about any discomfort during their weekly check-ins. Sometimes, redness may appear on your skin in a pattern after the heat therapy session, but it should fade within a day. Heat therapy sessions will occur in the laboratory once weekly and will be monitored by study personnel to ensure that no problems occur during heating sessions. The risk of burns due to personal heating pad use in older adults is not well-documented. However, in a 2021 study (PMID: 33031521), a review of burn injuries in older adults (age 65+) registered in the National Electronic Injury Surveillance System was performed. For adults aged 65-84 years, heating pads were not in the top 5 causes of burn injuries. In the 85+ year-old age group, heating pads were in the top 5 causes of burn injuries. An estimated 204 burns across 100 emergency departments in the United States over a 10-year period were attributable to electric heating pad use over a ten-year period, and all were in women. The risk of burns due to heating pads in this age group is increased by chronic comorbidities that are exclusionary for this study (i.e., diabetes, peripheral vascular disease, chronic kidney disease, neuropathy), minimizing risk. The risk also increases if heating pads are used while sleeping, so we will instruct participants to set a timer and/or alarm to ensure they do not exceed the 90-minute application time, also minimizing risk. Finally, it is estimated that a surface temperature between 42-43°C would produce a burn injury in 12-20 hours (PMID:1885636). Because the difference between skin and muscle temperature is approximately 2°C across a variety of studies, we anticipate that the heating pad setting to produce an intramuscular temperature of 40°C will be approximately 42°C. The 90-minute application time is far below the conservative end of the estimated contact time frame to produce a burn injury (12 hours).

12-2a(1) **Is a liability plan offered?**

Yes

✓ No

13: Participant Compensation

13-1 **Compensation - Payments to Research Participants**

Compensation through TTU funds must comply with TTU OP 62.25.

*required

13-1a **Are subjects being compensated for their participation in this study?**

✓ Yes

*required

Describe the compensation process.

Participant will be paid via cash upon completion of each visit:

Consent/screening ($10)

Major Measurement Testing (PRE, MID, POST):
•Muscle biopsy ($50) and OGTT/Questionnaires ($50)
•Physical function and exercise capacity tests ($50)
13-1a(1) *The major time point testing will be conducted twice for the HIIT group and three times for Local Heat groups.*

For each laboratory visit, participants will be compensated $15.
Phase 1:
•LHT: 12 visits = $180
•HIIT: 36 visits = $540

Phase 2:
•LHT: 36 visits = $540

Total:
•LHT: $1180
•HIIT: $850

13-1a(2) **Select the payment type used.**

✓ Cash

*required

Amount of Payment

13-1a(2)a

Amount will be paid according to the completion of each visit, please see above for details. Participants who are assigned to the LHT groups and complete each visit in its entirety will receive a total of $1180. Participants who are assigned to the HIIT groups and complete each visit in its entirety will receive a total of $850.

*required

13-1a(2)b

When and how will the participant be paid?

payments in cash will be disbursed after each visit

Check

Gift Card (not to be checked if a drawing is involved)

Drawing

Course Credit

Other (includes payment or credit from 3rd party affiliations (i.e., MTurk, SSI, etc.)

Investigator Assurances

Guidance: The Institutional Review Board (IRB) should determine that the risks to subjects are reasonable in relation to anticipated benefits [21 CFR 56.111(a)(2)] and that the consent document contains an adequate description of the study procedures [21 CFR 50.25(a)(1)] as well as the risks [21 CFR 50.25(a)(2)] and benefits [21 CFR 50.25(a)(3)]. It is not uncommon for subjects to be paid for their participation in research, especially in the early phases of investigational drug, biologic or device development. Payment to research subjects for participation in studies is not considered a benefit, it is a recruitment incentive. Financial incentives are often used when health benefits to subjects are remote or non-existent. The amount and schedule of all payments should be presented to the IRB at the time of initial review. The IRB should review both the amount of payment and the proposed method and timing of disbursement to assure that neither are coercive or present undue

influence [21 CFR 50.20].

13-1a(3)

Any credit for payment should accrue as the study progresses and not be contingent upon the subject completing the entire study. Unless it creates undue inconvenience or a coercive practice, payment to subjects who withdraw from the study may be made at the time they would have completed the study (or completed a phase of the study) had they not withdrawn. For example, in a study lasting only a few days, an IRB may find it permissible to allow a single payment date at the end of the study, even to subjects who had withdrawn before that date.

While the entire payment should not be contingent upon completion of the entire study, payment of a small proportion as an incentive for completion of the study is acceptable to FDA, providing that such incentive is not coercive. The IRB should determine that the amount paid as a bonus for completion is reasonable and not so large as to unduly induce subjects to stay in the study when they would otherwise have withdrawn. All information concerning payment, including the amount and schedule of payment(s), should be set forth in the informed consent document.

---

*required

13-1a(3)a **Participant Payment**

✓ I will provide compensation to all participants who complete the consent process and begin the study, regardless of whether they complete the study.

I will **NOT** provide compensation to all participants who complete the consent process and begin the study, regardless of whether they complete the study.

---

*required

13-1a(3)b **TTU OP 62.25**

All questions concerning participant payment should be directed to the TTU Payroll & Tax Services office or the researcher's business manager.

✓ I will follow the procedures outlined in TTU OP 62.25 regarding participant payment.

I will **NOT** follow the procedures outlined in TTU OP 62.25 regarding participant payment.

No

Conflict of Interest

Guidance: A conflict of interest refers to a situation in which an employee(s) financial, professional, or other personal considerations may directly or indirectly affect, or have the appearance of affecting, the employee(s) judgment in exercising any duty or responsibility, including the conduct or reporting of research, owed to the institution (TTU OP 10.20 (2)(d)).

14-1

"Significant business or financial interest" means anything of monetary value including, but not limited to, salary or other payments for services (e.g., consulting fees or honorarium; equity interests (e.g., stocks, stock options, or other ownership interests); and intellectual property rights (e.g., patents, copyrights, and royalties from such rights). (TTU OP 74.17).

"...a set of conditions in which an investigator's judgment concerning a primary interest (e.g., subject welfare, integrity of research) could be biased by a secondary interest (e.g., personal or financial gain)." Institutional Review Board Management and Function, Bankert and Amdur, 2006, p. 167.

*required

14-1a **Does anyone on the research team have a potential conflict of interest with the research project?**

Yes

✓ No

15: Investigator Attestations

*required

I, as the PI and co-investigators, understand Texas Tech University's policy concerning research involving human subjects and by checking below, I certify:

- ✓ I have read The Belmont Report "Ethical Principles and Guidelines for the Protection of Human Subjects of Research" and subscribe to the principles it contains.
- ✓ I have read 45 CFR 46, Protection of Human Subjects and subscribe to the regulations it contains.
- ✓ I accept responsibility for the scientific and ethical conduct of this research study and understand my responsibility.
- ✓ I will ensure that all study personnel are appropriately trained and are competent to perform the study.
- ✓ I will obtain prior approval from the Institutional Review Board (IRB) before making any modifications of the previously approved research, including modifications to the informed consent process and document.
- ✓ I will submit a Study Closure Submission upon completion of this study and agree to honor any other commitments.

✓ I will follow TTU procedures and guidelines to protect the health and well-being of research participants, research team members, and liaison(s) during any unforeseen circumstances.

✓ I will immediately report to the IRB any deviations, violations, non-compliance, unanticipated problems, and adverse events related to subjects or others which occurred or possibly occurred as a result of this study.

45 CFR 46.103(a) and TTU IRB Policy 5.4 require Adverse Events, Unanticipated Problems and Deviations from the research protocol to be reported **IMMEDIATELY** to the HRPP office.

Human Research Protection Program
Box 41075
Lubbock, Texas 79409
Phone: (806) 742-2064
Email: hrpp@ttu.edu

16: Additional Information

16-1 **Include any additional points to help clarify the research, if applicable.**

16-1a

16-2 **Enter or attach any additional documentation.**

16-2a

Modification Submission

Modification

*required

Cayuse IRB Modification:

Provide a summary of the changes you are requesting in the text box below. Details of the changes are required in the appropriate sections. The "Compare" feature is a useful tool as you edit each section.

Summary of Changes.

In this modification, we are submitting oral scripts for door-to-door, phone, and group recruitment efforts, along with a written script for email recruitment.

*required

Current Research Activities

Check all that apply.

Research procedures have **NOT** started.

✓ Participants are being recruited for this research.

*required

Explain the recruitment procedures that have started.

We have begun posting flyers at TTU and HSC campuses, pharmacies, supermarkets, and on TechAnnounce.

Recruitment has ended.

Participants have been consented to do the research.

Participants have **NOT** been consented to do the research.

Data collection is **ONGOING.**

Data collection is **COMPLETE.**

Analysis of **IDENTIFIABLE** data.

Analysis of **DE-IDENTIFIED** data.

1: Human Subject Research

Answer the following questions to determine if you need IRB review and approval.

1-1 Research

*required

Research is defined by 45 CFR 46.102(l) as:

A systematic investigation, including research development, testing and evaluation, designed to develop or contribute to generalizable knowledge.

Does your proposed study meet the definition of research?

✓ Yes

No

1-2 Human Subjects

*required

Human Subject is defined by 45 CFR 46.102(e) as:

A living individual about whom an investigator (whether professional or student) conducting research

- Obtains or receives private identifiable information or identifiable biospecimens about the individual(s)
- Obtains information or biospecimens through intervention or interaction with the individual(s)

Does your proposed study involve and meet the definition of human subjects?

✓ Yes

No

If you answered YES to all questions in Section 1 complete the rest of the sections.

Complete the following section.

*required

2-1 Review Type

Select the appropriate review type.

Exempt

Expedited

✓ Full Board

The project involves greater than minimal risk to human subjects.

Relying on IRB approval from another institution (IAA)

2-2 External Collaboration

Only select collaboration if there are Non Texas Tech researchers, employees, and/or students working on this research.

Collaboration

2-3 International Research

Research will be conducted outside of the United States of America and/or data will be shared across borders.

International Research

Complete the following section.

Lay Summary

*required

Provide a description of your study in vernacular language.

Avoid the use of technical terms, undefined acronyms, or excessive scientific terminology.

Aging people with prediabetes are at increased risk for frailty, and skeletal muscle architectural factors (which may improve with exercise or heat therapy) could underlie development of frailty and progression to type 2 diabetes. The proposed study will test local heat therapy as a method to improve skeletal muscle architecture (i.e., cross sectional area, capillarization, mitochondria), glucose tolerance, frailty indicators, and exercise adaptation in prediabetic older adults. Data generated will provide evidence supporting a directly translatable, easily implemented therapeutic intervention to ameliorate risk of developing overt type 2 diabetes and frailty in at-risk aging individuals.

Complete the following section. List all researchers involved with recruitment, data collection, and analysis.

NOTICE: HUMAN SUBJECT TRAINING REQUIREMENT

All research study personnel who engage with participants will need to complete human subject training prior to IRB approval and prior to conducting any human research-related activities. The new training requirement will be applied when an existing study is due for renewal, requires modification(s), or is chosen for a post approval monitoring.

Human research-related activities include recruitment of participants, consenting of participants, data collection (anonymous or identifiable), gaining access to identifiable data, and/or conducting analysis of identifiable data. These training requirements will apply to all Principal Investigators, Co-Investigators, and research staff/students. This includes both individuals within the TTU system and external collaborators.

This requirement will go into effect January 1, 2023. For more information visit our webpage.

Effective January 1, 2023, TTU research personnel must complete one of the following trainings every 3 years:

- CITI - TTU Human Subject Research - Required Basic
- TTU Human Subject Training
- External – CITI Human Subject Training

4-1 **Principal Investigator**

TTU OP 74.09

*required

4-1a **Check the appropriate box.**

✓ TTU full-time or tenured faculty member

*required

4-1a(1) **College**

College of Arts and Sciences

*required

4-1a(2) **Department**

Kinesiology & Sport Management

TTU full-time employee with a terminal degree in their discipline

*required

Find and add PI to the submission.

4-1b Name: Hui Ying Luk
Organization: Kinesiology and Sport Mgmt
Address: 2500 Broadway MS3011, Lubbock, TX 79409
Phone: 8068340827
Email: huiying.luk@ttu.edu

4-1a(3)a Attach human subject training.

Luk_CITI.pdf

Primary Contact

4-2

Primary contacts need to be included in section 4.3 as a co-investigator if they are not the principal investigator. The primary contact and principal investigator will receive all notifications and letters.

*required

4-2a **Find and add primary contact to the submission.**

Name: Danielle Levitt-Budnar
Organization: Kinesiology and Sport Mgmt
Address: 2500 Broadway MS3011, Lubbock, TX 79409
Phone: 8068341830
Email: Danielle.Levitt@ttu.edu

Name: Hui Ying Luk
Organization: Kinesiology and Sport Mgmt
Address: 2500 Broadway MS3011, Lubbock, TX 79409
Phone: 8068340827
Email: huiying.luk@ttu.edu

4-3 **Co-Investigators**

Include investigators assisting with instrument development, recruitment, data collection, and data analysis.

*required

4-3a **Select all investigators involved with this research project.**

None

✓ List TTU Faculty or Staff Co-Investigator(s):

*required

Multiple names are allowed.

Name: Fangyuan Zhang
Organization: Mathematics and Statistics
Address: 2500 Broadway MS1042, Lubbock, TX 79409
Phone: 8068342587
Email: fangyuan.zhang@ttu.edu

Name: Heather Vellers
Organization: Kinesiology and Sport Mgmt
Address: 2500 Broadway MS43011, Lubbock, TX 79409-3011
Phone: 8068348554
Email: heather.l.vellers@ttu.edu

4-3a(1) Name: Danielle Levitt-Budnar
Organization: Kinesiology and Sport Mgmt
Address: 2500 Broadway MS3011, Lubbock, TX 79409
Phone: 8068341830
Email: Danielle.Levitt@ttu.edu

Name: Terrell Hilliard
Organization: Kinesiology and Sport Mgmt
Address: 2500 Broadway Ms 3011, Lubbock, TX 79409
Phone: 8068345585
Email: terhilli@ttu.edu

Name: Jaden Hendrix
Organization: Kinesiology and Sport Mgmt
Address: , Lubbock, TX 79409-1035
Phone: 8067423371
Email: jadhendr@ttu.edu

Name: Benjamin Barr
Organization: Kinesiology and Sport Mgmt
Address: 2500 Broadway MS 3131, Lubbock, TX 79409-3131
Phone: 8067423371
Email: Benjamin.Barr@ttu.edu

Attach human subject training.

4-3a(1)(a) Terrell_ Human Subject Research.pdf

Jaden_Human_Subject_JH_7.24.pdf

TTU Human Subject Research - CITI.pdf

✓ List TTU Graduate or Undergraduate Co-Investigator(s):

4-3a(2) *Multiple names and selections are allowed.*

✓ Graduate Student(s)

*required

Name: Casey Appell
Organization: Kinesiology and Sport Mgmt
Address: , Lubbock, TX 79409-1035
Phone:
Email: Casey.Appell@ttu.edu

Name: Parisa Foroozan
Organization: Kinesiology and Sport Mgmt
4-3a(2)a Address: , Lubbock, TX 79409-1035

Phone:
Email: parisa.foroozan@ttu.edu

Name: Masoumeh Rasoli
Organization: Kinesiology and Sport Mgmt
Address: , Lubbock, TX 79409-1035
Phone:
Email: mrasoli@ttu.edu

4-3a(2)a1 Attach human subject training.

---

*required

4-3a(2)b **The purpose of this study:**

Doctoral Dissertation

Master's Thesis

Research conducted as a class assignment with the intention of publishing results

Class assignment requiring IRB submission with no intention of publishing results

✓ Students are members of the research team

Other

✓ Undergraduate Student(s)

*required

4-3a(2)c

Name: Ashley Williams
Organization: Kinesiology and Sport Mgmt
Address: , Lubbock, TX 79409-1035
Phone:
Email: wil98200@ttu.edu

Name: Matthew DiLeo
Organization: Kinesiology and Sport Mgmt
Address: , Lubbock, TX 79409-1035
Phone:
Email: madileo@ttu.edu

Name: Connor Mills
Organization: Kinesiology and Sport Mgmt
Address: , Lubbock, TX 79409-1035

Phone:
Email: mil67126@ttu.edu

Name: Ernan Carrasco
Organization: Kinesiology and Sport Mgmt
Address: , Lubbock, TX 79409-1035
Phone:
Email: erncarra@ttu.edu

4-3a(2)c1 Attach human subject training.

---

*required

4-3a(2)d **The purpose of this study:**

Undergraduate Research

✓ Students are members of the research team

Research conducted as a class assignment with the intention of publishing results

Class assignment requiring IRB submission with no intention of publishing results

Other

✓ External Researchers: Co-investigators outside of Texas Tech University

*required

4-3a(3) **List non-TTU investigator(s) engaged in this research. Provide their contact information, title, and place of work.**

Robyn Richmond, MD
Assistant Professor of Surgery
Associate Program Director, General Surgery Residency
Trauma, Surgical Critical Care, Acute Care Surgery
Department of Surgery
Texas Tech University Health Sciences Center
robyn.richmond@ttuhsc.edu
972 987 9616

Leslie Shen, Ph.D.
Associate Dean for Research & Professor of Pathology

School of Medicine
Department of Pathology
Texas Tech University Health Sciences Center
leslie.shen@ttuhsc.edu

Rama Chemitiganti, MD
ECHD Endowed Chair of Medicine
Director, Center of Excellence for Diabetes and Endocrinology
School of Medicine
Department of Internal Medicine
Texas Tech University Health Sciences Center at Permian Basin
rama.chemitiganti@ttuhsc.edu
806-743-3280

*required

4-3a(3)a **Check all that apply.**

Developing instrument

Recruiting participants

Interaction with participants

Working with de-identified data

Working with identifiable data

Assisting with writing manuscript

✓ Other

*required

Include any information that helps to explain their role with the research team.

4-3a(3)a1

Dr. Richmond will provide medical-related oversight/consultation on the muscle biopsy procedures. There will be no direct interaction between Dr. Richmond and the participants.

Dr. Shen will provide consultation on participant recruitment.

Dr. Chemitiganti is the NIH-approved Safety Officer for this study. There will be no direct interaction between Dr. Chemitiganti and the participants.

Attach human subject training.

5: Funding

Complete the following section.

5-1 Funding

Funding is used to supplement the cost of the research and/or participant payment.

*required

5-1a **Select the type of funding to be used.**

✓ External (Office of Research Services - ORS)

*required

5-1a(1) **Cayuse SP - Sponsored Project Number (Example: 16-0123)**

23-0477

*required

5-1a(2) **Sponsored Project Title**

Glycemic control and frailty risk in older people at risk for type 2 diabetes: Impact of local heat therapy

*required

5-1a(3) **Find and add the Sponsor's name.**

Name

-

- Name - A to Z
- Name - Z to A

NIH - National Institute on Aging

*required

5-1a(4) **Attach a copy of the sponsored project that was submitted to the funding agency.**

For example: scope of work, abstract, research description.

FINAL_ESI R01_HYLDEL_Specific aims.docx

FINAL_ESI R01_HYLDEL_Research Strategy.docx

Internal (TTU Funding)

Personal Monies

No Funding

5-2 **Future Funding**

If funding is secured, a modification will be required.

5-2a **Enter the potential sponsor(s) name below.**

6: Rationale

Please respond to the following statements to help our IRB reviewers understand your research project.

Research Objective

6-1

*required

Briefly summarize the relevant background information.

Frailty-related medical expenses cost approximately $18 billion annually in the USA. Progressive losses in skeletal muscle (SKM) mass and function, often observed with aging and type 2 diabetes (T2D), contribute to phenotypic frailty characterized by slow gait speed, weakness, weight loss, fatigue, and low physical activity. T2D increases frailty risk by nearly 50% and risk for both conditions increases with age; therefore, preventing progression to T2D in older adults with prediabetes is critical. Additionally, SKM is the largest glucose disposal site in the body and insulin responsiveness, a component of glycemic control, is essential to maintain functional SKM mass. Thus, impaired glycemic control, a pathophysiological change underlying the development of T2D and observed in prediabetes, increases frailty risk. Conversely, improving capillarization and mitochondrial function support increased SKM mass and glycemic control, thereby decreasing T2D and frailty risk. Together, these SKM architectural variables (e.g., cross-sectional area [CSA], capillarization, mitochondria) are attractive targets for interventions such as exercise in prediabetic older people. High-intensity interval training (HIIT) has been used effectively in older adults with SKM benefits similar to those from aerobic and resistance training, and is more time-efficient. However, prediabetic older people may be exercise-intolerant or -resistant, underscoring the need for alternative therapies in place of (or in addition to) exercise. Emerging evidence supports repeated heat therapy as an alternative method to improve glycemic control and SKM architecture, and such adaptations may also improve muscle growth responses to subsequent exercise. While whole-body heat therapy is widely studied, local heat therapy (e.g., heat pad) is more practical and likely provides similar health benefits. Whether local heat therapy would have similar benefits to HIIT or whether heat pre-conditioning would improve adaptations to subsequent HIIT in prediabetic older adults is unknown.

6-2 Importance of Research

*required

Explain the scientific importance of the knowledge to be obtained as a result of this research.

Exercise is a first-line intervention for improving glycemic control and decreasing frailty risk due largely to adaptations in SKM. While exercise has a host of whole-body benefits, older individuals in general and those at risk for T2D (i.e., prediabetic) in particular may be exercise-resistant or -intolerant. Therefore, there is an urgent need for **efficacious and practical interventions** to improve muscle health in aging people to decrease their risk of developing T2D and frailty. The proposed project is innovative because we will:

- Use local heat therapy as an *easily implemented intervention* to improve the fundamental aspects of muscle health in aging prediabetic people, a novel application of this widely-used therapy
- Compare the efficacy of our proposed intervention alone and as a means of *preconditioning muscle* for improved exercise adaptations

Finally, this project will provide essential fundamental evidence to lay the groundwork for a subsequent clinical trial that examines the use of this technique in aging people with other SKM-related comorbidities. This work has the potential to **shift clinical practice** by providing direct evidence that supports a low-cost, practical intervention to improve metabolic health in at-risk individuals.

6-3 Research Questions/Specific Aims

*required

List the research question(s)/specific aims to be addressed.

The overarching hypothesis of this study is that local heat therapy improves muscle architecture, glycemic control, and subsequent exercise adaptations, and decreases frailty risk in prediabetic older adults, with TRPV1 as an underlying mechanism [mechanistic experiments will be in Aim 3, not part of this IRB application]. We will test this hypothesis through the following specific aims:

Aim 1: Test the hypothesis that local heat therapy improves muscle architecture, glucose tolerance, and frailty indicators similarly to HIIT in older prediabetic people.

Aim 2: Test the hypothesis that local heat therapy pre-conditioning improves the skeletal muscle response to HIIT in older prediabetic people.

6-5 Citations

Enter or attach the references cited .

The references below are from our Specific Aims and Research Strategy documents. Although they are not cited in the project summary, importance, and aim above, they are cited in the funding application.

ESI R01_Bibliography & References Cited.docx

Please respond to the following statements/questions to help our IRB reviewers know more about the participants in this study.

7-1 **Relationship with Participants**

*required

7-1a **Is there the potential for any relationship between the researcher(s) and the proposed target population?**

(Note: if recruiting in the Lubbock (which includes TTU) community please click yes as students and employees are possible)

✓ Yes

*required

7-1a(1) **Explain**

Recruiting older adults with prediabetes in Lubbock; it is possible that other TTU employees could be recruited.

No

Coercion or Undue Influence

Guidance for IRB and Investigators

Coercion - Coercion entails influencing an individual's decision about whether or not to

7-2 do something by using explicit or implied threats (loss of good standing in a job, poor grades, etc.). This area also includes the perception of students as to any potential backlash for failing to take part in an instructor's research.

Undue Influence - This is defined as an offer of an excessive, unwarranted, inappropriate, or improper reward or other overture in order to obtain compliance (used in association with recruiting research participants).

*required

7-2a **Does this research involve participants who could be coerced or unduly influenced to participate or may feel that there is perceived coercion or undue influence (i.e., students, employees, prisoners, colleagues, friends/acquaintances, family members, etc.)?**

✓ Yes

*required

Describe the plan to mitigate (perceived) coercion or undue influence to participants.

7-2a(1) One example of mitigating coercion/undue influence would be to add a sentence to the consent form that clearly states their decision to participate, not participate, or withdraw in the research will not impact their employment status or academic standing.

We will emphasize to participants that their enrollment and continued participation is completely up to them. They can withdraw from the study at any time for any reason without consequences. The PIs of this study do not currently have any influence over the employment of individuals who would qualify for this study. Any future non-traditional student who might qualify will be assured that their grade(s) will noty be impacted, positively or negatively, by their participation or withdrawal. Colleagues in KSM who might qualify and enroll will be assured that our working relationships will not be impacted, positively or negatively, by their participation or withdrawal. The PIs are committed to ensuring that participation and/or withdrawal of KSM colleagues who might choose to participate and/or withdrawal does not impact our decisions for any future faculty votes, recommendations, etc. Finally, we will emphasize the importance of confiidentiality with our research team, **especially** when a participant might be a TTU (including KSM) employee.

No

7-3 Target Population

*required

7-3a **Describe the population of human subjects to be recruited and why this research specifically targets this group?**

In this study, participants with elevated blood glucose, prediabetes, or are at risk for developing diabetes; are sedentary will be recruited.

This population is targeted because the muscular pathophysiology of T2D (i.e., insulin resistance [IR], decreased skeletal muscle [SKM] mass and function) contributes to phenotypic frailty including slow gait speed, weakness, unintentional weight loss, fatigue, and low physical activity. Approximately 7-15% of community-dwelling older adults meet criteria for frailty, and 25-40% of these individuals are prediabetic. Moreover, a recent meta-analysis of community-based studies found that baseline T2D is associated with nearly 50% increased risk of frailty development.

Although exercise training is a key technique to manage metabolic disease and decrease T2D risk, in older adults at risk for T2D, compromised oxygen delivery and mitochondrial dysfunction result in exercise intolerance, a potential contributor to low exercise adherence. Thus, it is critical to find alternative means to manage metabolic disease risk. Heat therapy is one possible alternative. Heat therapy adaptations could improve exercise tolerance by preconditioning the microenvironment of skeletal muscle, likely enhancing exercise adherence and improving subsequent exercise adaptations.

*required

7-3b **Does your target population require specific characteristics?**

Yes, my target population **DOES** require specific characteristics.

✓ No, my target population **DOES NOT** require specific characteristics.

*required

7-3c **Age Range**

Check all that apply.

✓ Adults

*required

7-3c(1) **Enter the age range.**

≥60

Children

*required

7-3d **Enter the number of participants.**

27 men and 27 women

7-4 **Inclusion/Exclusion Criteria**

Enter or attach all criteria used to include or exclude participants for this research.

Individuals with elevated blood glucose, prediabetes, or are at risk for developing diabetes; are sedentary

Inclusion:
•Sedentary (structured exercise <30 minutes, 3x/week)
•Meet criteria for prediabetes (fasting blood glucose 100-125 mg/dl and/or hemoglobin A1c 5.7-6.4%)
•Age ≥ 60 years
• Body weight is at least 110 pounds
•Consume <8 (women) or <15 (men) alcohol-containing beverages per week
•Do not use nicotine or cannabis

•Not taking any medications that could interfere with responses to the interventions (e.g., corticosteroids, opiates, benzodiazepines, tricyclic antidepressants, beta blockers, sulfonylureas, insulin, metformin, anticoagulants, barbiturates, insulin sensitizers, fibrates [PPAR gamma agonist], immunosuppressants)

Exclusion:
•History of peripheral neuropathies
•Currently taking prescription blood thinners
7-4a •Medical complications that could would contraindicate participation in the high intensity interval training (HIIT) intervention including: orthopedic complications that would limit your ability to perform cycling exercise, significant cardiovascular impairments (e.g., history of arrhythmias, severe uncontrolled hypertension, etc.), diagnosed metabolic disease (e.g., diabetes), renal disease, sickle cell anemia, or cancer in remission for <6 months.
•Known history of slow wound healing
•Excessive subcutaneous fat over the vastus lateralis (greater than 1.5")
• Have experienced symptoms suggestive of cardiovascular, respiratory, metabolic, or renal diseases including discomfort, pressure, or pain in your chest, neck, jaw, arms, calves, or other areas potentially related to ischemia; shortness of breath at rest or with mild exertion; dizziness or fainting (syncope); difficulty breathing while lying flat (orthopnea) or sudden nighttime breathing difficulties (paroxysmal nocturnal dyspnea); palpitations or rapid heartbeat (tachycardia); pain or cramping in your legs during physical activity (intermittent claudication); a known heart murmur; swelling in your ankles (edema); unusual fatigue or shortness of breath during routine activities or at rest.
• Currently pregnant
• Lidocaine allergy
• Latex allergy

We will screen for these criteria using the medical history form (attached). After inquiring with the participant, "Yes" to the following questions would result in exclusion from the study: 1, 3-7, 9-12, 15-17 (unless acute illness), 19 (lidocaine only), 20 and 23. Answers to other questions will be compared against inclusion/exclusion criteria (i.e., injuries, illnesses, chronic conditions, other reason that they should not exercise, etc.) to determine whether the participant meets all inclusion and no exclusion criteria. A key for research staff is included as a separate page in the form (beginning in Column Z on the spreadsheet).

7-4a(1) Inclusion/Exclusion Screening Form (if applicable)

R4_Feb 3_IM_HEAT study_Medical History Questionnaire.xlsx

7-5 **End of Subject Participation**

*required

7-5a **Enter specific criteria used by the researchers to prematurely end a subject's participation in the study.**

Sometimes a PI will decide to remove a participant from a research study. List any reasons you would anticipate that may require this removal.

This is not a participant deciding they would like to stop/withdraw from the research. This is a researcher ending their participation. For example: unforeseen risk to the participants, risks to the other participants/research team members, non-adherence, disruptive behavior.

The researchers will end participants' involvement in the study early if they do not follow the study's instructions, which include:

- Adopting a new lifestyle
- Starting a new exercise routine and/or diet program
- Taking any new supplements
- New medical conditions or complications that would place participants at increased risk
- Low adherence to interventions (<80% compliance)

7-6 **Investigator Assurances**

*required

7-6a **Data Storage and Retention**

✓ I will provide participants with information about data storage and retention if they withdraw from the study, when contacted by subjects per contact information provided in the consent form.

I will **NOT** provide participants with information about data storage and retention if they withdraw from the study, when contacted by subjects per contact information provided in the consent form.

*required

7-6b Participant Withdraw

✓ I will provide participants with information about how to withdraw from the study through the provided consent form.

I will **NOT** provide participants with information about how to withdraw from the study through the provided consent form.

8: Recruitment Procedures and Material

Please respond to the following statements/questions.

8-1 **Recruitment Procedures**

*required

Describe the process of how participants will be recruited and provide the sequence of these events.

This section needs to include the details of each step for recruitment up to the point of consent. This includes pre-screening procedures.

8-1a

All planned recruitment efforts and scripts will be approved by the Texas Tech University IRB. We will begin recruitment through the University Medical Center in partnership with Texas Tech University Health Sciences Center physicians who see patients from the community that may qualify. Email addresses and/or phone numbers will be obtained by searching for internal medicine, family medicine, geriatrics, and endocrinology clinics. Specially, fliers will be placed in the clinic and provided to physicians to distribute to potential participants.

Electronic advertisements will be posted on listservs (e.g., TechAnnounce, Obesity Research Institute listserv) and on social media (e.g., Facebook). Social media announcements will specify targeting older adults (60+) in the Lubbock, TX area. Additionally, after site-specific approval, flyers will be distributed around the community to frequently visited locations such as grocery stores, pharmacies, and community centers.

In addition, we will conduct door-to-door recruitment, make recruitment phone calls, and deliver recruitment speeches at various events (e.g., community center gatherings, church events, etc.). Lastly, we will disseminate recruitment emails with an attached flyer through personal and professional networks, including friends and colleagues who are willing to share the information.

Drs. Luk and Levitt will be responsible for executing recruitment efforts, collecting contact information for potential participants, conducting initial phone screenings, and following up with potential participants to schedule them for their informed consent and screening visit. Once a clinical coordinator is hired, these responsibilities will primarily shift to that

person. The initial phone screening is to ensure participant is at least 60 years old, sedentary, and their physician has informed them they have elevated blood glucose, prediabetes, or are at risk for developing diabetes.

8-2 **Recruitment Methods**

Templates

*required

8-2a **Select all methods of recruitment.**

Check all that apply.

No Recruitment

✓ Oral Script

*required

Enter or attach the Oral Script(s).

8-2a(2) *If more than one oral script is entered or attached, label each script separately.*

Script for UMC and TTUHSC phone recruitment is attached.

Scripts for door-to-door recruitment, phone recruitment, and recruitment events are attached.

If you attach a document, state *See Attachment* in the text box above.

8-2a(2)a

M2_July 16_Script_UMC and TTUHSC recruitment (1).docx

M2_July 16_event script.docx

M2_July 16_Phone Script.docx

M2_July 16_Door-to-door.docx

✓ Tech Announce

*required

8-2a(3) **Enter or attach TechAnnounce ad.**

See attached

8-2a(3)a If you attach a document, state *See Attachment* in the text box above.

R5_March 5_IM_Tech Announce Ad - HEAT study (2) (3).docx

✓ Recruiting Letter(s) or Email(s)

*required

Explain how email addresses or physical addresses of potential participants are obtained.

8-2a(4) We will search for any email addresses for UMC or TTUHSC physicians' offices.

Also, we will disseminate recruitment emails with an attached flyer through personal and professional networks, including friends and colleagues who are willing to share the information.

*required

8-2a(5) **Enter or attach all recruiting emails or letters.**

If more than one email is entered or attached, label each separately.

Scripts attached

8-2a(5)a If you attach a document, state *See Attachment* in the text box above.

Email template.docx

M2_July 16_Email Script_UMC and TTUHSC recruitment (1).docx

Information Sheet

✓ Advertisement(s)

*required

8-2a(8) **Check all that apply.**

✓ Print Advertisement (eg. newspaper, magazine, fliers)

Website

✓ Social Media

*required

8-2a(9) **Enter or attach the advertisement.**

If more than one advertisement is entered, label each separately.

The attached flyer will be used for print and social media advertising.

8-2a(9)a If you attach a document, state *See Attachment* in the text box above.

M1_R1_June 11_HEAT Flier (2).doc

Subject/Participant Pools

Third Party Recruitment

Other

8-2b Investigator Assurances

*required

8-2b(1) Permission to Recruit Participants

✓ I will acquire proper permission prior to recruiting participants.

I will **NOT** acquire proper permission prior to recruiting participants.

Permission to Conduct Research

8-3

*required

8-3a **Is this research study being conducted in a setting that requires prior permission from an entity to conduct research on their premises (i.e., school system, business, government entity, etc.)?**

Yes

✓ No

Visit the HRPP website for instructions and templates on the Consent Process, Assent Process with Minors, Short Form Consent Process, and Waivers of Consent.

Consent forms can be destroyed three years after the completion of the research. 45 CFR 46.115(7)(b).

9-1 **Consent**

*required

9-1a **Consent Process & Forms**

Check all that apply.

✓ Consent (Adult)

*required

9-1a(1) **Check all that apply.**

✓ Participant

Legal Authorized Representative Consent (LAR)

Parent/Legal Guardian

*required

Describe the process for obtaining consent. Include where and when the consent process takes place and who will be obtaining consent.

Drs. Luk or Levitt will provide a brief overview of the study using the information sheet and then seek informed consent from all potential participants in their offices in the KSM building to ensure privacy. All participants will be provided with copies of the information sheet and informed consent sheet. They will also be provided with a complete verbal explanation regarding the reasons for conducting the study, the procedures, risks, and benefits of the study, and any questions will be answered. At the end of the consent process, participants will be asked the following questions to establish capacity to consent:

9-1a(2)

Do you understand why this study is being done?
If you decide to participate in the study, what are some of the things you will be asked to do?
Describe some of the risks or discomforts you may experience if you participate in this study.
Will this study help you?
Do you have to be in this study?
What will happen if you decide not to be in the study?
Who should you contact if you have questions or experience a problem while in the study?

Correctly answering these questions will demonstrate an understanding of the study and determine the capacity to provide consent. Adults unable to demonstrate the capacity to consent will not be enrolled in the study.

*required

Enter or attach the consent form.

9-1a(3)

If more than one consent form is entered or attached, label each separately.

See attachment

If you attach a document, state *See Attachment* in the text box above.

9-1a(3)a

DXA Acknowledgement Form (1) (1).pdf

M1_R1_June 11_Informed Consent - HEAT study_Laymen term (4).docx

*required

Will a Short Form Consent be used?

9-1a(4) Only used in complex studies when the PI cannot be sure that a signed written consent is understood well enough to indicate a valid consent process.

Yes

✓ No

Assent (Minors & Individuals with Impaired Decision Making Skills that cannot consent for themselves)

Texas Tech Neuroimaging Institute (TTNI) Consent Form

No consent will be obtained.

9-2 **Waivers**

A consent form must still be included in the documentation.

9-2a **Check all that apply.**

Request for a Waiver of Written Consent

Request for a Waiver or Alteration of the Elements of Consent

FERPA Authorization

IRB approval or determination of exemption of the research project does not constitute institutional permission to access the requested records.

9-3 **Guidance:** FERPA applies to personally identifiable information in educational records. This includes items such as the student's name, names of family members, addresses, personal identifiers such as social security numbers, and personal characteristics or

other information that make the student's identity easily traceable.

Educational records are all records that contain information directly related to a student and are maintained by an educational agency or institution, or by a party acting on its behalf. A record means any information recorded in any way, including handwriting, print, tape, film, microfilm, microfiche, and digital images.

9-3a **Will you be using student records protected under FERPA?**

Yes

✓ No

HIPAA Authorization or Waiver

9-4

Indicate whether you are obtaining Participant Authorization or requesting a Full or Partial Waiver of HIPAA Authorization.

9-4a **Select the appropriate authorization.**

Participant Authorization of Protected Health Information

Full or Partial Waiver of Protected Health Information

10: Data Collection Procedures & Documentation

Complete the following section.

Data Collection

10-1 IRB members find it very helpful to have copies of all instruments. Reading the instrument(s) is essential to the board's assessment of participant risk and burden and the adequacy of the study description in the informed consent. Occasionally, however, investigators do not include all or full copies of instruments on the grounds that the instrument is copyrighted and in the belief that making copies to submit with an IRB application is a violation of copyright law. However, instrument developers and publishers should have a reasonable expectation that instrument users must obtain IRB approval of their research and that approval requires review of all study materials. Further, IRB members are well aware of their own ethical responsibility to honor copyright and purchase contracts by investigators and to refrain from use of copyrighted material for purposes other than those related to their IRB obligations. If records are being withheld pursuant to a Non-Disclosure Agreement (NDA), you (the investigator) are expected to provide the relevant portion of the NDA or any similar documentation requiring the records be withheld specifically from the IRB. If the NDA or license precludes sharing with the IRB, it is the PI's responsibility to get approval from the company or licensor to share the instrument(s) with TTU's IRB for review.

*required

Describe the sequence of events and timeline the participants will experience.

A randomized controlled intervention design will be used. The study consists of 2 phases, each 12-weeks in duration.

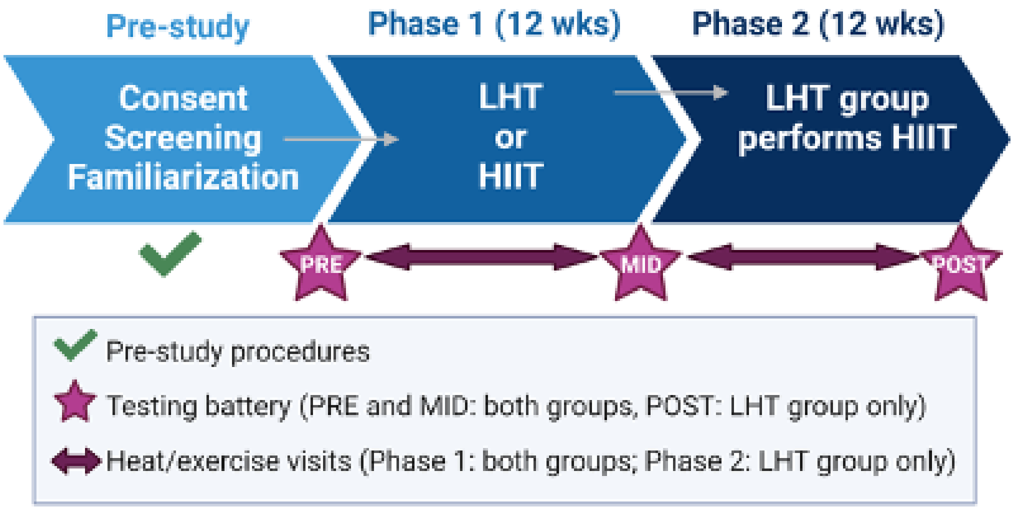

Visit 1: Consent and screening: (~2h)
•Participant will need to fast (i.e., no food or beverages other than water; plain water allowed and encouraged) for 12h before consent and screening.
•After an explanation of study requirements, participant will review and sign the informed consent form.
• Complete the medical history questionnaires
•We will collect a drop of participant blood using finger prick to determine their fasting blood glucose and HbA1c concentrations to make sure they have met the inclusion criteria.
•We will put a small drop of jelly on participant's thigh and use an ultrasound machine to image the participant's vastus lateralis muscle. This will help us make sure that the lidocaine can reach the muscle (i.e., subcutaneous fat over the muscle no greater than 1.5").
• Lastly, you will be familiarized the following set of questionnaires.
Questionnaires 1-6 will be assessed at T1, T2, and T3.
Questionnaires 2-6 will also be assessed weekly during your lab visits.
1.Unintentional weight loss (self-report, ≥10 lbs or ≥5% of body mass in the past year or between major study time points)
2.Fatigue (SF-36 energy subscale)
3.Physical activity (International Physical Activity Questionnaire)
4.24h Diet Recall log (ASA24: Healthy Eating Index 1-100 scale).
5.Sleep quality and well-being (7-point likert scale)
6. Alcohol use (TLFB: Timeline Follow Back)

PRE Testing (Before Phase 1 Begin): All major measurements for the PRE Testing will be completed over 2 separate days within a week. These major measurements will also be conducted at MID (end of Phase 1), and POST (end of Phase 2). However, the sequence of these major measurements will be different.

Preparing for Visits 2 and 3:
1. Overnight fast for 12h (only water allowed)
2. No alcohol for 24h
3. No medications or supplements from this list for 48h:
•Tylenol (acetaminophen)
•Non-steroidal anti-inflammatory drugs (e.g., Ibuprofen)

Visit 2 (Day 1 of the PRE Testing): (~ 4-5h)
1. Hydration assessment: Participant will provide a urine sample so we can assess the hydration status (urine specific gravity, a measure of hydration, must be <1.020). Also, blood sample will be collected for plasma osmolality measurement. In addition, thirst sensation will be assessed using a thirst scale and urine color will be recorded. If participant is not sufficiently hydrated, then they will be given at least 8 oz of water and re-test their hydration after 15 minutes. After 15 minutes, urine sample and thirst sensation will be measured.

2.Anthropometric Measurements:
•We will measure your height, weight, waist-hip ratio, and a body composition scan will be performed using dual X-ray absorptiometry (DXA).

3. Blood pressure:
• We will measure your blood pressure.

4.Muscle Biopsy: We will take muscle sample (~200 mg) from your outer thigh. This procedure will last about 30 minutes.

5.Oral Glucose Tolerance test (OGTT):
• Participants will consume a beverage containing 75 g glucose (TRUTOL, ThermoFisher Scientific).
•Before and at 30 min, 1 hr, and 2 hrs after beverage consumption, venous blood will be collected into vacuum tubes using the standard venipuncture technique.
6. Questionnaires: During the OGTT, you will complete the following set of questionnaires. Questionnaires 2-6 will also be filled out weekly during the study.
1.Unintentional weight loss (self-report, ≥10 lbs or ≥5% of body mass in the past year or between major study time points)
2.Fatigue (SF-36 energy subscale)
3.Physical activity (International Physical Activity Questionnaire)
4.24h Diet Recall (ASA24: Healthy Eating Index 1-100 scale).
5.Sleep quality and well-being (7-point likert scale)
6.Alcohol use (Timeline Follow Back)

Visit 3 (Day 2 of the PRE Testing) (~1.5h)
1. **Hydration assessment:** Participant will provide a urine sample so we can assess the hydration status (urine specific gravity, a measure of hydration, must be <1.020). In addition, thirst sensation will be assessed using a thirst scale and urine color will be recorded. If participant is not sufficiently hydrated, then they will be given at least 8 oz of water and re-test their hydration after 15 minutes. After 15 minutes, urine sample and thirst sensation will be measured.
2. Body weight: Body mass will be measured
3.Physical Function (part of Phenotypic Frailty Assessment) Testing:
•Perform the gait speed test (4-m walk test), 30-second Sit-to-stand test, and handgrip strength test on both hands.
4.Exercise Capacity Test:
•Perform an exercise capacity (VO2peak) test using a cycle ergometer.

Phase 1:

For the Local Heat Therapy Groups

If participants are assigned to one of the local heat therapy(LHT) groups (Sham (CON) and LHT), they will be sent home with 2 heating pads and apply local heat to their thigh 6 days for 12 weeks (5 sessions at home and 1 session at the Lab). Participants assigned to the heating group will be advised to set their heat pads to the temperature previosuly determined to achive an intramuscular temperature increase by 3-4 C; participants assigned to the sham (CON) group will be advised to set their heat pads to a lower tempereature (e.g., 36 C) to maintain a neutral intramuscualr temperature.

Visit 4: Establish heat pad temperature: (1st week of the 12-week LHT intervention)

• During the familiarization visit, we will establish the specific heat pad temperature needed to elevate each participant’s intramuscular temperature by 3-4 °C. Intramuscular temperature sensors will be used to calibrate the heat pad to approximately 3-4°C increase over a 90-minute period. For participants in the LHT group, these measurements will determine the optimal heat pad temperature. To reach the target 3-4 C increase in intramuscular temperature, the heat pad temperature will be increased by 5°C every 5 minutes. If the intramuscular temperature rises beyond 4C after resting, the heat pad temperature will be decreased to maintain the intramuscular temperature by 3-4°C above resting temerpature. After the 90-minute heating session, sensors will remain in the muscle for an additional 60 minutes to monitor temperature changes.

• To monitor skin temperature, one temperature sensor will be placed on the skin approximately 5 cm below the site of the intramuscular temperature sensor to record skin temperature. A second skin temperature sensor will be placed on the contralateral leg in a corresponding location. After securing the thermocouple, a commercially available heat pad will be positioned to surround each of the thighs. To minimize the risk of heat-related skin discomfort or burns, a size-matched flannel cloth will be inserted into the heat pad sleeve as a protective barrier. To ensure secure placement and consistent surface contact, two Velcro straps will be used to secure the heat pad firmly around the upper and lower portion of the thigh.

To measure the intramuscular temperature using intramuscular temperature probe, we will utilize the following published method (Richey et al., 2024). The PIs will receive training from consultant Dr. Steven Romero to ensure accurate technique and proper care throughout the following procedure.

1. The opposite leg from the biopsy site will be used to measure intramuscular temperature.
2. Apply lidocaine under the skin and into the muscle.
3. Put a sterile catheter or sterile 18G needle into the midpoint of the vastus lateralis, then remove the needle from the catheter.

10-1a 4. Insert the thermocouple (IT-18; Physitemp: Type T thermocouple; temperature range: -100 to +150 C/-148F to +300F) through the needle or catheter.

5. Position the thermocouple approximately 1 cm deep into the muscle.
6. Carefully retract the needle or catheter to ensure the thermocouple is correctly positioned

within the muscle.
7. Cover the area with skin-safe adhesive tape (Tegaderm Film) to ensure everything stays in place.
8. Apply heat pad on the thigh for 90 min.
9. Then remove for up to 60 additional minutes (or until the intramuscular temperature returns to baseline, whichever is first).
10. Muscle temperature and body temperature (forehead scanning) will be monitored throughout.
11. Blood pressure will be measured every 10 minutes throughout the 90-min heat pad application.
12. A bandage will be placed on the site of the incision.

o If the intramuscular temperature is above 4°C increase, we will decrease the heat pad temperature by an increment of 1°C until the intramuscular temperature stabilizes at a 4°C. If the skin temperature exceeds 41.5°C, the heat pad temperature will also be reduced by 1°C, and the pad will be loosened to increase airflow and reduce surface heating.

5. Once the intramuscular temperature has increased by 3-4°C above resting, the heat pad temperature will be set and temperature (skin and intramuscular temperatures) will be monitored for the remainder of the session.

o *Note:* We are aiming for an intramuscular temperature increased by 3-4°C. We anticipate that this will be well-tolerated because 1) core temperature remains stable during local heating on the thigh and 2) intramuscular temperatures increased by 3-4°C (vastus lateralis) have been documented in human participants without adverse effects. However, if a participant is unable to tolerate this, we will reduce the initial target intramuscular temperature as needed to a minimum of 38°C based on intramuscular adaptations previously documented in the literature.

At home Local Heat Therapy Sessions (5d/wk):
• Participants will be encouraged to empty their bladder before the start of each session to minimize the number of times they have to remove the heat pads during a session. For any reason they have to remove the heat pad, they will record it.

•For each session, participant will place the heating pads to completely surround each thigh with a snug fit. The heating pads will be secured in place using Velcro and set to the predetermined temperature continuously for 90 minutes.

• Participants will record the following for each session:
1. Start time
2. End time
3. Number of times you need to remove the heat pad during a session
4. The duration for which it is removed
5. Discomfort

•There is no restriction on the location (at home or in the office), time of day, and seated activity during the session (e.g., watching TV, reading a book, etc.).

In-Lab Local Heat Therapy Sessions (1d/wk): Visit 5-15 (~2h each) (1d/wk for the remaining 11 weeks of the 12-week LHT intervention)

•During the weekly lab visit (1d/wk), comfort will be monitored, and we will collect the logs of participants' at-home sessions to confirm compliance.
• Blood pressure will be measured every 10 minutes while the heating pad is applied.
• The following will also be assessed each week: sleep quality and activity (Hooper questionnaire and International Physical Activity Questionnaire [IPAQ]), alcohol use (Timeline Follow-back), 24-hr diet recall (ASA24), and anthropometrics (height, body mass, waist circumference, and hip circumference)
• At weeks 6 and 12 of Phase 1, we will measure both intramuscular and skin temperatures during the in-lab heat pad therapy session.

For the High_Intensity Training (HIIT) Groups

In Lab HIIT Session: Visit 4-39 (Total of 36 visits) (~1h each)

•Participants assigned to the HIIT group will arrive at the laboratory 3 d/wk for 12 weeks. They will be fitted with a heart rate monitor (Polar) and skin temperature sensors (thigh) and will perform a 3-minute cycling warm-up at 30W before beginning the HIIT protocol.
•On the first day of each training week, the following will also be assessed each week: sleep quality and activity (Hooper questionnaire and International Physical Activity Questionnaire [IPAQ]), alcohol use (Timeline Follow-back), 24-hr diet recall (ASA24), and anthropometrics (height, body mass, waist circumference, and hip circumference)
• At weeks 1, 6, and 12 of Phase 1, we will measure skin temperatures during the HIIT session.

MID Testing (End of Phase 1):

The final LHT or HIIT session must be completed 2 days before MID testing.
All major measurements for the MID Testing is identical to PRE Testing including the order of measurements. Detail protocols, please refer to PRE Testing.

Preparing for the following Visits:
1. Overnight fast for 12h (only water allowed)
2. No alcohol for 24h
3. No medications or supplements from this list for 48h:
•Tylenol (acetaminophen)
•Non-steroidal anti-inflammatory drugs (e.g., Ibuprofen)

Visit 16 (LHT)/ Visit 40 (HIIT): Day 1 MID Testing (~4-5h)

1. Hydration assessment
2. Anthropometric measurements
3. Blood Pressure
4. Muscle Biopsy
5. OGTT
6. Questionnaires

Visit 17 (LHT)/ Visit 41 (HIIT): Day 2 MID Testing (~ 1.5h)

1. Hydration assessment
2. Body weight
3. Physical functional tests
4. Exercise capacity test

Phase 2:

Visit 18 - 53 - Phase 2 (Sham (CON) and LHT groups): Participants assigned to the Sham (CON) and LHT groups will continue to Phase 2. In Phase 2, they will complete HIIT 3d/wk for 12 weeks. For detailed protocols, please refer to above (HIIT group).

POST Testing (End of Phase 2):

All major measurements and order of measurements for the POST Testing is identical to PRE and MID Testing. Detail protocols, please refer to PRE Testing.

The final LHT session must be completed 2 days before POST testing.
All major measurements for the MID Testing is identical to PRE Testing including the order of measurements. Detail protocols, please refer to PRE Testing.

Preparing for the following Visits:
1. Overnight fast for 12h (only water allowed)
2. No alcohol for 24h
3. No medications or supplements from this list for 48h:
•Tylenol (acetaminophen)
•Non-steroidal anti-inflammatory drugs (e.g., Ibuprofen)

Visit 54 (LHT): Day 1 POST Testing (~4-5h)
1. Hydration assessment
2. Anthropometric measurements
3. Blood Pressure
4. Muscle Biopsy
5. OGTT
6. Questionnaires

Visit 55 (LHT): Day 2 POST Testing (~ 1.5h)
1. Hydration assessment
2. Body weight
3. Physical functional tests
4. Exercise capacity test

*For Sham (CON) and LHT groups, participants will have a total of **55 Lab visits** with a total time spent for about **81.5h (Lab)** **plus** **90h (at home heat pad therapy)**.*

*For the HIIT group, participants will have a total of **41 Lab visits** with a total time spent for about **50h**.*

*required

What is the total time commitment for the participant?

10-1a(1) For CON (Sham, considered under the "LHT groups" in the consent form) or true LHT group, the duration will be approximately 7 months (testing: 1 month total; Phase 1: 3 months, Phase 2: 3 months) with a total of about 81.5h (lab) plus 90h (at home heat pad therapy). For HIIT group, the participation duration will last for approximately 4 months (testing: 3 wks total; Phase 1: 3 months) with a total of about 50h.

*required

10-1b **Select procedure(s) to be used.**

Check all that apply.

✓ Surveys

*required

10-1b(1) **Check all that apply.**

Online

✓ On-Site

Mailing

Other

*required

10-1b(2) **How much participant time is involved?**

The time commitment for surveys is included in the overall study visit time.

*required

Enter or attach the survey(s) questions or a list of survey items.

If multiple surveys are used, label them separately.

Hooper well-being survey questions (completed weekly; scored on 1-7 scale, low (fatigue, stress, soreness) or good (sleep quality) and 7 indicates high (fa soreness) or bad (sleep quality):

1. Please rate your fatigue level over the past 24 hours.
2. Please rate your level of stress over the past 24 hours.

3. Please rate your level of soreness over the past 24 hours.
4. Please rate the quality of sleep you had last night.

10-1b(3) Automated Self-Administered 24-hour Dietary Assessment Tool (ASA-24; cor
Registration for use of the ASA-24 will be completed upon Notice of Award ar
monies. A link to a demonstration website for the ASA-24 is here: https://asa2
(2024 version)

Unintentional weight loss (at PRE, MID, and POST testing): In the past year [I
12 weeks [MID and POST], have you unintentionally lost body weight? If so, h

Other surveys: see attachments.
1) Medical history (screening visit)
2) International Physical Activity Questionnaire (IPAQ)-SF (weekly)
3) Timeline Follow Back (TLFB, weekly)
4) 36-item Short Form Health Survey (SF-36) energy subscale (weekly; only i
and 31;
scored per https://www.rand.org/health-care/surveys_tools/mos/36-item-short

If you attach a document, state *See Attachment* in the text box above.

IPAQ-SF.pdf

TLFB_Alcoholinstructions.pdf

10-1b(3)a TLFB_ALoverview.docx

SF-36.pdf

Thirst Scale.docx

urine color chart.jpg

R1_11.19_IM_Detail the intervention procedures.docx

*required

Describe the process for the distribution and return of surveys.

10-1b(4) Surveys questions will be uploaded into Research Electronic Data Capture (REDCap), a free, secure, web-based application designed to support data capture. Researchers will assist particiants to electronically complete surveys during their laboratory visits.

Interviews

Focus Groups

Observation

Non-Invasive Procedures (MRI, X-Rays, etc.)
Invasive Medical Procedures (Blood draws, skin pricks, biopsies, etc.)

✓ Intervention

*required

10-1b(25) **How much participant time is involved?**

LHT groups ~81.5h (lab) plus 90h (at home heat therapy) and HIIT group ~50h in the laboratory

*required

10-1b(26) **Where will the intervention(s) take place?**

At home (Sham (CON) and LHT groups) and KSM building (all groups)

*required

Describe in detail the intervention procedures.

10-1b(27)

Attach any documentation if applicable.

See attachment. The LHT group described in the Informed Consent document includes LHT (heat) and CON (control; sham - low-temperature heat pads).

10-1b(27)a If you attach a document, state *See Attachment* in the text box above.

*required

Who will be conducting the intervention(s)? What are their qualifications?

Attach any documentation if applicable.

For Oral Glucose Tolerance Testing (OGTT): Drs. Luk and Levitt are experienced in phlebotomy; Dr. Levitt has carried out the OGTT procedure. Additional study team members are trained in phlebotomy (Casey Appell) and will help carry out this procedure under the supervision of Drs. Luk and Levitt. Jaden Hendrix (research aide) will be primarily responsible for processing biospecimens. Drs. Barr and Hilliard, and the listed graduate and undergraduate students will assist in these procedures as needed.

For body composition measurement, Drs. Luk and Levitt are certified DEXA technicians in the State of Texas and are approved to operate the DEXA housed in the Department of Kinesiology & Sport Management (KSM) at Texas Tech University (TTU). Their students trained on use of the DEXA will carry out this procedure under their supervision. Drs. Barr and Hilliard, and the listed graduate and undergraduate students will assist in this procedure as needed.

10-1b(28)

All biopsy procedures will be performed by Dr. Hui-Ying Luk, who is not a licensed physician but is trained specifically in the muscle biopsy procedure. Robyn Richmond, M.D. (robyn.richmond@ttuhsc.edu) will oversee the procedure (i.e., approve safety protocols, provide technical and medical advice regarding the procedures and potential adverse effects of biopsy). Dr. Richmond will determine the competency of the person who will perform the preparation (sterilized procedures, lidocaine injection) and the operation (maintaining sterile procedure for the primary operator: Dr. Luk). Since the assistant (applying suction through a syringe) does not have direct contact with the participant and does not require sterile procedures, the competency of the assistant will be determined by Dr. Luk. Our members are not going to diagnose these symptoms, instead, if the participant is self-reporting pain, erythema (warmth), or ecchymosis (bruising) to us, we will advise them to use a cold pack. Also, our members will follow up with them regarding the self-reported signs. Similarly, if the participant is seeing and self-reporting signs of infection, inflammation, hematoma (e.g., prolonged redness, pus formation, increasing pain, bruising at the site of biopsy, and fever, etc.), and numbness at the site of biopsy, we will refer them to consult with a medical provider. Lastly, an individual with CPR training (current) will be on-site within close proximity to the subject during procedures.

All intramuscular temperature measurement will be performed by Drs. Hui Ying Luk and Danielle Levitt who are trained specifically in this procedure by Dr. Steven Romero. The proposed technique has been safely and successfully implemented in participants (e.g., PMID: 38059292). Drs. Barr and Hilliard and Casey Appell will assist in this procedure as needed.

For exercise testing and intervention, Drs. Luk and Levitt have experience in exercise testing and intervention and are Certified Strength and Conditioning Specialists (CSCS) through the National Strength and Conditioning Association (NSCA). The proposed exercise capacity testing and HIIT intervention have been safely and successfully implemented in older adult participants (e.g. PMID: 29077855, 28273480). Dr. Luk and Levitt will train and supervise all personnel (graduate and undergraduate students on this IRB application) in conducting the exercise testing and intervention included herein. Only study personnel with currect CPR and first aid training will perform exercise testing and intervention. Dr. Levitt has an up-to-date CPR and first aid certification, and has held valid CPR

certification continuously since 2003. Drs. Barr and Hilliard, and the listed graduate and undergraduate students will assist in these procedures as needed.

For frailty risk assessment, Dr. Levitt has assessed frailty risk in people living with HIV. She will train and supervise all personnel (graduate and undergraduate students on this IRB application) in conducting the physical function testing to assess frailty risk. Drs. Barr and Hilliard, and the listed graduate and undergraduate students will assist in these procedures as needed.

If you attach a document, state *See Attachment* in the text box above.

10-1b(28)a

incidents updated (1) (2) (2) (2) (1).docx

IM temp and heat therapy in older adults.docx

Other

Deception

10-2

The result of investigators providing false or incomplete information to participants for the purpose of misleading research subjects.

*required

10-2a **Does this research project involve the use of deception?**

✓ Yes

*required

10-2a(1) **Explain why deception is necessary.**

A sham group is needed for scientific purposes.

No

10-3 Debriefing

10-3a **Will a debriefing script be used at the conclusion of the research?**

✓ Yes

*required

10-3a(1) **Select the reason for needing a debriefing script.**

✓ Deception

Sexual Misconduct

Mental Health, Self-harm, Substance Abuse, Criminal Behavior

✓ Education Tool

*required

10-3a(2) **Enter or attach a copy of the debriefing script.**

See attachment

If you attach a document, state *See Attachment* in the text box above.

10-3a(2)a Biopsy care kit.doc

Deception Script.docx

IM care kit.doc

*required

Provide information on who will conduct the debriefing and how it will be delivered to the participant.

Dr. Luk, Dr. Levitt, or clinical coordinator (future) will conduct the debriefing.

Upon the completion of the biopsy procedures, we will provide a take-home kit (i.e., notes and necessary consumables, e.g., extra bandage, alcohol

10-3a(3) wipes). Also, we will verbally explain the biopsy care instructions and a list of drugs that can increase bleeding (see the attachment), especially focusing on the medication that should be avoided. Furthermore, our research team will follow up with the participant in the following two days by text/call to make sure no adverse effect was observed (swelling and warmth at the site of biopsy). You can also send us a photo of the biopsy site through email or text message if you have any concerns. We will look at your biopsy site on your next visit.

For the control group (CON), we will disclose that the temperature setting for the heat pad used during the study was for control purposes, and we will specify the temperature they need to set to achieve an intramuscular temperature of 40 degrees Celsius.

No

10-4 TTU Compliance Committees

Additional approvals

*required

10-4a Does this research require internal approval from another TTU compliance committee or entity?

✓ Yes

10-4a(1) Select the TTU Committee(s) that grants approval to conduct research at that location and attach the approval letter.

Texas Tech Neuroimaging Institute (TTNI)

✓ TTU Institutional Biosafety Committee (IBC)

*required

Attach the approval letter.

Luk_IBC.pdf

IBC-2023-1083 approved v9.pdf

2023-2025_KSM_DXA_Sublicense.pdf

10-4a(1)b

Luk_DEXA training.pdf

Luk_Handling Radioactive Material.pdf

Luk_Introduction to Radiation Safety.pdf

Luk_Radiation Safety.pdf

Luk_X-Ray Safety.pdf

✓ TTU Institutional Laboratory Safety Committee (ILSC)
*required

Attach the approval letter.

10-4a(1)c

Luk_IBC.pdf

IBC-2023-1083 approved v9.pdf

2023-2025_KSM_DXA_Sublicense.pdf

Luk_DEXA training.pdf

Luk_Handling Radioactive Material.pdf

Luk_Introduction to Radiation Safety.pdf

Luk_Radiation Safety.pdf

Luk_X-Ray Safety.pdf

Institutional Animal Care and Use Committee (IACUC)

✓ TTU Radiation and Laser Safety Committee (RLSC)
*required

Attach the approval letter.

10-4a(1)e

Luk_IBC.pdf

IBC-2023-1083 approved v9.pdf

2023-2025_KSM_DXA_Sublicense.pdf

Luk_DEXA training.pdf

Luk_Handling Radioactive Material.pdf

Luk_Introduction to Radiation Safety.pdf

Luk_Radiation Safety.pdf

Luk_X-Ray Safety.pdf

Other

No

11: Privacy, Confidentiality & Security

Complete the following section.

11-1 Personal Identifiers

*required

11-1a Select personal identifiers that will be collected for the purpose of this research.

Check any identifier you will be in possession of during the research process.

- ✓ Names
- ✓ Telephone numbers
- ✓ Any elements of dates (other than year) for dates directly related to an individual, birth date, admission date, discharge date, date of death. For ages over 89 all elements of dates (including year) indicative of such age, expect that ages and elements may be aggregated into a single category of age 90 and older
- Any geographic subdivisions smaller than a state, including street address, city, county, precinct, zip code and their equivalent geocodes except for the initial three digits of a zip code
- Fax numbers
- ✓ E-mail addresses
- Social security number
- Medical record number
- Student ID username/number
- Health plan beneficiary numbers
- Account numbers

Certificate/license numbers

Vehicle identifiers and serial numbers (VIN), including license tag identification

Device identifiers and serial numbers (e.g., implanted medical device)

Web universal resource locators (URLs)

Internet protocol (IP) address numbers

Biometric identifiers, including finger and voice prints

Full face photographic images and any comparable images (i.e., videos, etc.)

✔ Biological specimens

Any other unique identifying number, code, or characteristic, other than dummy identifiers that are not derived from actual identifiers and for which the re-identification key is maintained by the health care provider and not disclosed to the researcher

No personal identifiers will be collected

11-2 **Privacy of Participants**

---

*required

11-2a **Describe how you will protect the privacy of the participants.**

Consider how you will protect the participant's privacy during:

- Recruitment
- Consent
- Data Collection
- Storage of Data
- Analysis of Data
- Findings

Data Safety:
To protect against breach of confidentiality, any hard copy materials (e.g., informed consent form, any hard copies of exercise records with heart rate and rating of perceived exertion

data, etc.) will be kept in a locked drawer in Dr. Luk's private office. The informed consent form and demographics files will be the only places participants' names appear, and only the PIs and essential study personnel will have access. Participant samples, data, and records will be deidentified and labeled using a unique ID number. Data, images, and files generated from the project will always be stored in raw form, separate from files with calculations and analyses. Lastly, all electronic material, including data from questionnaires administered using computer-assisted interview techniques (i.e., no hard copy) will be stored in a secure database (REDCap).

11-3 **De-Identifying Data**

Anonymous v. Identifiable Data

*required

11-3a **Explain how you will de-identify the data collected. If data will be given a code, explain this process.**

For example:
Personal identifiers will be removed from the data and replaced with a participant code. The participant code will be used on the individual's data. The master list containing the personal identifier(s) and participant code will be in a separate document stored away from the data. The master list and data will be password protected. The passwords will be different for the master list and data.

For each identifier explain when and how you will code/de-identify/destroy the identifier.
Consider the following:

- What identifiers are associated with data?
- What identifiers are NOT associated with the data?
- What descriptors can be used to identify someone? For example: schools, towns, buildings, colleagues, companies, titles, etc.

Upon participant's admission to the study, a unique study identification code (ID codes) will be assigned to the participant in the order they are enrolled. The file containing participants' names and IDs will be stored electronically in REDCap.

Thereafter, all data will be kept in coded participant files. All trained investigators, professional staff, and technicians are aware of the confidentiality involved with this study.

Identifiable data will not be available or divulged to anyone outside of the experimental research team and data files will be kept for at least 3 years after the study is terminated. The confidentiality of individual information will be maintained in any publications or presentations regarding this study.

11-4 **Data Transcription**

*required

11-4a **Select who will transcribe audio or voice recording.**

Check all that apply.

Member of the research team/lab

Professional transcription company

Other

✓ Not applicable

11-5 **Transfer of Data**

*required

11-5a **Will data be transferred from one location to another?**

Transfer of data can be done electronically (email, removable storage device, shared drives, remote access, hyperlinks, etc.) and/or physically (carrying, mailing, etc).

✓ Yes

*required

Explain how it will be securely transported and by whom.

1. Data type (human subjects data only)

A. **Types and amount of scientific data expected to be generated in the project:**
Data from human subjects: Demographic and anthropometric (e.g., age, sex, race, ethnicity, body mass, body composition, height), clinical (e.g., medical history, oral glucose tolerance test parameters, and questionnaires about alcohol use, diet, activity, sleep, changes in body mass, and fatigue), functional (e.g., exercise capacity, grip and leg strength, gait speed), and molecular (e.g., type I and II muscle fiber cross-sectional area, capillarization, mitochondrial enzyme activity, mitochondrial and TRPV1 protein expression, mitochondrial DNA quantification, and mitochondrial content and morphology) data will be collected from 54 older adults at risk for type 2 diabetes (described in detail in the “Human Subjects” attachment) at several time points throughout the study. Weekly assessments will include body mass measurement, diet and alcohol questionnaires, and activity and sleep monitoring via and self-report questionnaires. All other data will be collected only at the beginning of the study (i.e., demographics, medical history) or at the 2-3 major time points. Participants assigned to the high-intensity interval training (HIIT) group (n=18) for phase 1 will have 2 major time points (before [T1] and after [T2] phase 1). Participants assigned to the LHT (n=18) and CON (n=18) groups for phase 1 and HIIT for phase 2 will have 3 major time points (before phase 1 [T1], between phases 1 and 2 [T2], and after phase 2 [T3]).

Large-scale data sets: It is possible that large scale mitochondrial genomic data (human participants) and proteomic data (cell culture) could be generated from this project if alternative approaches must be used as described in the research strategy.

The total amount of anticipated data if alternative approaches are not needed is ~25 GB of data.

B. **Scientific data that will be preserved, shared, and the rationale for doing so:** All raw data generated from this project will be stored in a password-protected manner on cloud-based servers at Texas Tech University. Cleaned data in an analysis-ready format will be stored in REDCap and periodically uploaded to repositories. Data of sufficient quality will be uploaded to allow others to validate and replicate research findings described in the Aims.

C. **Metadata, other relevant data, and associated documentation**: In alignment with the Data Submission Worksheet for the Aging Research Biobank, the following will be submitted to accompany data derived from human subjects:
•Full study protocol
•Names and descriptions of data sets
•Manual of operations

•Annotated data collection forms
•Data dictionary
•Documentation of calculated variables
•Summary of deidentification and crosslinking of study ID with new randomized ID
•Any changes made to the protocol over time
•Frozen datasets used for the primary publication, when applicable
Similarly, the full study protocol, dataset names/descriptions, data dictionary, documentation of calculated variables, and any changes to the protocol over time will be deposited in TTU Dataverse for molecular procedures.

2. **Related tools, software, and/or code:** Statistical analyses will be run using standard statistical software (SPSS version 29) and graphs made using GraphPad Prism (Version 9). Image processing will be performed using ImageJ and in part using custom Python code to automate quantification where appropriate.

3. **Standards:** No consensus standards of data formatting such as Dublin or Darwin core exist for our discipline. Data dictionaries defining each variable and decoding any coded responses will accompany all uploaded data.

4. **Data preservation, access, and associated timelines:**
A. Repository where scientific data and metadata will be archived:
Human subjects data: NIA's Aging Research Biobank
Molecular data: TTU Dataverse Collection
Mitochondrial genomic data: Database of Genotypes and Phenotypes
11-5a(1) (dbGaP; if performed)
Custom code: GitHub

B. How scientific data will be findable and identifiable: Data will be findable for the research community through the repositories listed above. Collections will be established in the Aging Research Biobank and in Dataverse when this application is funded. Collections will be established on dbGaP should a large data set be generated. Data stored in the Aging Research Biobank will be made available by reasonable request, and this will be noted in the "Data Availability Statement" in associated publications. When data are made public via Dataverse, a digital object identifier (DOI) will be assigned. This DOI be referenced in associated publications to allow the research community access to the data used in each publication. If dbGaP is used, researchers can submit requests to dbGaP to access and browse data, and this information will be included in any associated publications. Any custom code will be shared on GitHub, accessible by searching "rbudnar" on GitHub. The readme.md file for the project will include instructions for the analyses and rationale for parameter choices.

C. When and how long the scientific data will be made available: Data associated with each publication will be made available when preprints are available and will continue to be available on the repositories and long as

they are supported. Any data that are not yet available by the end of the award period will be made available at that time.

5. Access, distribution, or reuse considerations:
A. Factors affecting subsequent access, distribution, or reuse of scientific data: Broad data sharing will be included in the informed consent form. Data deidentification, linking data using unique identifiers, protection of privacy, and making data available will be explained to participants and any questions answered as part of the informed consent process.

B. Whether access to scientific data will be controlled: Access to human subjects data will be controlled. Requests will be submitted through the appropriate repository (Aging Research Biobank, Dataverse, dbGaP).

C. Protections for privacy, rights, and confidentiality of human research participants: The only location that personal information will be paired with participant ID will be on a single administrative data collection form in the secure REDCap database. This is necessary because data will be collected from the same participants and multiple time points. However, each participant will be assigned an ID code and only the ID code will be linked to data so that the data are not identifiable. Data from human participants will be further deidentified upon submission to the repository(ies) and linked only through the study ID. The Aging Research Biobank further anonymizes data by randomly generating a new ID for each subject. dbGaP interfaces with the Aging Research Biobank so clinical and mitochondrial genomic data (if generated) can be linked, using only the randomly generated ID. Further, access to these datasets will be controlled and require that users register and submit reasonable requests. The PIs attest that no attempt will be made to reidentify participants from deidentified data.

6. Oversight of DMS: Drs. Hui-Ying Luk and Danielle Levitt (PIs) will deposit clinical and associated data into the NIA Aging Research Biobank, TTU Dataverse, and into dbGaP if mitochondrial genomic data are generated. Data upload will occur when publications associated with each data set are submitted and made public (where applicable) upon acceptance. Data sharing will be included in the annual progress report.

7. Representatives of the following people/groups within *Texas Tech University* may use your study records and share them with other specific groups in connection with this research study.
• The principal investigators (*Hui Ying Luk, Danielle Levitt*)
• The *Texas Tech University* Institutional Review Board
• The *Texas Tech University* Human Subjects Protection Office
The above people/groups may share your unidentifable study records with the following people/groups outside *Texas Tech University* for their use in connection with this research study. These groups, while monitoring the research study, may also review and/or copy your original *Texas Tech University* records.

• The Office of Human Research Protections in the U. S. Department of Health and Human Services.
• The Safety Officer appointed for this study.
• The National Institutes of Health and its authorized representatives.

No

11-6 **Certificate of Confidentiality**

*required

11-6a **Has a Certificate of Confidentiality been requested?**

Yes

✓ No

11-7 **Investigator Assurances**

*required

11-7a **Data Security**

✓ I will protect the data through at least one of these security measures: encryption software, password protection, and /or physical barrier (locked doors, file cabinets, etc.).

I will **NOT** protect the data through at least one of these security measures: encryption software, password protection, and /or physical barrier (locked doors, file cabinets, etc.).

*required

11-7b **Access to Data**

✓ I will only allow the research team access to the identifiable data.

I will allow others outside of the research team to have access to identifiable data.

*required

11-7c **Destruction of Personal Identifiers**

✓ I will destroy personal identifiers prior to closing the IRB with the exception of the consent forms.

I will NOT destroy personal identifiers prior to closing the IRB with the exception of the consent forms.

*required

11-7d **Retention Policy**

✓ I will keep de-identified data and/or consent forms for at least 3 years following the closure of the IRB.

I will NOT keep de-identified data and/or consent forms for at least 3 years following the closure of the IRB.

*required

11-7e **Data Repository**

✓ I will post the de-identified data to a data repository.

I will **NOT** post the de-identified data to a data repository.

12: Benefits & Risks

Please respond to the following statements/questions to help our IRB reviewers recognize the benefits and risks associated with this study.

12-1 **Benefits**

*required

Describe the potential direct benefit(s) to subjects.

Payments to subjects, course credit or contribution to science should not be included in this section.

12-1a

All efforts will be made throughout the study to minimize any study-related risks to subjects. The Physical Activity Guidelines for Americans, 2nd Edition (2018), suggest that adults should engage in at least 150 minutes per week of moderate-intensity aerobic exercise (or an equivalent 75 minutes per week of vigorous-intensity aerobic exercise, including HIIT) to maximize the many health benefits of aerobic exercise. Even if people do not meet the minimum of 150 minutes, smaller amounts of exercise are still beneficial. Participating in this study will provide participants with a structured environment to help meet that exercise goal during one of the two phases. Among anticipated benefits from exercise, we emphasize that there might be metabolic health benefits and decreased frailty risk, particularly because participants will be prediabetic older adults, indicating risk for the development of type 2 diabetes and frailty.

Furthermore, benefits of heat therapy are becoming increasingly recognized and we anticipate that participants assigned to the local heat therapy group will have improved skeletal muscle health, glycemic control, and decreased frailty risk. Because of the benefits anticipated from local heat therapy, all participants will be provided with heating pads regardless of group. The HIIT group will be provided with heating pads and instructions for use at the end of their participation (T2). The control group will be provided with heating pads but instructed to use a lower heat setting T1. At the end of their study participation (T3), they will be provided with instructions for use. Regardless of group, participants will have regular interaction with members of the study team. Together with exercise, prosocial interaction may have psychological benefits.

Participants will be provided with the following non-diagnostic results from testing performed at each major time point (PRE, MID, and POST):

- Dual-energy X-ray absorptiometry (DEXA) reports: Body composition and bone mineral density compared to age- and sex-specific norms.
- Body mass index (BMI): Value calculated from body mass and height (kg/m^2) and BMI category (i.e., underweight, normal weight, overweight, obese).
- Gait speed: 4-m walk time compared to age- and sex- specific norms.
- Muscle strength: Average grip strength (3 trials per hand) and 30-second sit-to-stand test with comparison to age- and sex- specific norms.
- Exercise capacity: VO_{2peak} and comparison to age- and sex-specific norms.
- Blood sugar control: If fasting blood sugar, HbA1c, or 2-hr glucose values after the glucose tolerance test fall into the diabetic range at any time point, the participant will receive a letter advising you of the laboratory measurement and to follow up with your physician.

12-2 Risks

*required

Describe any potential risk to subjects and how you will minimize that risk.

While exercise and heat have many potential benefits, they always carry possible risks. These risks are minimal, and the benefits of exercise and heat therapy largely outweigh the risks of a sedentary lifestyle. Those with higher-than-average risk for complications of exercise (e.g., preexisting cardiovascular conditions) will be excluded from the study. Risks of sample collection are also minimal, and the precautions taken will further offset these risks. The qualifications of the PIs and the protections in place for human subjects will ensure a safe environment for study participation.

Protections against risk
Strategies for minimizing risks associated with medical history and questionnaires, collection of biological specimens, and exercise are described below. All procedures will be fully approved by the TTU IRB. Drs. Luk and Levitt are trained in phlebotomy and muscle biopsies techniques and have previously conducted these procedures in IRB-approved studies. Dr. Luk is currently approved and actively performing muscle biopsies at TTU, and Dr. Levitt is in the process of obtaining such approval. All study personnel will be required to maintain valid CPR and First Aid certifications, and emergency contact information will be kept on hand. All personal information and data will be kept in a secure database (REDCap).

Protection against and minimization of risks by procedure:
•Interviews and completing questionnaires: To minimize risks of emotional stress and fatigue, the length and number of questionnaires will be kept to a minimum, and the study team will establish rapport with participants to minimize emotional stress. Contact information for

guidance counselors will be available. Protection against breach of confidentiality will be ensured by keeping all physical study materials in locked, secure locations and all electronic material in a secure database (REDCap). Except for the demographics form, all data will only be linked with unique identifiers and not with any personally identifiable information. Data uploaded to repositories will have different randomly generated unique identifiers generated during upload to the NIA Aging Research Biobank that are different from those used in REDCap or on study forms.

•Blood collection: Participants will be informed of the risks of venipuncture and finger prick. Risk for infection is low with the use of sterile technique, and a sterile adhesive bandage will be applied to the blood collection sites. Blood collection will be performed by trained personnel using standard laboratory techniques: disinfecting the site with alcohol, using sterile disposable supplies, and applying pressure with sterile gauze to minimize bleeding and bruising. Syncope is possible, so phlebotomy procedures will be performed using phlebotomy chairs in case legs need to be elevated. Cold packs will be available. Participants will be asked if they have a history of fainting prior to blood collection.

•Muscle biopsy: The following are risks that are related to the muscle biopsy procedure and are only associated with/around the incision site: Pain, bleeding, excessive redness, bruising, tenderness, swelling, warmness of the skin, localized numbness, and pink-tinted drainage from the incision. In extreme cases, a low-grade fever may occur, which would indicate that the site may be infected. None of these complications are usually life-threatening, and most complications can be managed conservatively without hospitalization.

To minimize risks, muscle biopsies will be performed using standard sterile technique. The skin over the vastus lateralis will be cleaned using povidone-iodine and the area anesthetized using 1% lidocaine without epinephrine. A small incision will be made through the skin and fascia using a single-use sterile scalpel (#11). A sterilized Bergstrom biopsy needle will be inserted into the muscle and approximately 200 mg of muscle will be collected. After the sample has been collected, pressure will be applied to the area for 5 min. The skin will be closed with a sterile adhesive bandage, covered using sterile gauze, and compression wrapped. Each participant will be given verbal and written instructions for biopsy care and extra supplies (e.g., sterile adhesive bandages, alcohol pads) will be provided. The study team will follow up with each participant 2 and 5 days following their biopsies. Sterile supplies (gloves, gauze, scalpel, needles, etc.) will be used and the PIs will train personnel to assist in the procedure. In case a participant becomes lightheaded or feels faint, we will provide food or beverages containing simple sugar (e.g., juice).

• Intramuscular temperature measurement: There may be some discomfort during the insertion of the probe into your muscle. The topical anesthetic will numb the insertion area to minimize this discomfort, but you may feel pressure or a dull ache in the muscle as the needle moves through the muscle. At the end of the session, the probe will be withdrawn and a sterile dressing will be applied. Any swelling or redness after the study should be gone a few hours after completion of the study, but you may feel some muscle soreness for several days. Although the small probes are sterile, there is a slight risk of infection at the sites where the probes were placed. You will be instructed on how to keep the area clean
12-2a for a day or two following the study and will need to inform the researchers immediately

if you have any redness or swelling in the area. To minimize risks, this measurement will be performed using standard sterile technique. The skin over the incision site will be cleaned using povidone-iodine and the area anesthetized using topical anesthetic cream. At the end of the session, the probe will be withdrawn and the skin will be closed with a sterile adhesive bandage, covered using sterile gauze, and compression wrapped. Each participant will be given verbal and written instructions for care and extra supplies (e.g., sterile adhesive bandages, alcohol pads) will be provided. Sites will also be re-assessed at the next study visit.

•Anthropometric measurements: To minimize risks associated with fatigue, these procedures will be performed near a wall or other support and chairs kept nearby in case participants need to rest between measurements. Contact information for guidance counselors will be available.

•Dual-energy x-ray absorptiometry (DEXA): DEXA is considered a low-risk procedure. However, the use of x-rays, even in low amounts, does present some risk associated with ionizing radiation. To minimize these risks, the number of DEXA scans will be kept to a minimum.

•Functional tests, exercise capacity (VO2peak) testing, and HIIT: During screening, we will ensure that participants meet ACSM medical criteria for initiating an exercise program and do not have medical complications that would put them at undue risk (e.g., diabetes, renal disease, uncontrolled hypertension, other cardiovascular disease, etc.). Participants may become faint, nauseous, experience muscle strains or soreness, or fall. To minimize risk associated with physical activity, participants will be counseled on the importance of proper hydration and will regularly be asked about their physical condition and adjustments made as necessary. Chairs will be available for rest during functional testing, and a researcher will remain close by the participant in case they lose their balance. During VO2peak testing and HIIT, we will utilize cycle-based exercise instead of treadmill exercise to prevent the risk of falls while walking, and participants will be helped on and off the cycle ergometer or exercise bicycle as needed. Participants will perform proper warm-ups and cool downs. We will also monitor heart rate and rating of perceived exertion during exercise. The VO2peak test will be performed in 3-minute stages with predetermined power output (i.e., males start at 50W, females start at 25W, and power increases by 20W every 3 minutes). If the participants cannot maintain the power output for a given stage, their cycling cadence falls below 50 rpm for two consecutive minutes, or they ask to stop the test, the test will end. In case a participant becomes lightheaded or feels faint, that day's exercise will be stopped and we will provide food or beverages containing simple sugar (e.g., juice).

•Local heat therapy: The potential risks of using local heat therapy include discomfort, skin redness, and, in rare cases, burns, particularly if used improperly or for extended periods. Core temperature increases are not typically observed with local heating on the thigh and thus is not considered a potential risk for the proposed study. However, we will monitor body temperature using a forehead scanner. If the body temperature reaches 40°C, we will stop the heat pad therapy. The risk of discomfort will be minimized by determining the lowest optimal heat pad temperature prior to the onset of the heat therapy intervention and that temperature will not be exceeded. Further, participants will be provided with contact information for the PIs should any problems occur during their at-home local heat therapy

sessions and will be asked about any discomfort during their weekly check-ins. Sometimes, redness may appear on your skin in a pattern after the heat therapy session, but it should fade within a day. Heat therapy sessions will occur in the laboratory once weekly and will be monitored by study personnel to ensure that no problems occur during heating sessions. The risk of burns due to personal heating pad use in older adults is not well-documented. However, in a 2021 study (PMID: 33031521), a review of burn injuries in older adults (age 65+) registered in the National Electronic Injury Surveillance System was performed. For adults aged 65-84 years, heating pads were not in the top 5 causes of burn injuries. In the 85+ year-old age group, heating pads were in the top 5 causes of burn injuries. An estimated 204 burns across 100 emergency departments in the United States over a 10-year period were attributable to electric heating pad use over a ten-year period, and all were in women. The risk of burns due to heating pads in this age group is increased by chronic comorbidities that are exclusionary for this study (i.e., diabetes, peripheral vascular disease, chronic kidney disease, neuropathy), minimizing risk. The risk also increases if heating pads are used while sleeping, so we will instruct participants to set a timer and/or alarm to ensure they do not exceed the 90-minute application time, also minimizing risk. Finally, it is estimated that a surface temperature between 42-43°C would produce a burn injury in 12-20 hours (PMID:1885636). Because the difference between skin and muscle temperature is approximately 2°C across a variety of studies, we anticipate that the heating pad setting to produce an intramuscular temperature of 40°C will be approximately 42°C. The 90-minute application time is far below the conservative end of the estimated contact time frame to produce a burn injury (12 hours).

12-2a(1) **Is a liability plan offered?**

Yes

✓ No

13: Participant Compensation

13-1 Compensation - Payments to Research Participants

Compensation through TTU funds must comply with TTU OP 62.25.

*required

13-1a **Are subjects being compensated for their participation in this study?**

✓ Yes

*required

Describe the compensation process.

13-1a(1)

Participant will be paid via cash upon completion of each visit:

Consent/screening ($10)

Major Measurement Testing (PRE, MID, POST):
•Muscle biopsy ($50) and OGTT/Questionnaires ($50)
•Physical function and exercise capacity tests ($50)
The major time point testing will be conducted twice for the HIIT group and three times for Local Heat groups.

For each laboratory visit, participants will be compensated $15.
Phase 1:
•LHT: 12 visits = $180
•HIIT: 36 visits = $540

Phase 2:
•LHT: 36 visits = $540

Total:
•LHT: $1180
•HIIT: $850

13-1a(2) **Select the payment type used.**

✓ Cash

*required

Amount of Payment

13-1a(2)a

Amount will be paid according to the completion of each visit, please see above for details. Participants who are assigned to the LHT groups and complete each visit in its entirety will receive a total of $1180. Participants who are assigned to the HIIT groups and complete each visit in its entirety will receive a total of $850.

*required

13-1a(2)b

When and how will the participant be paid?

payments in cash will be disbursed after each visit

Check

Gift Card (not to be checked if a drawing is involved)

Drawing

Course Credit

Other (includes payment or credit from 3rd party affiliations (i.e., MTurk, SSI, etc.)

Investigator Assurances

Guidance: The Institutional Review Board (IRB) should determine that the risks to subjects are reasonable in relation to anticipated benefits [21 CFR 56.111(a)(2)] and that the consent document contains an adequate description of the study procedures [21 CFR 50.25(a)(1)] as well as the risks [21 CFR 50.25(a)(2)] and benefits [21 CFR 50.25(a)(3)]. It is not uncommon for subjects to be paid for their participation in research, especially in the early phases of investigational drug, biologic or device development. Payment to research subjects for participation in studies is not considered a benefit, it is a recruitment incentive. Financial incentives are often used when health benefits to subjects are remote or non-existent. The amount and schedule of all payments should be presented to the IRB at the time of initial review. The IRB should review both the amount of payment and the proposed method and timing of disbursement to assure that neither are coercive or present undue

influence [21 CFR 50.20].

13-1a(3)

Any credit for payment should accrue as the study progresses and not be contingent upon the subject completing the entire study. Unless it creates undue inconvenience or a coercive practice, payment to subjects who withdraw from the study may be made at the time they would have completed the study (or completed a phase of the study) had they not withdrawn. For example, in a study lasting only a few days, an IRB may find it permissible to allow a single payment date at the end of the study, even to subjects who had withdrawn before that date.

While the entire payment should not be contingent upon completion of the entire study, payment of a small proportion as an incentive for completion of the study is acceptable to FDA, providing that such incentive is not coercive. The IRB should determine that the amount paid as a bonus for completion is reasonable and not so large as to unduly induce subjects to stay in the study when they would otherwise have withdrawn. All information concerning payment, including the amount and schedule of payment(s), should be set forth in the informed consent document.

---

*required

13-1a(3)a **Participant Payment**

✓ I will provide compensation to all participants who complete the consent process and begin the study, regardless of whether they complete the study.

I will **NOT** provide compensation to all participants who complete the consent process and begin the study, regardless of whether they complete the study.

---

*required

13-1a(3)b **TTU OP 62.25**

All questions concerning participant payment should be directed to the TTU Payroll & Tax Services office or the researcher's business manager.

✓ I will follow the procedures outlined in TTU OP 62.25 regarding participant payment.

I will **NOT** follow the procedures outlined in TTU OP 62.25 regarding participant payment.

No

Conflict of Interest

Guidance: A conflict of interest refers to a situation in which an employee(s) financial, professional, or other personal considerations may directly or indirectly affect, or have the appearance of affecting, the employee(s) judgment in exercising any duty or responsibility, including the conduct or reporting of research, owed to the institution (TTU OP 10.20 (2)(d)).

14-1

"Significant business or financial interest" means anything of monetary value including, but not limited to, salary or other payments for services (e.g., consulting fees or honorarium; equity interests (e.g., stocks, stock options, or other ownership interests); and intellectual property rights (e.g., patents, copyrights, and royalties from such rights). (TTU OP 74.17).

"...a set of conditions in which an investigator's judgment concerning a primary interest (e.g., subject welfare, integrity of research) could be biased by a secondary interest (e.g., personal or financial gain)." Institutional Review Board Management and Function, Bankert and Amdur, 2006, p. 167.

*required

14-1a **Does anyone on the research team have a potential conflict of interest with the research project?**

Yes

✓ No

15: Investigator Attestations

*required

I, as the PI and co-investigators, understand Texas Tech University's policy concerning research involving human subjects and by checking below, I certify:

- ✓ I have read The Belmont Report "Ethical Principles and Guidelines for the Protection of Human Subjects of Research" and subscribe to the principles it contains.

- ✓ I have read 45 CFR 46, Protection of Human Subjects and subscribe to the regulations it contains.

- ✓ I accept responsibility for the scientific and ethical conduct of this research study and understand my responsibility.

- ✓ I will ensure that all study personnel are appropriately trained and are competent to perform the study.

- ✓ I will obtain prior approval from the Institutional Review Board (IRB) before making any modifications of the previously approved research, including modifications to the informed consent process and document.

- ✓ I will submit a Study Closure Submission upon completion of this study and agree to honor any other commitments.

✓ I will follow TTU procedures and guidelines to protect the health and well-being of research participants, research team members, and liaison(s) during any unforeseen circumstances.

✓ I will immediately report to the IRB any deviations, violations, non-compliance, unanticipated problems, and adverse events related to subjects or others which occurred or possibly occurred as a result of this study.

45 CFR 46.103(a) and TTU IRB Policy 5.4 require Adverse Events, Unanticipated Problems and Deviations from the research protocol to be reported **IMMEDIATELY** to the HRPP office.

Human Research Protection Program
Box 41075
Lubbock, Texas 79409
Phone: (806) 742-2064
Email: hrpp@ttu.edu

16: Additional Information

16-1 **Include any additional points to help clarify the research, if applicable.**

16-1a

16-2 **Enter or attach any additional documentation.**

16-2a

Modification Submission

Modification

*required

Cayuse IRB Modification:

Provide a summary of the changes you are requesting in the text box below. Details of the changes are required in the appropriate sections. The "Compare" feature is a useful tool as you edit each section.

Summary of Changes.

We have received approval from the Program Officer to lower the age criterion from 60 to 50 years and older. For women, eligibility additionally requires postmenopausal status. Accordingly, we have updated the inclusion criteria to reflect the revised age requirement and explicitly note that women must be postmenopausal.

*required

Current Research Activities

Check all that apply.

✓ Research procedures have **NOT** started.

✓ Participants are being recruited for this research.

*required

Explain the recruitment procedures that have started.

We have pre-screened potential participants and completed three consent/screening visits; however, none met the inclusion or exclusion criteria.

Recruitment has ended.

Participants have been consented to do the research.

Participants have **NOT** been consented to do the research.

Data collection is **ONGOING.**

Data collection is **COMPLETE.**

Analysis of **IDENTIFIABLE** data.

Analysis of **DE-IDENTIFIED** data.

1: Human Subject Research

Answer the following questions to determine if you need IRB review and approval.

1-1 Research

*required

Research is defined by 45 CFR 46.102(l) as:

A systematic investigation, including research development, testing and evaluation, designed to develop or contribute to generalizable knowledge.

Does your proposed study meet the definition of research?

✓ Yes

No

1-2 Human Subjects

*required

Human Subject is defined by 45 CFR 46.102(e) as:

A living individual about whom an investigator (whether professional or student) conducting research

- Obtains or receives private identifiable information or identifiable biospecimens about the individual(s)
- Obtains information or biospecimens through intervention or interaction with the individual(s)

Does your proposed study involve and meet the definition of human subjects?

✓ Yes

No

If you answered YES to all questions in Section 1 complete the rest of the sections.

2: Review Type

Complete the following section.

*required

2-1 **Review Type**

Select the appropriate review type.

Exempt

Expedited

✓ Full Board

The project involves greater than minimal risk to human subjects.

Relying on IRB approval from another institution (IAA)

2-2 **External Collaboration**

Only select collaboration if there are Non Texas Tech researchers, employees, and/or students working on this research.

Collaboration

2-3 **International Research**

Research will be conducted outside of the United States of America and/or data will be shared across borders.

International Research

Complete the following section.

Lay Summary

*required

Provide a description of your study in vernacular language.

Avoid the use of technical terms, undefined acronyms, or excessive scientific terminology.

Aging people with prediabetes are at increased risk for frailty, and skeletal muscle architectural factors (which may improve with exercise or heat therapy) could underlie development of frailty and progression to type 2 diabetes. The proposed study will test local heat therapy as a method to improve skeletal muscle architecture (i.e., cross sectional area, capillarization, mitochondria), glucose tolerance, frailty indicators, and exercise adaptation in prediabetic older adults. Data generated will provide evidence supporting a directly translatable, easily implemented therapeutic intervention to ameliorate risk of developing overt type 2 diabetes and frailty in at-risk aging individuals.

Complete the following section. List all researchers involved with recruitment, data collection, and analysis.

NOTICE: HUMAN SUBJECT TRAINING REQUIREMENT

All research study personnel who engage with participants will need to complete human subject training prior to IRB approval and prior to conducting any human research-related activities. The new training requirement will be applied when an existing study is due for renewal, requires modification(s), or is chosen for a post approval monitoring.

Human research-related activities include recruitment of participants, consenting of participants, data collection (anonymous or identifiable), gaining access to identifiable data, and/or conducting analysis of identifiable data. These training requirements will apply to all Principal Investigators, Co-Investigators, and research staff/students. This includes both individuals within the TTU system and external collaborators.

This requirement will go into effect January 1, 2023. For more information visit our webpage.

Effective January 1, 2023, TTU research personnel must complete one of the following trainings every 3 years:

- CITI - TTU Human Subject Research - Required Basic
- TTU Human Subject Training
- External – CITI Human Subject Training

4-1 **Principal Investigator**

TTU OP 74.09

*required

4-1a **Check the appropriate box.**

✓ TTU full-time or tenured faculty member

*required

4-1a(1) **College**

College of Arts and Sciences

*required

4-1a(2) **Department**

Kinesiology & Sport Management

TTU full-time employee with a terminal degree in their discipline

*required

Find and add PI to the submission.

4-1b Name: Hui Ying Luk
Organization: Kinesiology and Sport Mgmt
Address: 2500 Broadway MS3011, Lubbock, TX 79409
Phone: 8068340827
Email: huiying.luk@ttu.edu

4-1a(3)a Attach human subject training.

Luk_CITI.pdf

Primary Contact

4-2

Primary contacts need to be included in section 4.3 as a co-investigator if they are not the principal investigator. The primary contact and principal investigator will receive all notifications and letters.

*required

4-2a **Find and add primary contact to the submission.**

Name: Danielle Levitt-Budnar
Organization: Kinesiology and Sport Mgmt
Address: 2500 Broadway MS3011, Lubbock, TX 79409
Phone: 8068341830
Email: Danielle.Levitt@ttu.edu

Name: Hui Ying Luk
Organization: Kinesiology and Sport Mgmt
Address: 2500 Broadway MS3011, Lubbock, TX 79409
Phone: 8068340827
Email: huiying.luk@ttu.edu

4-3 **Co-Investigators**

Include investigators assisting with instrument development, recruitment, data collection, and data analysis.

*required

4-3a **Select all investigators involved with this research project.**

None

✓ List TTU Faculty or Staff Co-Investigator(s):

*required

Multiple names are allowed.

Name: Fangyuan Zhang
Organization: Mathematics and Statistics
Address: 2500 Broadway MS1042, Lubbock, TX 79409
Phone: 8068342587
Email: fangyuan.zhang@ttu.edu

Name: Heather Vellers
Organization: Kinesiology and Sport Mgmt
Address: 2500 Broadway MS43011, Lubbock, TX 79409-3011
Phone: 8068348554
Email: heather.l.vellers@ttu.edu

4-3a(1) Name: Danielle Levitt-Budnar
Organization: Kinesiology and Sport Mgmt
Address: 2500 Broadway MS3011, Lubbock, TX 79409
Phone: 8068341830
Email: Danielle.Levitt@ttu.edu

Name: Terrell Hilliard
Organization: Kinesiology and Sport Mgmt
Address: 2500 Broadway Ms 3011, Lubbock, TX 79409
Phone: 8068345585
Email: terhilli@ttu.edu

Name: Jaden Hendrix
Organization: Kinesiology and Sport Mgmt
Address: , Lubbock, TX 79409-1035
Phone: 8067423371
Email: jadhendr@ttu.edu

Name: Benjamin Barr
Organization: Kinesiology and Sport Mgmt
Address: 2500 Broadway MS 3131, Lubbock, TX 79409-3131
Phone: 8067423371
Email: Benjamin.Barr@ttu.edu

4-3a(1)(a) Attach human subject training.

Terrell_ Human Subject Research.pdf

Jaden_Human_Subject_JH_7.24.pdf

TTU Human Subject Research - CITI.pdf

✔ List TTU Graduate or Undergraduate Co-Investigator(s):

4-3a(2) *Multiple names and selections are allowed.*

✔ Graduate Student(s)
*required

Name: Casey Appell
Organization: Kinesiology and Sport Mgmt
Address: , Lubbock, TX 79409-1035
Phone:
Email: Casey.Appell@ttu.edu

4-3a(2)a Name: Parisa Foroozan
Organization: Kinesiology and Sport Mgmt
Address: , Lubbock, TX 79409-1035

Phone:
Email: parisa.foroozan@ttu.edu

Name: Masoumeh Rasoli
Organization: Kinesiology and Sport Mgmt
Address: , Lubbock, TX 79409-1035
Phone:
Email: mrasoli@ttu.edu

4-3a(2)a1 Attach human subject training.

---

*required

4-3a(2)b **The purpose of this study:**

Doctoral Dissertation

Master's Thesis

Research conducted as a class assignment with the intention of publishing results

Class assignment requiring IRB submission with no intention of publishing results

✓ Students are members of the research team

Other

✓ Undergraduate Student(s)

*required

4-3a(2)c

Name: Ashley Williams
Organization: Kinesiology and Sport Mgmt
Address: , Lubbock, TX 79409-1035
Phone:
Email: wil98200@ttu.edu

Name: Matthew DiLeo
Organization: Kinesiology and Sport Mgmt
Address: , Lubbock, TX 79409-1035
Phone:
Email: madileo@ttu.edu

Name: Connor Mills
Organization: Kinesiology and Sport Mgmt
Address: , Lubbock, TX 79409-1035

Phone:
Email: mil67126@ttu.edu

Name: Ernan Carrasco
Organization: Kinesiology and Sport Mgmt
Address: , Lubbock, TX 79409-1035
Phone:
Email: erncarra@ttu.edu

4-3a(2)c1 Attach human subject training.

*required

4-3a(2)d **The purpose of this study:**

Undergraduate Research

✓ Students are members of the research team

Research conducted as a class assignment with the intention of publishing results

Class assignment requiring IRB submission with no intention of publishing results

Other

✓ External Researchers: Co-investigators outside of Texas Tech University

*required

List non-TTU investigator(s) engaged in this research. Provide their contact information, title, and place of work.

Robyn Richmond, MD
Assistant Professor of Surgery
Associate Program Director, General Surgery Residency
Trauma, Surgical Critical Care, Acute Care Surgery
Department of Surgery
Texas Tech University Health Sciences Center
robyn.richmond@ttuhsc.edu
972 987 9616

4-3a(3) Leslie Shen, Ph.D.
Associate Dean for Research & Professor of Pathology

School of Medicine
Department of Pathology
Texas Tech University Health Sciences Center
leslie.shen@ttuhsc.edu

Rama Chemitiganti, MD
ECHD Endowed Chair of Medicine
Director, Center of Excellence for Diabetes and Endocrinology
School of Medicine
Department of Internal Medicine
Texas Tech University Health Sciences Center at Permian Basin
rama.chemitiganti@ttuhsc.edu
806-743-3280

*required

4-3a(3)a **Check all that apply.**

Developing instrument

Recruiting participants

Interaction with participants

Working with de-identified data

Working with identifiable data

Assisting with writing manuscript

✓ Other

*required

Include any information that helps to explain their role with the research team.

4-3a(3)a1

Dr. Richmond will provide medical-related oversight/consultation on the muscle biopsy procedures. There will be no direct interaction between Dr. Richmond and the participants.

Dr. Shen will provide consultation on participant recruitment.

Dr. Chemitiganti is the NIH-approved Safety Officer for this study. There will be no direct interaction between Dr. Chemitiganti and the participants.

Attach human subject training.

5: Funding

Complete the following section.

5-1 Funding

Funding is used to supplement the cost of the research and/or participant payment.

*required

5-1a **Select the type of funding to be used.**

✓ External (Office of Research Services - ORS)

*required

5-1a(1) **Cayuse SP - Sponsored Project Number (Example: 16-0123)**

23-0477

*required

5-1a(2) **Sponsored Project Title**

Glycemic control and frailty risk in older people at risk for type 2 diabetes: Impact of local heat therapy

*required

5-1a(3) **Find and add the Sponsor's name.**

Name

-

- Name - A to Z
- Name - Z to A

NIH - National Institute on Aging

*required

5-1a(4) **Attach a copy of the sponsored project that was submitted to the funding agency.**

For example: scope of work, abstract, research description.

FINAL_ESI R01_HYLDEL_Specific aims.docx

FINAL_ESI R01_HYLDEL_Research Strategy.docx

Internal (TTU Funding)

Personal Monies

No Funding

5-2 **Future Funding**

If funding is secured, a modification will be required.

5-2a **Enter the potential sponsor(s) name below.**

6: Rationale

Please respond to the following statements to help our IRB reviewers understand your research project.

6-1 Research Objective

*required

Briefly summarize the relevant background information.

Frailty-related medical expenses cost approximately $18 billion annually in the USA. Progressive losses in skeletal muscle (SKM) mass and function, often observed with aging and type 2 diabetes (T2D), contribute to phenotypic frailty characterized by slow gait speed, weakness, weight loss, fatigue, and low physical activity. T2D increases frailty risk by nearly 50% and risk for both conditions increases with age; therefore, preventing progression to T2D in older adults with prediabetes is critical. Additionally, SKM is the largest glucose disposal site in the body and insulin responsiveness, a component of glycemic control, is essential to maintain functional SKM mass. Thus, impaired glycemic control, a pathophysiological change underlying the development of T2D and observed in prediabetes, increases frailty risk. Conversely, improving capillarization and mitochondrial function support increased SKM mass and glycemic control, thereby decreasing T2D and frailty risk. Together, these SKM architectural variables (e.g., cross-sectional area [CSA], capillarization, mitochondria) are attractive targets for interventions such as exercise in prediabetic older people. High-intensity interval training (HIIT) has been used effectively in older adults with SKM benefits similar to those from aerobic and resistance training, and is more time-efficient. However, prediabetic older people may be exercise-intolerant or -resistant, underscoring the need for alternative therapies in place of (or in addition to) exercise. Emerging evidence supports repeated heat therapy as an alternative method to improve glycemic control and SKM architecture, and such adaptations may also improve muscle growth responses to subsequent exercise. While whole-body heat therapy is widely studied, local heat therapy (e.g., heat pad) is more practical and likely provides similar health benefits. Whether local heat therapy would have similar benefits to HIIT or whether heat pre-conditioning would improve adaptations to subsequent HIIT in prediabetic older adults is unknown.

6-2 **Importance of Research**

---

*required

Explain the scientific importance of the knowledge to be obtained as a result of this research.

Exercise is a first-line intervention for improving glycemic control and decreasing frailty risk due largely to adaptations in SKM. While exercise has a host of whole-body benefits, older individuals in general and those at risk for T2D (i.e., prediabetic) in particular may be exercise-resistant or -intolerant. Therefore, there is an urgent need for **efficacious and practical interventions** to improve muscle health in aging people to decrease their risk of developing T2D and frailty. The proposed project is innovative because we will:

- Use local heat therapy as an *easily implemented intervention* to improve the fundamental aspects of muscle health in aging prediabetic people, a novel application of this widely-used therapy
- Compare the efficacy of our proposed intervention alone and as a means of *preconditioning muscle* for improved exercise adaptations

Finally, this project will provide essential fundamental evidence to lay the groundwork for a subsequent clinical trial that examines the use of this technique in aging people with other SKM-related comorbidities. This work has the potential to **shift clinical practice** by providing direct evidence that supports a low-cost, practical intervention to improve metabolic health in at-risk individuals.

6-3 **Research Questions/Specific Aims**

---

*required

List the research question(s)/specific aims to be addressed.

The overarching hypothesis of this study is that local heat therapy improves muscle architecture, glycemic control, and subsequent exercise adaptations, and decreases frailty risk in prediabetic older adults, with TRPV1 as an underlying mechanism [mechanistic experiments will be in Aim 3, not part of this IRB application]. We will test this hypothesis through the following specific aims:

Aim 1: Test the hypothesis that local heat therapy improves muscle architecture, glucose tolerance, and frailty indicators similarly to HIIT in older prediabetic people.

Aim 2: Test the hypothesis that local heat therapy pre-conditioning improves the skeletal muscle response to HIIT in older prediabetic people.

6-5 Citations

Enter or attach the references cited .

The references below are from our Specific Aims and Research Strategy documents. Although they are not cited in the project summary, importance, and aim above, they are cited in the funding application.

ESI R01_Bibliography & References Cited.docx

Please respond to the following statements/questions to help our IRB reviewers know more about the participants in this study.

7-1 **Relationship with Participants**

*required

7-1a **Is there the potential for any relationship between the researcher(s) and the proposed target population?**

(Note: if recruiting in the Lubbock (which includes TTU) community please click yes as students and employees are possible)

✓ Yes

*required

7-1a(1) **Explain**

Recruiting older adults with prediabetes in Lubbock; it is possible that other TTU employees could be recruited.

No

Coercion or Undue Influence

Guidance for IRB and Investigators

Coercion - Coercion entails influencing an individual's decision about whether or not to

7-2 do something by using explicit or implied threats (loss of good standing in a job, poor grades, etc.). This area also includes the perception of students as to any potential backlash for failing to take part in an instructor's research.

Undue Influence - This is defined as an offer of an excessive, unwarranted, inappropriate, or improper reward or other overture in order to obtain compliance (used in association with recruiting research participants).

*required

7-2a **Does this research involve participants who could be coerced or unduly influenced to participate or may feel that there is perceived coercion or undue influence (i.e., students, employees, prisoners, colleagues, friends/acquaintances, family members, etc.)?**

✓ Yes

*required

Describe the plan to mitigate (perceived) coercion or undue influence to participants.

7-2a(1)

One example of mitigating coercion/undue influence would be to add a sentence to the consent form that clearly states their decision to participate, not participate, or withdraw in the research will not impact their employment status or academic standing.

We will emphasize to participants that their enrollment and continued participation is completely up to them. They can withdraw from the study at any time for any reason without consequences. The PIs of this study do not currently have any influence over the employment of individuals who would qualify for this study. Any future non-traditional student who might qualify will be assured that their grade(s) will noty be impacted, positively or negatively, by their participation or withdrawal. Colleagues in KSM who might qualify and enroll will be assured that our working relationships will not be impacted, positively or negatively, by their participation or withdrawal. The PIs are committed to ensuring that participation and/or withdrawal of KSM colleagues who might choose to participate and/or withdrawal does not impact our decisions for any future faculty votes, recommendations, etc. Finally, we will emphasize the importance of confiidentiality with our research team, **especially** when a participant might be a TTU (including KSM) employee.

No

7-3 **Target Population**

*required

7-3a **Describe the population of human subjects to be recruited and why this research specifically targets this group?**

In this study, participants with elevated blood glucose, prediabetes, or are at risk for developing diabetes; are sedentary will be recruited.

This population is targeted because the muscular pathophysiology of T2D (i.e., insulin resistance [IR], decreased skeletal muscle [SKM] mass and function) contributes to phenotypic frailty including slow gait speed, weakness, unintentional weight loss, fatigue, and low physical activity. Approximately 7-15% of community-dwelling older adults meet criteria for frailty, and 25-40% of these individuals are prediabetic. Moreover, a recent meta-analysis of community-based studies found that baseline T2D is associated with nearly 50% increased risk of frailty development.

Although exercise training is a key technique to manage metabolic disease and decrease T2D risk, in older adults at risk for T2D, compromised oxygen delivery and mitochondrial dysfunction result in exercise intolerance, a potential contributor to low exercise adherence. Thus, it is critical to find alternative means to manage metabolic disease risk. Heat therapy is one possible alternative. Heat therapy adaptations could improve exercise tolerance by preconditioning the microenvironment of skeletal muscle, likely enhancing exercise adherence and improving subsequent exercise adaptations.

*required

7-3b **Does your target population require specific characteristics?**

Yes, my target population **DOES** require specific characteristics.

✓ No, my target population **DOES NOT** require specific characteristics.

*required

7-3c **Age Range**

Check all that apply.

✓ Adults

*required

7-3c(1) **Enter the age range.**

≥50

Children

*required

7-3d **Enter the number of participants.**

27 men and 27 women

7-4 **Inclusion/Exclusion Criteria**

Enter or attach all criteria used to include or exclude participants for this research.

Individuals with elevated blood glucose, prediabetes, or are at risk for developing diabetes; are sedentary

Inclusion:
•Sedentary (structured exercise <30 minutes, 3x/week)
•Meet criteria for prediabetes (fasting blood glucose 100-125 mg/dl and/or hemoglobin A1c 5.7-6.4%)
•Age ≥ 50 years
• Body weight is at least 110 pounds
•Consume <8 (women) or <15 (men) alcohol-containing beverages per week
•Women who are postmenopausal, defined as no menstrual period for at least 12 consecutive

months.
•Do not use nicotine or cannabis
•Not taking any medications that could interfere with responses to the interventions (e.g., corticosteroids, opiates, benzodiazepines, tricyclic antidepressants, beta blockers, sulfonylureas, insulin, metformin, anticoagulants, barbiturates, insulin sensitizers, fibrates [PPAR gamma agonist], immunosuppressants)

Exclusion:
•History of peripheral neuropathies
7-4a •Currently taking prescription blood thinners
•Medical complications that could would contraindicate participation in the high intensity interval training (HIIT) intervention including: orthopedic complications that would limit your ability to perform cycling exercise, significant cardiovascular impairments (e.g., history of arrhythmias, severe uncontrolled hypertension, etc.), diagnosed metabolic disease (e.g., diabetes), renal disease, sickle cell anemia, or cancer in remission for <6 months.
•Known history of slow wound healing
•Excessive subcutaneous fat over the vastus lateralis (greater than 1.5")
• Have experienced symptoms suggestive of cardiovascular, respiratory, metabolic, or renal diseases including discomfort, pressure, or pain in your chest, neck, jaw, arms, calves, or other areas potentially related to ischemia; shortness of breath at rest or with mild exertion; dizziness or fainting (syncope); difficulty breathing while lying flat (orthopnea) or sudden nighttime breathing difficulties (paroxysmal nocturnal dyspnea); palpitations or rapid heartbeat (tachycardia); pain or cramping in your legs during physical activity (intermittent claudication); a known heart murmur; swelling in your ankles (edema); unusual fatigue or shortness of breath during routine activities or at rest.
• Currently pregnant
• Lidocaine allergy
• Latex allergy

We will screen for these criteria using the medical history form (attached). After inquiring with the participant, "Yes" to the following questions would result in exclusion from the study: 1, 3-7, 9-12, 15-17 (unless acute illness), 19 (lidocaine only), 20 and 23. Answers to other questions will be compared against inclusion/exclusion criteria (i.e., injuries, illnesses, chronic conditions, other reason that they should not exercise, etc.) to determine whether the participant meets all inclusion and no exclusion criteria. A key for research staff is included as a separate page in the form (beginning in Column Z on the spreadsheet).

7-4a(1) Inclusion/Exclusion Screening Form (if applicable)

M3_age_Sep 3_Informed Consent - HEAT study_Laymen term (4).docx

7-5 **End of Subject Participation**

*required

7-5a **Enter specific criteria used by the researchers to prematurely end a subject's participation in the study.**

Sometimes a PI will decide to remove a participant from a research study. List any reasons you would anticipate that may require this removal.

This is not a participant deciding they would like to stop/withdraw from the research. This is a researcher ending their participation. For example: unforeseen risk to the participants, risks to the other participants/research team members, non-adherence, disruptive behavior.

The researchers will end participants' involvement in the study early if they do not follow the study's instructions, which include:

- Adopting a new lifestyle
- Starting a new exercise routine and/or diet program
- Taking any new supplements
- New medical conditions or complications that would place participants at increased risk
- Low adherence to interventions (<80% compliance)

7-6 **Investigator Assurances**

*required

7-6a **Data Storage and Retention**

✓ I will provide participants with information about data storage and retention if they withdraw from the study, when contacted by subjects per contact information provided in the consent form.

I will **NOT** provide participants with information about data storage and retention if they withdraw from the study, when contacted by subjects per contact information provided in the consent form.

*required

7-6b **Participant Withdraw**

✓ I will provide participants with information about how to withdraw from the study through the provided consent form.

I will **NOT** provide participants with information about how to withdraw from the study through the provided consent form.

Please respond to the following statements/questions.

8-1 **Recruitment Procedures**

*required

Describe the process of how participants will be recruited and provide the sequence of these events.

8-1a

This section needs to include the details of each step for recruitment up to the point of consent. This includes pre-screening procedures.

All planned recruitment efforts and scripts will be approved by the Texas Tech University IRB. We will begin recruitment through the University Medical Center in partnership with Texas Tech University Health Sciences Center physicians who see patients from the community that may qualify. Email addresses and/or phone numbers will be obtained by searching for internal medicine, family medicine, geriatrics, and endocrinology clinics. Specially, fliers will be placed in the clinic and provided to physicians to distribute to potential participants.

Electronic advertisements will be posted on listservs (e.g., TechAnnounce, Obesity Research Institute listserv) and on social media (e.g., Facebook). Social media announcements will specify targeting older adults (50+) in the Lubbock, TX area. Additionally, after site-specific approval, flyers will be distributed around the community to frequently visited locations such as grocery stores, pharmacies, and community centers.

In addition, we will conduct door-to-door recruitment, make recruitment phone calls, and deliver recruitment speeches at various events (e.g., community center gatherings, church events, etc.). Lastly, we will disseminate recruitment emails with an attached flyer through personal and professional networks, including friends and colleagues who are willing to share the information.

Drs. Luk and Levitt will be responsible for executing recruitment efforts, collecting contact information for potential participants, conducting initial phone screenings, and following up with potential participants to schedule them for their informed consent and screening visit. Once a clinical coordinator is hired, these responsibilities will primarily shift to that

person. The initial phone screening is to ensure participant is at least 50 years old, sedentary, and their physician has informed them they have elevated blood glucose, prediabetes, or are at risk for developing diabetes.

8-2 **Recruitment Methods**

[Templates]

*required

8-2a **Select all methods of recruitment.**

Check all that apply.

No Recruitment

✓ Oral Script

*required

Enter or attach the Oral Script(s).

8-2a(2) *If more than one oral script is entered or attached, label each script separately.*

Script for UMC and TTUHSC phone recruitment is attached.

Scripts for door-to-door recruitment, phone recruitment, and recruitment events are attached.

If you attach a document, state *See Attachment* in the text box above.

8-2a(2)a

M3_age_Sep 3_Script_UMC and TTUHSC recruitment.docx

M3_age_Sep 3_Phone Script.docx

M3_age_Sep 3_event script.docx

M3_age_Sep 3_Door-to-door.docx

✓ Tech Announce

*required

8-2a(3) **Enter or attach TechAnnounce ad.**

See attached

8-2a(3)a If you attach a document, state *See Attachment* in the text box above.

M3_age_Sep 4_IM_Tech Announce Ad - HEAT study (2).docx

✓ Recruiting Letter(s) or Email(s)

*required

Explain how email addresses or physical addresses of potential participants are obtained.

8-2a(4) We will search for any email addresses for UMC or TTUHSC physicians' offices.

Also, we will disseminate recruitment emails with an attached flyer through personal and professional networks, including friends and colleagues who are willing to share the information.

*required

8-2a(5) **Enter or attach all recruiting emails or letters.**

If more than one email is entered or attached, label each separately.

Scripts attached

8-2a(5)a If you attach a document, state *See Attachment* in the text box above.

M3_age_Sep 3_Email Script_UMC and TTUHSC recruitment (1).docx

M3_age_Sep 3_Email template.docx

Information Sheet

✓ Advertisement(s)

*required

8-2a(8) **Check all that apply.**

✓ Print Advertisement (eg. newspaper, magazine, fliers)

Website

✓ Social Media

*required

Enter or attach the advertisement.

8-2a(9)

If more than one advertisement is entered, label each separately.

The attached flyer will be used for print and social media advertising.

8-2a(9)a If you attach a document, state *See Attachment* in the text box above.

M3_age_Sep 3_HEAT Flier.doc

Subject/Participant Pools

Third Party Recruitment

Other

8-2b Investigator Assurances

*required

8-2b(1) Permission to Recruit Participants

✓ I will acquire proper permission prior to recruiting participants.

I will **NOT** acquire proper permission prior to recruiting participants.

Permission to Conduct Research

8-3

*required

8-3a **Is this research study being conducted in a setting that requires prior permission from an entity to conduct research on their premises (i.e., school system, business, government entity, etc.)?**

Yes

✓ No

Visit the HRPP website for instructions and templates on the Consent Process, Assent Process with Minors, Short Form Consent Process, and Waivers of Consent.

Consent forms can be destroyed three years after the completion of the research. 45 CFR 46.115(7)(b).

9-1 **Consent**

*required

9-1a **Consent Process & Forms**

Check all that apply.

✓ Consent (Adult)

*required

9-1a(1) **Check all that apply.**

✓ Participant

Legal Authorized Representative Consent (LAR)

Parent/Legal Guardian

*required

Describe the process for obtaining consent. Include where and when the consent process takes place and who will be obtaining consent.

Drs. Luk or Levitt will provide a brief overview of the study using the information sheet and then seek informed consent from all potential participants in their offices in the KSM building to ensure privacy. All participants will be provided with copies of the information sheet and informed consent sheet. They will also be provided with a complete verbal explanation regarding the reasons for conducting the study, the procedures, risks, and benefits of the study, and any questions will be answered. At the end of the consent process, participants will be asked the following questions to establish capacity to consent:

9-1a(2)

Do you understand why this study is being done?
If you decide to participate in the study, what are some of the things you will be asked to do?
Describe some of the risks or discomforts you may experience if you participate in this study.
Will this study help you?
Do you have to be in this study?
What will happen if you decide not to be in the study?
Who should you contact if you have questions or experience a problem while in the study?

Correctly answering these questions will demonstrate an understanding of the study and determine the capacity to provide consent. Adults unable to demonstrate the capacity to consent will not be enrolled in the study.

*required

Enter or attach the consent form.

9-1a(3)

If more than one consent form is entered or attached, label each separately.

See attachment

If you attach a document, state *See Attachment* in the text box above.

9-1a(3)a

DXA Acknowledgement Form (1) (1).pdf

M3_age_Sep 3_Informed Consent - HEAT study_Laymen term (4).docx

*required

Will a Short Form Consent be used?

9-1a(4) Only used in complex studies when the PI cannot be sure that a signed written consent is understood well enough to indicate a valid consent process.

Yes

✓ No

Assent (Minors & Individuals with Impaired Decision Making Skills that cannot consent for themselves)

Texas Tech Neuroimaging Institute (TTNI) Consent Form

No consent will be obtained.

9-2 **Waivers**

A consent form must still be included in the documentation.

9-2a **Check all that apply.**

Request for a Waiver of Written Consent

Request for a Waiver or Alteration of the Elements of Consent

9-3 **FERPA Authorization**

IRB approval or determination of exemption of the research project does not constitute institutional permission to access the requested records.

Guidance: FERPA applies to personally identifiable information in educational records. This includes items such as the student's name, names of family members, addresses, personal identifiers such as social security numbers, and personal characteristics or

other information that make the student's identity easily traceable.

Educational records are all records that contain information directly related to a student and are maintained by an educational agency or institution, or by a party acting on its behalf. A record means any information recorded in any way, including handwriting, print, tape, film, microfilm, microfiche, and digital images.

9-3a **Will you be using student records protected under FERPA?**

Yes

✓ No

HIPAA Authorization or Waiver

9-4

Indicate whether you are obtaining Participant Authorization or requesting a Full or Partial Waiver of HIPAA Authorization.

9-4a **Select the appropriate authorization.**

Participant Authorization of Protected Health Information

Full or Partial Waiver of Protected Health Information

10: Data Collection Procedures & Documentation

Complete the following section.

Data Collection

10-1 IRB members find it very helpful to have copies of all instruments. Reading the instrument(s) is essential to the board's assessment of participant risk and burden and the adequacy of the study description in the informed consent. Occasionally, however, investigators do not include all or full copies of instruments on the grounds that the instrument is copyrighted and in the belief that making copies to submit with an IRB application is a violation of copyright law. However, instrument developers and publishers should have a reasonable expectation that instrument users must obtain IRB approval of their research and that approval requires review of all study materials. Further, IRB members are well aware of their own ethical responsibility to honor copyright and purchase contracts by investigators and to refrain from use of copyrighted material for purposes other than those related to their IRB obligations. If records are being withheld pursuant to a Non-Disclosure Agreement (NDA), you (the investigator) are expected to provide the relevant portion of the NDA or any similar documentation requiring the records be withheld specifically from the IRB. If the NDA or license precludes sharing with the IRB, it is the PI's responsibility to get approval from the company or licensor to share the instrument(s) with TTU's IRB for review.

*required

Describe the sequence of events and timeline the participants will experience.

A randomized controlled intervention design will be used. The study consists of 2 phases, each 12-weeks in duration.

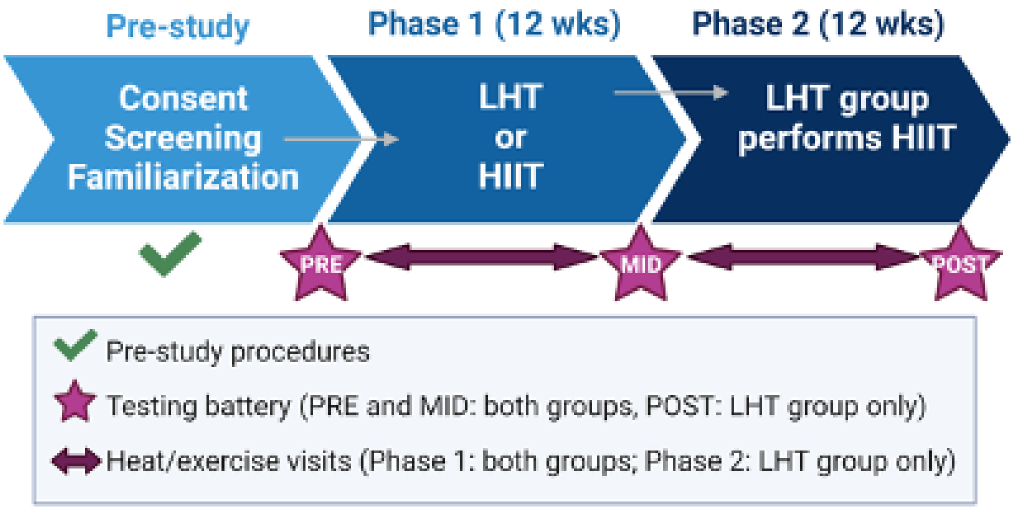

Visit 1: Consent and screening: (~2h)
•Participant will need to fast (i.e., no food or beverages other than water; plain water allowed and encouraged) for 12h before consent and screening.
•After an explanation of study requirements, participant will review and sign the informed consent form.
• Complete the medical history questionnaires
•We will collect a drop of participant blood using finger prick to determine their fasting blood glucose and HbA1c concentrations to make sure they have met the inclusion criteria.
•We will put a small drop of jelly on participant's thigh and use an ultrasound machine to image the participant's vastus lateralis muscle. This will help us make sure that the lidocaine can reach the muscle (i.e., subcutaneous fat over the muscle no greater than 1.5").
• Lastly, you will be familiarized the following set of questionnaires.
Questionnaires 1-6 will be assessed at T1, T2, and T3.
Questionnaires 2-6 will also be assessed weekly during your lab visits.
1.Unintentional weight loss (self-report, ≥10 lbs or ≥5% of body mass in the past year or between major study time points)
2.Fatigue (SF-36 energy subscale)
3.Physical activity (International Physical Activity Questionnaire)
4.24h Diet Recall log (ASA24: Healthy Eating Index 1-100 scale).
5.Sleep quality and well-being (7-point likert scale)
6. Alcohol use (TLFB: Timeline Follow Back)

PRE Testing (Before Phase 1 Begin): All major measurements for the PRE Testing will be completed over 2 separate days within a week. These major measurements will also be conducted at MID (end of Phase 1), and POST (end of Phase 2). However, the sequence of these major measurements will be different.

Preparing for Visits 2 and 3:
1. Overnight fast for 12h (only water allowed)
2. No alcohol for 24h
3. No medications or supplements from this list for 48h:
•Tylenol (acetaminophen)
•Non-steroidal anti-inflammatory drugs (e.g., Ibuprofen)

Visit 2 (Day 1 of the PRE Testing): (~ 4-5h)

1. Hydration assessment: Participant will provide a urine sample so we can assess the hydration status (urine specific gravity, a measure of hydration, must be <1.020). Also, blood sample will be collected for plasma osmolality measurement. In addition, thirst sensation will be assessed using a thirst scale and urine color will be recorded. If participant is not sufficiently hydrated, then they will be given at least 8 oz of water and re-test their hydration after 15 minutes. After 15 minutes, urine sample and thirst sensation will be measured.

2.Anthropometric Measurements:
•We will measure your height, weight, waist-hip ratio, and a body composition scan will be performed using dual X-ray absorptiometry (DXA).

3. Blood pressure:
• We will measure your blood pressure.

4.Muscle Biopsy: We will take muscle sample (~200 mg) from your outer thigh. This procedure will last about 30 minutes.

5.Oral Glucose Tolerance test (OGTT):
• Participants will consume a beverage containing 75 g glucose (TRUTOL, ThermoFisher Scientific).
•Before and at 30 min, 1 hr, and 2 hrs after beverage consumption, venous blood will be collected into vacuum tubes using the standard venipuncture technique.
6. Questionnaires: During the OGTT, you will complete the following set of questionnaires. Questionnaires 2-6 will also be filled out weekly during the study.
1.Unintentional weight loss (self-report, ≥10 lbs or ≥5% of body mass in the past year or between major study time points)
2.Fatigue (SF-36 energy subscale)
3.Physical activity (International Physical Activity Questionnaire)
4.24h Diet Recall (ASA24: Healthy Eating Index 1-100 scale).
5.Sleep quality and well-being (7-point likert scale)
6.Alcohol use (Timeline Follow Back)

Visit 3 (Day 2 of the PRE Testing) (~1.5h)

1. **Hydration assessment:** Participant will provide a urine sample so we can assess the hydration status (urine specific gravity, a measure of hydration, must be <1.020). In addition, thirst sensation will be assessed using a thirst scale and urine color will be recorded. If participant is not sufficiently hydrated, then they will be given at least 8 oz of water and re-test their hydration after 15 minutes. After 15 minutes, urine sample and thirst sensation will be measured.
2. Body weight: Body mass will be measured
3.Physical Function (part of Phenotypic Frailty Assessment) Testing:
•Perform the gait speed test (4-m walk test), 30-second Sit-to-stand test, and handgrip strength test on both hands.
4.Exercise Capacity Test:
•Perform an exercise capacity (VO2peak) test using a cycle ergometer.

Phase 1:

For the Local Heat Therapy Groups

If participants are assigned to one of the local heat therapy(LHT) groups (Sham (CON) and LHT), they will be sent home with 2 heating pads and apply local heat to their thigh 6 days for 12 weeks (5 sessions at home and 1 session at the Lab). Participants assigned to the heating group will be advised to set their heat pads to the temperature previosuly determined to achive an intramuscular temperature increase by 3-4 C; participants assigned to the sham (CON) group will be advised to set their heat pads to a lower tempereature (e.g., 36 C) to maintain a neutral intramuscualr temperature.

Visit 4: Establish heat pad temperature: (1st week of the 12-week LHT intervention)

• During the familiarization visit, we will establish the specific heat pad temperature needed to elevate each participant’s intramuscular temperature by 3-4 °C. Intramuscular temperature sensors will be used to calibrate the heat pad to approximately 3-4°C increase over a 90-minute period. For participants in the LHT group, these measurements will determine the optimal heat pad temperature. To reach the target 3-4 C increase in intramuscular temperature, the heat pad temperature will be increased by 5°C every 5 minutes. If the intramuscular temperature rises beyond 4C after resting, the heat pad temperature will be decreased to maintain the intramuscular temperature by 3-4°C above resting temerpature. After the 90-minute heating session, sensors will remain in the muscle for an additional 60 minutes to monitor temperature changes.

• To monitor skin temperature, one temperature sensor will be placed on the skin approximately 5 cm below the site of the intramuscular temperature sensor to record skin temperature. A second skin temperature sensor will be placed on the contralateral leg in a corresponding location. After securing the thermocouple, a commercially available heat pad will be positioned to surround each of the thighs. To minimize the risk of heat-related skin discomfort or burns, a size-matched flannel cloth will be inserted into the heat pad sleeve as a protective barrier. To ensure secure placement and consistent surface contact, two Velcro straps will be used to secure the heat pad firmly around the upper and lower portion of the thigh.

To measure the intramuscular temperature using intramuscular temperature probe, we will utilize the following published method (Richey et al., 2024). The PIs will receive training from consultant Dr. Steven Romero to ensure accurate technique and proper care throughout the following procedure.

1. The opposite leg from the biopsy site will be used to measure intramuscular temperature.
2. Apply lidocaine under the skin and into the muscle.
3. Put a sterile catheter or sterile 18G needle into the midpoint of the vastus lateralis, then remove the needle from the catheter.

10-1a 4. Insert the thermocouple (IT-18; Physitemp: Type T thermocouple; temperature range: -100 to +150 C/-148F to +300F) through the needle or catheter.

5. Position the thermocouple approximately 1 cm deep into the muscle.
6. Carefully retract the needle or catheter to ensure the thermocouple is correctly positioned

within the muscle.
7. Cover the area with skin-safe adhesive tape (Tegaderm Film) to ensure everything stays in place.
8. Apply heat pad on the thigh for 90 min.
9. Then remove for up to 60 additional minutes (or until the intramuscular temperature returns to baseline, whichever is first).
10. Muscle temperature and body temperature (forehead scanning) will be monitored throughout.
11. Blood pressure will be measured every 10 minutes throughout the 90-min heat pad application.
12. A bandage will be placed on the site of the incision.

o If the intramuscular temperature is above 4°C increase, we will decrease the heat pad temperature by an increment of 1°C until the intramuscular temperature stabilizes at a 4°C. If the skin temperature exceeds 41.5°C, the heat pad temperature will also be reduced by 1°C, and the pad will be loosened to increase airflow and reduce surface heating.

5. Once the intramuscular temperature has increased by 3-4°C above resting, the heat pad temperature will be set and temperature (skin and intramuscular temperatures) will be monitored for the remainder of the session.

o *Note:* We are aiming for an intramuscular temperature increased by 3-4°C. We anticipate that this will be well-tolerated because 1) core temperature remains stable during local heating on the thigh and 2) intramuscular temperatures increased by 3-4°C (vastus lateralis) have been documented in human participants without adverse effects. However, if a participant is unable to tolerate this, we will reduce the initial target intramuscular temperature as needed to a minimum of 38°C based on intramuscular adaptations previously documented in the literature.

At home Local Heat Therapy Sessions (5d/wk):
• Participants will be encouraged to empty their bladder before the start of each session to minimize the number of times they have to remove the heat pads during a session. For any reason they have to remove the heat pad, they will record it.

•For each session, participant will place the heating pads to completely surround each thigh with a snug fit. The heating pads will be secured in place using Velcro and set to the predetermined temperature continuously for 90 minutes.

• Participants will record the following for each session:
1. Start time
2. End time
3. Number of times you need to remove the heat pad during a session
4. The duration for which it is removed
5. Discomfort

•There is no restriction on the location (at home or in the office), time of day, and seated activity during the session (e.g., watching TV, reading a book, etc.).

In-Lab Local Heat Therapy Sessions (1d/wk): Visit 5-15 (~2h each) (1d/wk for the remaining 11 weeks of the 12-week LHT intervention)

•During the weekly lab visit (1d/wk), comfort will be monitored, and we will collect the logs of participants' at-home sessions to confirm compliance.

• Blood pressure will be measured every 10 minutes while the heating pad is applied.

• The following will also be assessed each week: sleep quality and activity (Hooper questionnaire and International Physical Activity Questionnaire [IPAQ]), alcohol use (Timeline Follow-back), 24-hr diet recall (ASA24), and anthropometrics (height, body mass, waist circumference, and hip circumference)

• At weeks 6 and 12 of Phase 1, we will measure both intramuscular and skin temperatures during the in-lab heat pad therapy session.

For the High_Intensity Training (HIIT) Groups

In Lab HIIT Session: Visit 4-39 (Total of 36 visits) (~1h each)

•Participants assigned to the HIIT group will arrive at the laboratory 3 d/wk for 12 weeks. They will be fitted with a heart rate monitor (Polar) and skin temperature sensors (thigh) and will perform a 3-minute cycling warm-up at 30W before beginning the HIIT protocol.

•On the first day of each training week, the following will also be assessed each week: sleep quality and activity (Hooper questionnaire and International Physical Activity Questionnaire [IPAQ]), alcohol use (Timeline Follow-back), 24-hr diet recall (ASA24), and anthropometrics (height, body mass, waist circumference, and hip circumference)

• At weeks 1, 6, and 12 of Phase 1, we will measure skin temperatures during the HIIT session.

MID Testing (End of Phase 1):

The final LHT or HIIT session must be completed 2 days before MID testing.

All major measurements for the MID Testing is identical to PRE Testing including the order of measurements. Detail protocols, please refer to PRE Testing.

Preparing for the following Visits:

1. Overnight fast for 12h (only water allowed)
2. No alcohol for 24h
3. No medications or supplements from this list for 48h:

•Tylenol (acetaminophen)

•Non-steroidal anti-inflammatory drugs (e.g., Ibuprofen)

Visit 16 (LHT)/ Visit 40 (HIIT): Day 1 MID Testing (~4-5h)

1. Hydration assessment
2. Anthropometric measurements
3. Blood Pressure
4. Muscle Biopsy
5. OGTT
6. Questionnaires

Visit 17 (LHT)/ Visit 41 (HIIT): Day 2 MID Testing (~ 1.5h)

1. Hydration assessment
2. Body weight
3. Physical functional tests
4. Exercise capacity test

Phase 2:
Visit 18 - 53 - Phase 2 (Sham (CON) and LHT groups): Participants assigned to the Sham (CON) and LHT groups will continue to Phase 2. In Phase 2, they will complete HIIT 3d/wk for 12 weeks. For detailed protocols, please refer to above (HIIT group).

POST Testing (End of Phase 2):
All major measurements and order of measurements for the POST Testing is identical to PRE and MID Testing. Detail protocols, please refer to PRE Testing.

The final LHT session must be completed 2 days before POST testing.
All major measurements for the MID Testing is identical to PRE Testing including the order of measurements. Detail protocols, please refer to PRE Testing.

Preparing for the following Visits:
1. Overnight fast for 12h (only water allowed)
2. No alcohol for 24h
3. No medications or supplements from this list for 48h:
•Tylenol (acetaminophen)
•Non-steroidal anti-inflammatory drugs (e.g., Ibuprofen)

Visit 54 (LHT): Day 1 POST Testing (~4-5h)
1. Hydration assessment
2. Anthropometric measurements
3. Blood Pressure
4. Muscle Biopsy
5. OGTT
6. Questionnaires

Visit 55 (LHT): Day 2 POST Testing (~ 1.5h)
1. Hydration assessment
2. Body weight
3. Physical functional tests
4. Exercise capacity test

*For Sham (CON) and LHT groups, participants will have a total of **55 Lab visits** with a total time spent for about **81.5h (Lab)** plus **90h (at home heat pad therapy)**.*

*For the HIIT group, participants will have a total of **41 Lab visits** with a total time spent for about **50h**.*

*required

10-1a(1) **What is the total time commitment for the participant?**

For CON (Sham, considered under the "LHT groups" in the consent form) or true LHT group, the duration will be approximately 7 months (testing: 1 month total; Phase 1: 3 months, Phase 2: 3 months) with a total of about 81.5h (lab) plus 90h (at home heat pad therapy). For HIIT group, the participation duration will last for approximately 4 months (testing: 3 wks total; Phase 1: 3 months) with a total of about 50h.

*required

10-1b **Select procedure(s) to be used.**

Check all that apply.

✓ Surveys

*required

10-1b(1) **Check all that apply.**

Online

✓ On-Site

Mailing

Other

*required

10-1b(2) **How much participant time is involved?**

The time commitment for surveys is included in the overall study visit time.

*required

Enter or attach the survey(s) questions or a list of survey items.

If multiple surveys are used, label them separately.

Hooper well-being survey questions (completed weekly; scored on 1-7 scale, low (fatigue, stress, soreness) or good (sleep quality) and 7 indicates high (fa soreness) or bad (sleep quality):

1. Please rate your fatigue level over the past 24 hours.
2. Please rate your level of stress over the past 24 hours.

3. Please rate your level of soreness over the past 24 hours.
4. Please rate the quality of sleep you had last night.

10-1b(3) Automated Self-Administered 24-hour Dietary Assessment Tool (ASA-24; cor
Registration for use of the ASA-24 will be completed upon Notice of Award ar
monies. A link to a demonstration website for the ASA-24 is here: https://asa2
(2024 version)

Unintentional weight loss (at PRE, MID, and POST testing): In the past year [I
12 weeks [MID and POST], have you unintentionally lost body weight? If so, h

Other surveys: see attachments.
1) Medical history (screening visit)
2) International Physical Activity Questionnaire (IPAQ)-SF (weekly)
3) Timeline Follow Back (TLFB, weekly)
4) 36-item Short Form Health Survey (SF-36) energy subscale (weekly; only i
and 31;
scored per https://www.rand.org/health-care/surveys_tools/mos/36-item-short

If you attach a document, state *See Attachment* in the text box above.

10-1b(3)a

[IPAQ-SF.pdf]()

[TLFB_Alcoholinstructions.pdf]()

[TLFB_ALoverview.docx]()

[SF-36.pdf]()

[Thirst Scale.docx]()

[urine color chart.jpg]()

[R1_11.19_IM_Detail the intervention procedures.docx]()

*required

Describe the process for the distribution and return of surveys.

10-1b(4) Surveys questions will be uploaded into Research Electronic Data Capture (REDCap), a free, secure, web-based application designed to support data capture. Researchers will assist particiants to electronically complete surveys during their laboratory visits.

Interviews

Focus Groups

Observation

Non-Invasive Procedures (MRI, X-Rays, etc.)
Invasive Medical Procedures (Blood draws, skin pricks, biopsies, etc.)

✓ Intervention

*required

10-1b(25) **How much participant time is involved?**

LHT groups ~81.5h (lab) plus 90h (at home heat therapy) and HIIT group ~50h in the laboratory

*required

10-1b(26) **Where will the intervention(s) take place?**

At home (Sham (CON) and LHT groups) and KSM building (all groups)

*required

Describe in detail the intervention procedures.

10-1b(27)

Attach any documentation if applicable.

See attachment. The LHT group described in the Informed Consent document includes LHT (heat) and CON (control; sham - low-temperature heat pads).

10-1b(27)a If you attach a document, state *See Attachment* in the text box above.

*required

Who will be conducting the intervention(s)? What are their qualifications?

Attach any documentation if applicable.

For Oral Glucose Tolerance Testing (OGTT): Drs. Luk and Levitt are experienced in phlebotomy; Dr. Levitt has carried out the OGTT procedure. Additional study team members are trained in phlebotomy (Casey Appell) and will help carry out this procedure under the supervision of Drs. Luk and Levitt. Jaden Hendrix (research aide) will be primarily responsible for processing biospecimens. Drs. Barr and Hilliard, and the listed graduate and undergraduate students will assist in these procedures as needed.

10-1b(28)

For body composition measurement, Drs. Luk and Levitt are certified DEXA technicians in the State of Texas and are approved to operate the DEXA housed in the Department of Kinesiology & Sport Management (KSM) at Texas Tech University (TTU). Their students trained on use of the DEXA will carry out this procedure under their supervision. Drs. Barr and Hilliard, and the listed graduate and undergraduate students will assist in this procedure as needed.

All biopsy procedures will be performed by Dr. Hui-Ying Luk, who is not a licensed physician but is trained specifically in the muscle biopsy procedure. Robyn Richmond, M.D. (robyn.richmond@ttuhsc.edu) will oversee the procedure (i.e., approve safety protocols, provide technical and medical advice regarding the procedures and potential adverse effects of biopsy). Dr. Richmond will determine the competency of the person who will perform the preparation (sterilized procedures, lidocaine injection) and the operation (maintaining sterile procedure for the primary operator: Dr. Luk). Since the assistant (applying suction through a syringe) does not have direct contact with the participant and does not require sterile procedures, the competency of the assistant will be determined by Dr. Luk. Our members are not going to diagnose these symptoms, instead, if the participant is self-reporting pain, erythema (warmth), or ecchymosis (bruising) to us, we will advise them to use a cold pack. Also, our members will follow up with them regarding the self-reported signs. Similarly, if the participant is seeing and self-reporting signs of infection, inflammation, hematoma (e.g., prolonged redness, pus formation, increasing pain, bruising at the site of biopsy, and fever, etc.), and numbness at the site of biopsy, we will refer them to consult with a medical provider. Lastly, an individual with CPR training (current) will be on-site within close proximity to the subject during procedures.

All intramuscular temperature measurement will be performed by Drs. Hui Ying Luk and Danielle Levitt who are trained specifically in this procedure by Dr. Steven Romero. The proposed technique has been safely and successfully implemented in participants (e.g., PMID: 38059292). Drs. Barr and Hilliard and Casey Appell will assist in this procedure as needed.

For exercise testing and intervention, Drs. Luk and Levitt have experience in exercise testing and intervention and are Certified Strength and Conditioning Specialists (CSCS) through the National Strength and Conditioning Association (NSCA). The proposed exercise capacity testing and HIIT intervention have been safely and successfully implemented in older adult participants (e.g. PMID: 29077855, 28273480). Dr. Luk and Levitt will train and supervise all personnel (graduate and undergraduate students on this IRB application) in conducting the exercise testing and intervention included herein. Only study personnel with currect CPR and first aid training will perform exercise testing and intervention. Dr. Levitt has an up-to-date CPR and first aid certification, and has held valid CPR

certification continuously since 2003. Drs. Barr and Hilliard, and the listed graduate and undergraduate students will assist in these procedures as needed.

For frailty risk assessment, Dr. Levitt has assessed frailty risk in people living with HIV. She will train and supervise all personnel (graduate and undergraduate students on this IRB application) in conducting the physical function testing to assess frailty risk. Drs. Barr and Hilliard, and the listed graduate and undergraduate students will assist in these procedures as needed.

If you attach a document, state *See Attachment* in the text box above.

10-1b(28)a

incidents updated (1) (2) (2) (2) (1).docx

IM temp and heat therapy in older adults.docx

Other

Deception

10-2

The result of investigators providing false or incomplete information to participants for the purpose of misleading research subjects.

*required

10-2a **Does this research project involve the use of deception?**

✓ Yes

*required

10-2a(1) **Explain why deception is necessary.**

A sham group is needed for scientific purposes.

No

10-3 **Debriefing**

10-3a **Will a debriefing script be used at the conclusion of the research?**

✓ Yes

*required

10-3a(1) **Select the reason for needing a debriefing script.**

✓ Deception

Sexual Misconduct

Mental Health, Self-harm, Substance Abuse, Criminal Behavior

✓ Education Tool

*required

10-3a(2) **Enter or attach a copy of the debriefing script.**

See attachment

If you attach a document, state *See Attachment* in the text box above.

10-3a(2)a Biopsy care kit.doc

Deception Script.docx

IM care kit.doc

*required

Provide information on who will conduct the debriefing and how it will be delivered to the participant.

Dr. Luk, Dr. Levitt, or clinical coordinator (future) will conduct the debriefing.

Upon the completion of the biopsy procedures, we will provide a take-home kit (i.e., notes and necessary consumables, e.g., extra bandage, alcohol

10-3a(3) wipes). Also, we will verbally explain the biopsy care instructions and a list of drugs that can increase bleeding (see the attachment), especially focusing on the medication that should be avoided. Furthermore, our research team will follow up with the participant in the following two days by text/call to make sure no adverse effect was observed (swelling and warmth at the site of biopsy). You can also send us a photo of the biopsy site through email or text message if you have any concerns. We will look at your biopsy site on your next visit.

For the control group (CON), we will disclose that the temperature setting for the heat pad used during the study was for control purposes, and we will specify the temperature they need to set to achieve an intramuscular temperature of 40 degrees Celsius.

No

10-4 TTU Compliance Committees

Additional approvals

*required

10-4a **Does this research require internal approval from another TTU compliance committee or entity?**

✓ Yes

10-4a(1) **Select the TTU Committee(s) that grants approval to conduct research at that location and attach the approval letter.**

Texas Tech Neuroimaging Institute (TTNI)

✓ TTU Institutional Biosafety Committee (IBC)

*required

Attach the approval letter.

Luk_IBC.pdf

IBC-2023-1083 approved v9.pdf

2023-2025_KSM_DXA_Sublicense.pdf

10-4a(1)b

Luk_DEXA training.pdf

Luk_Handling Radioactive Material.pdf

Luk_Introduction to Radiation Safety.pdf

Luk_Radiation Safety.pdf

Luk_X-Ray Safety.pdf

✓ TTU Institutional Laboratory Safety Committee (ILSC)
*required

Attach the approval letter.

10-4a(1)c

Luk_IBC.pdf

IBC-2023-1083 approved v9.pdf

2023-2025_KSM_DXA_Sublicense.pdf

Luk_DEXA training.pdf

Luk_Handling Radioactive Material.pdf

Luk_Introduction to Radiation Safety.pdf

Luk_Radiation Safety.pdf

Luk_X-Ray Safety.pdf

Institutional Animal Care and Use Committee (IACUC)

✓ TTU Radiation and Laser Safety Committee (RLSC)
*required

Attach the approval letter.

10-4a(1)e

Luk_IBC.pdf

IBC-2023-1083 approved v9.pdf

2023-2025_KSM_DXA_Sublicense.pdf

Luk_DEXA training.pdf

Luk_Handling Radioactive Material.pdf

Luk_Introduction to Radiation Safety.pdf

Luk_Radiation Safety.pdf

Luk_X-Ray Safety.pdf

Other

No

Complete the following section.

11-1 Personal Identifiers

*required

11-1a Select personal identifiers that will be collected for the purpose of this research.

Check any identifier you will be in possession of during the research process.

- ✓ Names
- ✓ Telephone numbers
- ✓ Any elements of dates (other than year) for dates directly related to an individual, birth date, admission date, discharge date, date of death. For ages over 89 all elements of dates (including year) indicative of such age, expect that ages and elements may be aggregated into a single category of age 90 and older
- Any geographic subdivisions smaller than a state, including street address, city, county, precinct, zip code and their equivalent geocodes except for the initial three digits of a zip code
- Fax numbers
- ✓ E-mail addresses
- Social security number
- Medical record number
- Student ID username/number
- Health plan beneficiary numbers
- Account numbers

Certificate/license numbers

Vehicle identifiers and serial numbers (VIN), including license tag identification

Device identifiers and serial numbers (e.g., implanted medical device)

Web universal resource locators (URLs)

Internet protocol (IP) address numbers

Biometric identifiers, including finger and voice prints

Full face photographic images and any comparable images (i.e., videos, etc.)

✔ Biological specimens

Any other unique identifying number, code, or characteristic, other than dummy identifiers that are not derived from actual identifiers and for which the re-identification key is maintained by the health care provider and not disclosed to the researcher

No personal identifiers will be collected

11-2 **Privacy of Participants**

---

*required

11-2a **Describe how you will protect the privacy of the participants.**

Consider how you will protect the participant's privacy during:

- Recruitment
- Consent
- Data Collection
- Storage of Data
- Analysis of Data
- Findings

Data Safety:
To protect against breach of confidentiality, any hard copy materials (e.g., informed consent form, any hard copies of exercise records with heart rate and rating of perceived exertion

data, etc.) will be kept in a locked drawer in Dr. Luk's private office. The informed consent form and demographics files will be the only places participants' names appear, and only the PIs and essential study personnel will have access. Participant samples, data, and records will be deidentified and labeled using a unique ID number. Data, images, and files generated from the project will always be stored in raw form, separate from files with calculations and analyses. Lastly, all electronic material, including data from questionnaires administered using computer-assisted interview techniques (i.e., no hard copy) will be stored in a secure database (REDCap).

11-3 **De-Identifying Data**

[Anonymous v. Identifiable Data]

*required

Explain how you will de-identify the data collected. If data will be given a code, explain this process.

For example:
Personal identifiers will be removed from the data and replaced with a participant code. The participant code will be used on the individual's data. The master list containing the personal identifier(s) and participant code will be in a separate document stored away from the data. The master list and data will be password protected. The passwords will be different for the master list and data.

For each identifier explain when and how you will code/de-identify/destroy the identifier.

11-3a Consider the following:

- What identifiers are associated with data?
- What identifiers are NOT associated with the data?
- What descriptors can be used to identify someone? For example: schools, towns, buildings, colleagues, companies, titles, etc.

Upon participant's admission to the study, a unique study identification code (ID codes) will be assigned to the participant in the order they are enrolled. The file containing participants' names and IDs will be stored electronically in REDCap.

Thereafter, all data will be kept in coded participant files. All trained investigators, professional staff, and technicians are aware of the confidentiality involved with this study.

Identifiable data will not be available or divulged to anyone outside of the experimental research team and data files will be kept for at least 3 years after the study is terminated. The confidentiality of individual information will be maintained in any publications or presentations regarding this study.

11-4 **Data Transcription**

*required

11-4a **Select who will transcribe audio or voice recording.**

Check all that apply.

Member of the research team/lab

Professional transcription company

Other

✓ Not applicable

11-5 **Transfer of Data**

*required

11-5a **Will data be transferred from one location to another?**

Transfer of data can be done electronically (email, removable storage device, shared drives, remote access, hyperlinks, etc.) and/or physically (carrying, mailing, etc).

✓ Yes

*required

Explain how it will be securely transported and by whom.

1. Data type (human subjects data only)

A. **Types and amount of scientific data expected to be generated in the project:**
Data from human subjects: Demographic and anthropometric (e.g., age, sex, race, ethnicity, body mass, body composition, height), clinical (e.g., medical history, oral glucose tolerance test parameters, and questionnaires about alcohol use, diet, activity, sleep, changes in body mass, and fatigue), functional (e.g., exercise capacity, grip and leg strength, gait speed), and molecular (e.g., type I and II muscle fiber cross-sectional area, capillarization, mitochondrial enzyme activity, mitochondrial and TRPV1 protein expression, mitochondrial DNA quantification, and mitochondrial content and morphology) data will be collected from 54 older adults at risk for type 2 diabetes (described in detail in the “Human Subjects” attachment) at several time points throughout the study. Weekly assessments will include body mass measurement, diet and alcohol questionnaires, and activity and sleep monitoring via and self-report questionnaires. All other data will be collected only at the beginning of the study (i.e., demographics, medical history) or at the 2-3 major time points. Participants assigned to the high-intensity interval training (HIIT) group (n=18) for phase 1 will have 2 major time points (before [T1] and after [T2] phase 1). Participants assigned to the LHT (n=18) and CON (n=18) groups for phase 1 and HIIT for phase 2 will have 3 major time points (before phase 1 [T1], between phases 1 and 2 [T2], and after phase 2 [T3]).

Large-scale data sets: It is possible that large scale mitochondrial genomic data (human participants) and proteomic data (cell culture) could be generated from this project if alternative approaches must be used as described in the research strategy.

The total amount of anticipated data if alternative approaches are not needed is ~25 GB of data.

B. **Scientific data that will be preserved, shared, and the rationale for doing so:** All raw data generated from this project will be stored in a password-protected manner on cloud-based servers at Texas Tech University. Cleaned data in an analysis-ready format will be stored in REDCap and periodically uploaded to repositories. Data of sufficient quality will be uploaded to allow others to validate and replicate research findings described in the Aims.

C. **Metadata, other relevant data, and associated documentation**: In alignment with the Data Submission Worksheet for the Aging Research Biobank, the following will be submitted to accompany data derived from human subjects:

•Full study protocol
•Names and descriptions of data sets
•Manual of operations

•Annotated data collection forms
•Data dictionary
•Documentation of calculated variables
•Summary of deidentification and crosslinking of study ID with new randomized ID
•Any changes made to the protocol over time
•Frozen datasets used for the primary publication, when applicable
Similarly, the full study protocol, dataset names/descriptions, data dictionary, documentation of calculated variables, and any changes to the protocol over time will be deposited in TTU Dataverse for molecular procedures.

2. **Related tools, software, and/or code:** Statistical analyses will be run using standard statistical software (SPSS version 29) and graphs made using GraphPad Prism (Version 9). Image processing will be performed using ImageJ and in part using custom Python code to automate quantification where appropriate.

3. **Standards:** No consensus standards of data formatting such as Dublin or Darwin core exist for our discipline. Data dictionaries defining each variable and decoding any coded responses will accompany all uploaded data.

4. **Data preservation, access, and associated timelines:**
A. Repository where scientific data and metadata will be archived:
Human subjects data: NIA's Aging Research Biobank
Molecular data: TTU Dataverse Collection
Mitochondrial genomic data: Database of Genotypes and Phenotypes
11-5a(1) (dbGaP; if performed)
Custom code: GitHub

B. How scientific data will be findable and identifiable: Data will be findable for the research community through the repositories listed above. Collections will be established in the Aging Research Biobank and in Dataverse when this application is funded. Collections will be established on dbGaP should a large data set be generated. Data stored in the Aging Research Biobank will be made available by reasonable request, and this will be noted in the "Data Availability Statement" in associated publications. When data are made public via Dataverse, a digital object identifier (DOI) will be assigned. This DOI be referenced in associated publications to allow the research community access to the data used in each publication. If dbGaP is used, researchers can submit requests to dbGaP to access and browse data, and this information will be included in any associated publications. Any custom code will be shared on GitHub, accessible by searching "rbudnar" on GitHub. The readme.md file for the project will include instructions for the analyses and rationale for parameter choices.

C. When and how long the scientific data will be made available: Data associated with each publication will be made available when preprints are available and will continue to be available on the repositories and long as

they are supported. Any data that are not yet available by the end of the award period will be made available at that time.

5. Access, distribution, or reuse considerations:
A. Factors affecting subsequent access, distribution, or reuse of scientific data: Broad data sharing will be included in the informed consent form. Data deidentification, linking data using unique identifiers, protection of privacy, and making data available will be explained to participants and any questions answered as part of the informed consent process.

B. Whether access to scientific data will be controlled: Access to human subjects data will be controlled. Requests will be submitted through the appropriate repository (Aging Research Biobank, Dataverse, dbGaP).

C. Protections for privacy, rights, and confidentiality of human research participants: The only location that personal information will be paired with participant ID will be on a single administrative data collection form in the secure REDCap database. This is necessary because data will be collected from the same participants and multiple time points. However, each participant will be assigned an ID code and only the ID code will be linked to data so that the data are not identifiable. Data from human participants will be further deidentified upon submission to the repository(ies) and linked only through the study ID. The Aging Research Biobank further anonymizes data by randomly generating a new ID for each subject. dbGaP interfaces with the Aging Research Biobank so clinical and mitochondrial genomic data (if generated) can be linked, using only the randomly generated ID. Further, access to these datasets will be controlled and require that users register and submit reasonable requests. The PIs attest that no attempt will be made to reidentify participants from deidentified data.

6. Oversight of DMS: Drs. Hui-Ying Luk and Danielle Levitt (PIs) will deposit clinical and associated data into the NIA Aging Research Biobank, TTU Dataverse, and into dbGaP if mitochondrial genomic data are generated. Data upload will occur when publications associated with each data set are submitted and made public (where applicable) upon acceptance. Data sharing will be included in the annual progress report.

7. Representatives of the following people/groups within *Texas Tech University* may use your study records and share them with other specific groups in connection with this research study.
• The principal investigators (*Hui Ying Luk, Danielle Levitt*)
• The *Texas Tech University* Institutional Review Board
• The *Texas Tech University* Human Subjects Protection Office
The above people/groups may share your unidentifable study records with the following people/groups outside *Texas Tech University* for their use in connection with this research study. These groups, while monitoring the research study, may also review and/or copy your original *Texas Tech University* records.

• The Office of Human Research Protections in the U. S. Department of Health and Human Services.
• The Safety Officer appointed for this study.
• The National Institutes of Health and its authorized representatives.

No

11-6 **Certificate of Confidentiality**

*required

11-6a **Has a Certificate of Confidentiality been requested?**

Yes

✓ No

11-7 **Investigator Assurances**

*required

11-7a **Data Security**

✓ I will protect the data through at least one of these security measures: encryption software, password protection, and /or physical barrier (locked doors, file cabinets, etc.).

I will **NOT** protect the data through at least one of these security measures: encryption software, password protection, and /or physical barrier (locked doors, file cabinets, etc.).

*required

11-7b **Access to Data**

✓ I will only allow the research team access to the identifiable data.

I will allow others outside of the research team to have access to identifiable data.

*required

11-7c **Destruction of Personal Identifiers**

✓ I will destroy personal identifiers prior to closing the IRB with the exception of the consent forms.

I will **NOT** destroy personal identifiers prior to closing the IRB with the exception of the consent forms.

*required

11-7d **Retention Policy**

✓ I will keep de-identified data and/or consent forms for at least 3 years following the closure of the IRB.

I will **NOT** keep de-identified data and/or consent forms for at least 3 years following the closure of the IRB.

*required

11-7e **Data Repository**

✓ I will post the de-identified data to a data repository.

I will **NOT** post the de-identified data to a data repository.

12: Benefits & Risks

Please respond to the following statements/questions to help our IRB reviewers recognize the benefits and risks associated with this study.

12-1 **Benefits**

*required

Describe the potential direct benefit(s) to subjects.

12-1a

Payments to subjects, course credit or contribution to science should not be included in this section.

All efforts will be made throughout the study to minimize any study-related risks to subjects. The Physical Activity Guidelines for Americans, 2nd Edition (2018), suggest that adults should engage in at least 150 minutes per week of moderate-intensity aerobic exercise (or an equivalent 75 minutes per week of vigorous-intensity aerobic exercise, including HIIT) to maximize the many health benefits of aerobic exercise. Even if people do not meet the minimum of 150 minutes, smaller amounts of exercise are still beneficial. Participating in this study will provide participants with a structured environment to help meet that exercise goal during one of the two phases. Among anticipated benefits from exercise, we emphasize that there might be metabolic health benefits and decreased frailty risk, particularly because participants will be prediabetic older adults, indicating risk for the development of type 2 diabetes and frailty.

Furthermore, benefits of heat therapy are becoming increasingly recognized and we anticipate that participants assigned to the local heat therapy group will have improved skeletal muscle health, glycemic control, and decreased frailty risk. Because of the benefits anticipated from local heat therapy, all participants will be provided with heating pads regardless of group. The HIIT group will be provided with heating pads and instructions for use at the end of their participation (T2). The control group will be provided with heating pads but instructed to use a lower heat setting T1. At the end of their study participation (T3), they will be provided with instructions for use. Regardless of group, participants will have regular interaction with members of the study team. Together with exercise, prosocial interaction may have psychological benefits.

Participants will be provided with the following non-diagnostic results from testing performed at each major time point (PRE, MID, and POST):

- Dual-energy X-ray absorptiometry (DEXA) reports: Body composition and bone mineral density compared to age- and sex-specific norms.
- Body mass index (BMI): Value calculated from body mass and height (kg/m^2) and BMI category (i.e., underweight, normal weight, overweight, obese).
- Gait speed: 4-m walk time compared to age- and sex- specific norms.
- Muscle strength: Average grip strength (3 trials per hand) and 30-second sit-to-stand test with comparison to age- and sex- specific norms.
- Exercise capacity: VO_{2peak} and comparison to age- and sex-specific norms.
- Blood sugar control: If fasting blood sugar, HbA1c, or 2-hr glucose values after the glucose tolerance test fall into the diabetic range at any time point, the participant will receive a letter advising you of the laboratory measurement and to follow up with your physician.

12-2 Risks

*required

Describe any potential risk to subjects and how you will minimize that risk.

While exercise and heat have many potential benefits, they always carry possible risks. These risks are minimal, and the benefits of exercise and heat therapy largely outweigh the risks of a sedentary lifestyle. Those with higher-than-average risk for complications of exercise (e.g., preexisting cardiovascular conditions) will be excluded from the study. Risks of sample collection are also minimal, and the precautions taken will further offset these risks. The qualifications of the PIs and the protections in place for human subjects will ensure a safe environment for study participation.

Protections against risk
Strategies for minimizing risks associated with medical history and questionnaires, collection of biological specimens, and exercise are described below. All procedures will be fully approved by the TTU IRB. Drs. Luk and Levitt are trained in phlebotomy and muscle biopsies techniques and have previously conducted these procedures in IRB-approved studies. Dr. Luk is currently approved and actively performing muscle biopsies at TTU, and Dr. Levitt is in the process of obtaining such approval. All study personnel will be required to maintain valid CPR and First Aid certifications, and emergency contact information will be kept on hand. All personal information and data will be kept in a secure database (REDCap).

Protection against and minimization of risks by procedure:
•Interviews and completing questionnaires: To minimize risks of emotional stress and fatigue, the length and number of questionnaires will be kept to a minimum, and the study team will establish rapport with participants to minimize emotional stress. Contact information for

guidance counselors will be available. Protection against breach of confidentiality will be ensured by keeping all physical study materials in locked, secure locations and all electronic material in a secure database (REDCap). Except for the demographics form, all data will only be linked with unique identifiers and not with any personally identifiable information. Data uploaded to repositories will have different randomly generated unique identifiers generated during upload to the NIA Aging Research Biobank that are different from those used in REDCap or on study forms.

•Blood collection: Participants will be informed of the risks of venipuncture and finger prick. Risk for infection is low with the use of sterile technique, and a sterile adhesive bandage will be applied to the blood collection sites. Blood collection will be performed by trained personnel using standard laboratory techniques: disinfecting the site with alcohol, using sterile disposable supplies, and applying pressure with sterile gauze to minimize bleeding and bruising. Syncope is possible, so phlebotomy procedures will be performed using phlebotomy chairs in case legs need to be elevated. Cold packs will be available. Participants will be asked if they have a history of fainting prior to blood collection.

•Muscle biopsy: The following are risks that are related to the muscle biopsy procedure and are only associated with/around the incision site: Pain, bleeding, excessive redness, bruising, tenderness, swelling, warmness of the skin, localized numbness, and pink-tinted drainage from the incision. In extreme cases, a low-grade fever may occur, which would indicate that the site may be infected. None of these complications are usually life-threatening, and most complications can be managed conservatively without hospitalization.

To minimize risks, muscle biopsies will be performed using standard sterile technique. The skin over the vastus lateralis will be cleaned using povidone-iodine and the area anesthetized using 1% lidocaine without epinephrine. A small incision will be made through the skin and fascia using a single-use sterile scalpel (#11). A sterilized Bergstrom biopsy needle will be inserted into the muscle and approximately 200 mg of muscle will be collected. After the sample has been collected, pressure will be applied to the area for 5 min. The skin will be closed with a sterile adhesive bandage, covered using sterile gauze, and compression wrapped. Each participant will be given verbal and written instructions for biopsy care and extra supplies (e.g., sterile adhesive bandages, alcohol pads) will be provided. The study team will follow up with each participant 2 and 5 days following their biopsies. Sterile supplies (gloves, gauze, scalpel, needles, etc.) will be used and the PIs will train personnel to assist in the procedure. In case a participant becomes lightheaded or feels faint, we will provide food or beverages containing simple sugar (e.g., juice).

• Intramuscular temperature measurement: There may be some discomfort during the insertion of the probe into your muscle. The topical anesthetic will numb the insertion area to minimize this discomfort, but you may feel pressure or a dull ache in the muscle as the needle moves through the muscle. At the end of the session, the probe will be withdrawn and a sterile dressing will be applied. Any swelling or redness after the study should be gone a few hours after completion of the study, but you may feel some muscle soreness for several days. Although the small probes are sterile, there is a slight risk of infection at the sites where the probes were placed. You will be instructed on how to keep the area clean
12-2a for a day or two following the study and will need to inform the researchers immediately

if you have any redness or swelling in the area. To minimize risks, this measurement will be performed using standard sterile technique. The skin over the incision site will be cleaned using povidone-iodine and the area anesthetized using topical anesthetic cream. At the end of the session, the probe will be withdrawn and the skin will be closed with a sterile adhesive bandage, covered using sterile gauze, and compression wrapped. Each participant will be given verbal and written instructions for care and extra supplies (e.g., sterile adhesive bandages, alcohol pads) will be provided. Sites will also be re-assessed at the next study visit.

•Anthropometric measurements: To minimize risks associated with fatigue, these procedures will be performed near a wall or other support and chairs kept nearby in case participants need to rest between measurements. Contact information for guidance counselors will be available.

•Dual-energy x-ray absorptiometry (DEXA): DEXA is considered a low-risk procedure. However, the use of x-rays, even in low amounts, does present some risk associated with ionizing radiation. To minimize these risks, the number of DEXA scans will be kept to a minimum.

•Functional tests, exercise capacity (VO2peak) testing, and HIIT: During screening, we will ensure that participants meet ACSM medical criteria for initiating an exercise program and do not have medical complications that would put them at undue risk (e.g., diabetes, renal disease, uncontrolled hypertension, other cardiovascular disease, etc.). Participants may become faint, nauseous, experience muscle strains or soreness, or fall. To minimize risk associated with physical activity, participants will be counseled on the importance of proper hydration and will regularly be asked about their physical condition and adjustments made as necessary. Chairs will be available for rest during functional testing, and a researcher will remain close by the participant in case they lose their balance. During VO2peak testing and HIIT, we will utilize cycle-based exercise instead of treadmill exercise to prevent the risk of falls while walking, and participants will be helped on and off the cycle ergometer or exercise bicycle as needed. Participants will perform proper warm-ups and cool downs. We will also monitor heart rate and rating of perceived exertion during exercise. The VO2peak test will be performed in 3-minute stages with predetermined power output (i.e., males start at 50W, females start at 25W, and power increases by 20W every 3 minutes). If the participants cannot maintain the power output for a given stage, their cycling cadence falls below 50 rpm for two consecutive minutes, or they ask to stop the test, the test will end. In case a participant becomes lightheaded or feels faint, that day's exercise will be stopped and we will provide food or beverages containing simple sugar (e.g., juice).

•Local heat therapy: The potential risks of using local heat therapy include discomfort, skin redness, and, in rare cases, burns, particularly if used improperly or for extended periods. Core temperature increases are not typically observed with local heating on the thigh and thus is not considered a potential risk for the proposed study. However, we will monitor body temperature using a forehead scanner. If the body temperature reaches 40°C, we will stop the heat pad therapy. The risk of discomfort will be minimized by determining the lowest optimal heat pad temperature prior to the onset of the heat therapy intervention and that temperature will not be exceeded. Further, participants will be provided with contact information for the PIs should any problems occur during their at-home local heat therapy

sessions and will be asked about any discomfort during their weekly check-ins. Sometimes, redness may appear on your skin in a pattern after the heat therapy session, but it should fade within a day. Heat therapy sessions will occur in the laboratory once weekly and will be monitored by study personnel to ensure that no problems occur during heating sessions. The risk of burns due to personal heating pad use in older adults is not well-documented. However, in a 2021 study (PMID: 33031521), a review of burn injuries in older adults (age 65+) registered in the National Electronic Injury Surveillance System was performed. For adults aged 65-84 years, heating pads were not in the top 5 causes of burn injuries. In the 85+ year-old age group, heating pads were in the top 5 causes of burn injuries. An estimated 204 burns across 100 emergency departments in the United States over a 10-year period were attributable to electric heating pad use over a ten-year period, and all were in women. The risk of burns due to heating pads in this age group is increased by chronic comorbidities that are exclusionary for this study (i.e., diabetes, peripheral vascular disease, chronic kidney disease, neuropathy), minimizing risk. The risk also increases if heating pads are used while sleeping, so we will instruct participants to set a timer and/or alarm to ensure they do not exceed the 90-minute application time, also minimizing risk. Finally, it is estimated that a surface temperature between 42-43°C would produce a burn injury in 12-20 hours (PMID:1885636). Because the difference between skin and muscle temperature is approximately 2°C across a variety of studies, we anticipate that the heating pad setting to produce an intramuscular temperature of 40°C will be approximately 42°C. The 90-minute application time is far below the conservative end of the estimated contact time frame to produce a burn injury (12 hours).

12-2a(1) **Is a liability plan offered?**

Yes

✓ No

13: Participant Compensation

13-1 Compensation - Payments to Research Participants

Compensation through TTU funds must comply with TTU OP 62.25.

*required

13-1a **Are subjects being compensated for their participation in this study?**

✓ Yes

*required

13-1a(1) **Describe the compensation process.**

Participant will be paid via cash upon completion of each visit:

Consent/screening ($10)

Major Measurement Testing (PRE, MID, POST):
•Muscle biopsy ($50) and OGTT/Questionnaires ($50)
•Physical function and exercise capacity tests ($50)
The major time point testing will be conducted twice for the HIIT group and three times for Local Heat groups.

For each laboratory visit, participants will be compensated $15.
Phase 1:
•LHT: 12 visits = $180
•HIIT: 36 visits = $540

Phase 2:
•LHT: 36 visits = $540

Total:
•LHT: $1180
•HIIT: $850

13-1a(2) **Select the payment type used.**

✓ Cash

*required

Amount of Payment

13-1a(2)a

Amount will be paid according to the completion of each visit, please see above for details. Participants who are assigned to the LHT groups and complete each visit in its entirety will receive a total of $1180. Participants who are assigned to the HIIT groups and complete each visit in its entirety will receive a total of $850.

*required

13-1a(2)b

When and how will the participant be paid?

payments in cash will be disbursed after each visit

Check

Gift Card (not to be checked if a drawing is involved)

Drawing

Course Credit

Other (includes payment or credit from 3rd party affiliations (i.e., MTurk, SSI, etc.)

Investigator Assurances

Guidance: The Institutional Review Board (IRB) should determine that the risks to subjects are reasonable in relation to anticipated benefits [21 CFR 56.111(a)(2)] and that the consent document contains an adequate description of the study procedures [21 CFR 50.25(a)(1)] as well as the risks [21 CFR 50.25(a)(2)] and benefits [21 CFR 50.25(a)(3)]. It is not uncommon for subjects to be paid for their participation in research, especially in the early phases of investigational drug, biologic or device development. Payment to research subjects for participation in studies is not considered a benefit, it is a recruitment incentive. Financial incentives are often used when health benefits to subjects are remote or non-existent. The amount and schedule of all payments should be presented to the IRB at the time of initial review. The IRB should review both the amount of payment and the proposed method and timing of disbursement to assure that neither are coercive or present undue

influence [21 CFR 50.20].

13-1a(3)

Any credit for payment should accrue as the study progresses and not be contingent upon the subject completing the entire study. Unless it creates undue inconvenience or a coercive practice, payment to subjects who withdraw from the study may be made at the time they would have completed the study (or completed a phase of the study) had they not withdrawn. For example, in a study lasting only a few days, an IRB may find it permissible to allow a single payment date at the end of the study, even to subjects who had withdrawn before that date.

While the entire payment should not be contingent upon completion of the entire study, payment of a small proportion as an incentive for completion of the study is acceptable to FDA, providing that such incentive is not coercive. The IRB should determine that the amount paid as a bonus for completion is reasonable and not so large as to unduly induce subjects to stay in the study when they would otherwise have withdrawn. All information concerning payment, including the amount and schedule of payment(s), should be set forth in the informed consent document.

---

*required

13-1a(3)a **Participant Payment**

✓ I will provide compensation to all participants who complete the consent process and begin the study, regardless of whether they complete the study.

I will **NOT** provide compensation to all participants who complete the consent process and begin the study, regardless of whether they complete the study.

---

*required

TTU OP 62.25

13-1a(3)b

All questions concerning participant payment should be directed to the TTU Payroll & Tax Services office or the researcher's business manager.

✓ I will follow the procedures outlined in TTU OP 62.25 regarding participant payment.

I will **NOT** follow the procedures outlined in TTU OP 62.25 regarding participant payment.

No

Conflict of Interest

Guidance: A conflict of interest refers to a situation in which an employee(s) financial, professional, or other personal considerations may directly or indirectly affect, or have the appearance of affecting, the employee(s) judgment in exercising any duty or responsibility, including the conduct or reporting of research, owed to the institution (TTU OP 10.20 (2)(d)).

14-1

"Significant business or financial interest" means anything of monetary value including, but not limited to, salary or other payments for services (e.g., consulting fees or honorarium; equity interests (e.g., stocks, stock options, or other ownership interests); and intellectual property rights (e.g., patents, copyrights, and royalties from such rights). (TTU OP 74.17).

"...a set of conditions in which an investigator's judgment concerning a primary interest (e.g., subject welfare, integrity of research) could be biased by a secondary interest (e.g., personal or financial gain)." Institutional Review Board Management and Function, Bankert and Amdur, 2006, p. 167.

*required

14-1a **Does anyone on the research team have a potential conflict of interest with the research project?**

Yes

✓ No

15: Investigator Attestations

*required

I, as the PI and co-investigators, understand Texas Tech University’s policy concerning research involving human subjects and by checking below, I certify:

- ✓ I have read The Belmont Report “Ethical Principles and Guidelines for the Protection of Human Subjects of Research” and subscribe to the principles it contains.
- ✓ I have read 45 CFR 46, Protection of Human Subjects and subscribe to the regulations it contains.
- ✓ I accept responsibility for the scientific and ethical conduct of this research study and understand my responsibility.
- ✓ I will ensure that all study personnel are appropriately trained and are competent to perform the study.
- ✓ I will obtain prior approval from the Institutional Review Board (IRB) before making any modifications of the previously approved research, including modifications to the informed consent process and document.
- ✓ I will submit a Study Closure Submission upon completion of this study and agree to honor any other commitments.

✓ I will follow TTU procedures and guidelines to protect the health and well-being of research participants, research team members, and liaison(s) during any unforeseen circumstances.

✓ I will immediately report to the IRB any deviations, violations, non-compliance, unanticipated problems, and adverse events related to subjects or others which occurred or possibly occurred as a result of this study.

45 CFR 46.103(a) and TTU IRB Policy 5.4 require Adverse Events, Unanticipated Problems and Deviations from the research protocol to be reported **IMMEDIATELY** to the HRPP office.

Human Research Protection Program
Box 41075
Lubbock, Texas 79409
Phone: (806) 742-2064
Email: hrpp@ttu.edu

16: Additional Information

16-1 **Include any additional points to help clarify the research, if applicable.**

16-1a

16-2 **Enter or attach any additional documentation.**

16-2a
